# Supplementary figures and images for: Gli1 labels progenitors during chondrogenesis in postnatal mice (part 1 of 3)
Source: EMBO Rep. 2024 Feb 26;25(4):12. doi: 10.1038/s44319-024-00093-x (PMC11014955; doi:10.1038/s44319-024-00093-x)

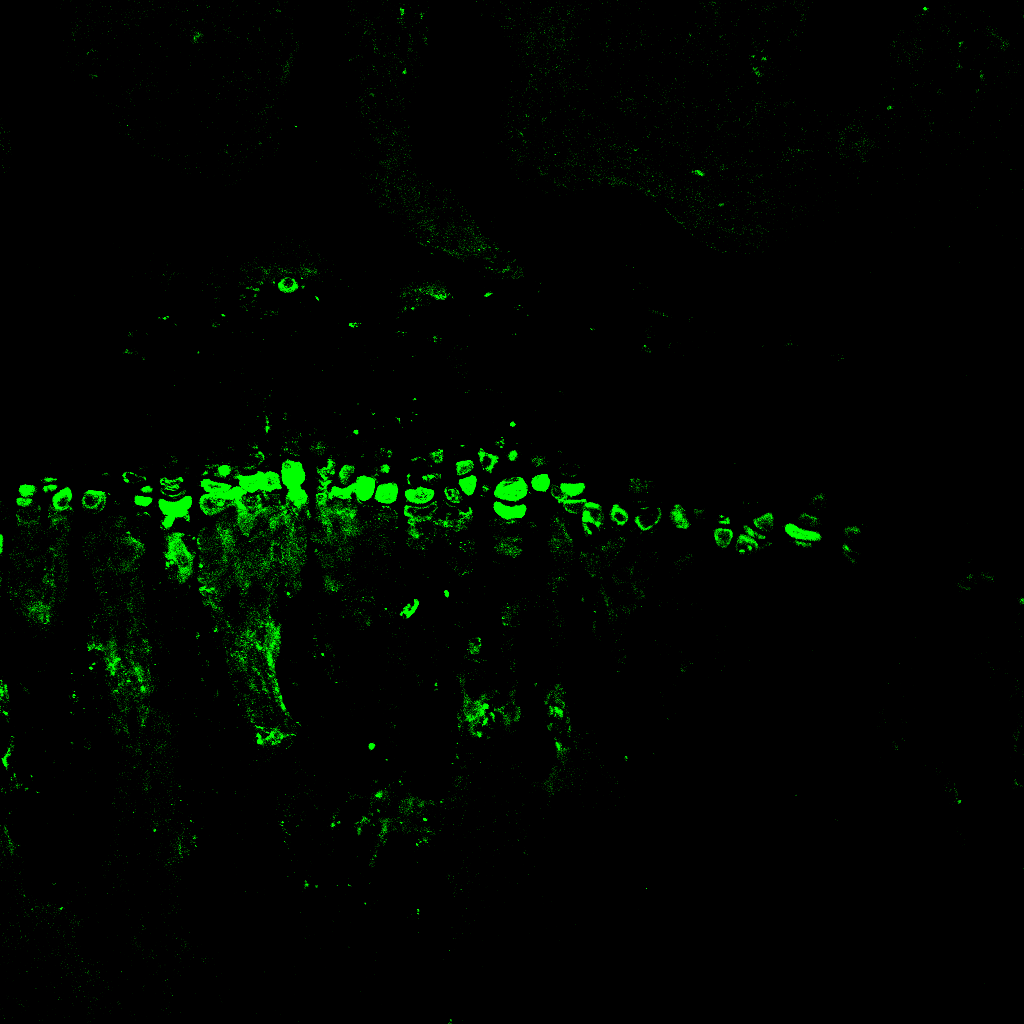

Supplement: Supplementary file 1 — Source Data Fig. 1 [file 44319_2024_93_MOESM1_ESM.zip › Figure1/1A/1month/TM1M_1mo_GP_acan_green.tif]

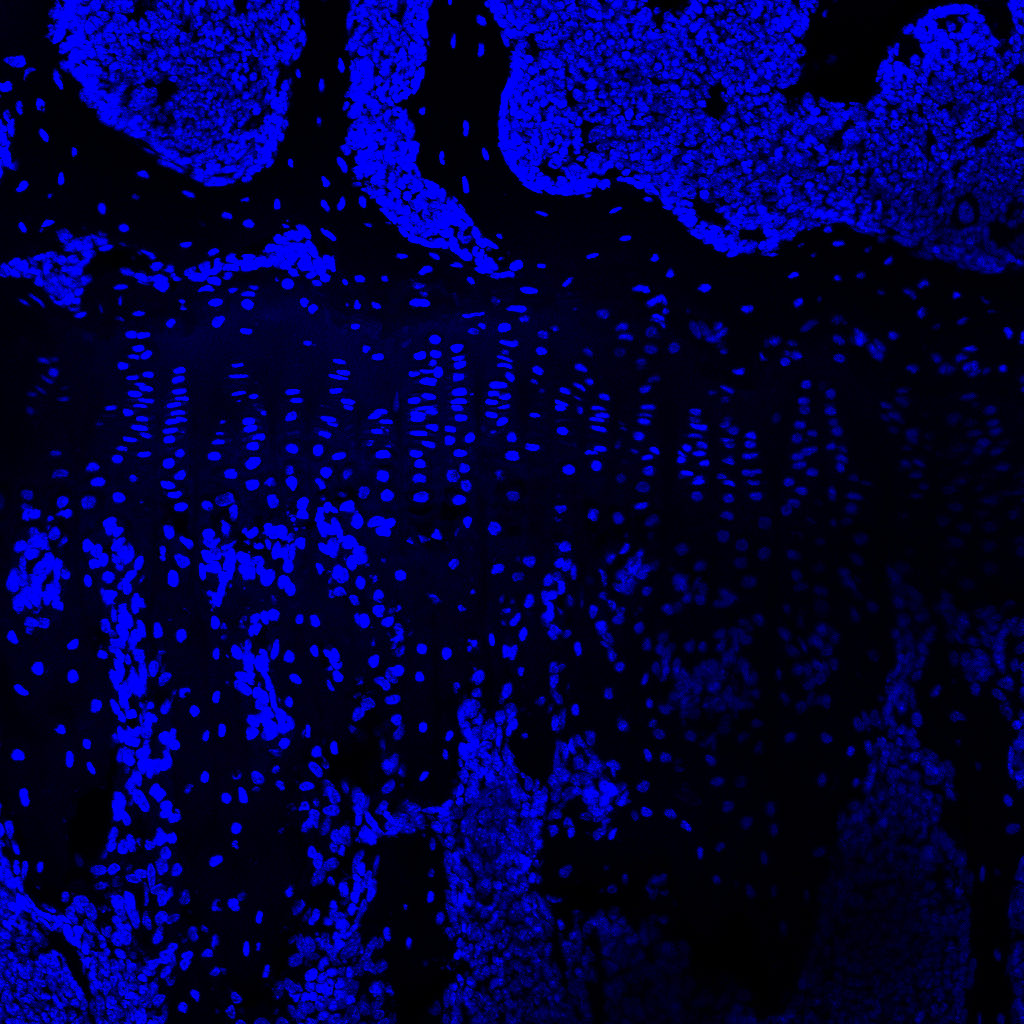

Supplement: Supplementary file 1 — Source Data Fig. 1 [file 44319_2024_93_MOESM1_ESM.zip › Figure1/1A/1month/TM1M_1mo_GP_dapi_blue.tif]

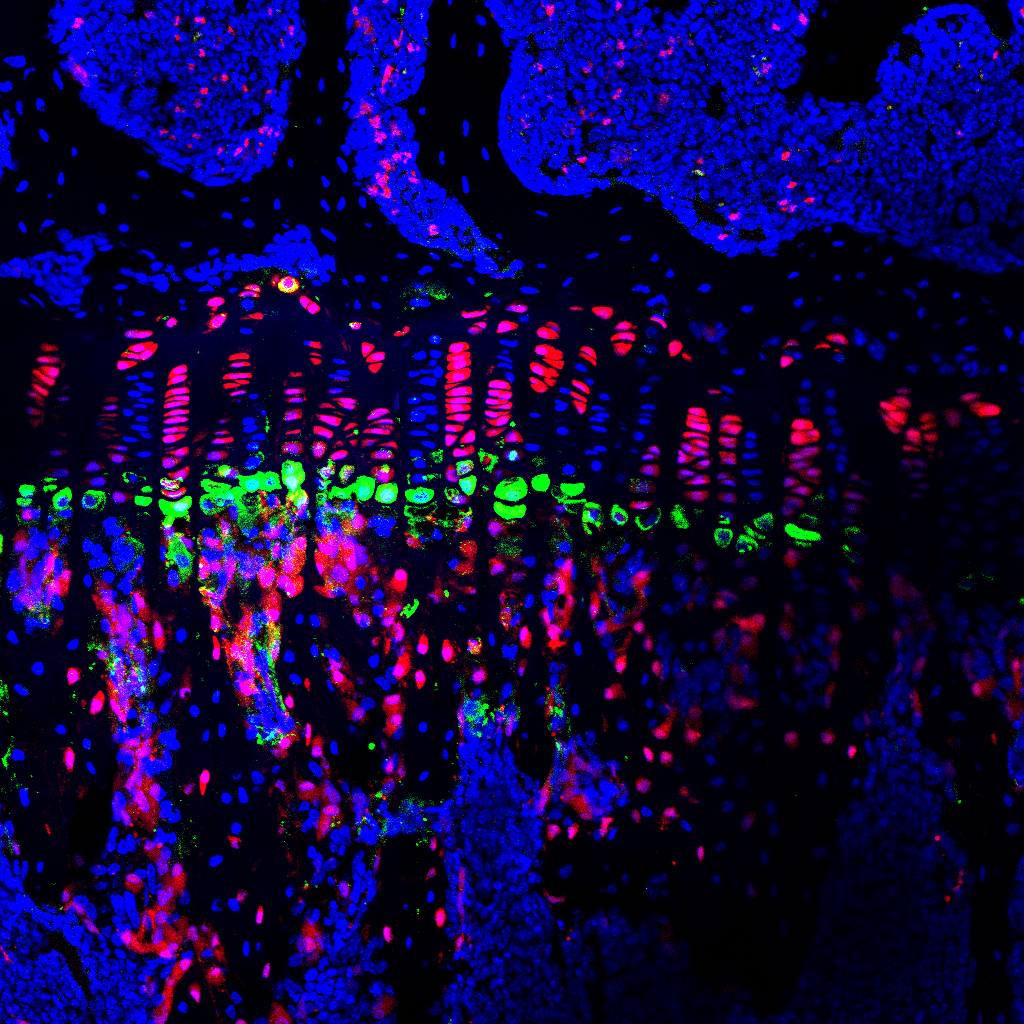

Supplement: Supplementary file 1 — Source Data Fig. 1 [file 44319_2024_93_MOESM1_ESM.zip › Figure1/1A/1month/TM1M_1mo_GP_merge.tif]

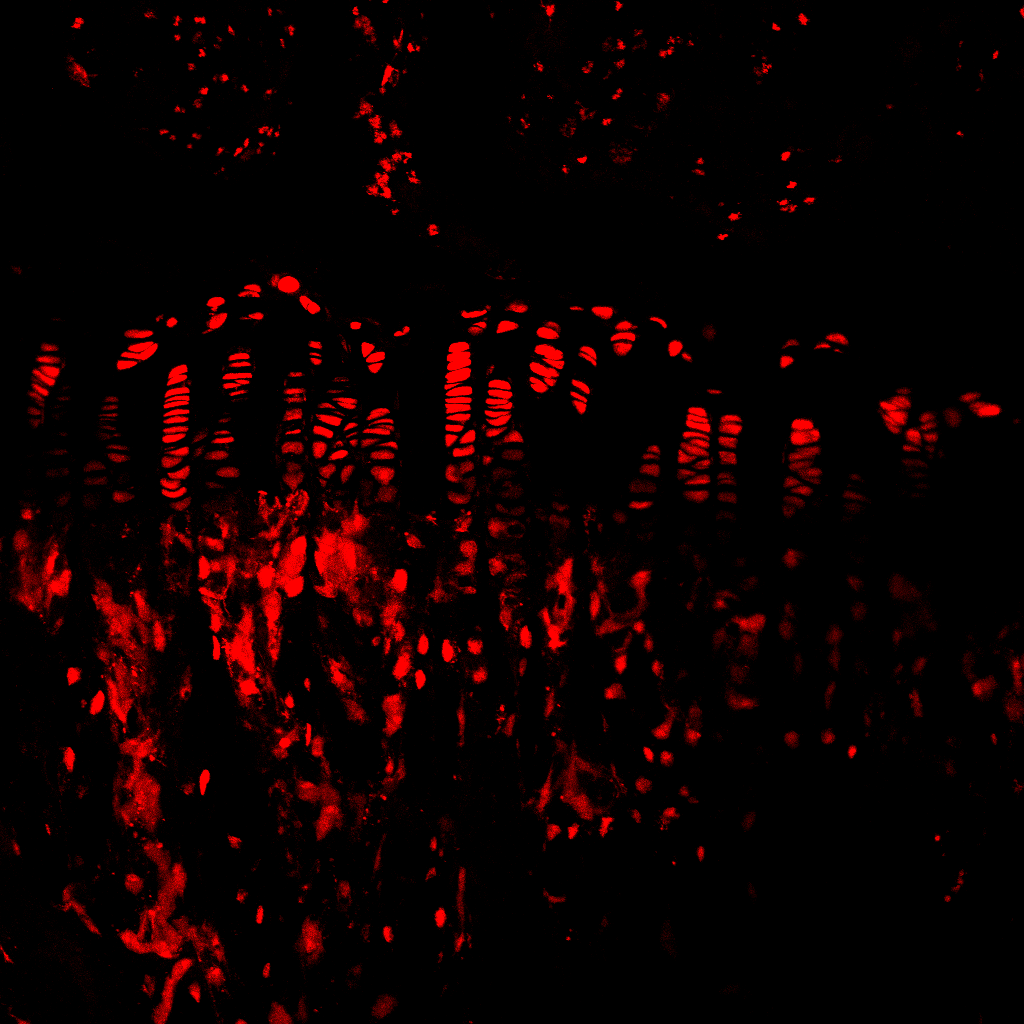

Supplement: Supplementary file 1 — Source Data Fig. 1 [file 44319_2024_93_MOESM1_ESM.zip › Figure1/1A/1month/TM1M_1mo_GP_td_red.tif]

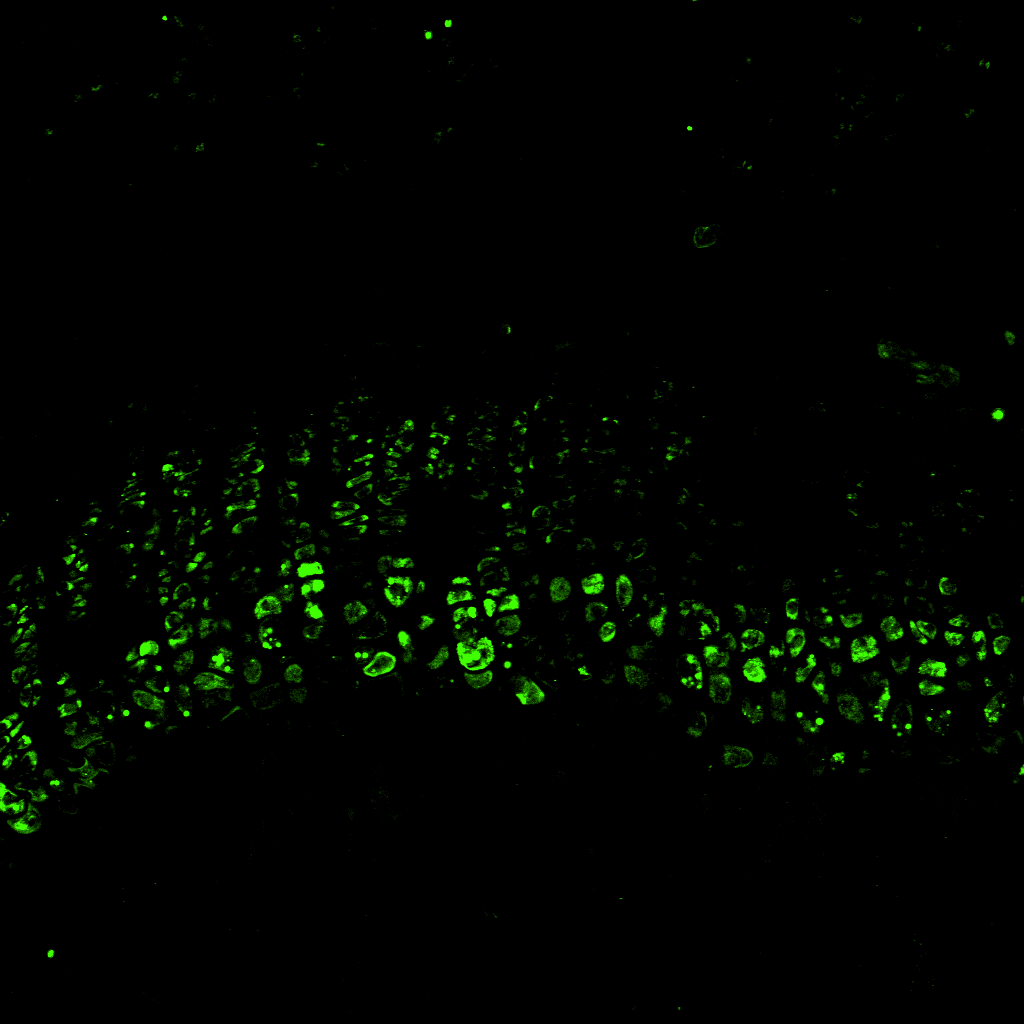

Supplement: Supplementary file 1 — Source Data Fig. 1 [file 44319_2024_93_MOESM1_ESM.zip › Figure1/1A/Day1/TM1M_D1_GP_acan_green.tif]

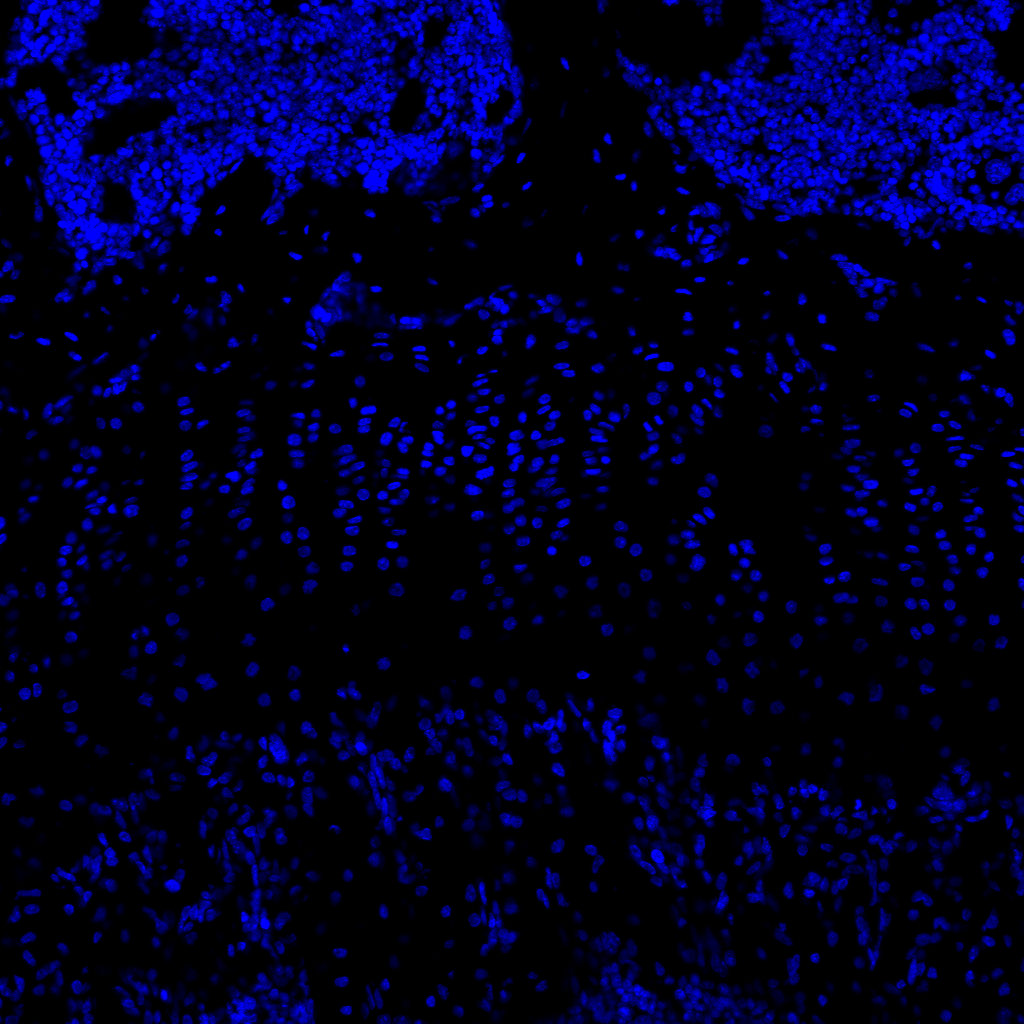

Supplement: Supplementary file 1 — Source Data Fig. 1 [file 44319_2024_93_MOESM1_ESM.zip › Figure1/1A/Day1/TM1M_D1_GP_dapi_blue.tif]

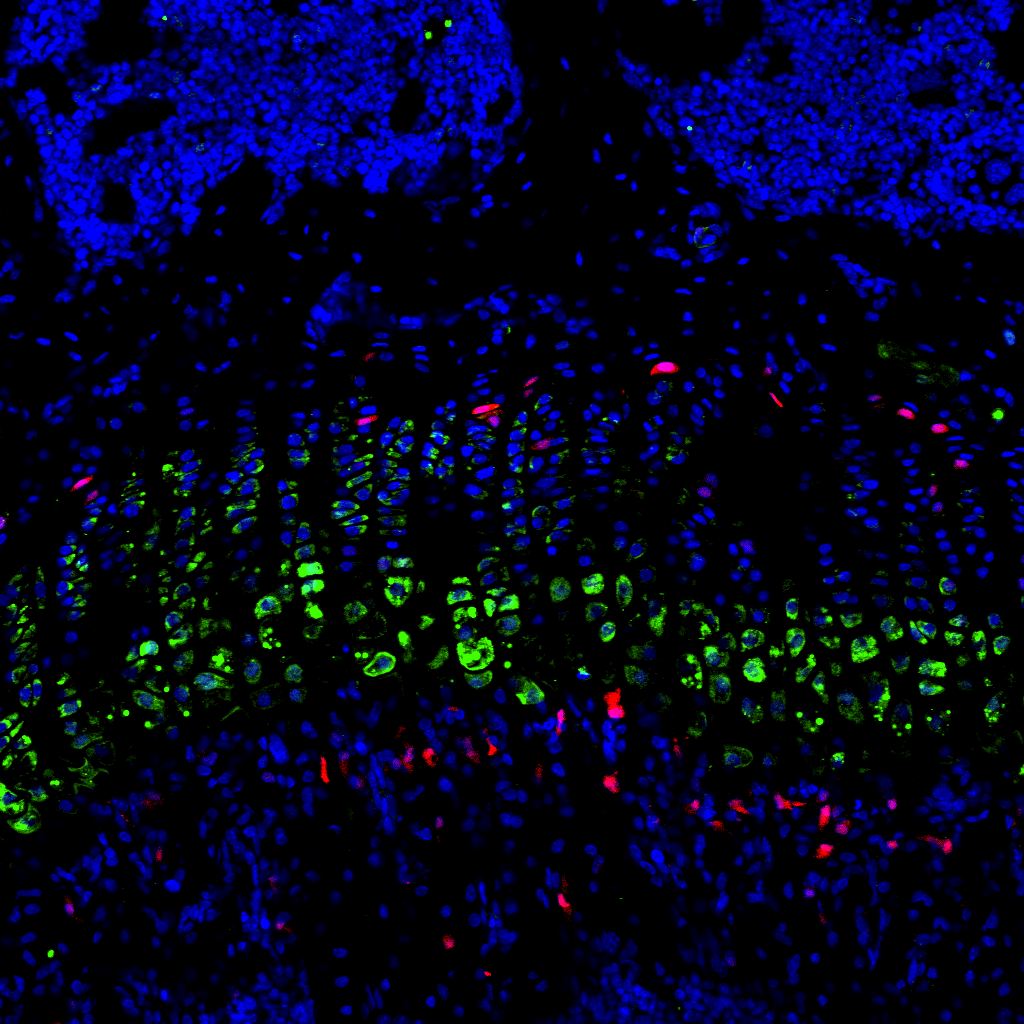

Supplement: Supplementary file 1 — Source Data Fig. 1 [file 44319_2024_93_MOESM1_ESM.zip › Figure1/1A/Day1/TM1M_D1_GP_merge.tif]

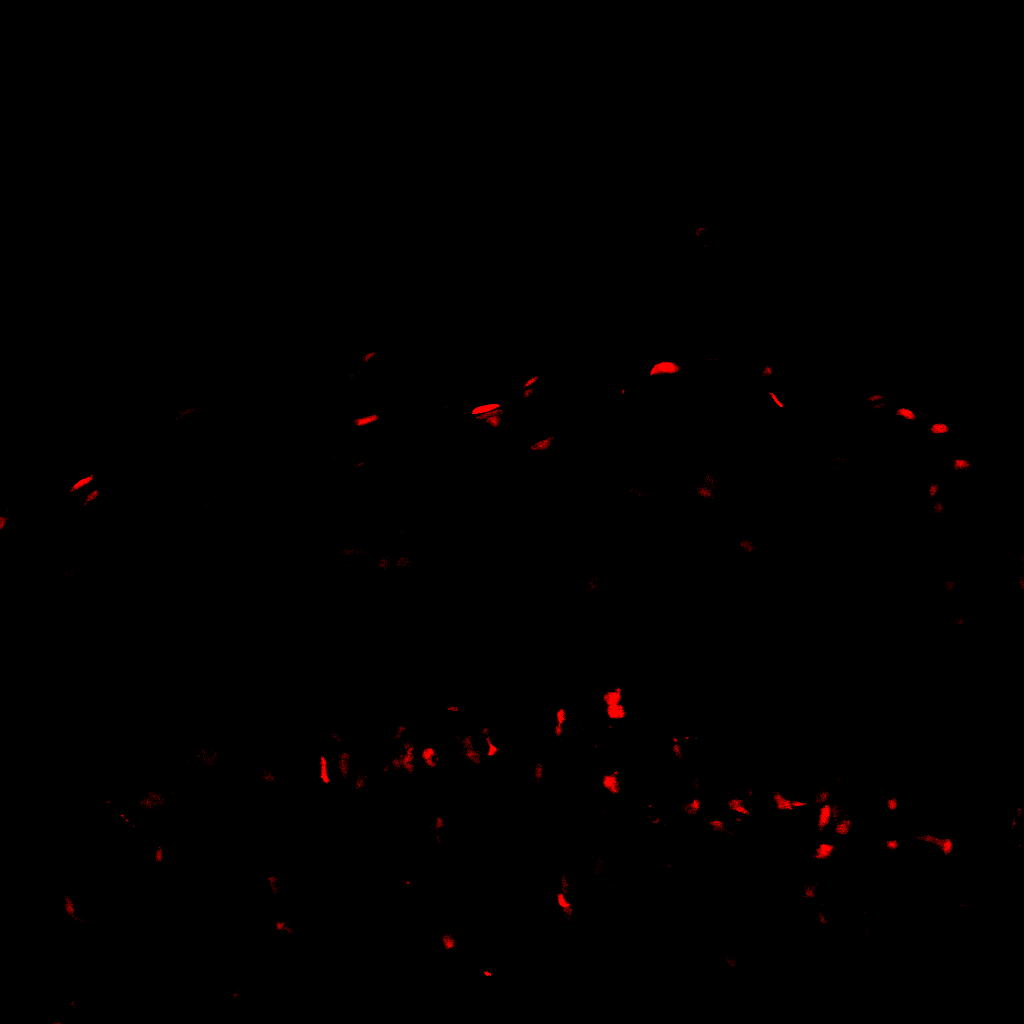

Supplement: Supplementary file 1 — Source Data Fig. 1 [file 44319_2024_93_MOESM1_ESM.zip › Figure1/1A/Day1/TM1M_D1_GP_td_red.tif]

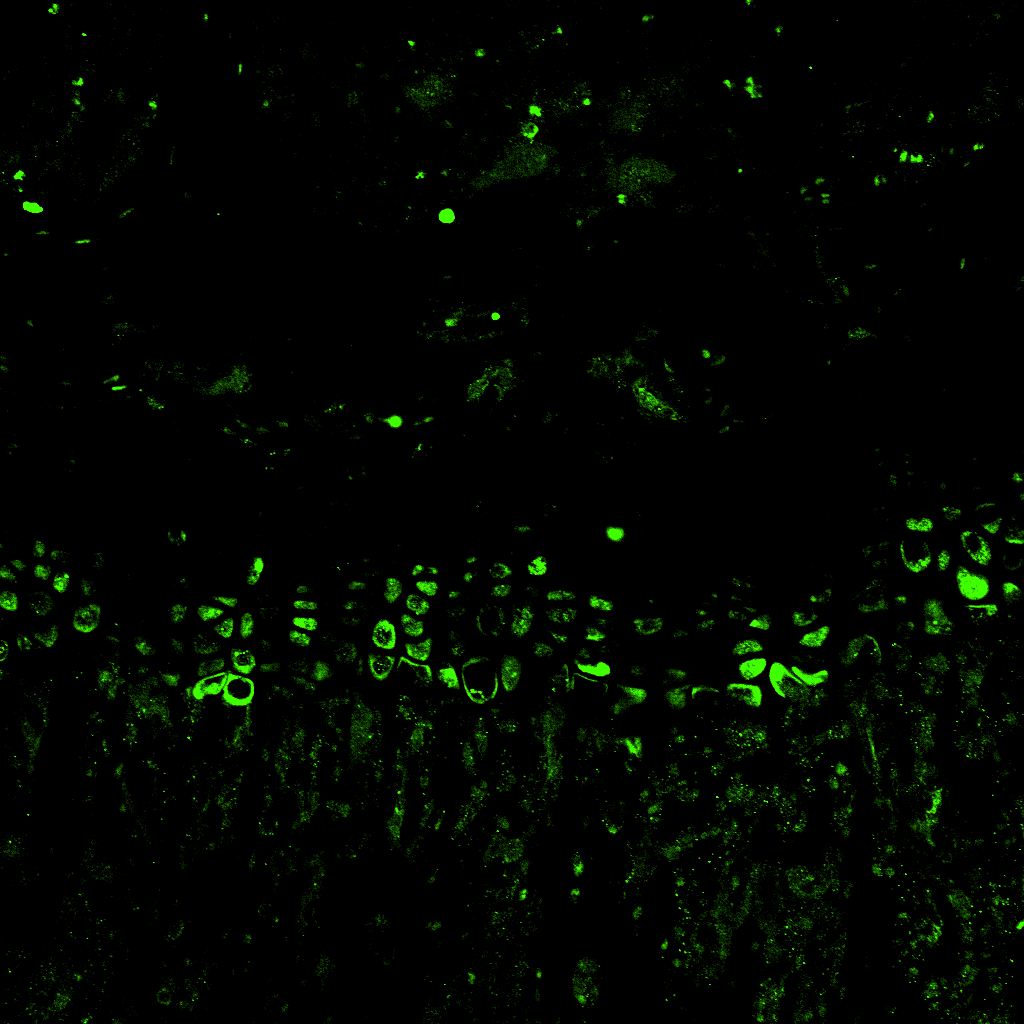

Supplement: Supplementary file 1 — Source Data Fig. 1 [file 44319_2024_93_MOESM1_ESM.zip › Figure1/1A/Day14/TM1M_D14_GP_acan_green.tif]

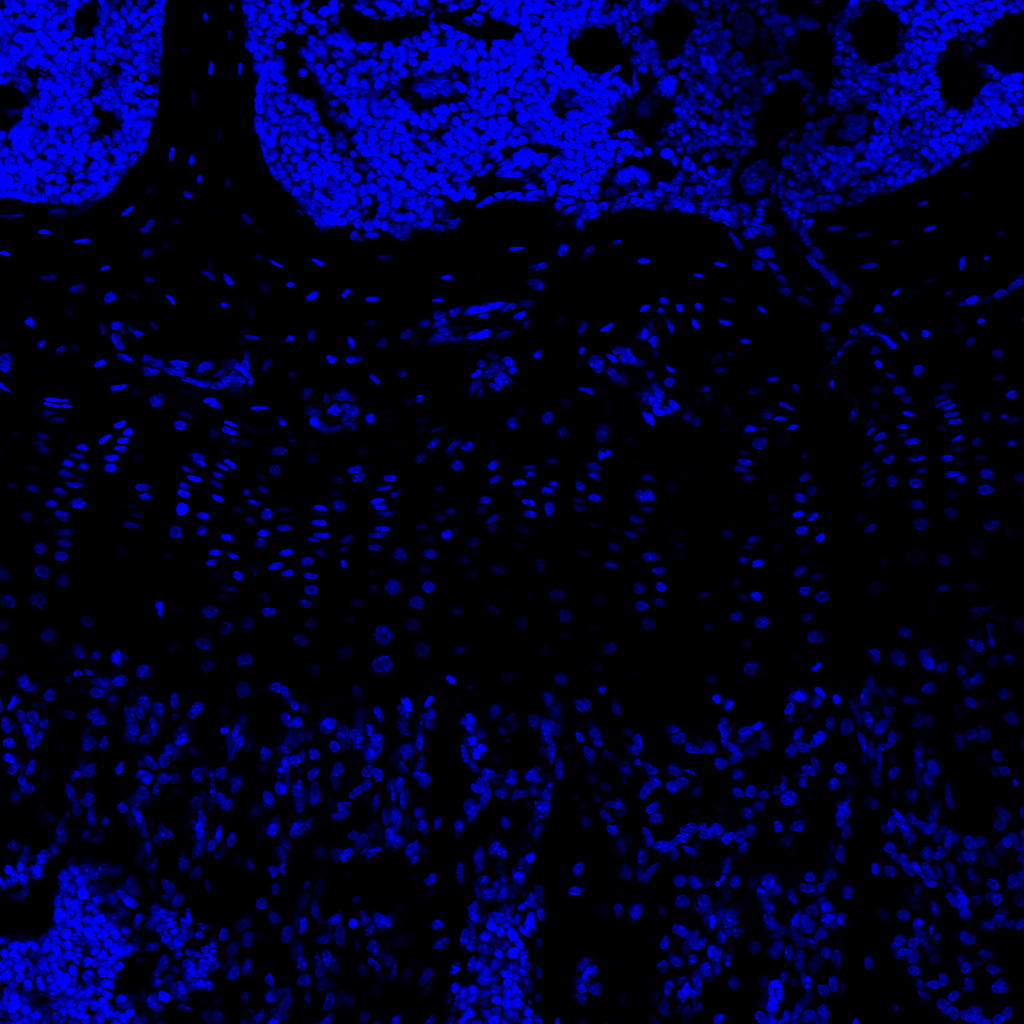

Supplement: Supplementary file 1 — Source Data Fig. 1 [file 44319_2024_93_MOESM1_ESM.zip › Figure1/1A/Day14/TM1M_D14_GP_dapi_blue.tif]

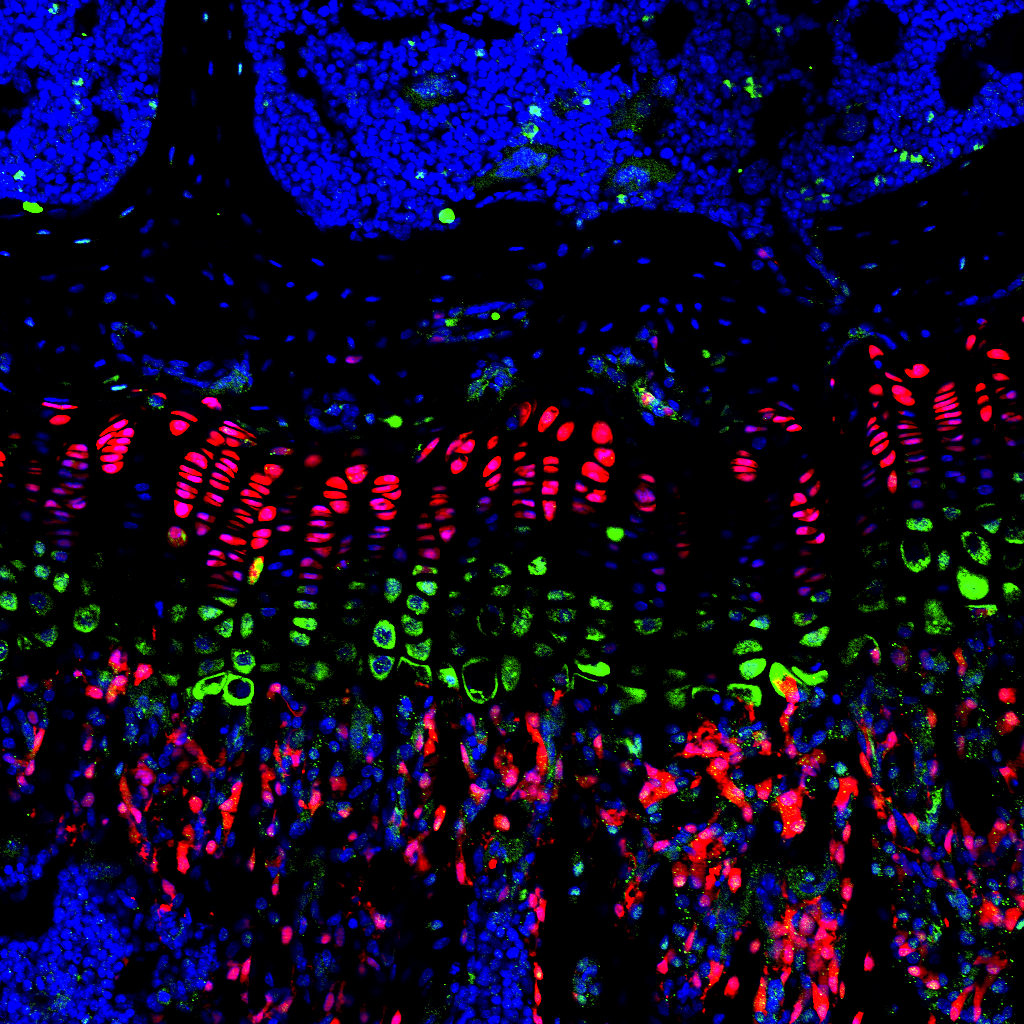

Supplement: Supplementary file 1 — Source Data Fig. 1 [file 44319_2024_93_MOESM1_ESM.zip › Figure1/1A/Day14/TM1M_D14_GP_merge.tif]

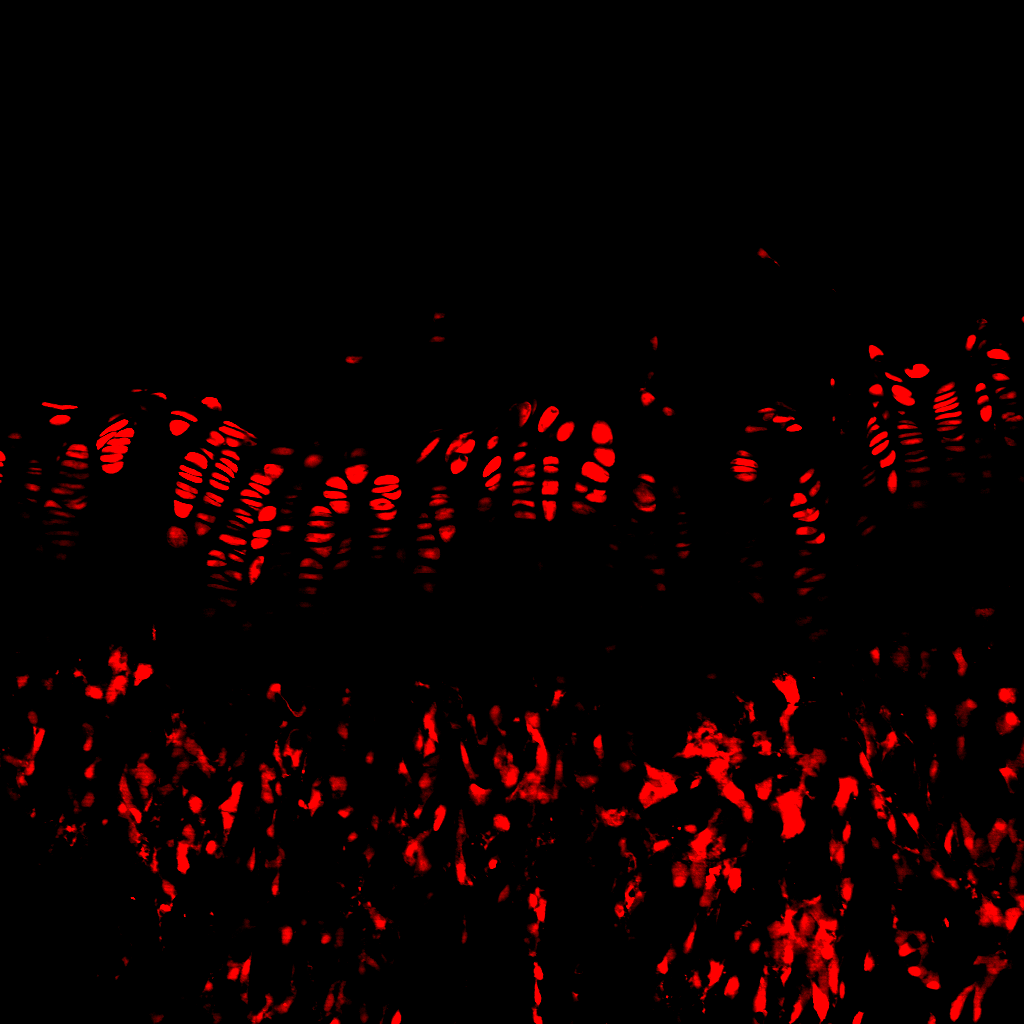

Supplement: Supplementary file 1 — Source Data Fig. 1 [file 44319_2024_93_MOESM1_ESM.zip › Figure1/1A/Day14/TM1M_D14_GP_td_red.tif]

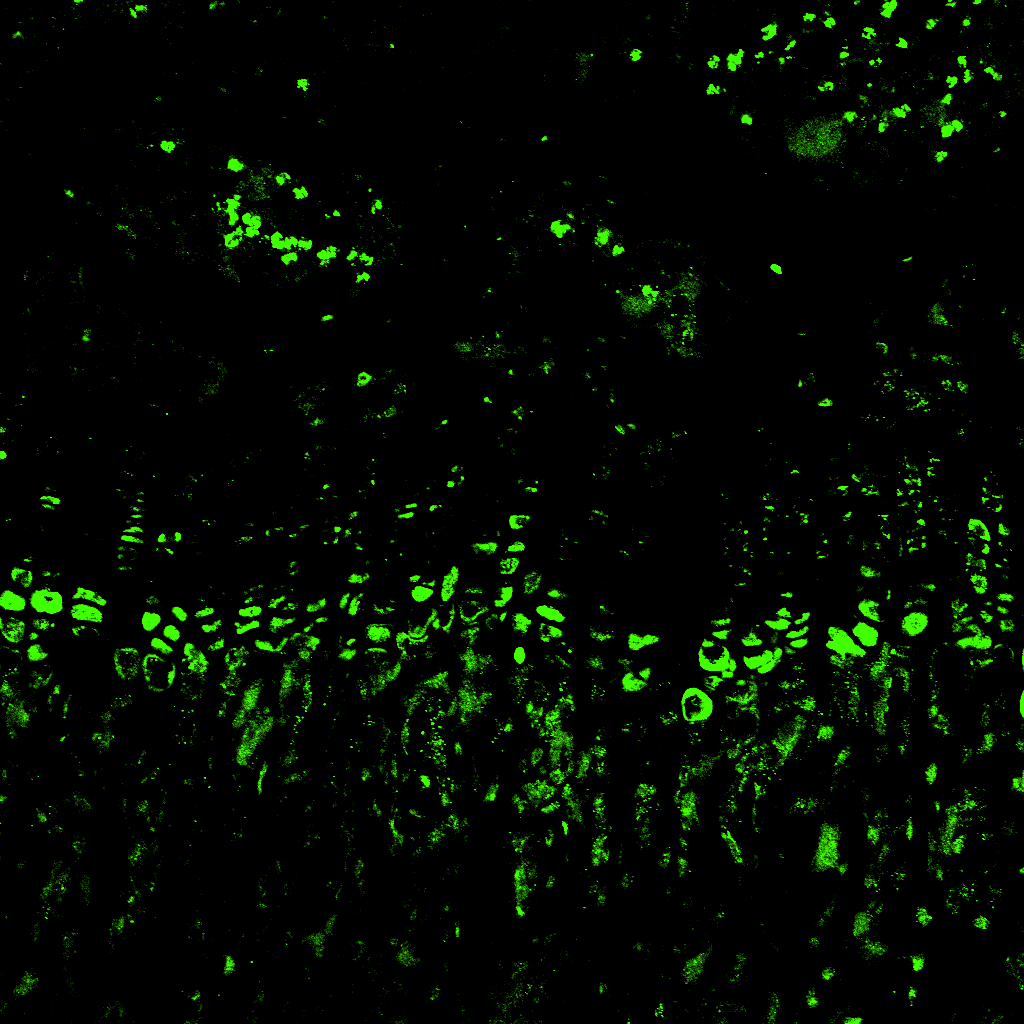

Supplement: Supplementary file 1 — Source Data Fig. 1 [file 44319_2024_93_MOESM1_ESM.zip › Figure1/1A/Day21/TM1M_D21_GP_acan_green.tif]

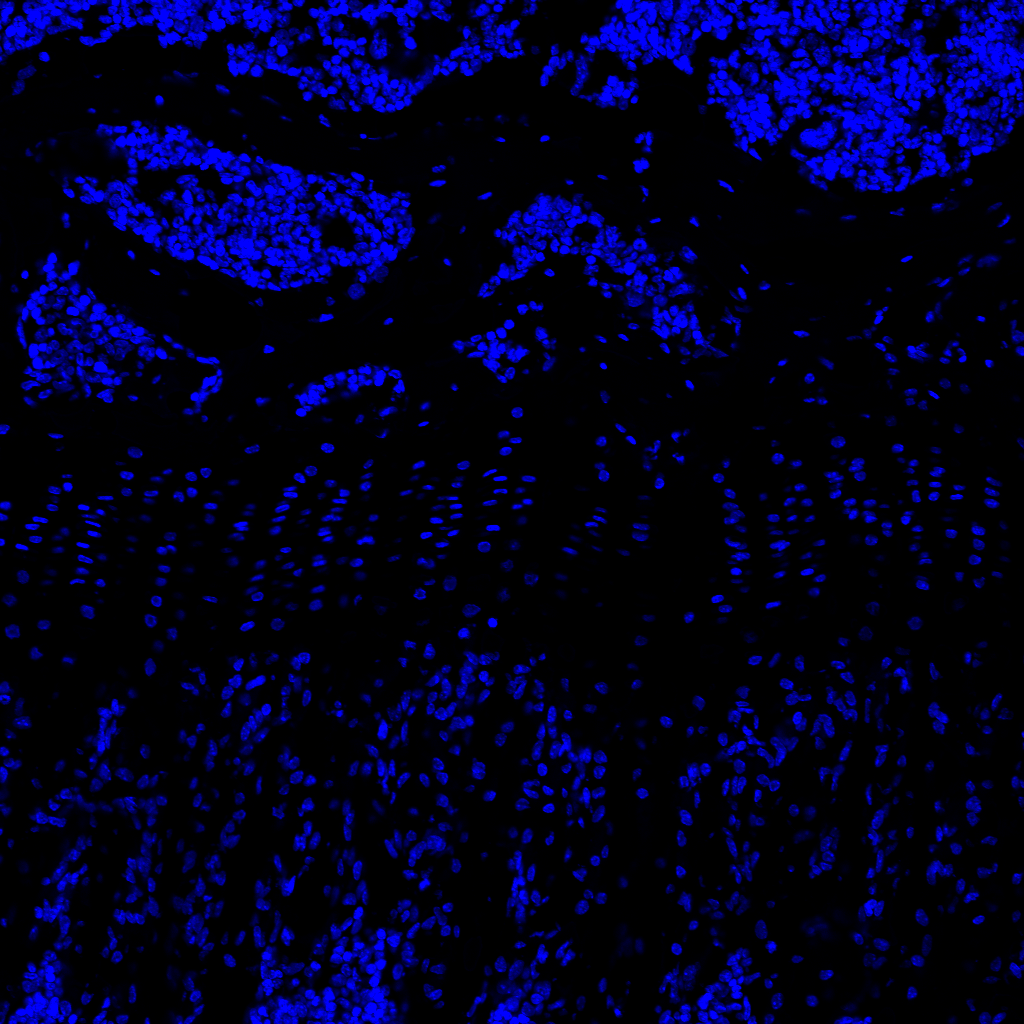

Supplement: Supplementary file 1 — Source Data Fig. 1 [file 44319_2024_93_MOESM1_ESM.zip › Figure1/1A/Day21/TM1M_D21_GP_dapi_blue.tif]

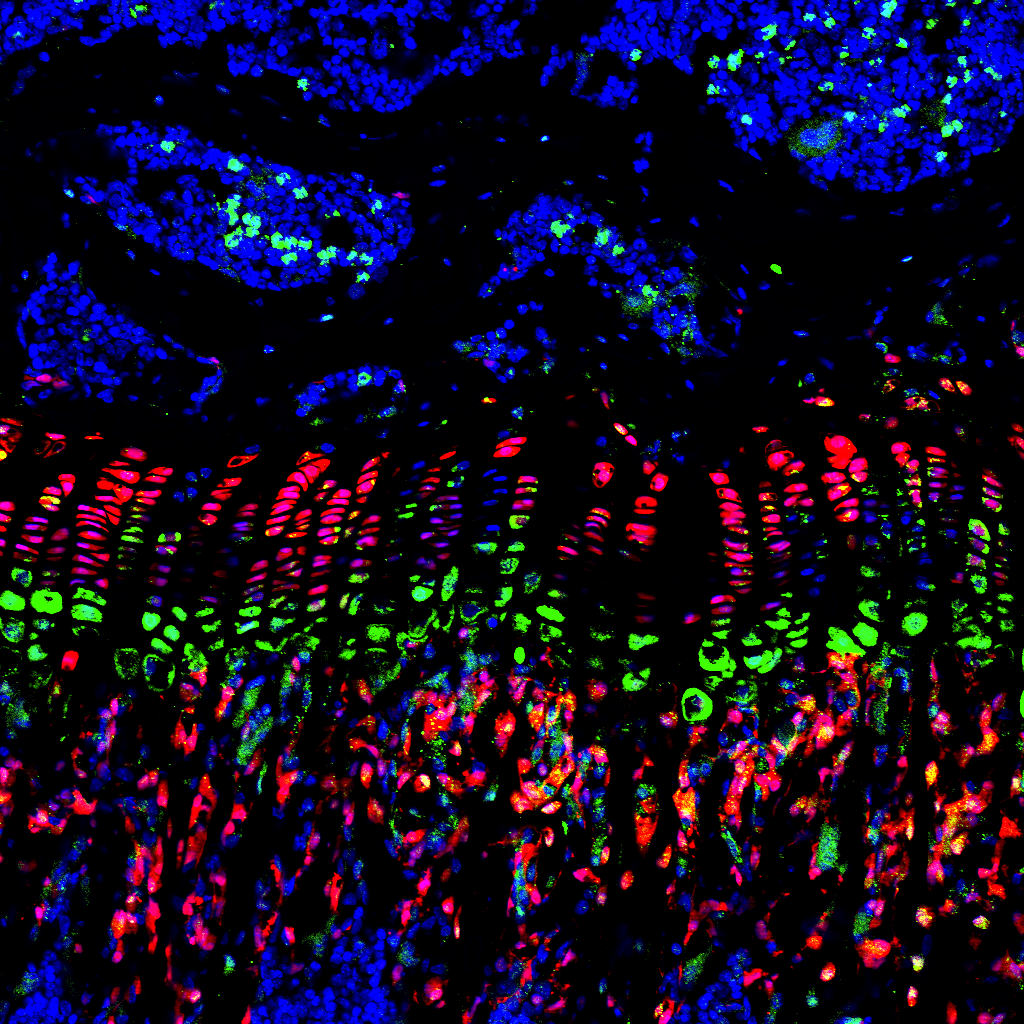

Supplement: Supplementary file 1 — Source Data Fig. 1 [file 44319_2024_93_MOESM1_ESM.zip › Figure1/1A/Day21/TM1M_D21_GP_merge.tif]

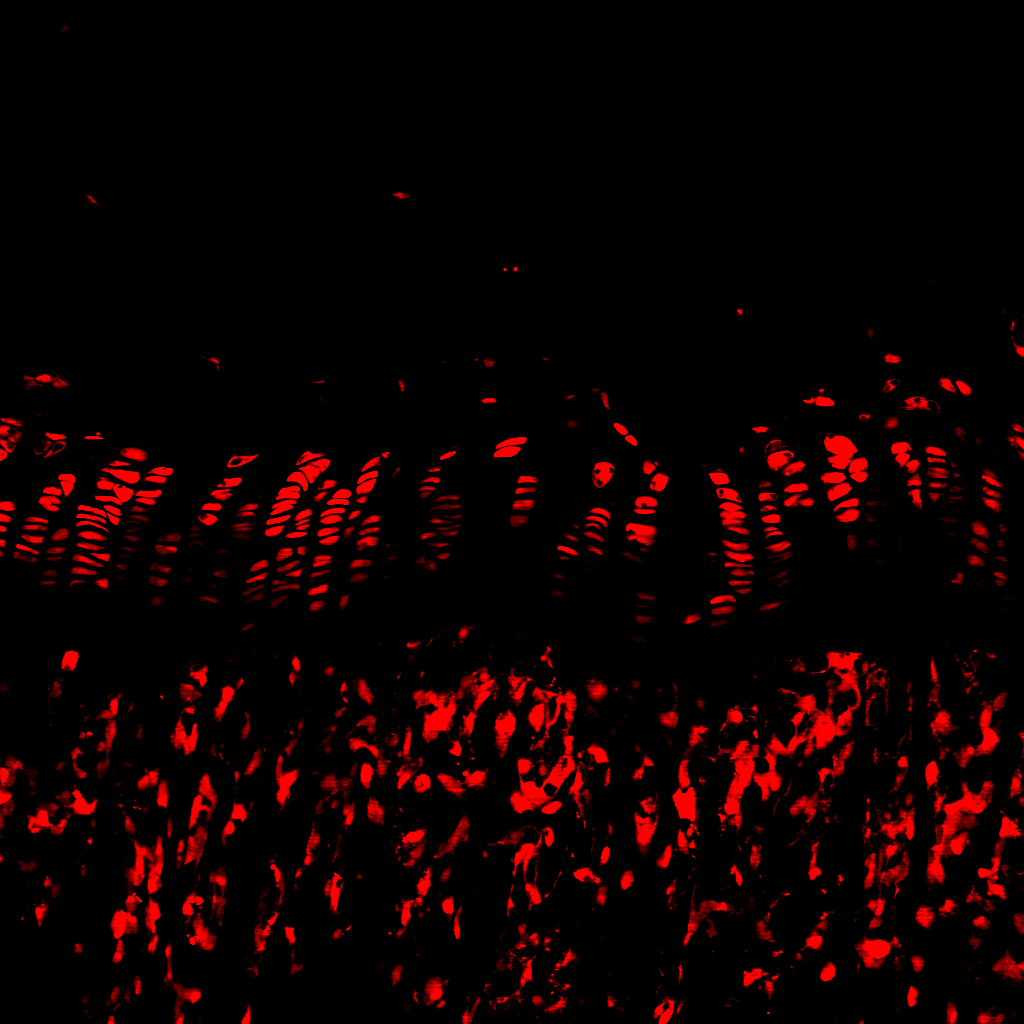

Supplement: Supplementary file 1 — Source Data Fig. 1 [file 44319_2024_93_MOESM1_ESM.zip › Figure1/1A/Day21/TM1M_D21_GP_td_red.tif]

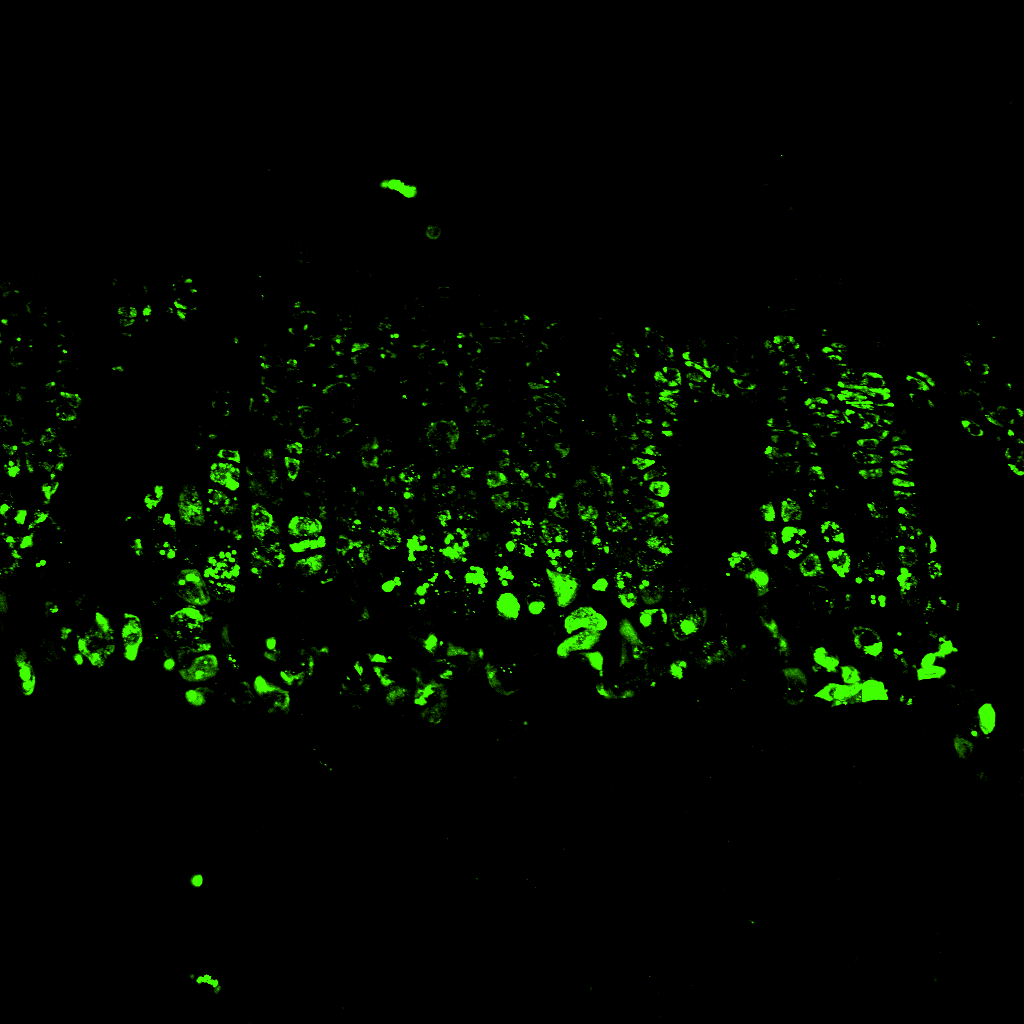

Supplement: Supplementary file 1 — Source Data Fig. 1 [file 44319_2024_93_MOESM1_ESM.zip › Figure1/1A/Day3/TM1M_D3_GP_acan_green.tif]

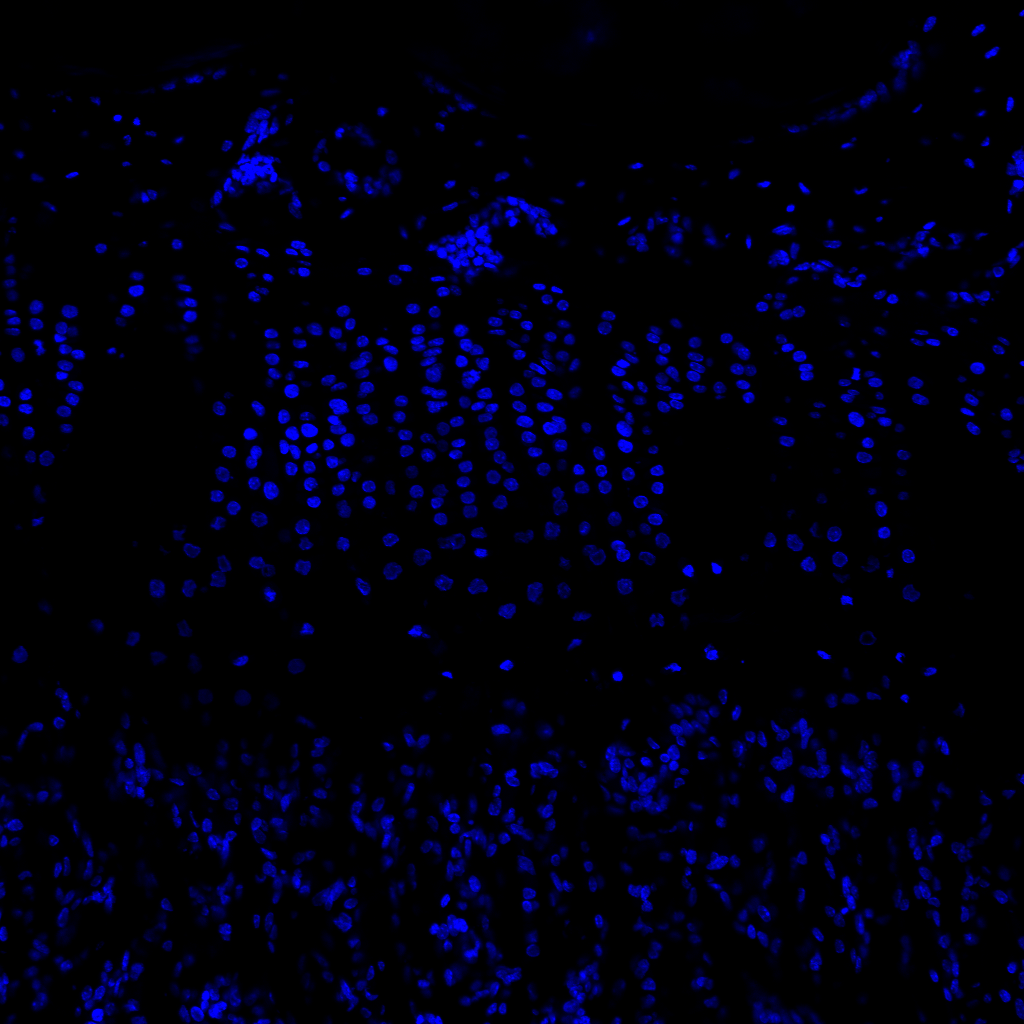

Supplement: Supplementary file 1 — Source Data Fig. 1 [file 44319_2024_93_MOESM1_ESM.zip › Figure1/1A/Day3/TM1M_D3_GP_dapi_blue.tif]

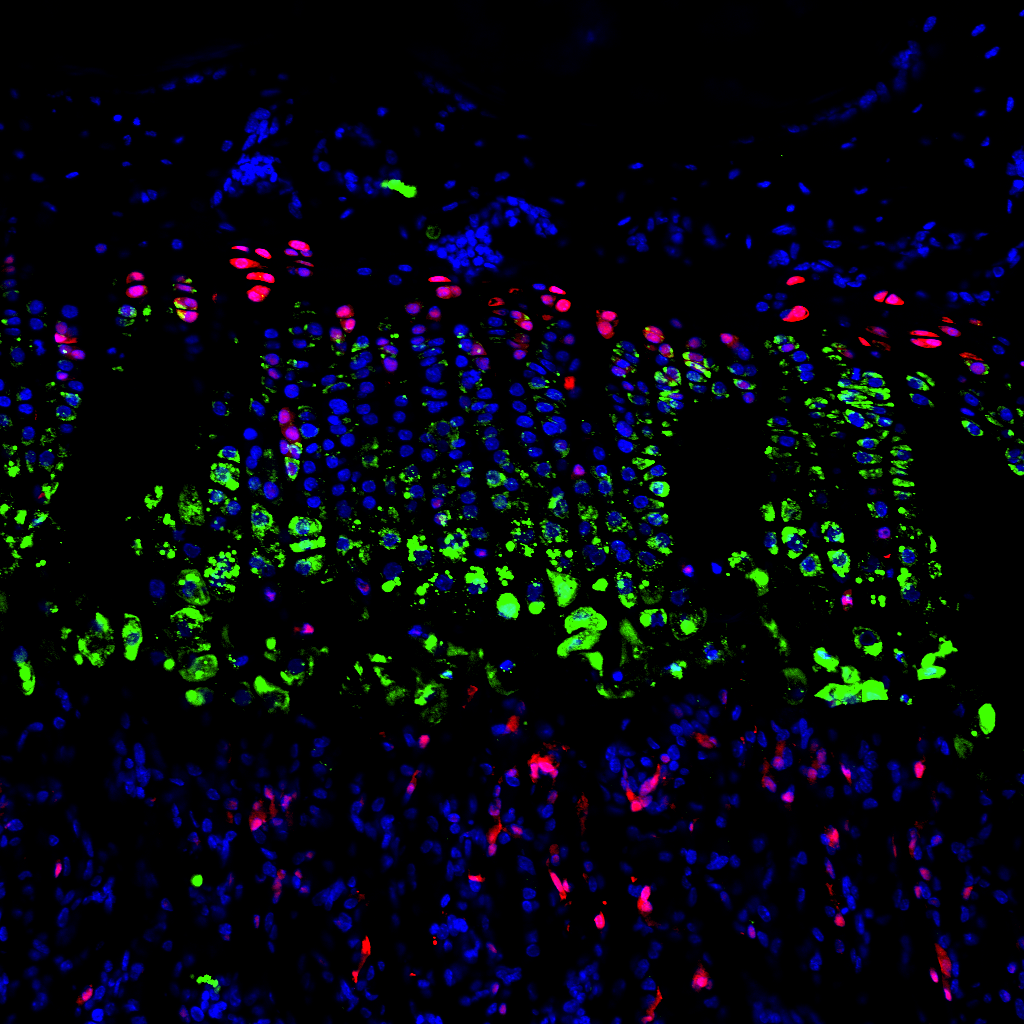

Supplement: Supplementary file 1 — Source Data Fig. 1 [file 44319_2024_93_MOESM1_ESM.zip › Figure1/1A/Day3/TM1M_D3_GP_merge.tif]

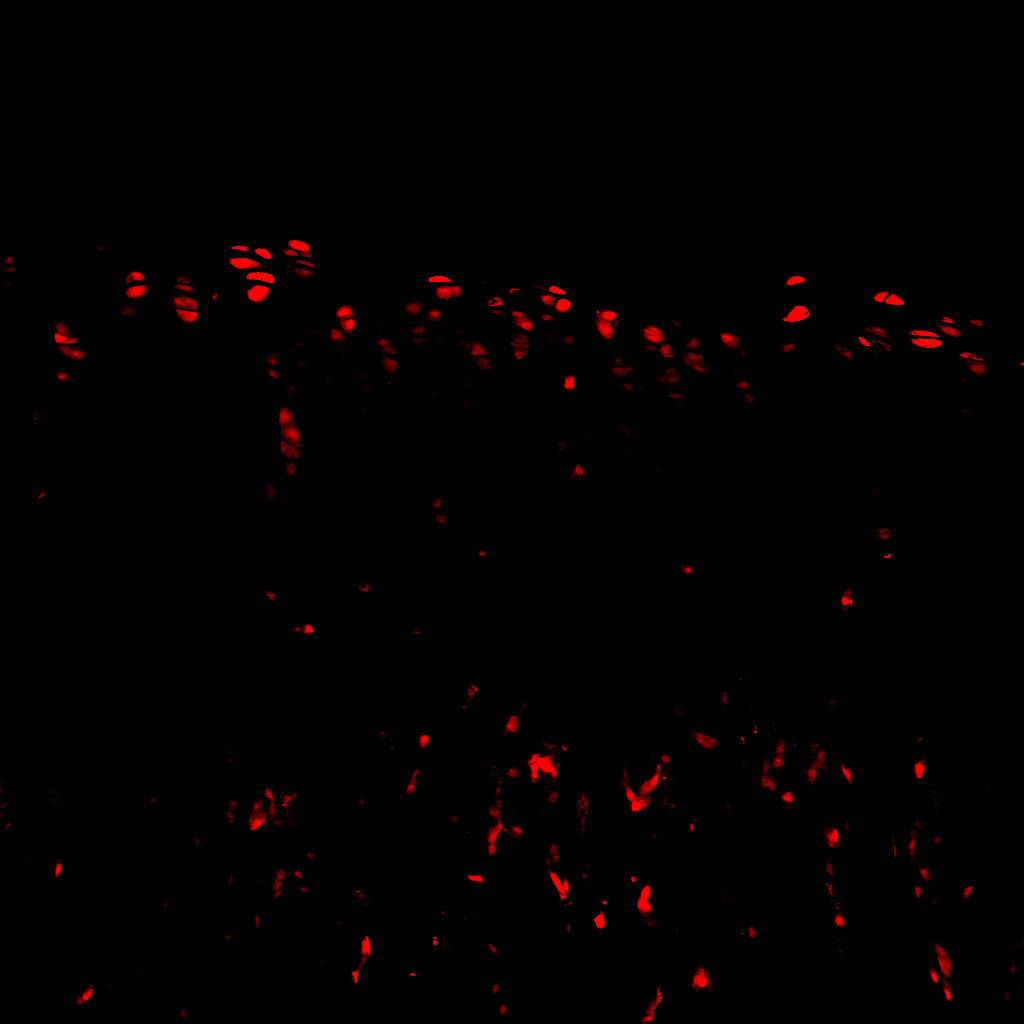

Supplement: Supplementary file 1 — Source Data Fig. 1 [file 44319_2024_93_MOESM1_ESM.zip › Figure1/1A/Day3/TM1M_D3_GP_td_red.tif]

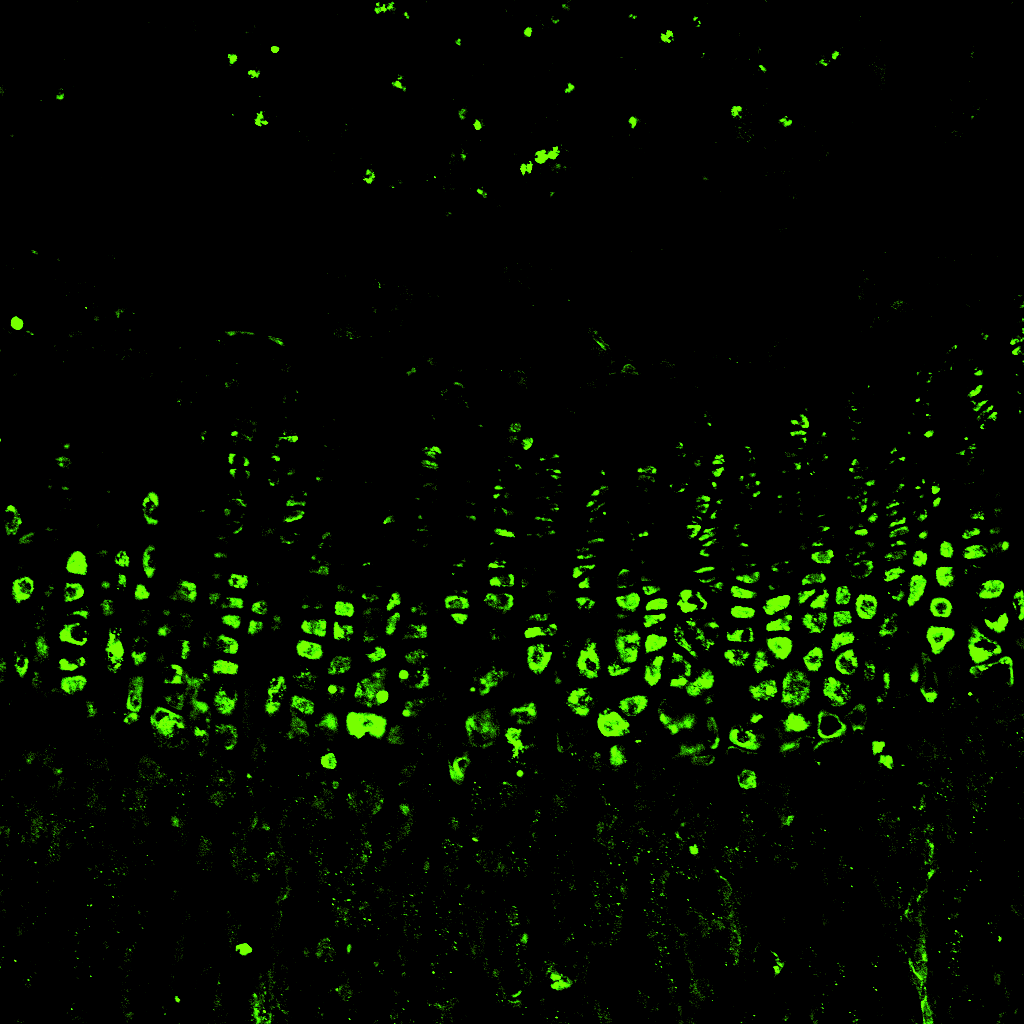

Supplement: Supplementary file 1 — Source Data Fig. 1 [file 44319_2024_93_MOESM1_ESM.zip › Figure1/1A/Day7/TM1M_D7_GP_acan_green.tif]

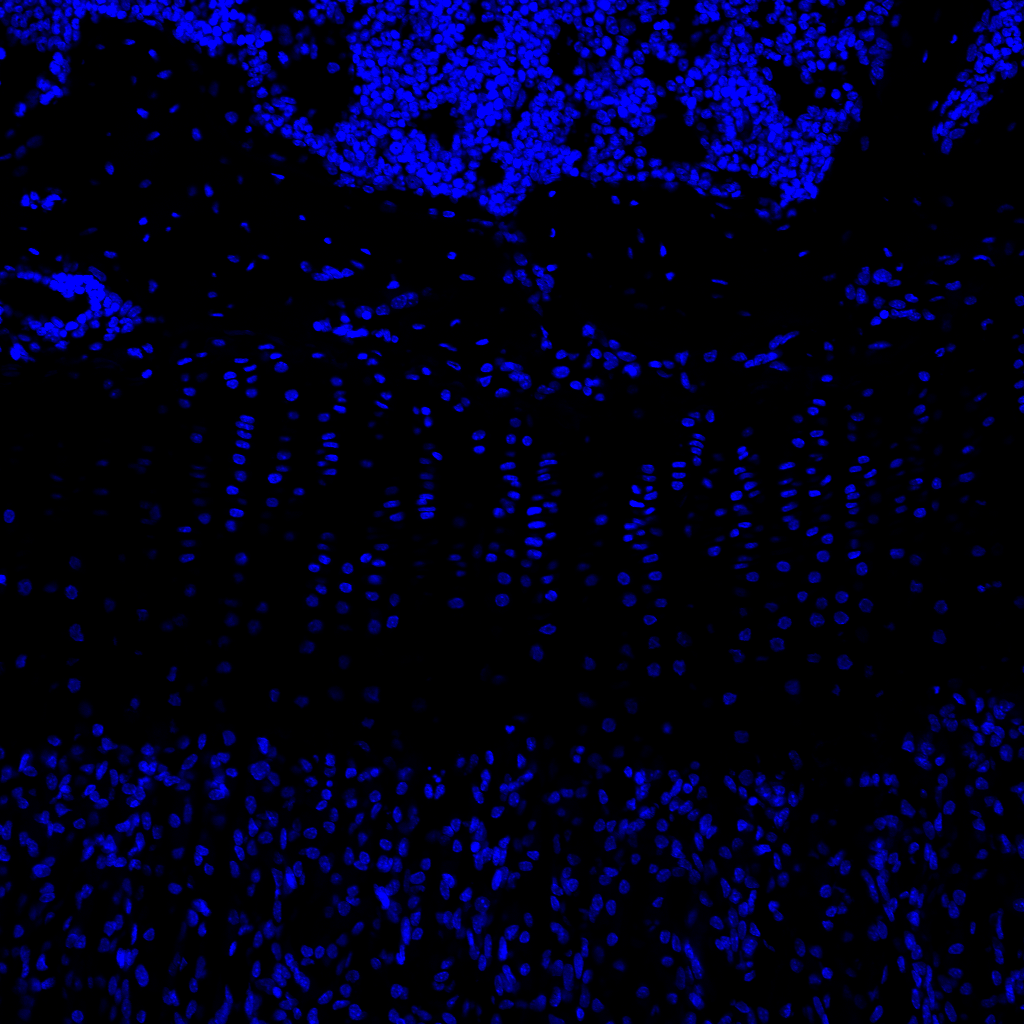

Supplement: Supplementary file 1 — Source Data Fig. 1 [file 44319_2024_93_MOESM1_ESM.zip › Figure1/1A/Day7/TM1M_D7_GP_dapi_blue.tif]

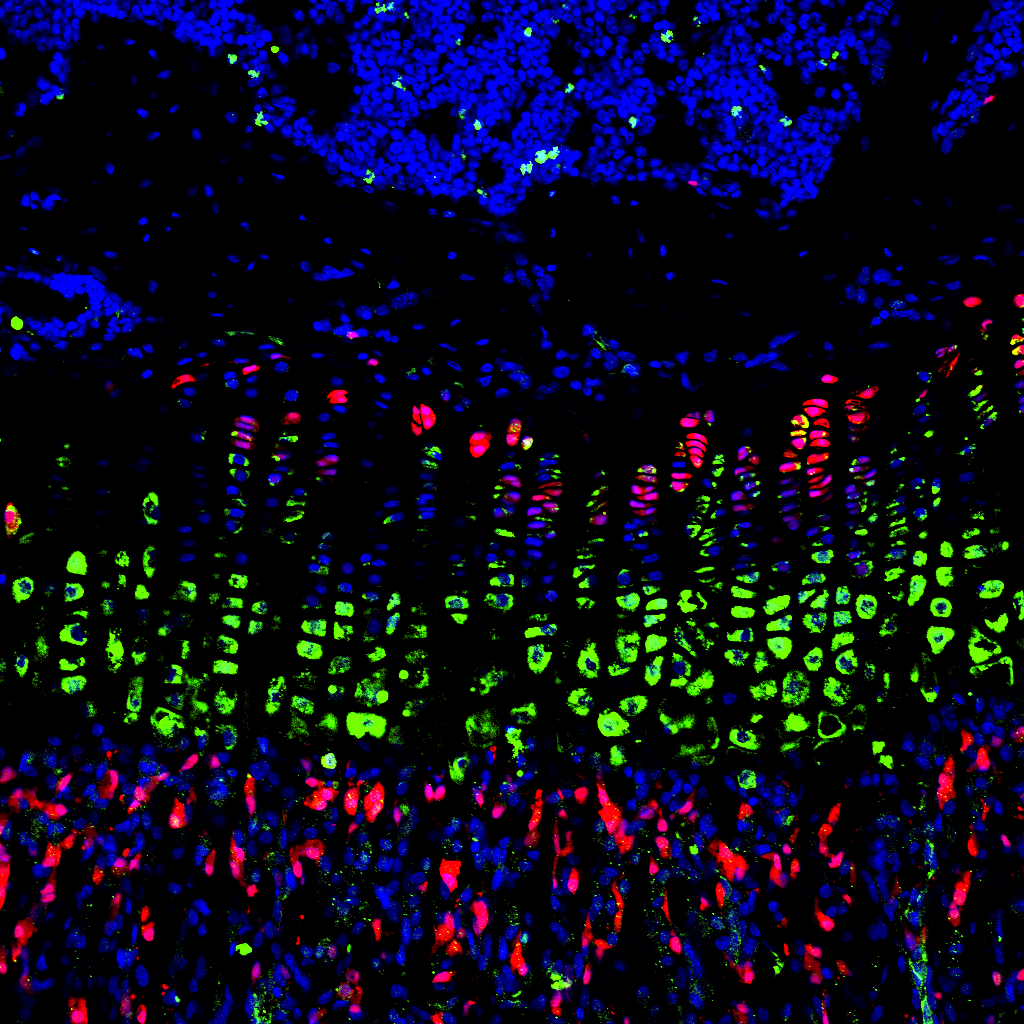

Supplement: Supplementary file 1 — Source Data Fig. 1 [file 44319_2024_93_MOESM1_ESM.zip › Figure1/1A/Day7/TM1M_D7_GP_merge.tif]

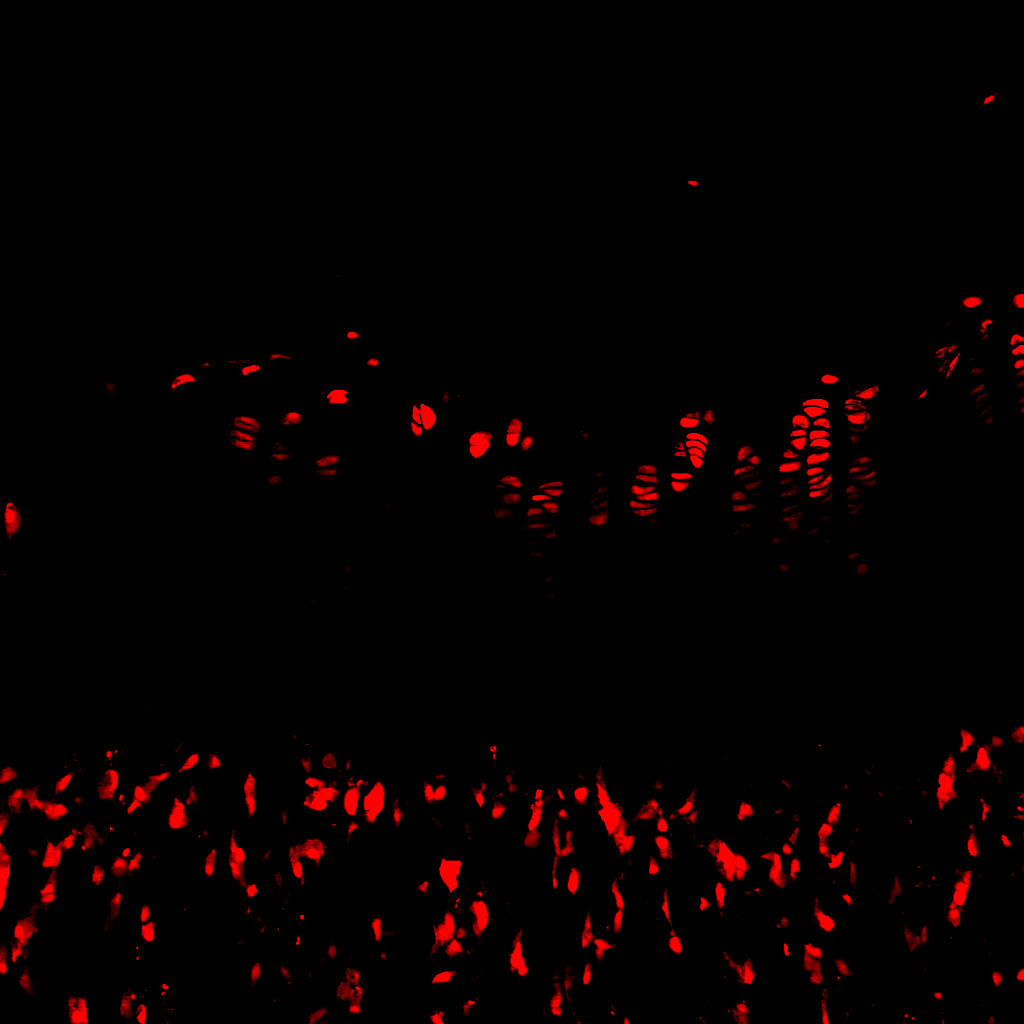

Supplement: Supplementary file 1 — Source Data Fig. 1 [file 44319_2024_93_MOESM1_ESM.zip › Figure1/1A/Day7/TM1M_D7_GP_td_red.tif]

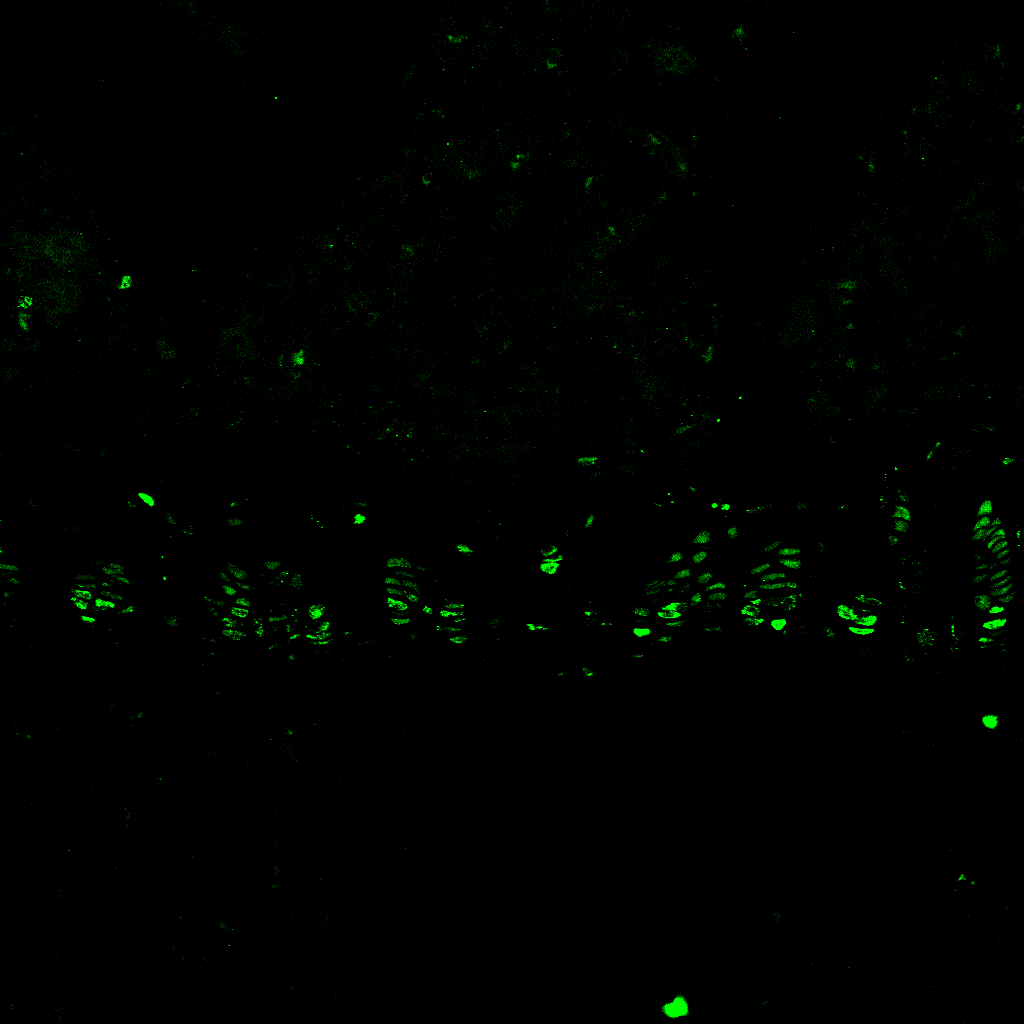

Supplement: Supplementary file 1 — Source Data Fig. 1 [file 44319_2024_93_MOESM1_ESM.zip › Figure1/1C/TM12_1mo/TM12M_1mo_GP_acan_green.tif]

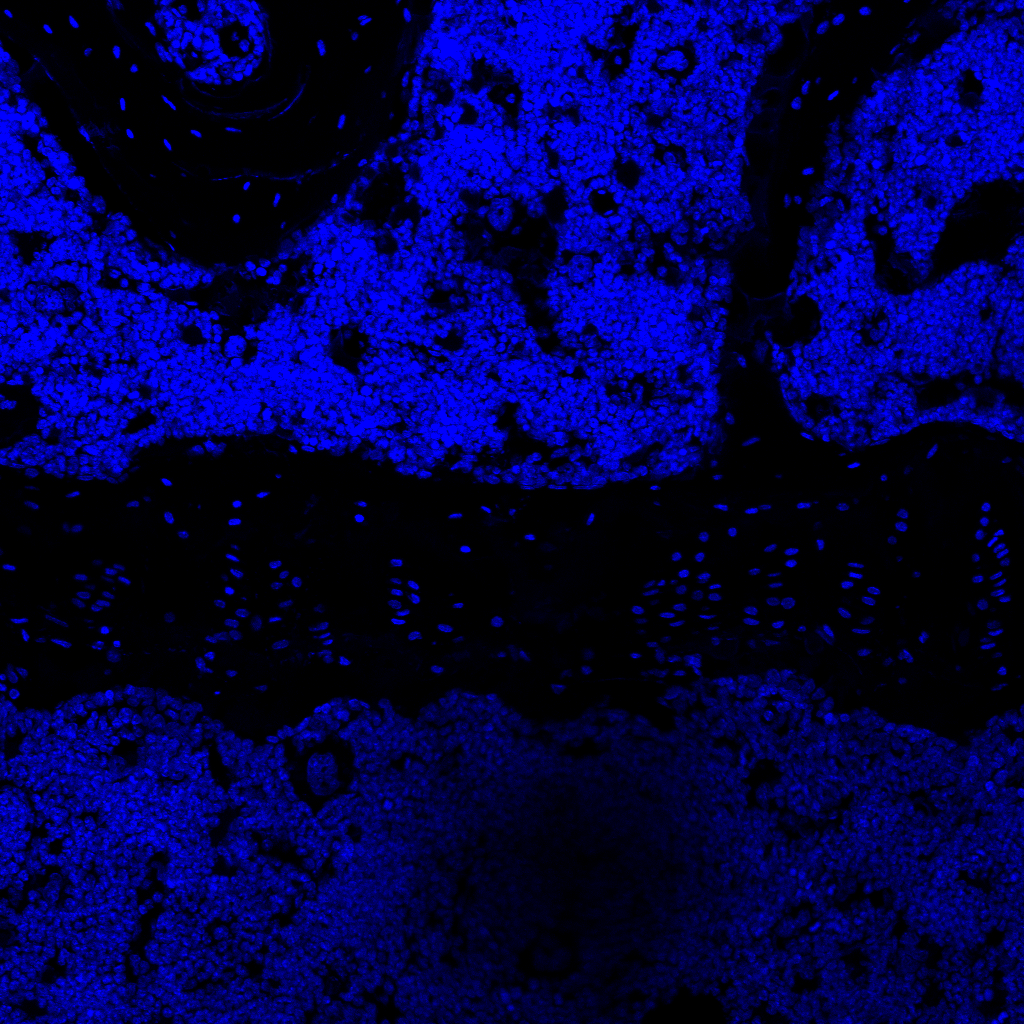

Supplement: Supplementary file 1 — Source Data Fig. 1 [file 44319_2024_93_MOESM1_ESM.zip › Figure1/1C/TM12_1mo/TM12M_1mo_GP_dapi_blue.tif]

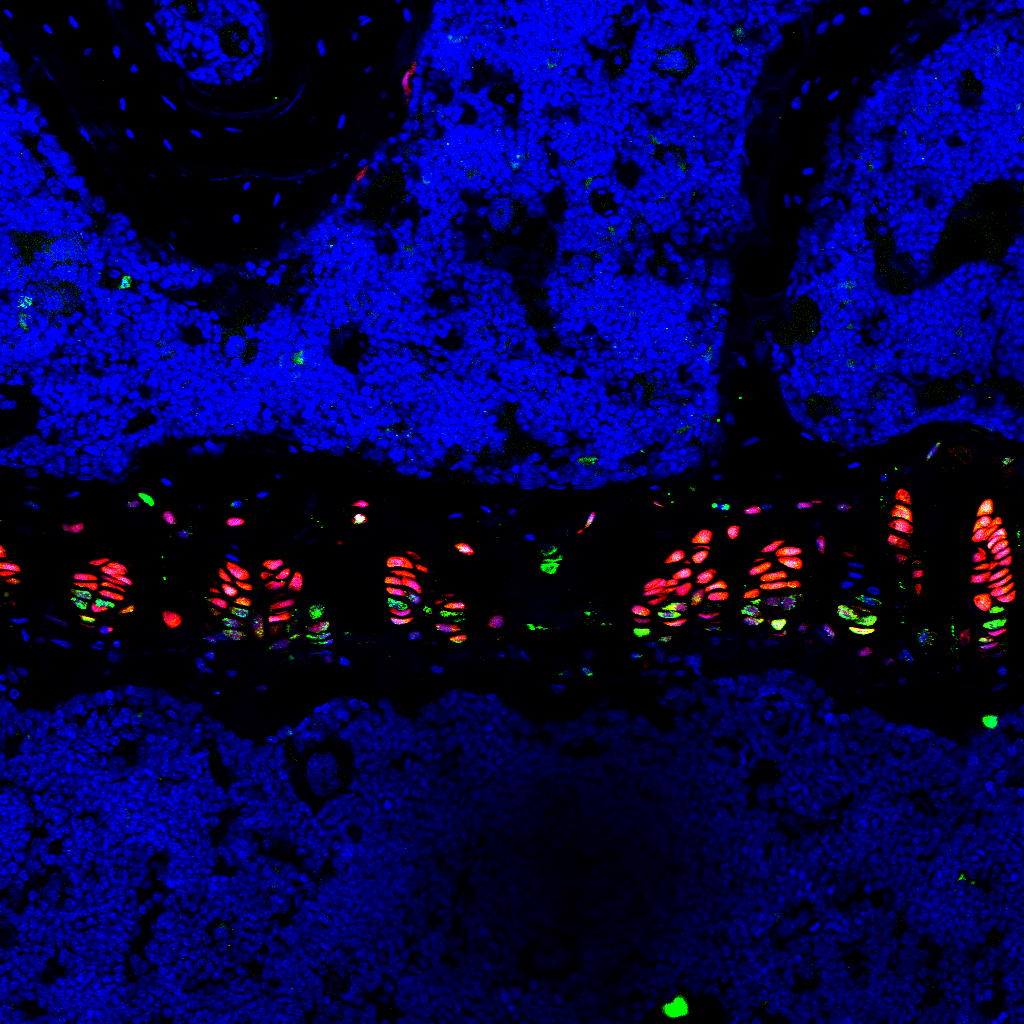

Supplement: Supplementary file 1 — Source Data Fig. 1 [file 44319_2024_93_MOESM1_ESM.zip › Figure1/1C/TM12_1mo/TM12M_1mo_GP_merge.tif]

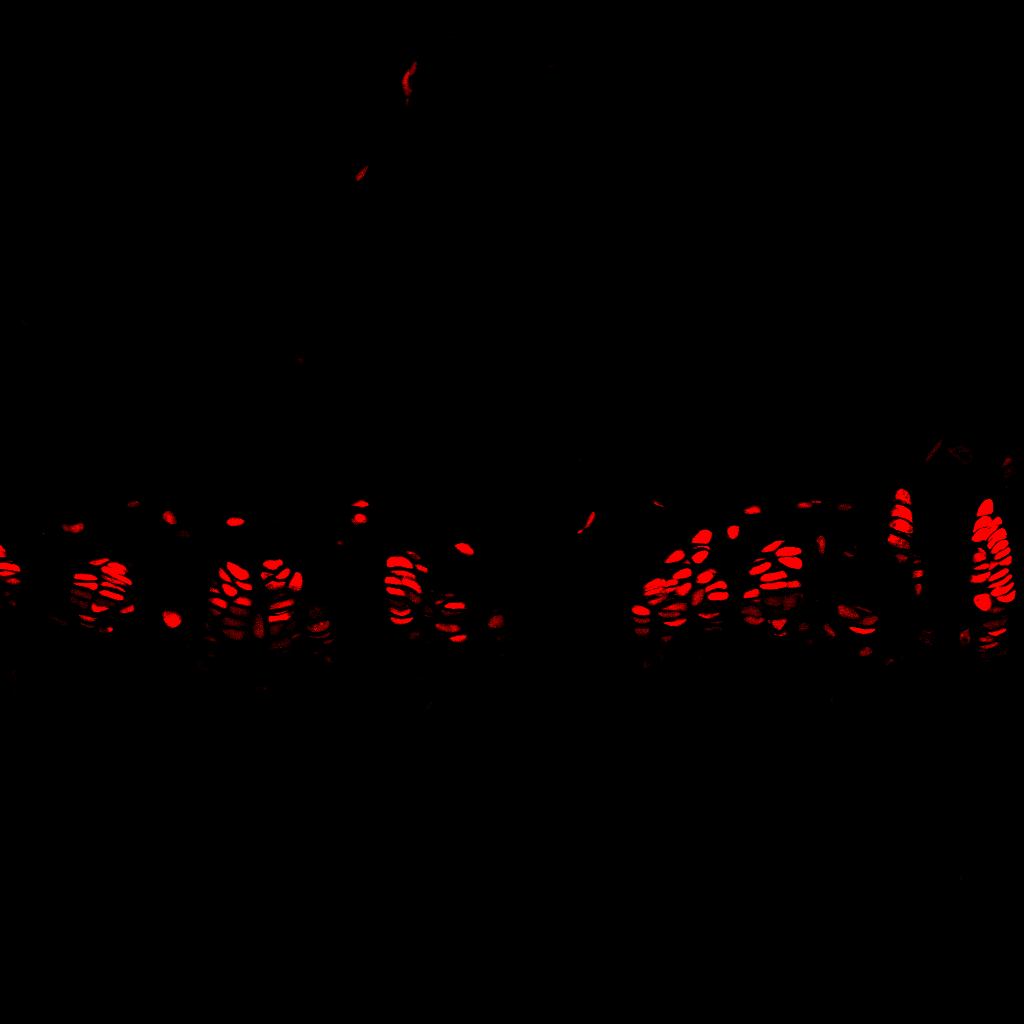

Supplement: Supplementary file 1 — Source Data Fig. 1 [file 44319_2024_93_MOESM1_ESM.zip › Figure1/1C/TM12_1mo/TM12M_1mo_GP_td_red.tif]

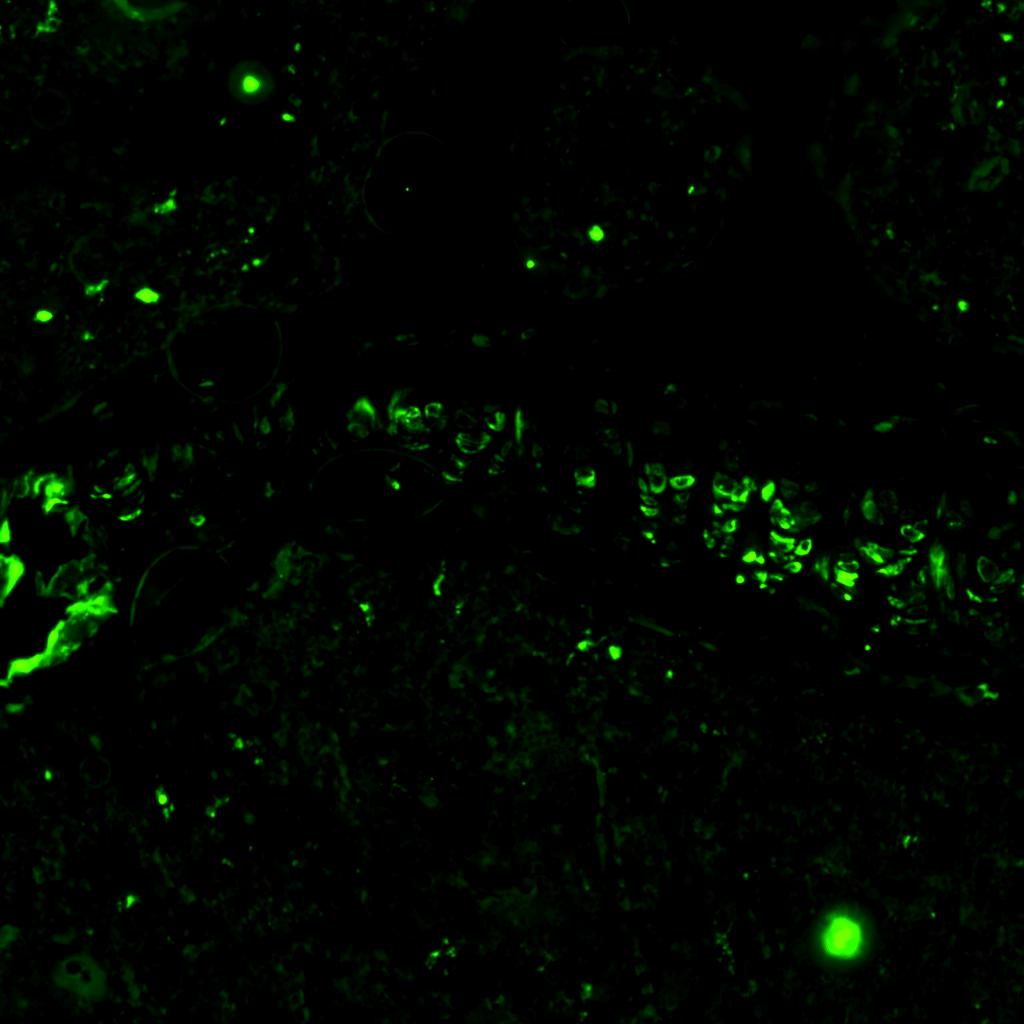

Supplement: Supplementary file 1 — Source Data Fig. 1 [file 44319_2024_93_MOESM1_ESM.zip › Figure1/1C/TM12_D1/TM12M_D1_GP_acan_green.tif]

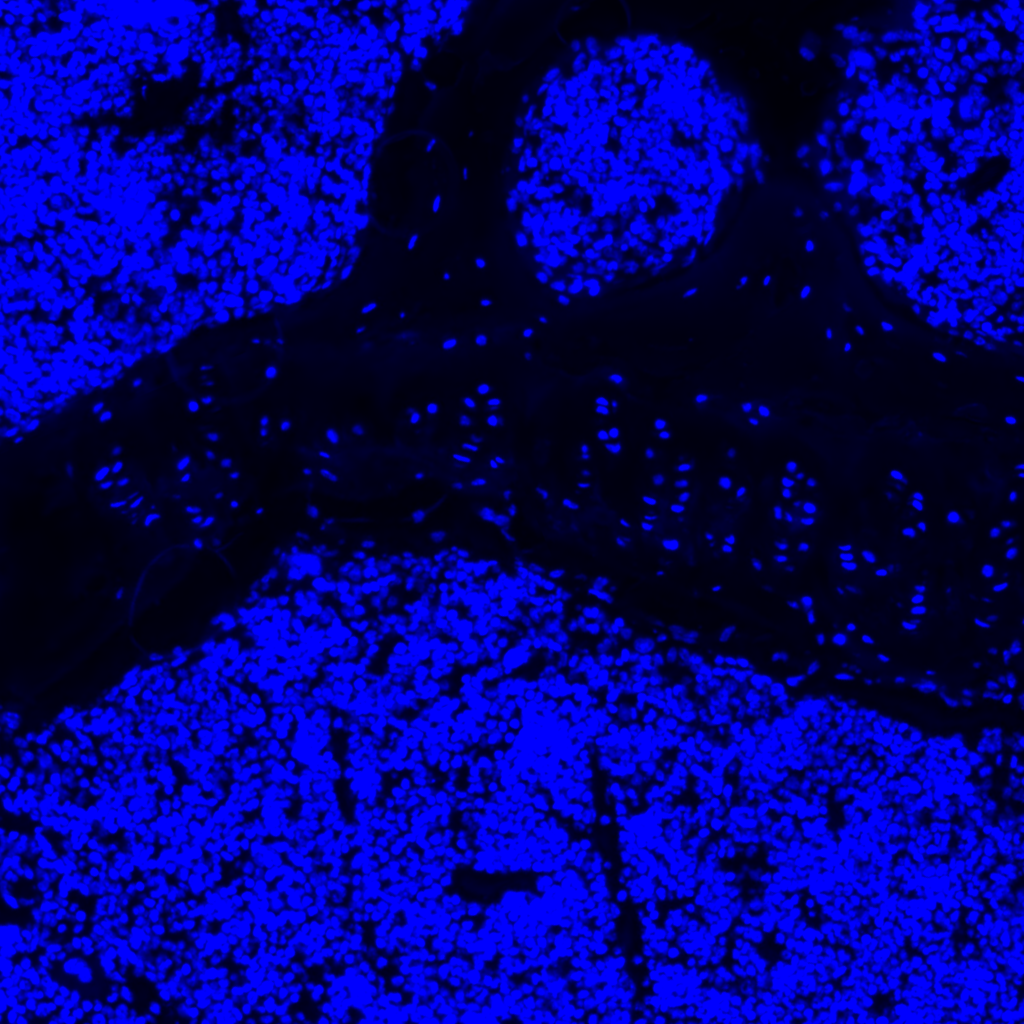

Supplement: Supplementary file 1 — Source Data Fig. 1 [file 44319_2024_93_MOESM1_ESM.zip › Figure1/1C/TM12_D1/TM12M_D1_GP_dapi_blue.tif]

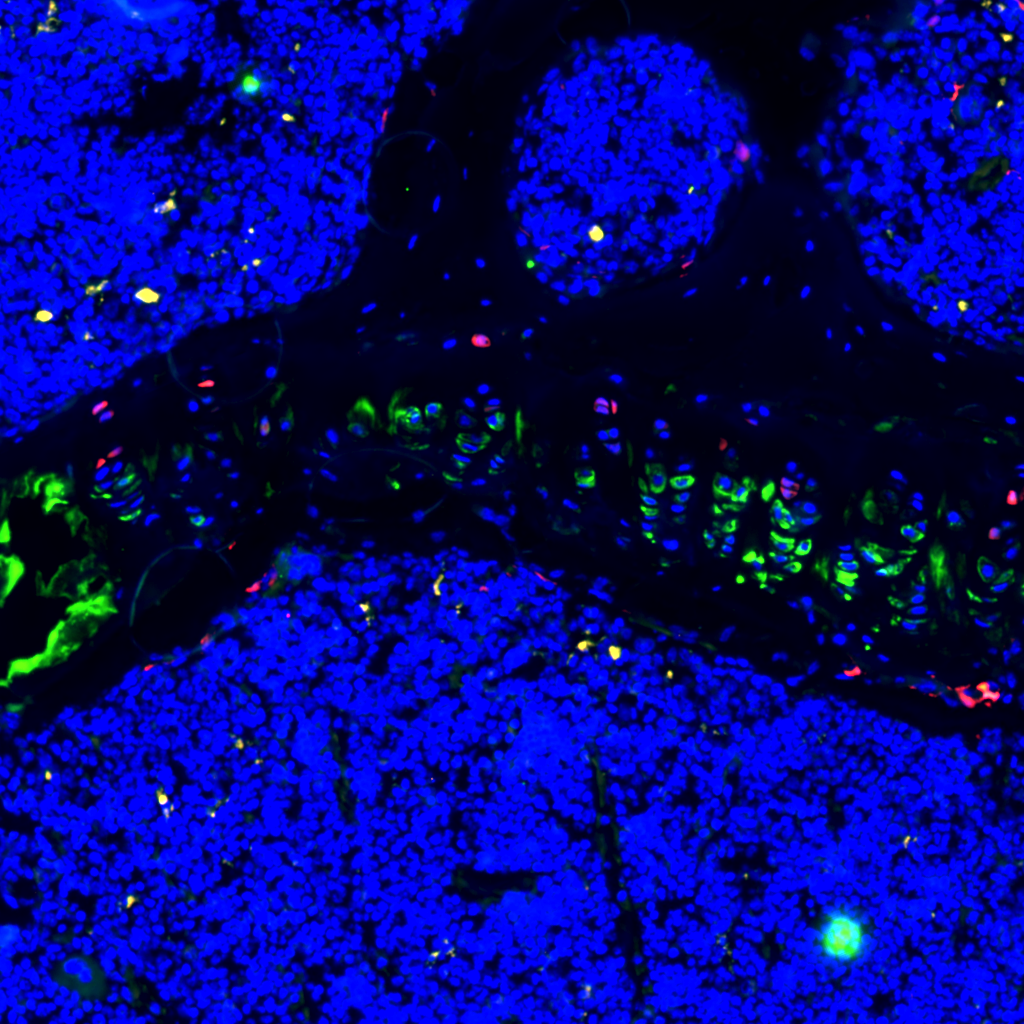

Supplement: Supplementary file 1 — Source Data Fig. 1 [file 44319_2024_93_MOESM1_ESM.zip › Figure1/1C/TM12_D1/TM12M_D1_GP_merge.tif]

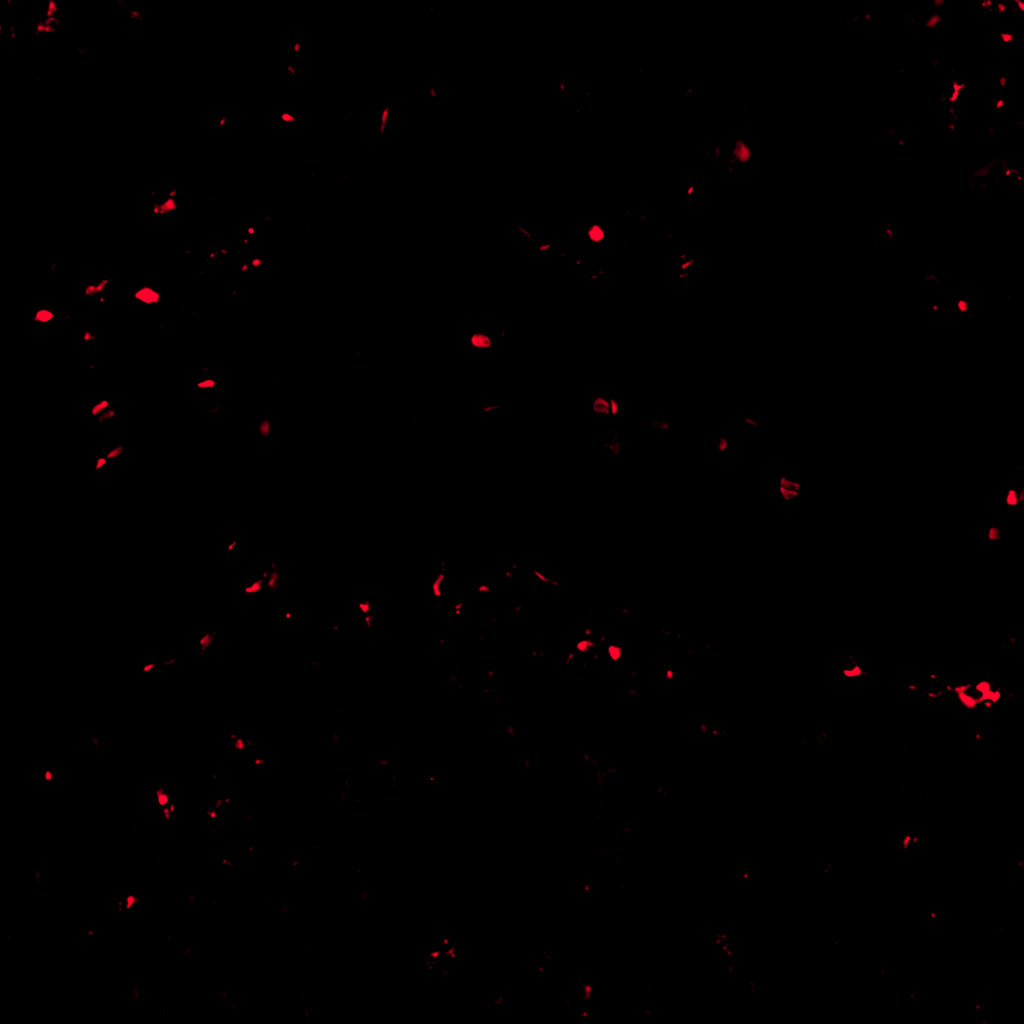

Supplement: Supplementary file 1 — Source Data Fig. 1 [file 44319_2024_93_MOESM1_ESM.zip › Figure1/1C/TM12_D1/TM12M_D1_GP_td_red.tif]

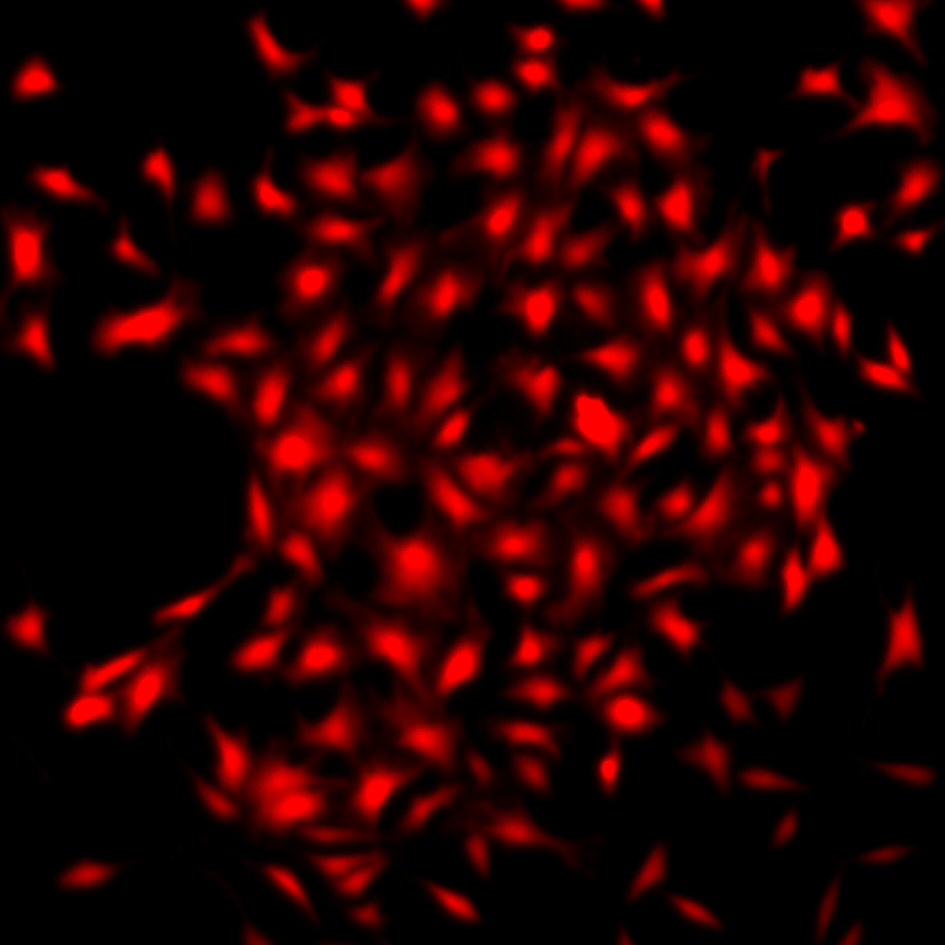

Supplement: Supplementary file 2 — Source Data Fig. 2 [file 44319_2024_93_MOESM2_ESM.zip › Figure2/2B/p0.tif]

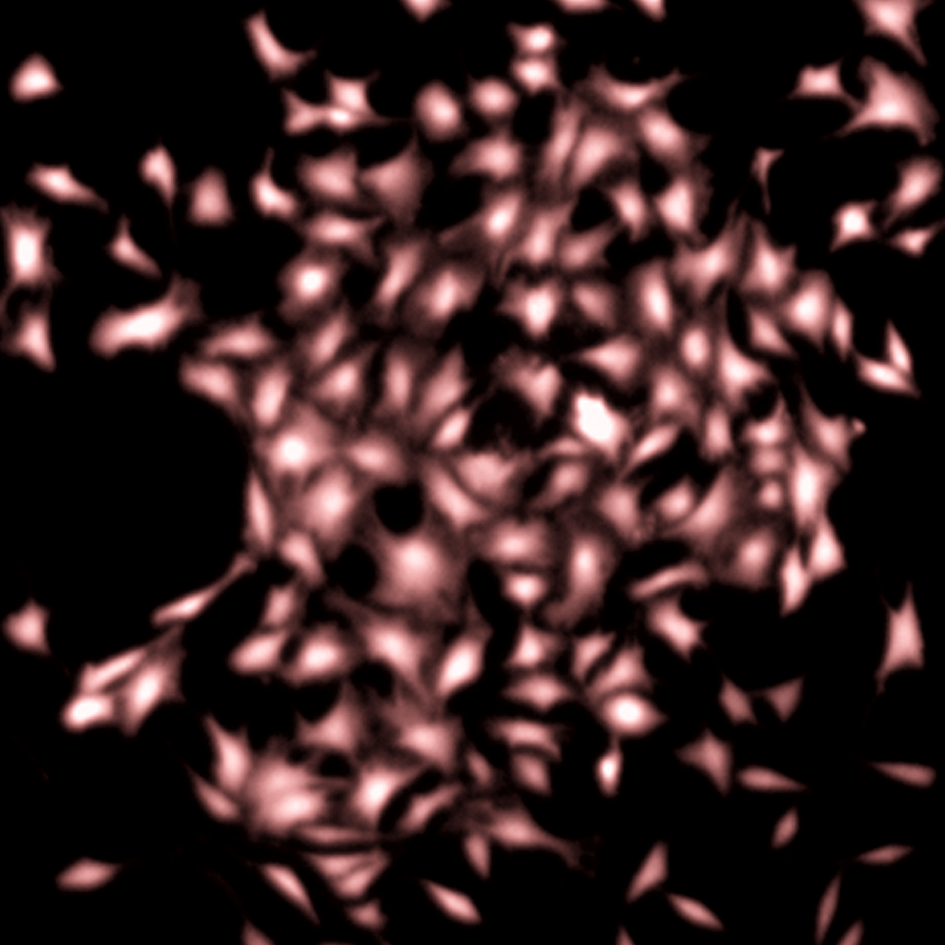

Supplement: Supplementary file 2 — Source Data Fig. 2 [file 44319_2024_93_MOESM2_ESM.zip › Figure2/2B/p0_bf.tif]

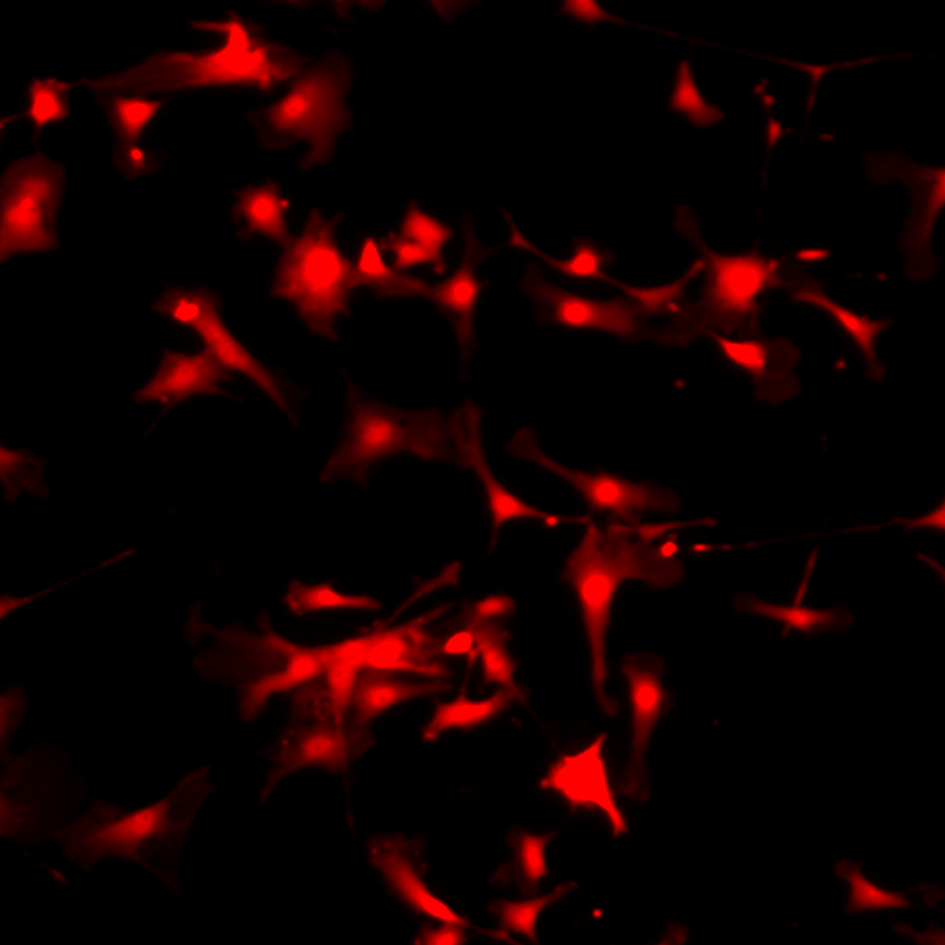

Supplement: Supplementary file 2 — Source Data Fig. 2 [file 44319_2024_93_MOESM2_ESM.zip › Figure2/2B/p1.tif]

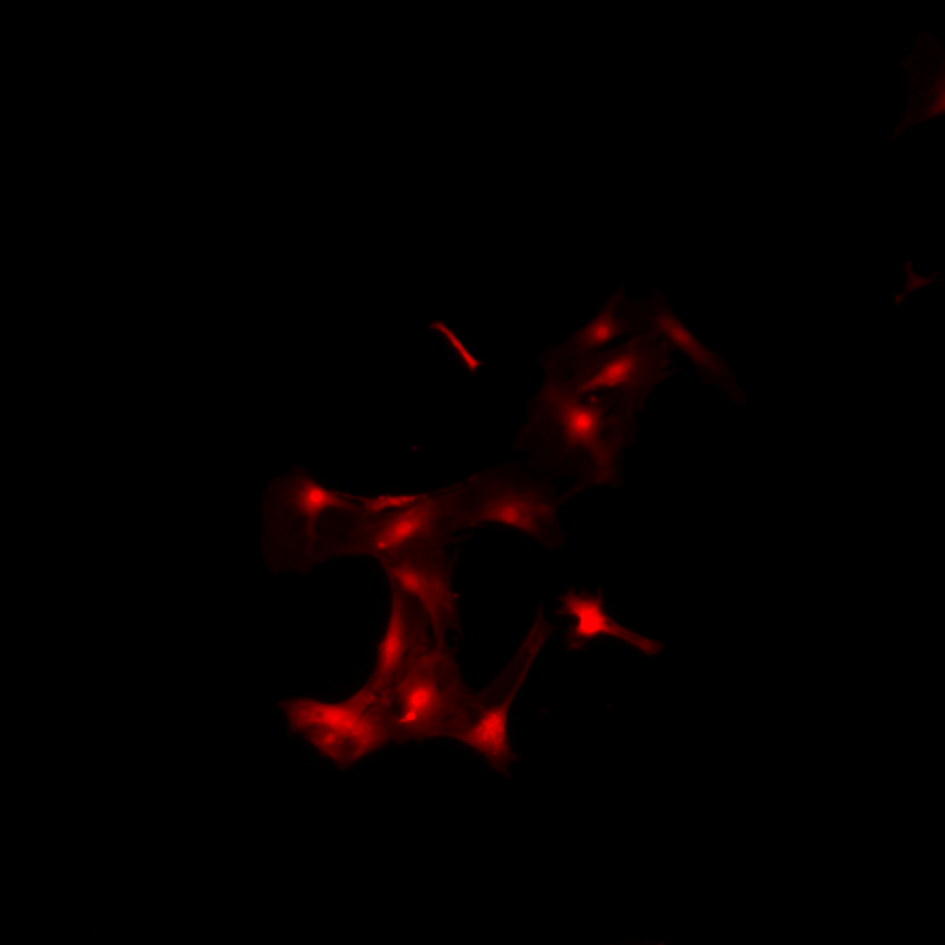

Supplement: Supplementary file 2 — Source Data Fig. 2 [file 44319_2024_93_MOESM2_ESM.zip › Figure2/2B/p5.tif]

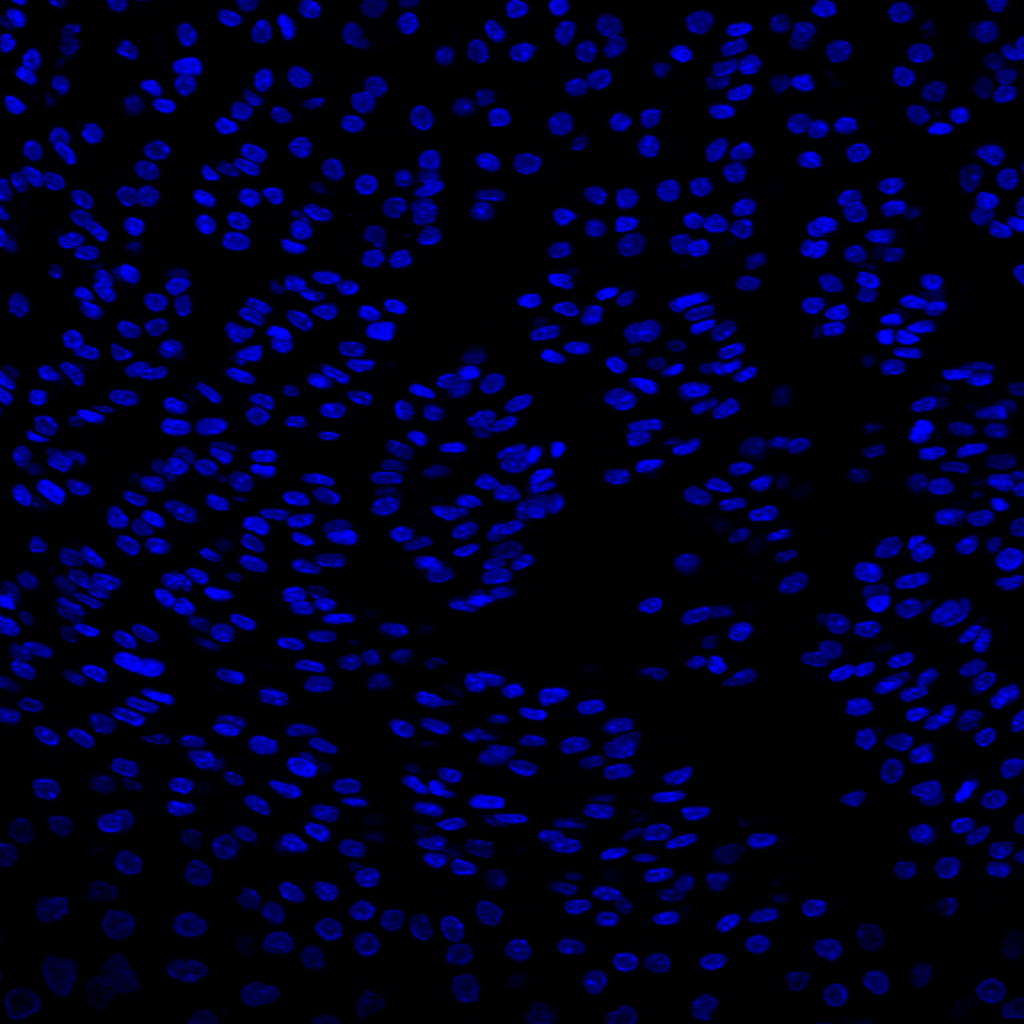

Supplement: Supplementary file 2 — Source Data Fig. 2 [file 44319_2024_93_MOESM2_ESM.zip › Figure2/2D/CC_DAPI_blue.tif]

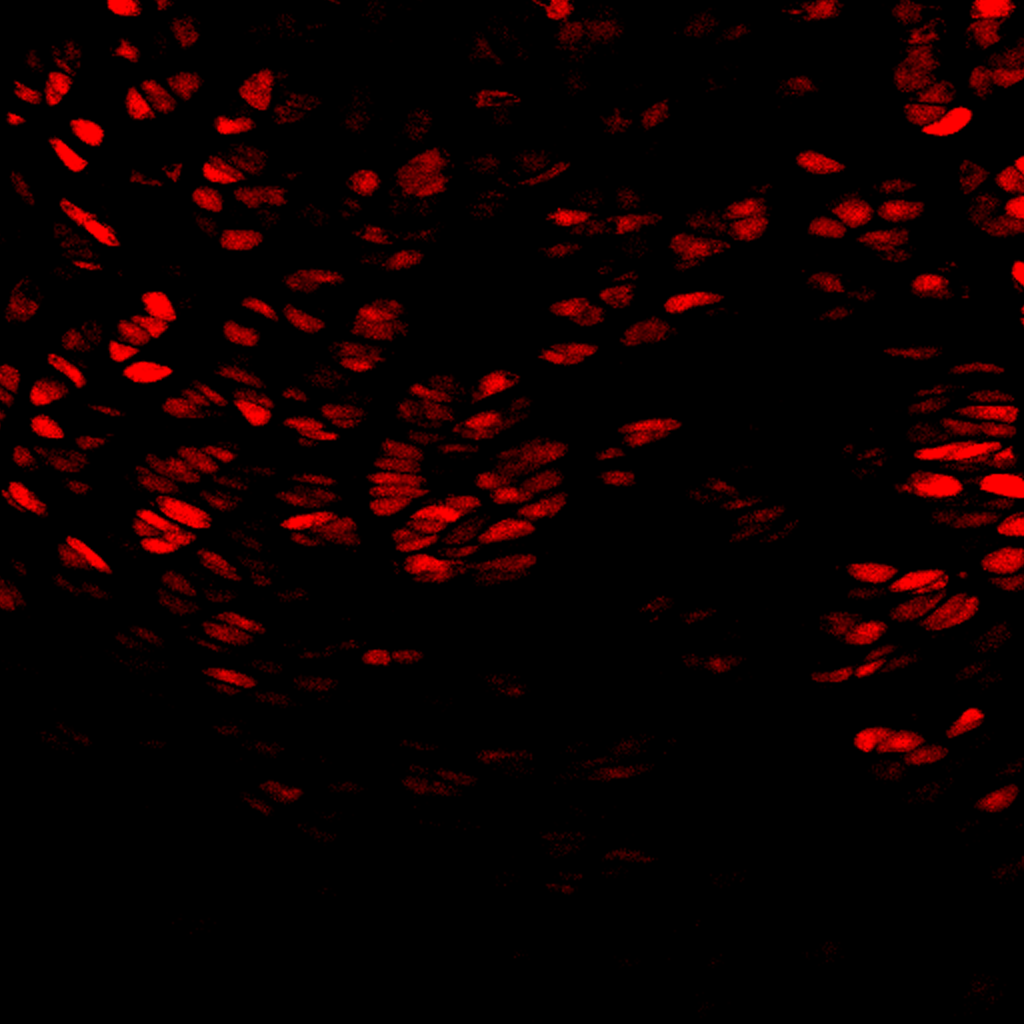

Supplement: Supplementary file 2 — Source Data Fig. 2 [file 44319_2024_93_MOESM2_ESM.zip › Figure2/2D/CC_Gli_red.tif]

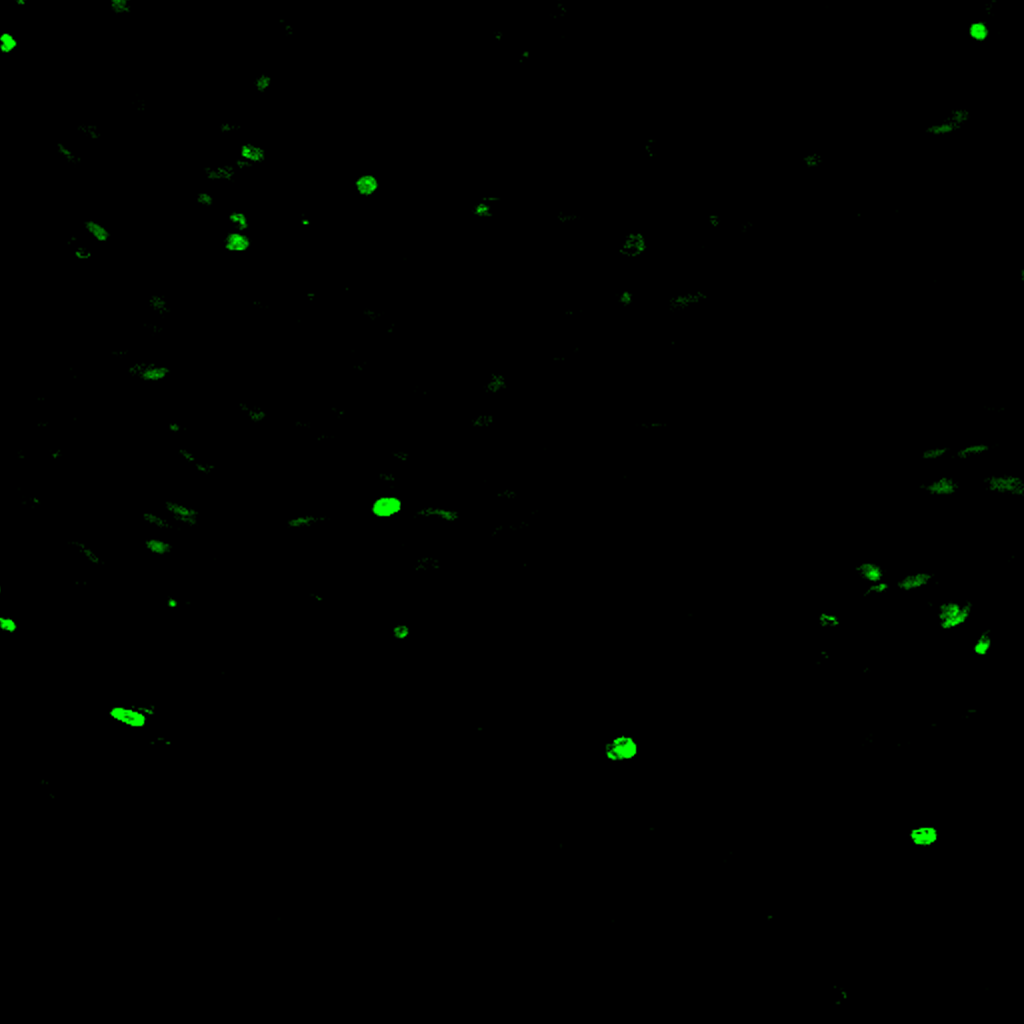

Supplement: Supplementary file 2 — Source Data Fig. 2 [file 44319_2024_93_MOESM2_ESM.zip › Figure2/2D/CC_Ki67_green.tif]

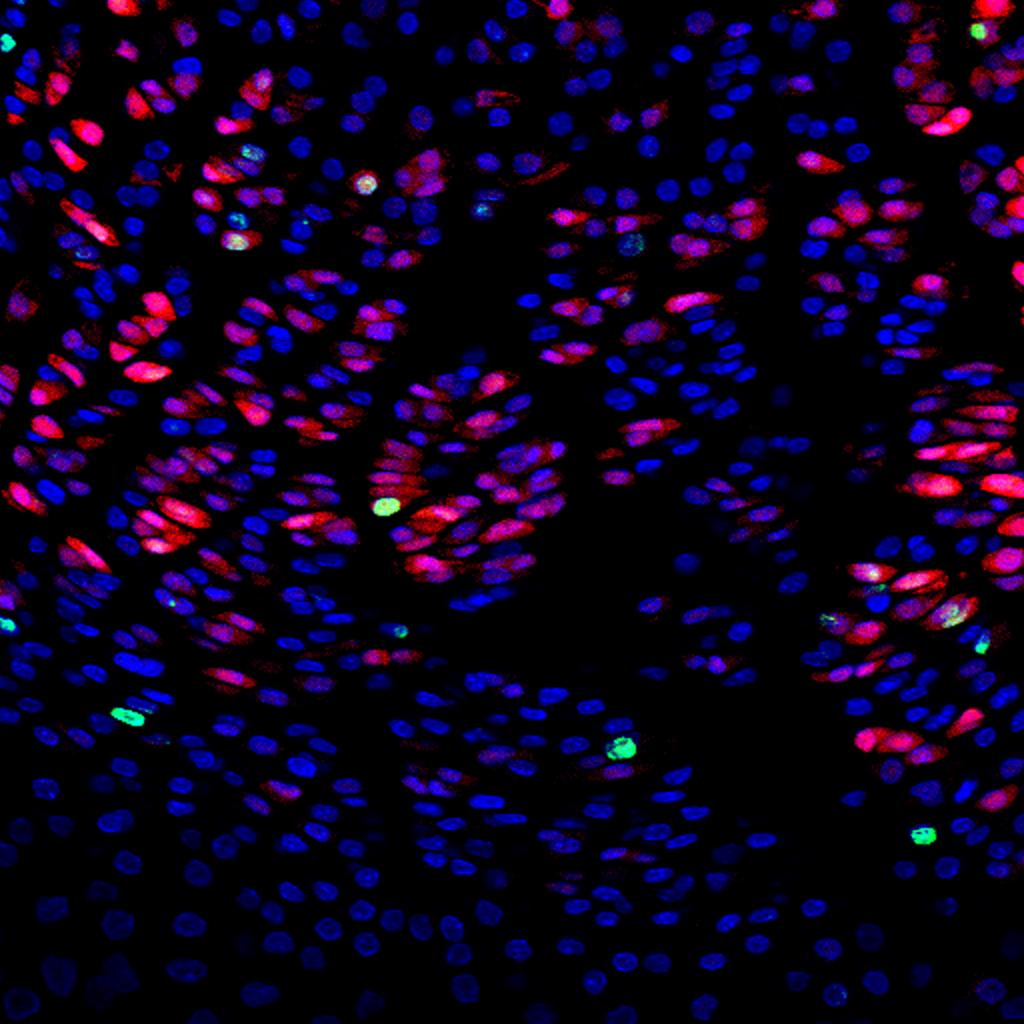

Supplement: Supplementary file 2 — Source Data Fig. 2 [file 44319_2024_93_MOESM2_ESM.zip › Figure2/2D/CC_merge.tif]

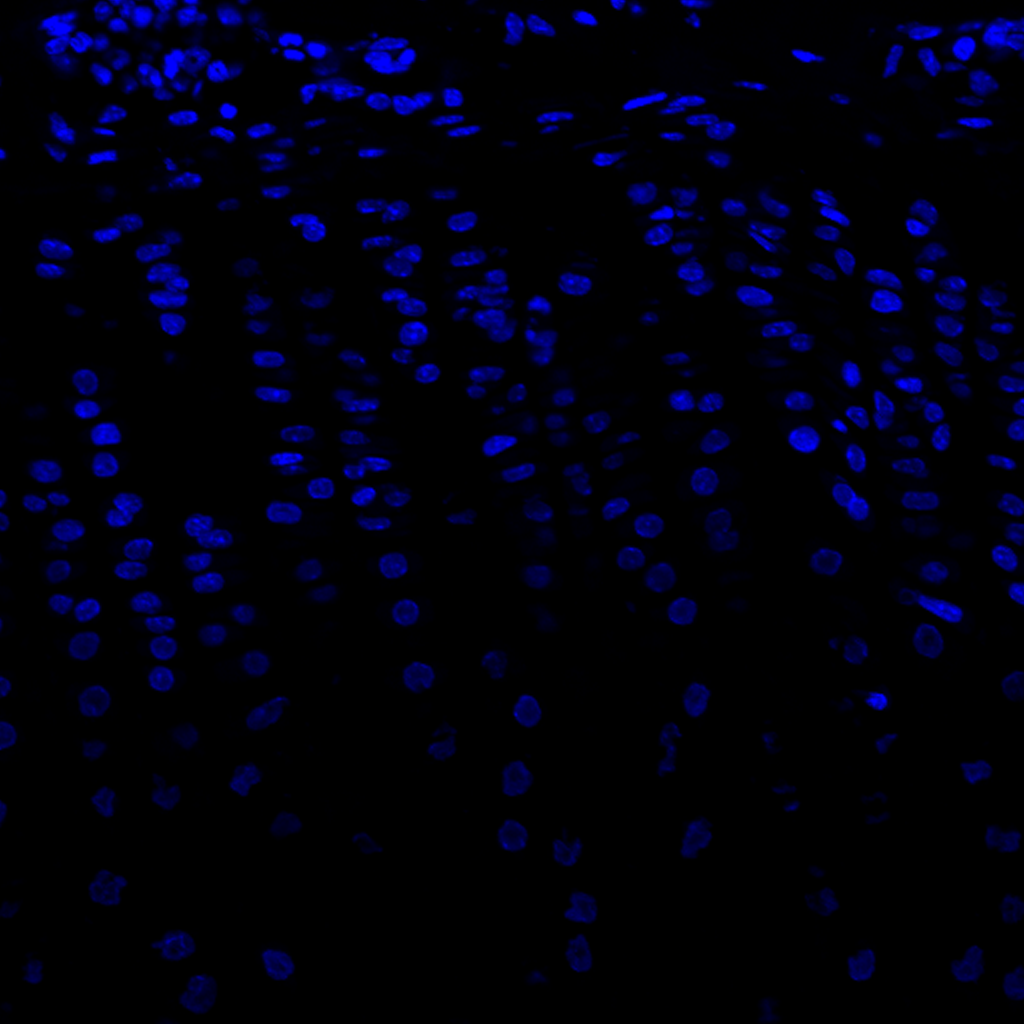

Supplement: Supplementary file 2 — Source Data Fig. 2 [file 44319_2024_93_MOESM2_ESM.zip › Figure2/2D/GP_Dapi_blue.tif]

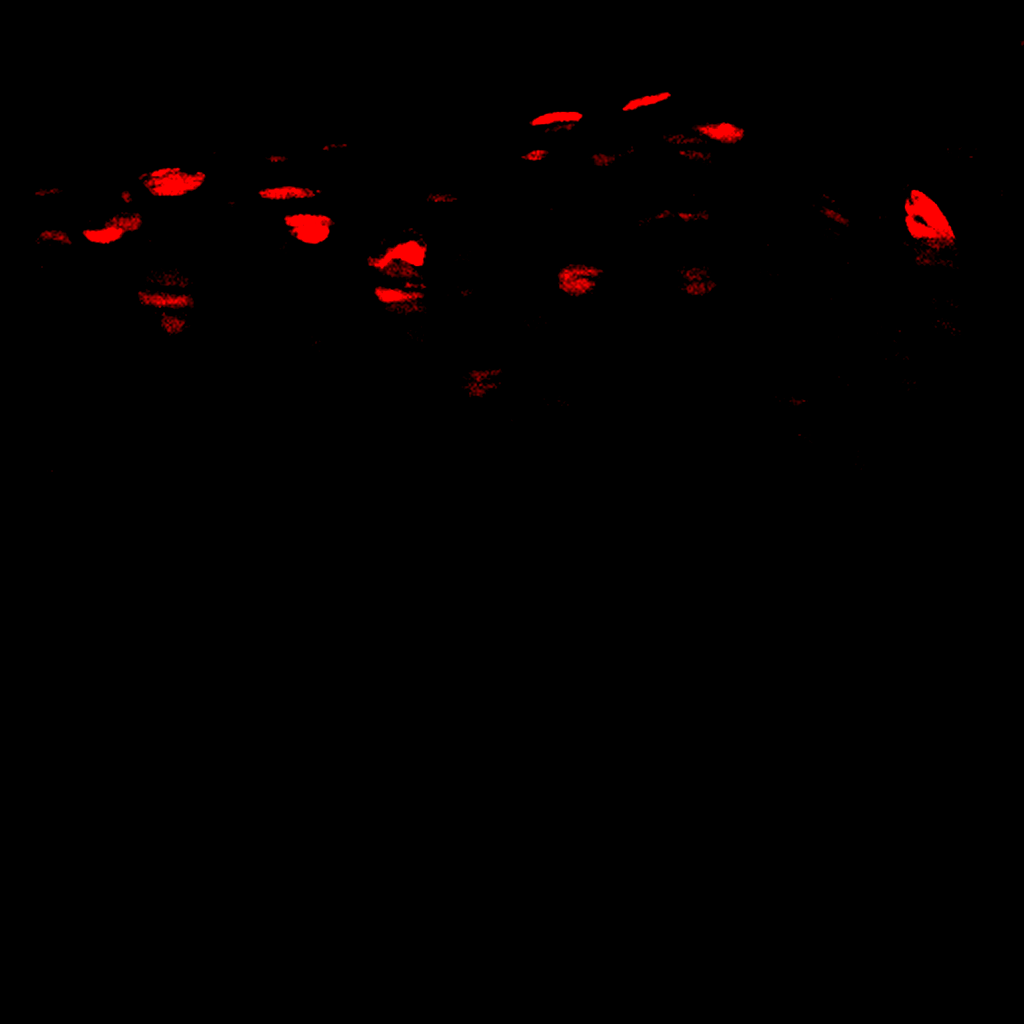

Supplement: Supplementary file 2 — Source Data Fig. 2 [file 44319_2024_93_MOESM2_ESM.zip › Figure2/2D/GP_Gli_red.tif]

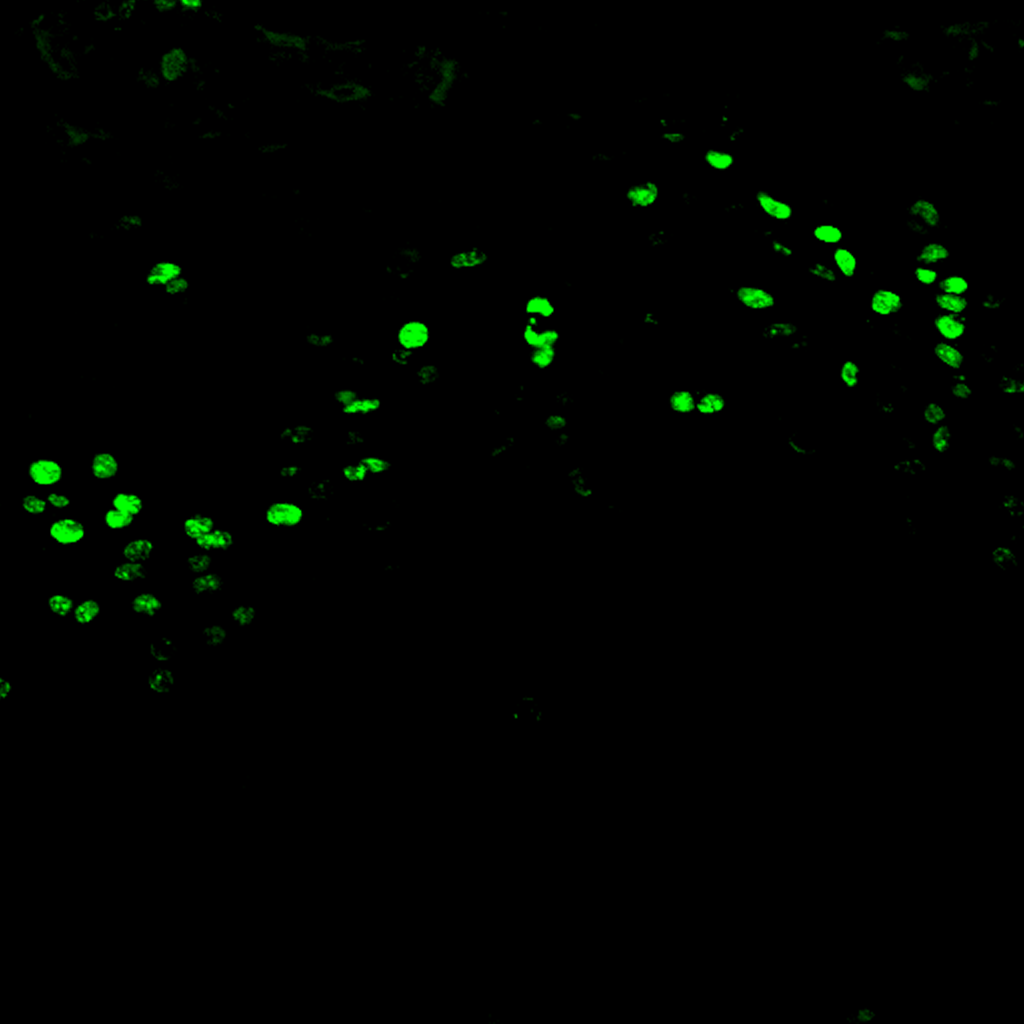

Supplement: Supplementary file 2 — Source Data Fig. 2 [file 44319_2024_93_MOESM2_ESM.zip › Figure2/2D/GP_Ki67_green.tif]

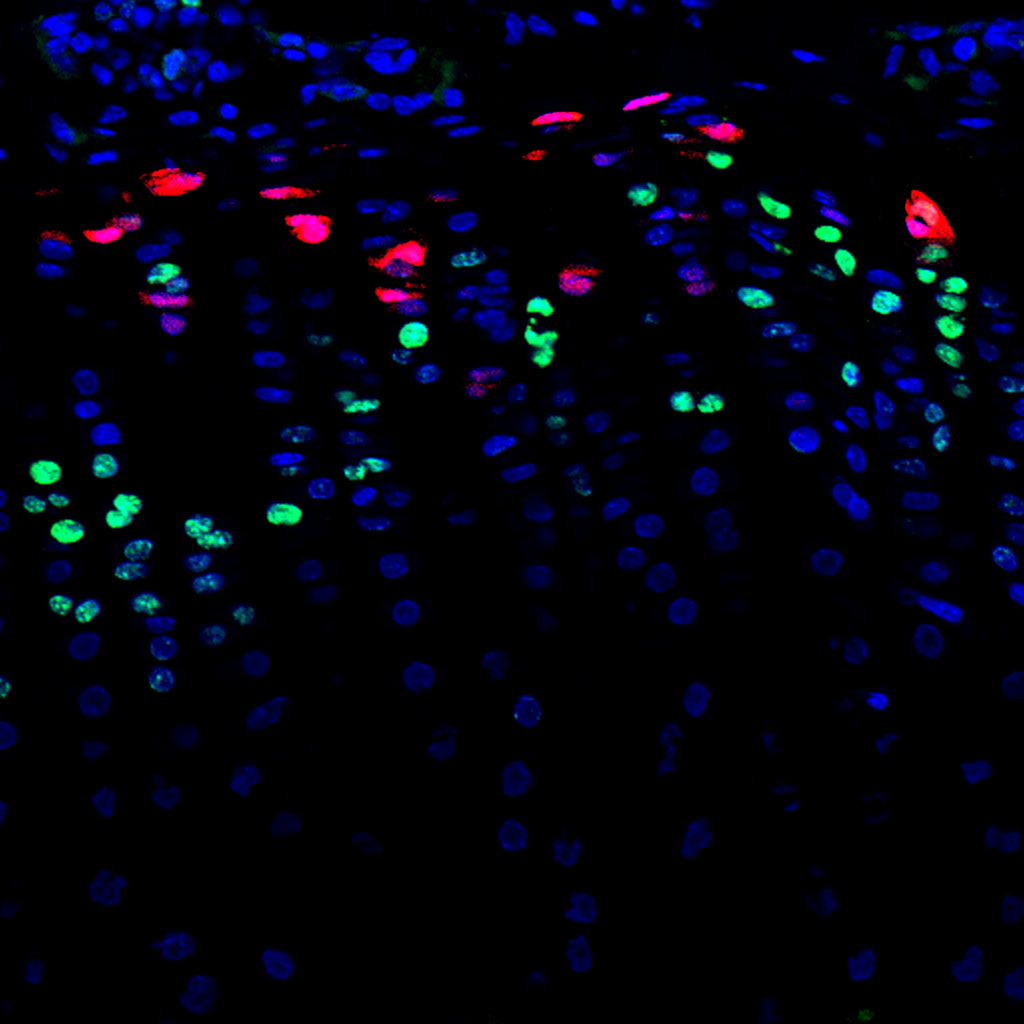

Supplement: Supplementary file 2 — Source Data Fig. 2 [file 44319_2024_93_MOESM2_ESM.zip › Figure2/2D/GP_merge.tif]

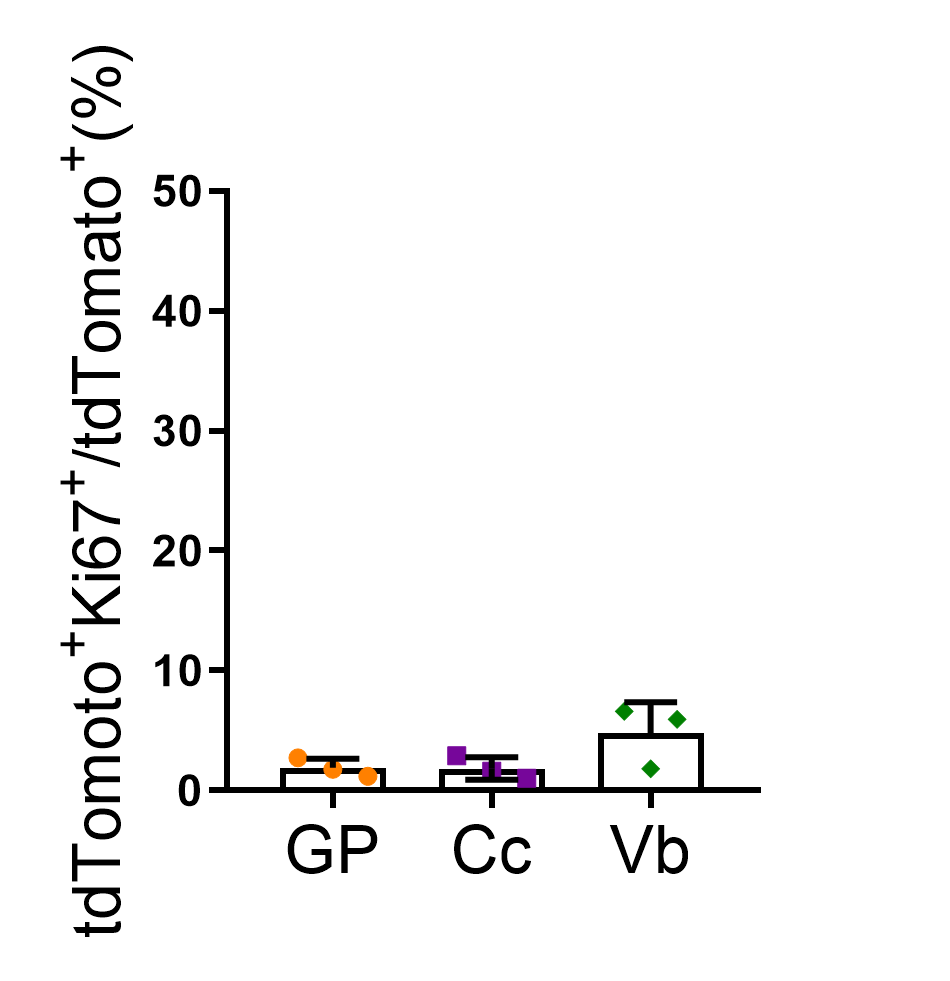

Supplement: Supplementary file 2 — Source Data Fig. 2 [file 44319_2024_93_MOESM2_ESM.zip › Figure2/2D/quant.tif]

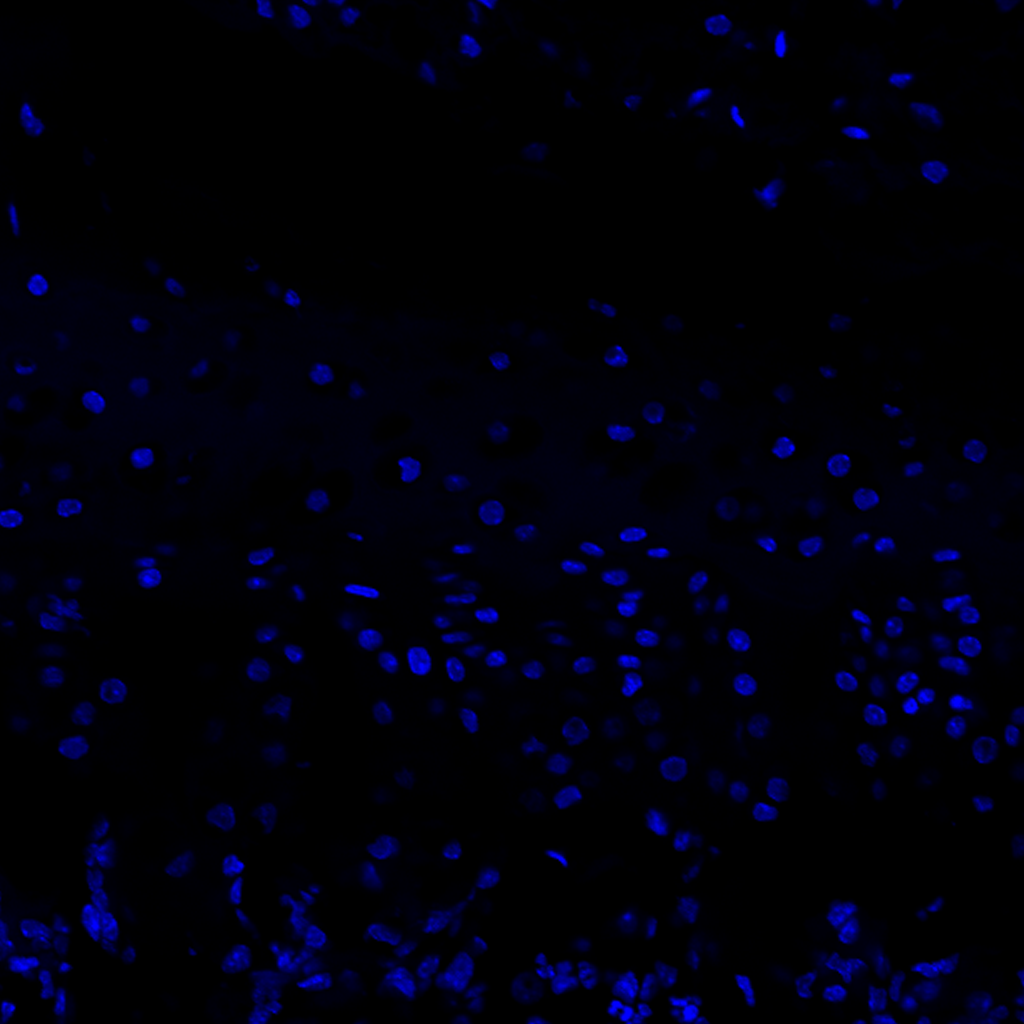

Supplement: Supplementary file 2 — Source Data Fig. 2 [file 44319_2024_93_MOESM2_ESM.zip › Figure2/2D/VB_DAPI_blue.tif]

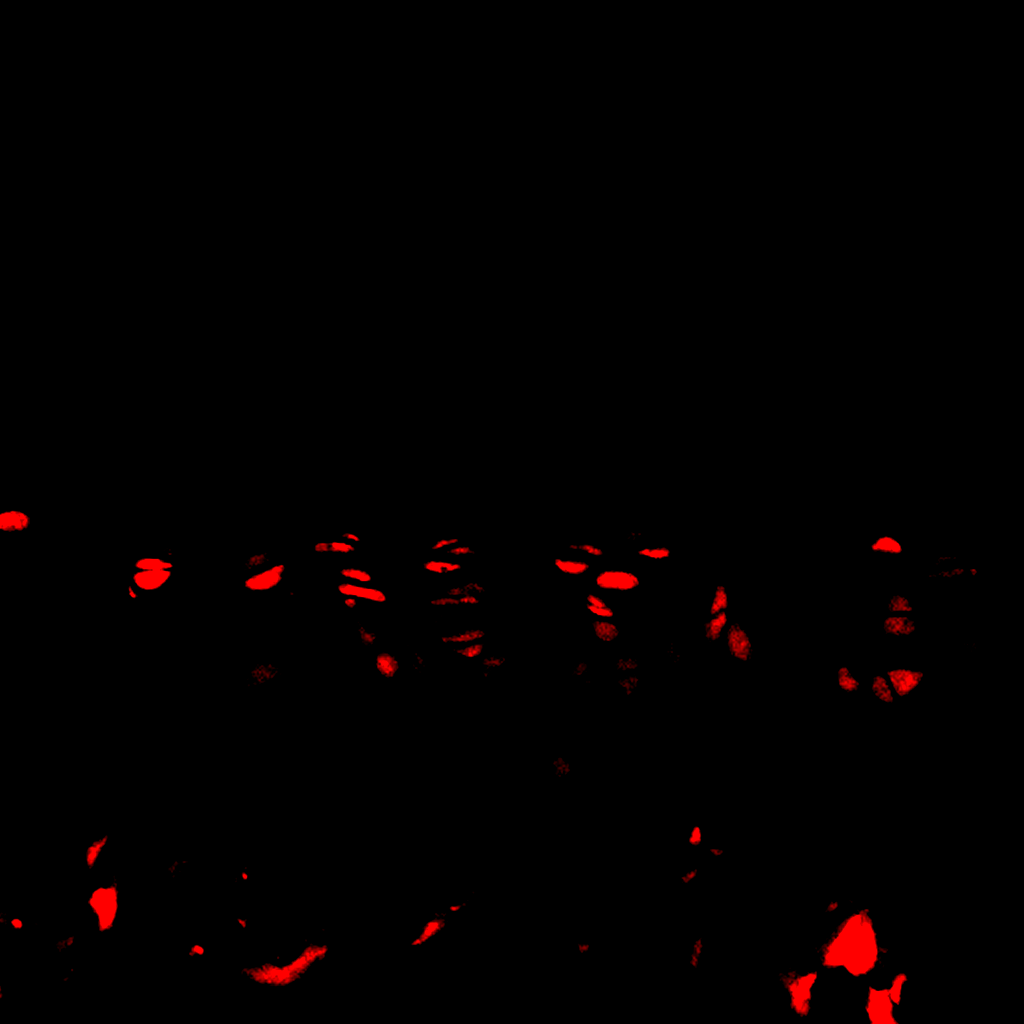

Supplement: Supplementary file 2 — Source Data Fig. 2 [file 44319_2024_93_MOESM2_ESM.zip › Figure2/2D/VB_Gli_red.tif]

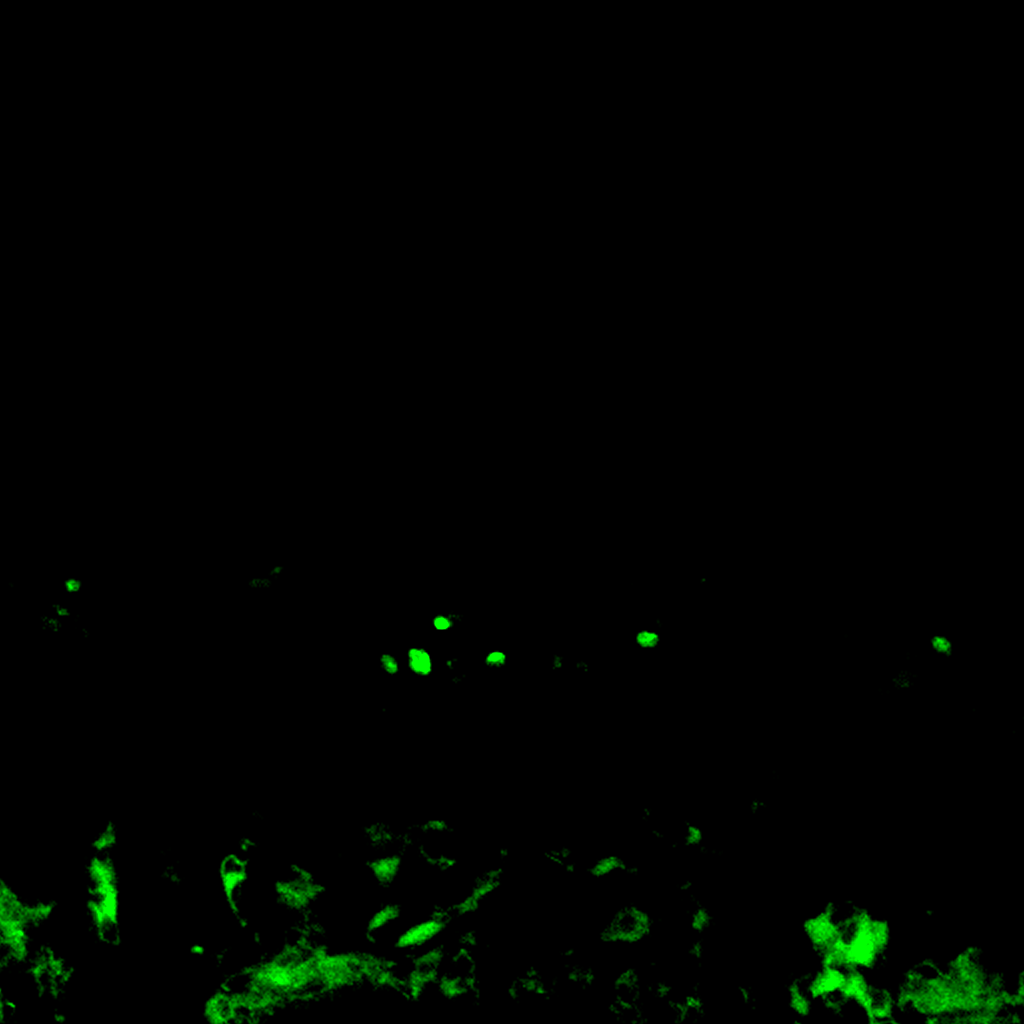

Supplement: Supplementary file 2 — Source Data Fig. 2 [file 44319_2024_93_MOESM2_ESM.zip › Figure2/2D/VB_Ki67_green.tif]

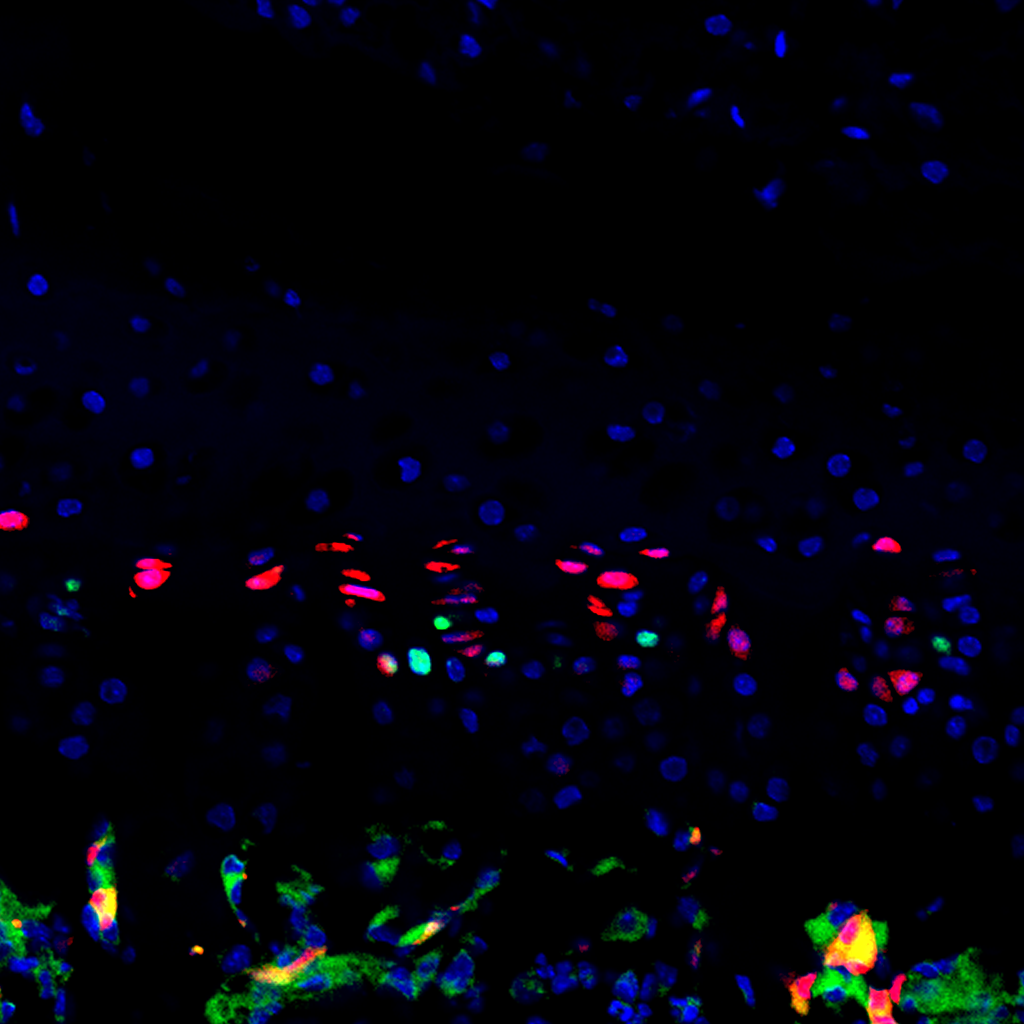

Supplement: Supplementary file 2 — Source Data Fig. 2 [file 44319_2024_93_MOESM2_ESM.zip › Figure2/2D/VB_merge.tif]

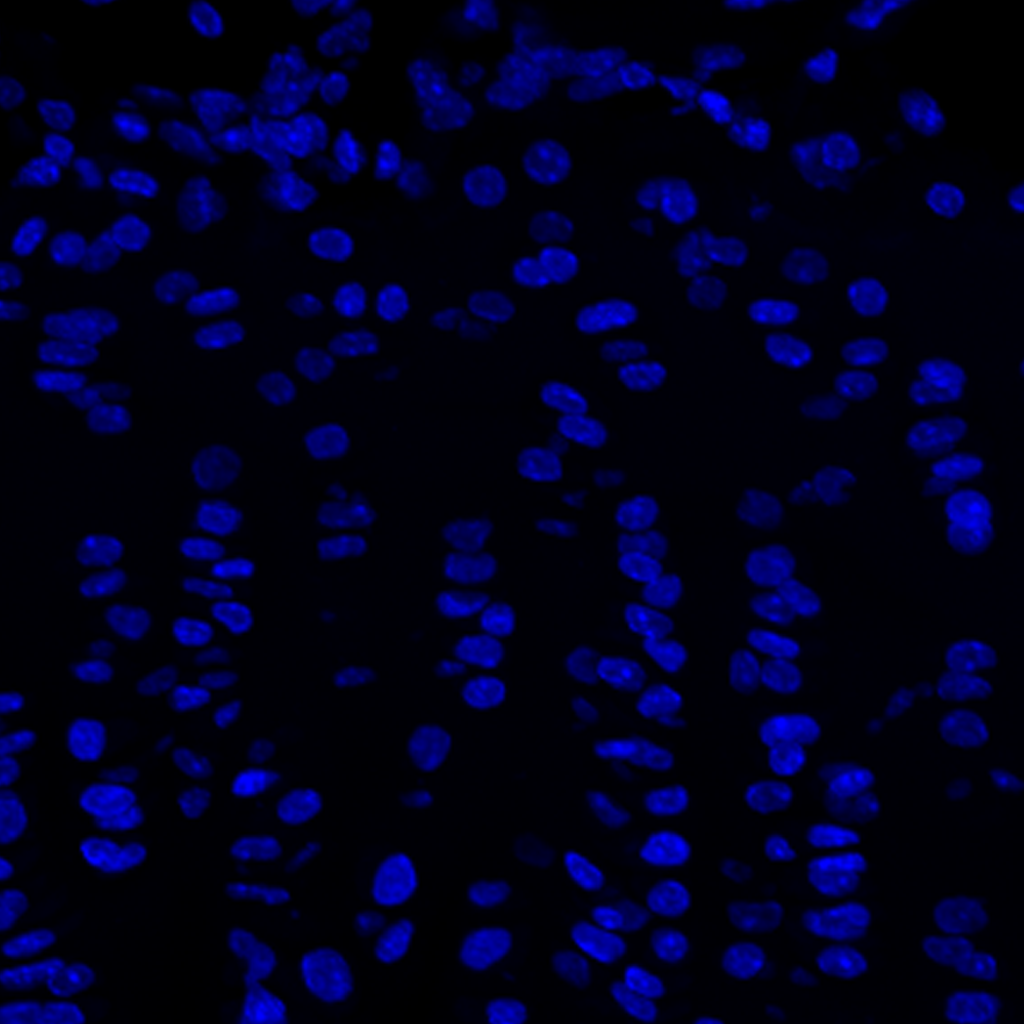

Supplement: Supplementary file 2 — Source Data Fig. 2 [file 44319_2024_93_MOESM2_ESM.zip › Figure2/2F/TM1M_D1_FOXA2_td_DAPI_blue.tif]

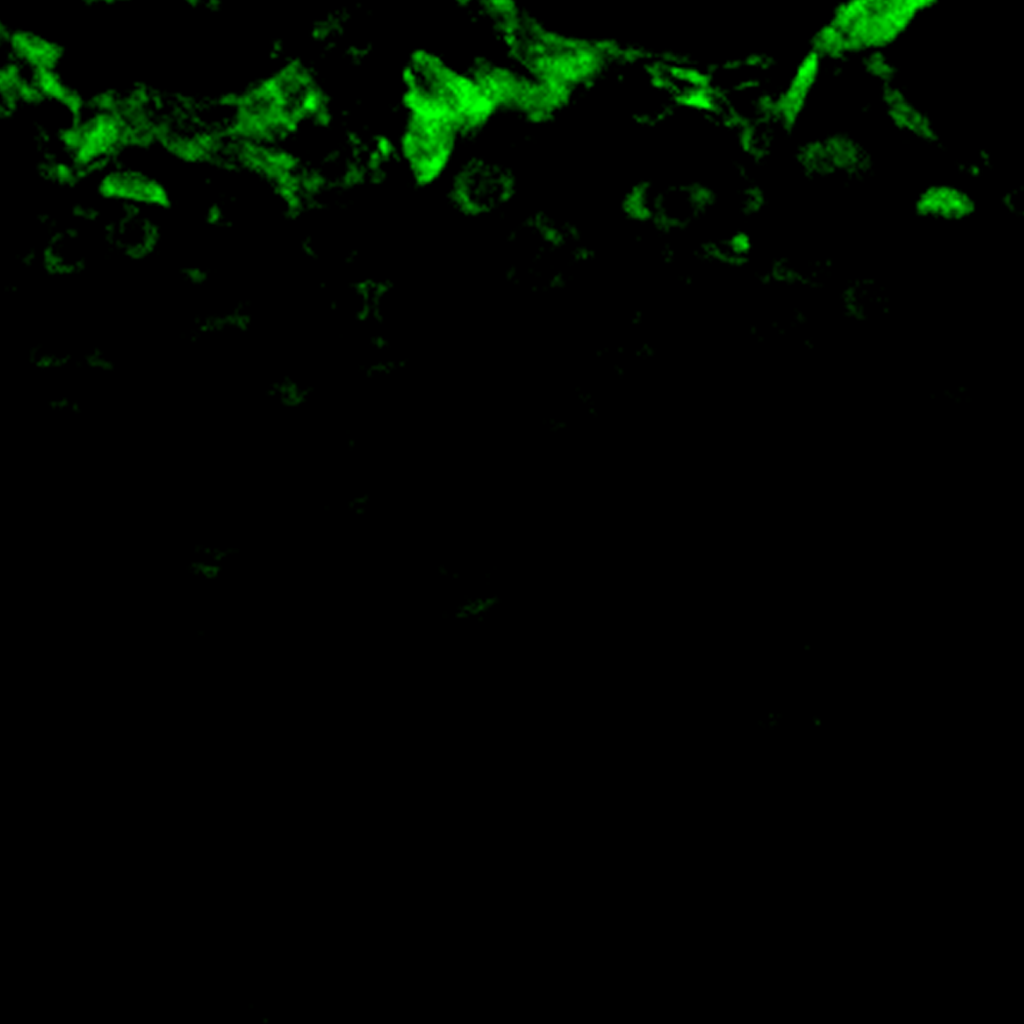

Supplement: Supplementary file 2 — Source Data Fig. 2 [file 44319_2024_93_MOESM2_ESM.zip › Figure2/2F/TM1M_D1_FOXA2_td_FOXA2_green.tif]

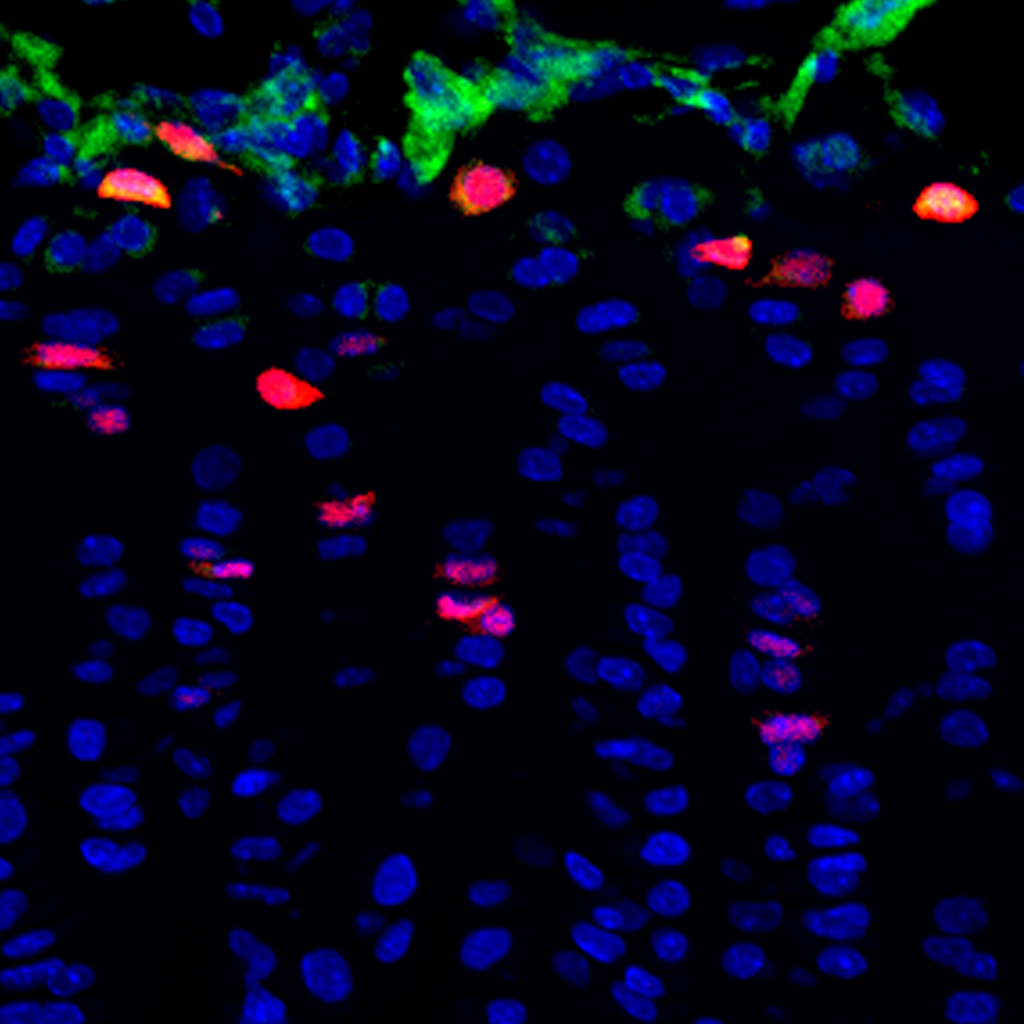

Supplement: Supplementary file 2 — Source Data Fig. 2 [file 44319_2024_93_MOESM2_ESM.zip › Figure2/2F/TM1M_D1_FOXA2_td_merge.tif]

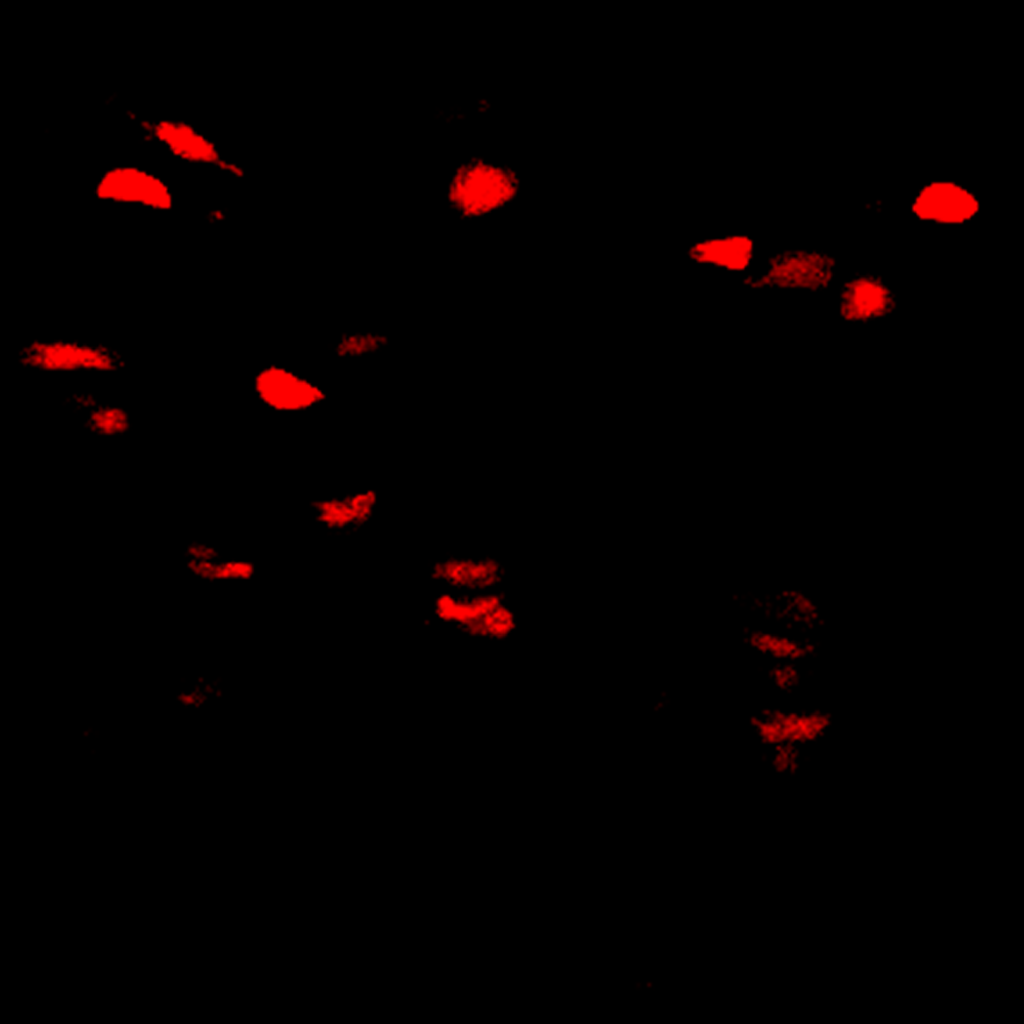

Supplement: Supplementary file 2 — Source Data Fig. 2 [file 44319_2024_93_MOESM2_ESM.zip › Figure2/2F/TM1M_D1_FOXA2_td_td_red.tif]

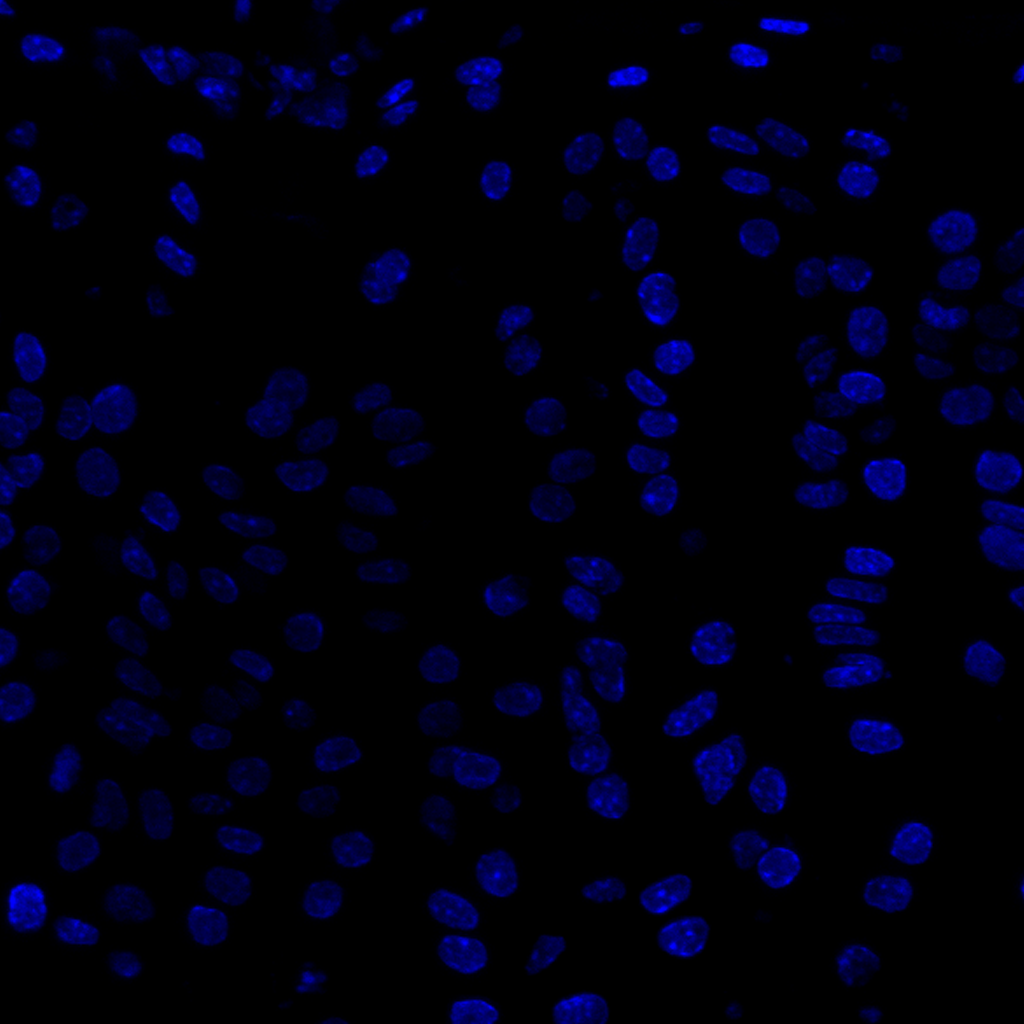

Supplement: Supplementary file 2 — Source Data Fig. 2 [file 44319_2024_93_MOESM2_ESM.zip › Figure2/2F/TM1M_D1_pthlp_td_dapi_blue.tif]

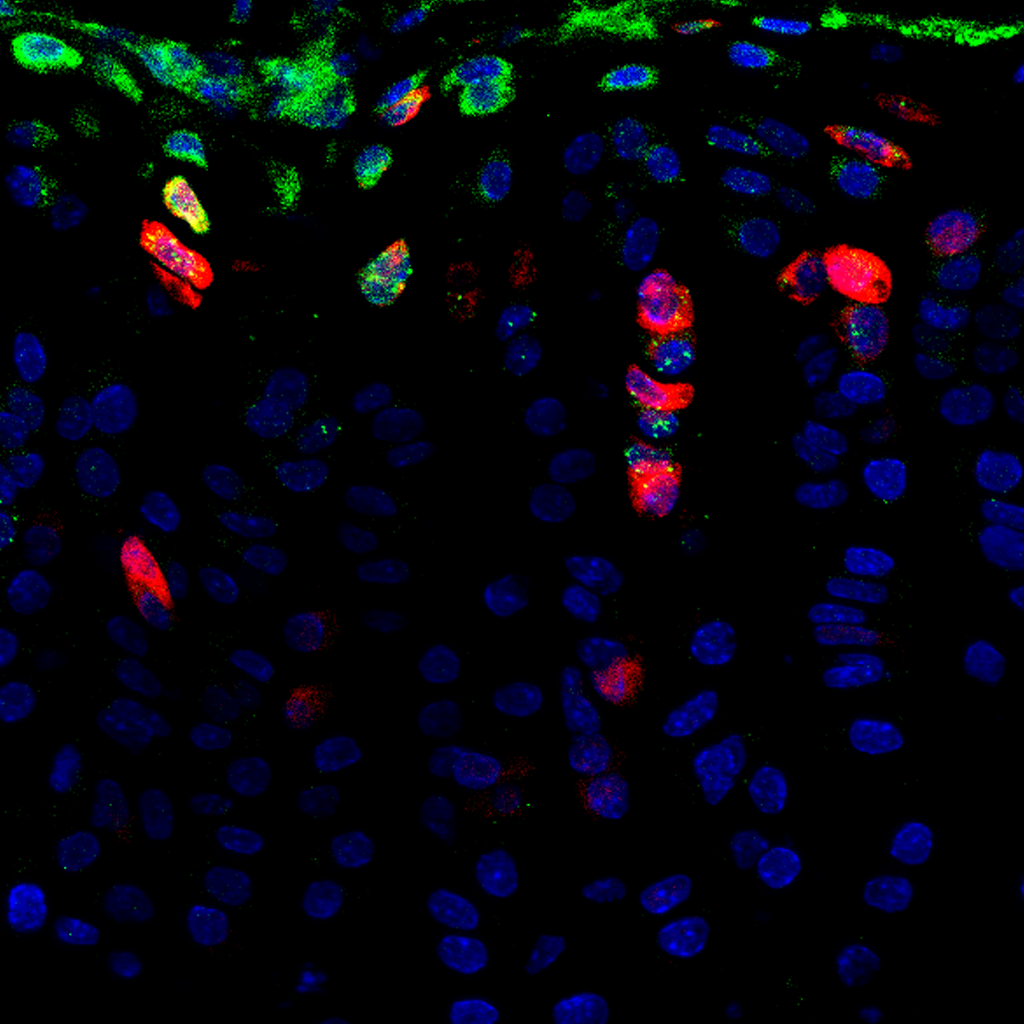

Supplement: Supplementary file 2 — Source Data Fig. 2 [file 44319_2024_93_MOESM2_ESM.zip › Figure2/2F/TM1M_D1_pthlp_td_merge.tif]

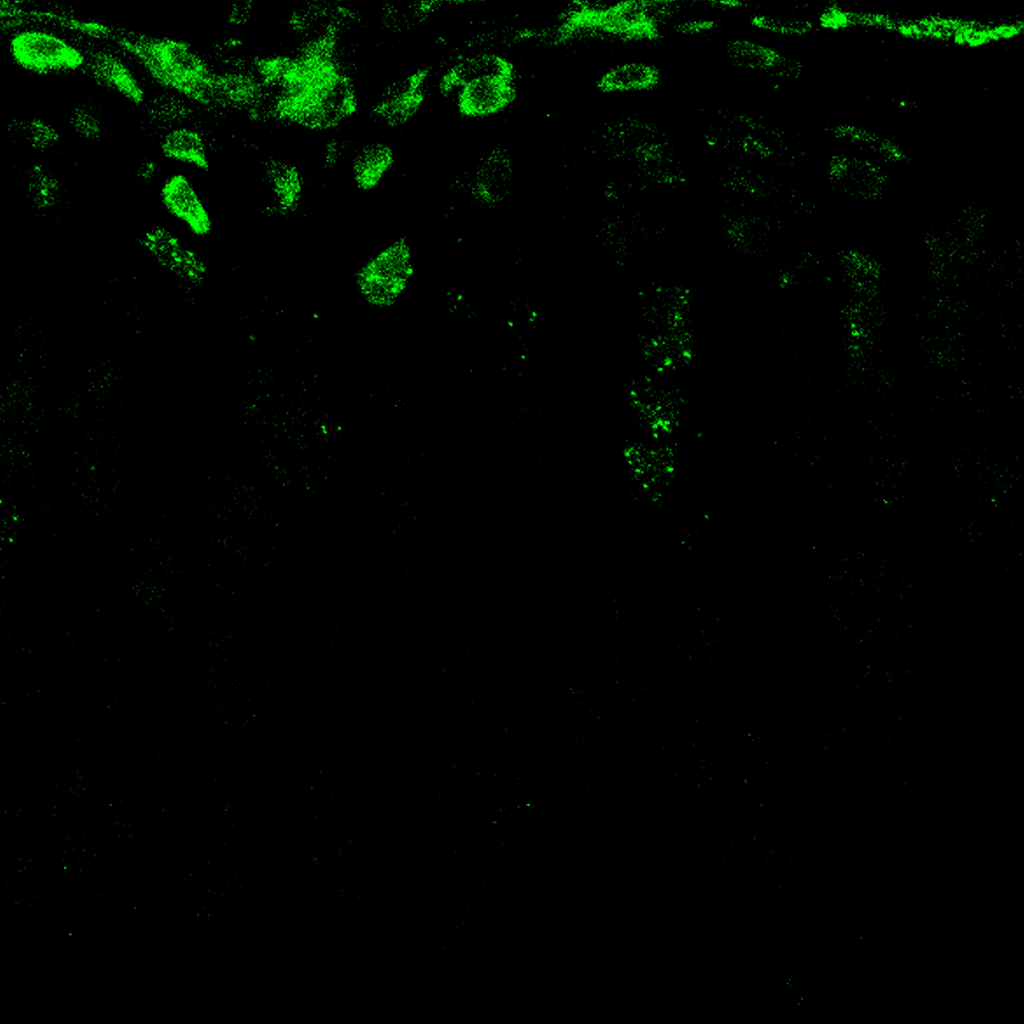

Supplement: Supplementary file 2 — Source Data Fig. 2 [file 44319_2024_93_MOESM2_ESM.zip › Figure2/2F/TM1M_D1_pthlp_td_pthlp_green.tif]

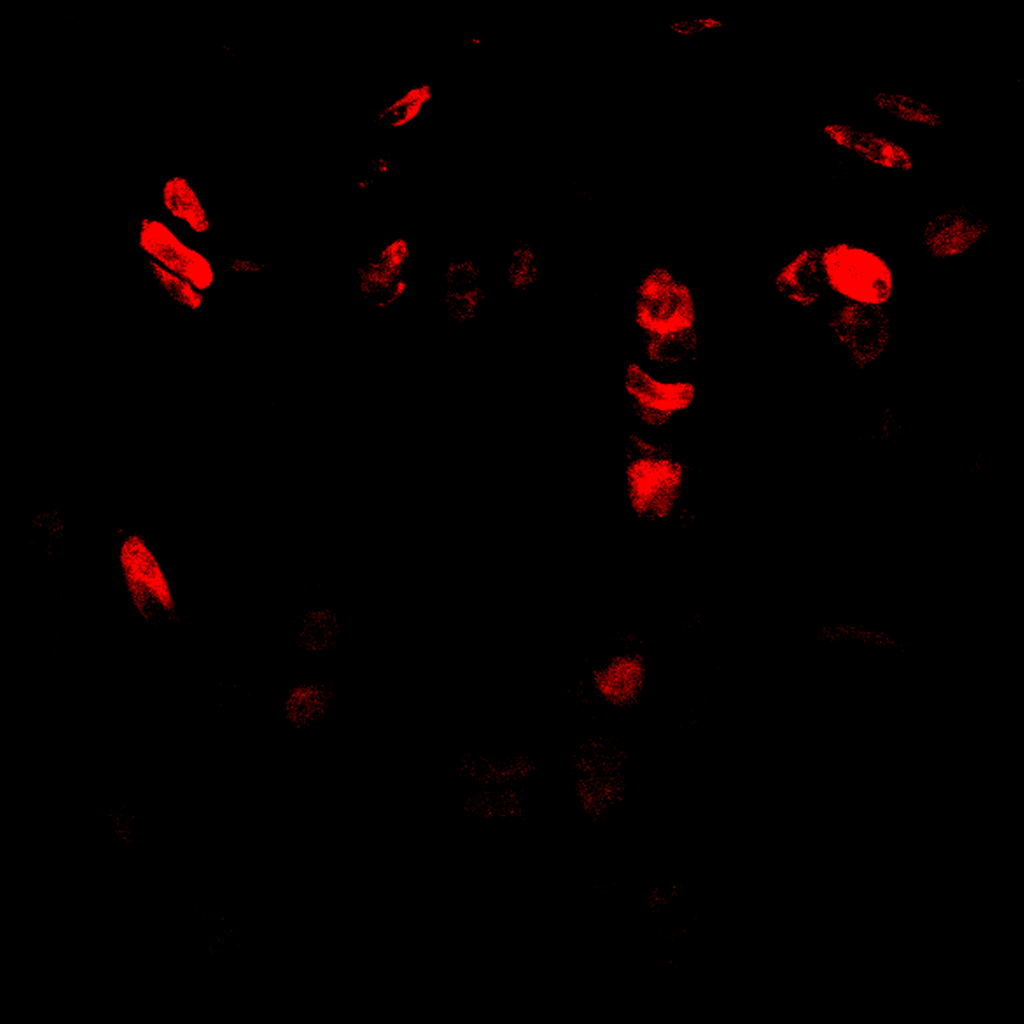

Supplement: Supplementary file 2 — Source Data Fig. 2 [file 44319_2024_93_MOESM2_ESM.zip › Figure2/2F/TM1M_D1_pthlp_td_td_red.tif]

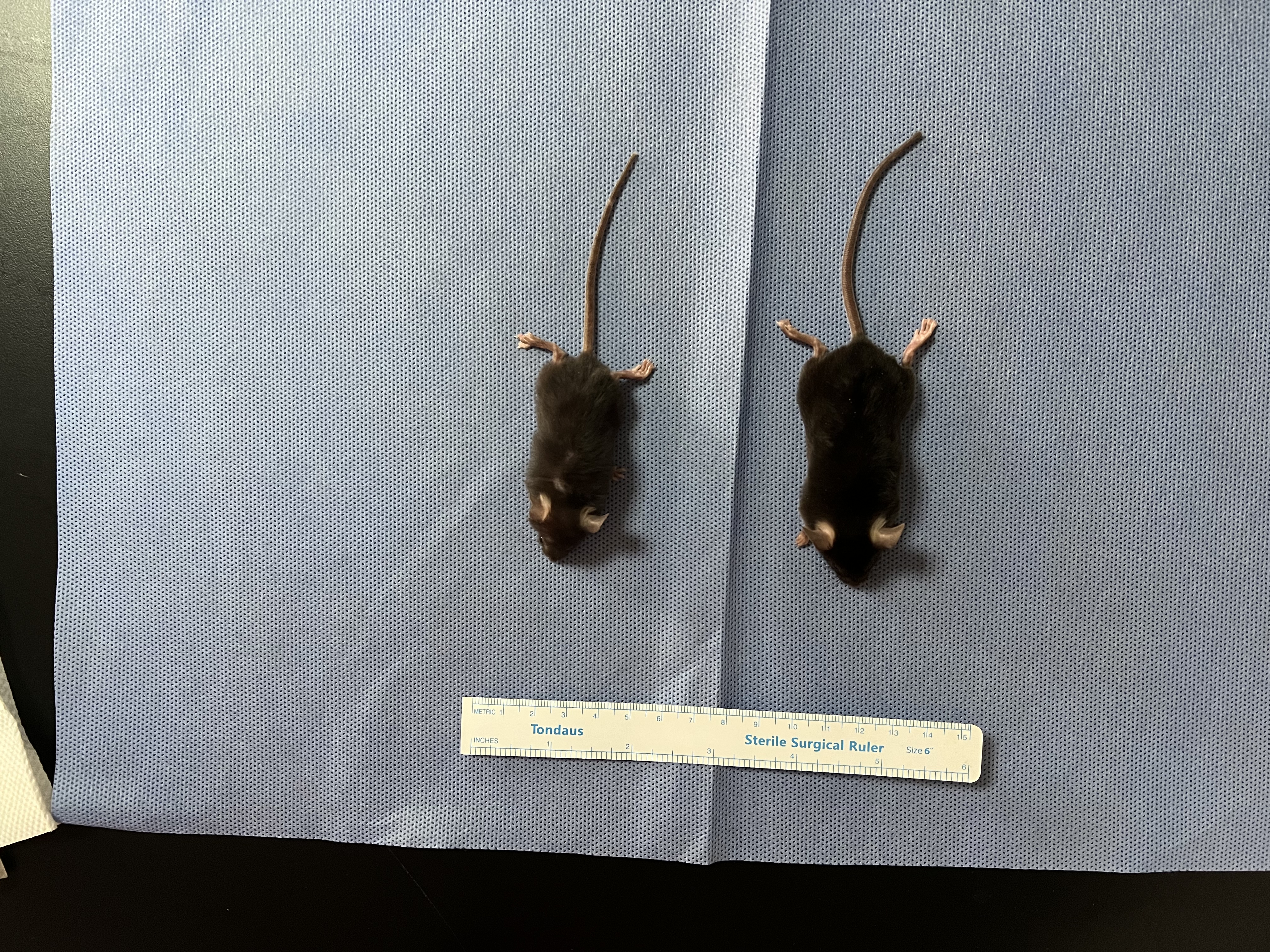

Supplement: Supplementary file 3 — Source Data Fig. 3 [file 44319_2024_93_MOESM3_ESM.zip › Figure3/3A/CTRL VS DTA.jpg]

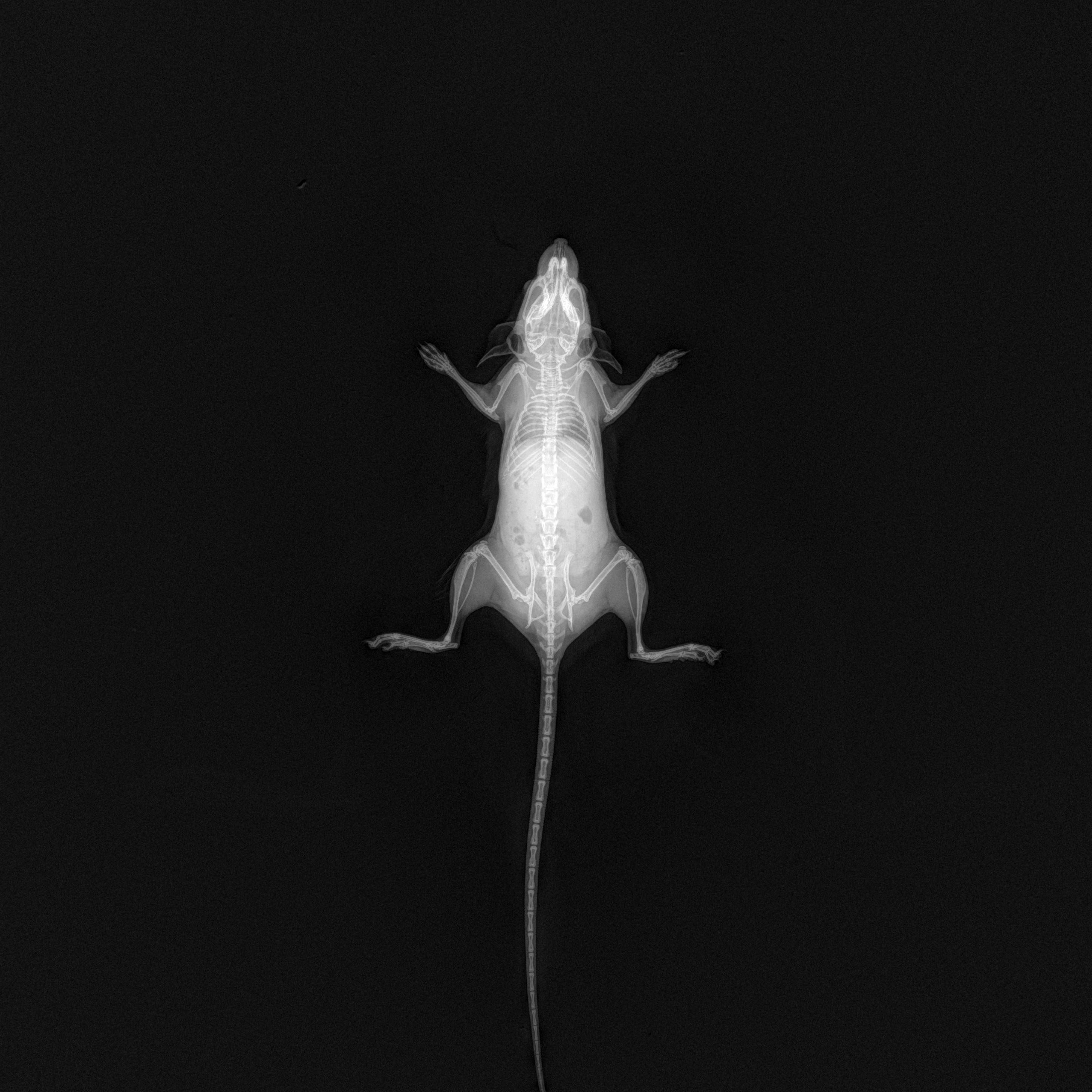

Supplement: Supplementary file 3 — Source Data Fig. 3 [file 44319_2024_93_MOESM3_ESM.zip › Figure3/3B/Ctrl.tif]

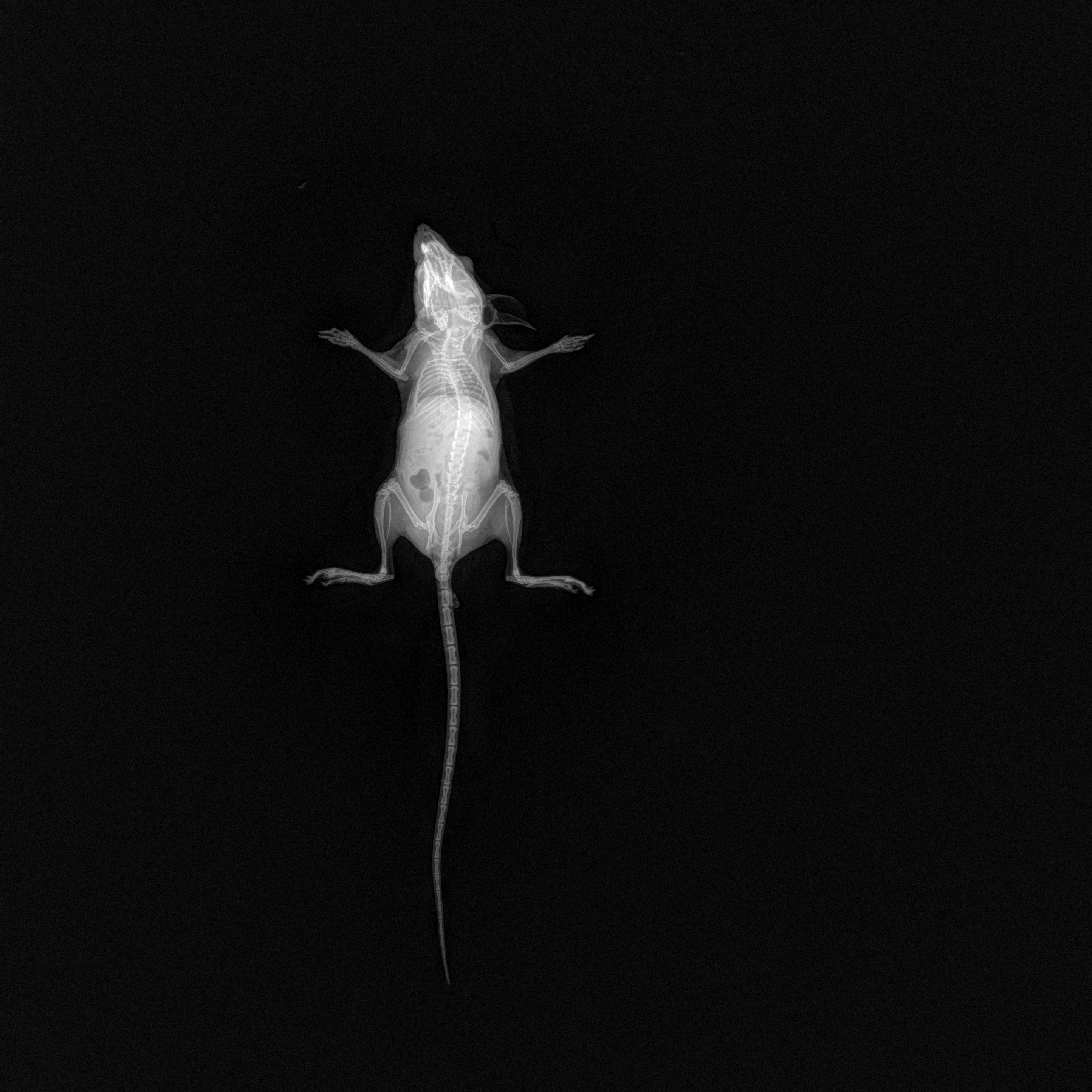

Supplement: Supplementary file 3 — Source Data Fig. 3 [file 44319_2024_93_MOESM3_ESM.zip › Figure3/3B/dta.tif]

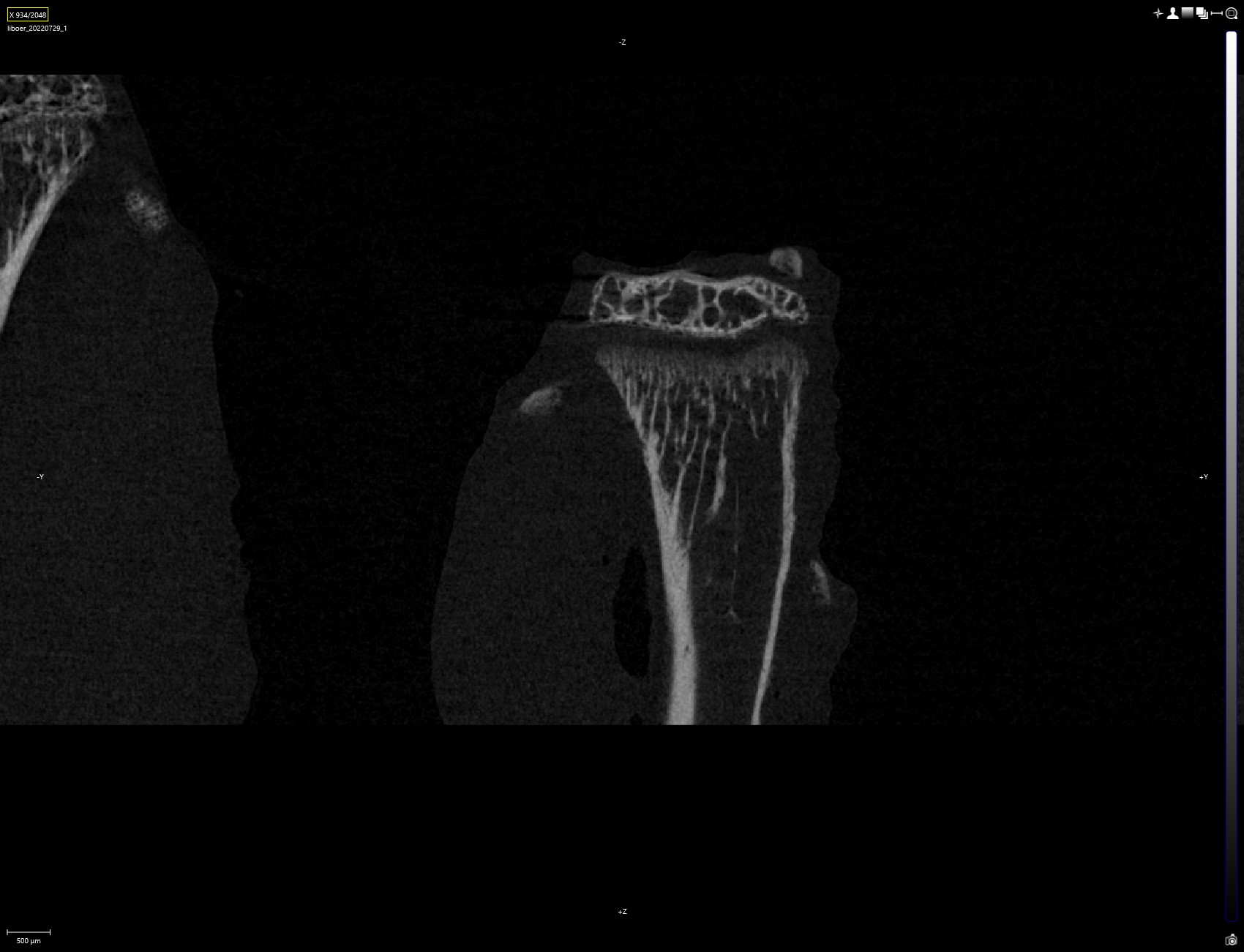

Supplement: Supplementary file 3 — Source Data Fig. 3 [file 44319_2024_93_MOESM3_ESM.zip › Figure3/3C/Ctrl.jpg]

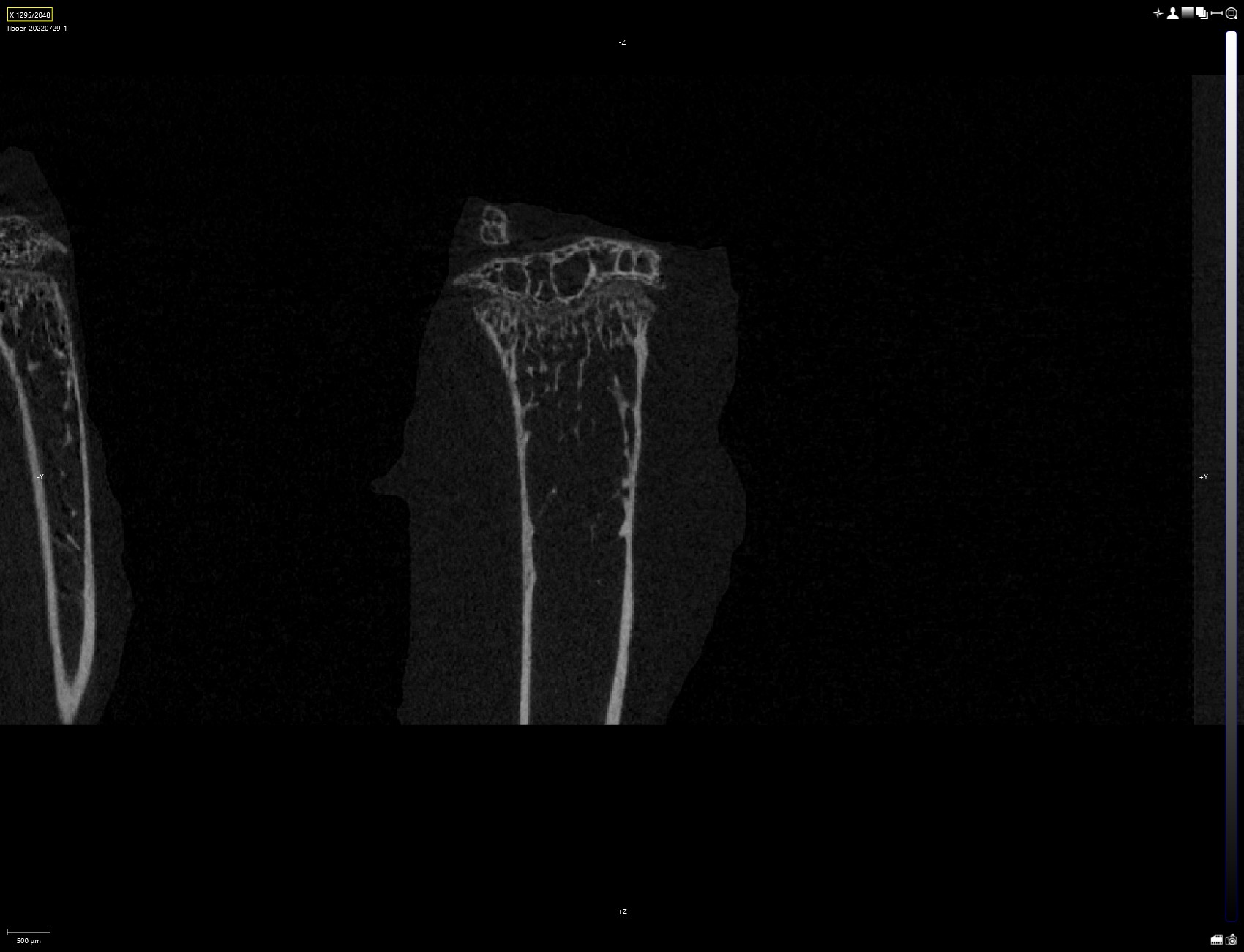

Supplement: Supplementary file 3 — Source Data Fig. 3 [file 44319_2024_93_MOESM3_ESM.zip › Figure3/3C/DTA.jpg]

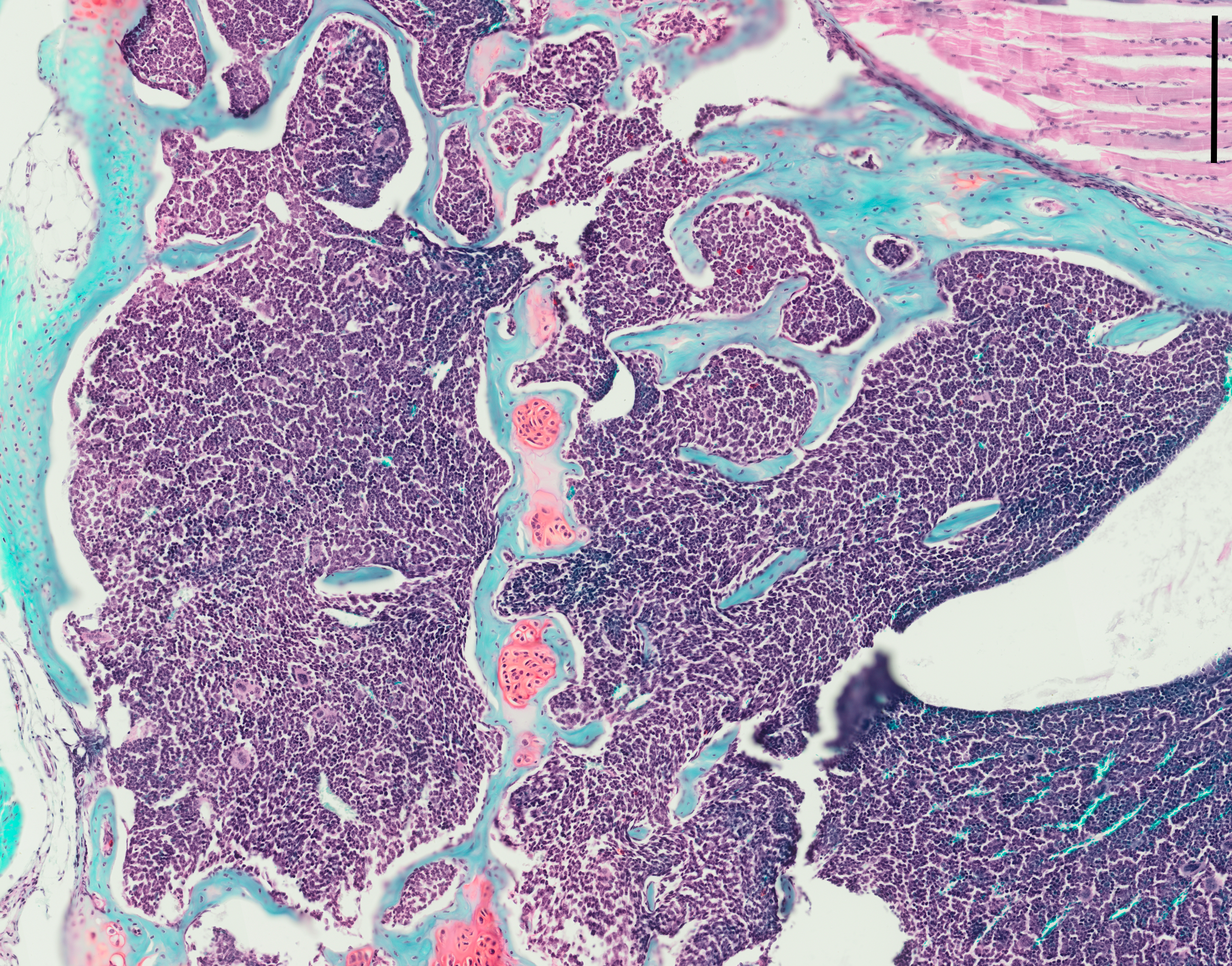

Supplement: Supplementary file 3 — Source Data Fig. 3 [file 44319_2024_93_MOESM3_ESM.zip › Figure3/3D/DTA_GP.tif]

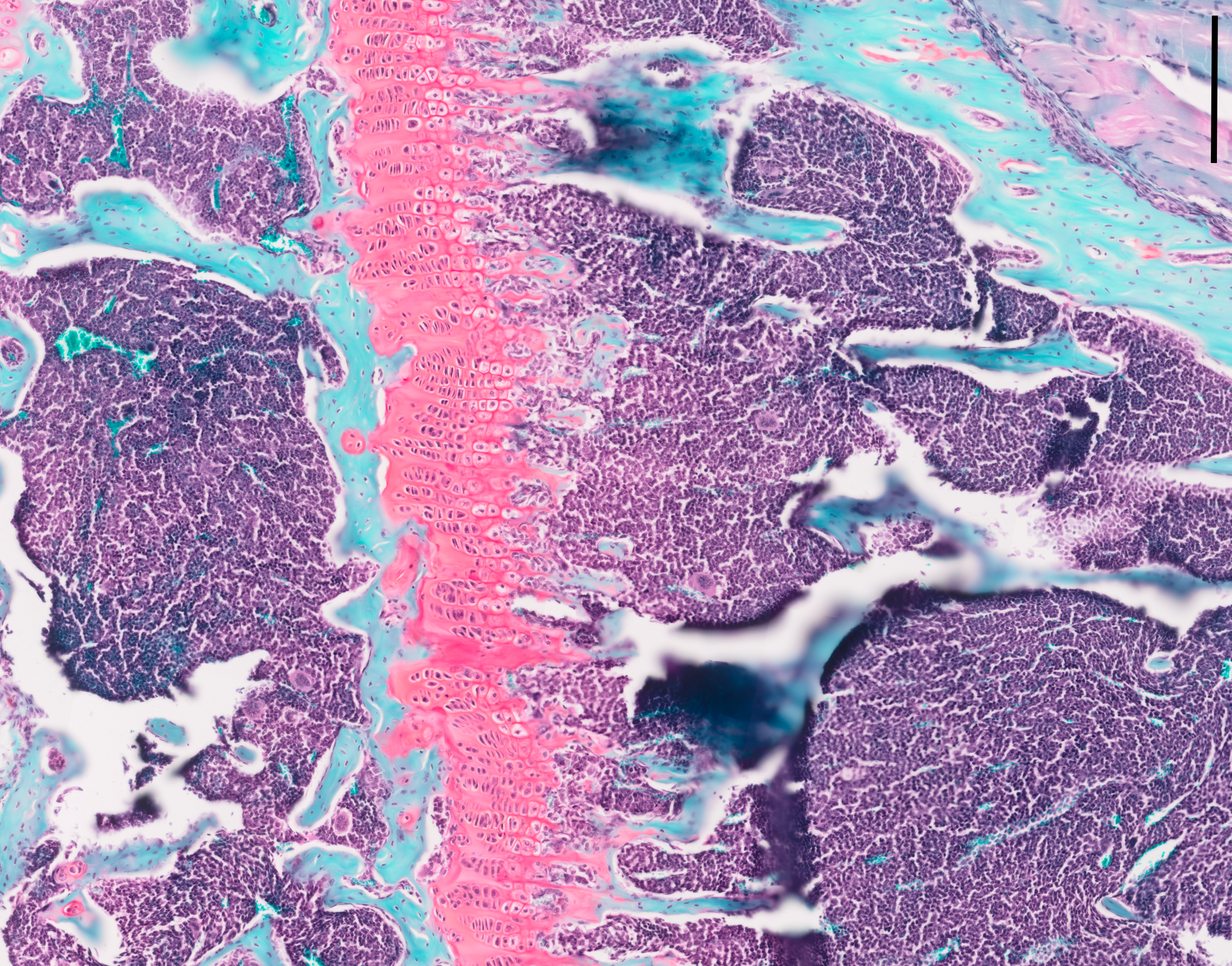

Supplement: Supplementary file 3 — Source Data Fig. 3 [file 44319_2024_93_MOESM3_ESM.zip › Figure3/3D/WT_GP.tif]

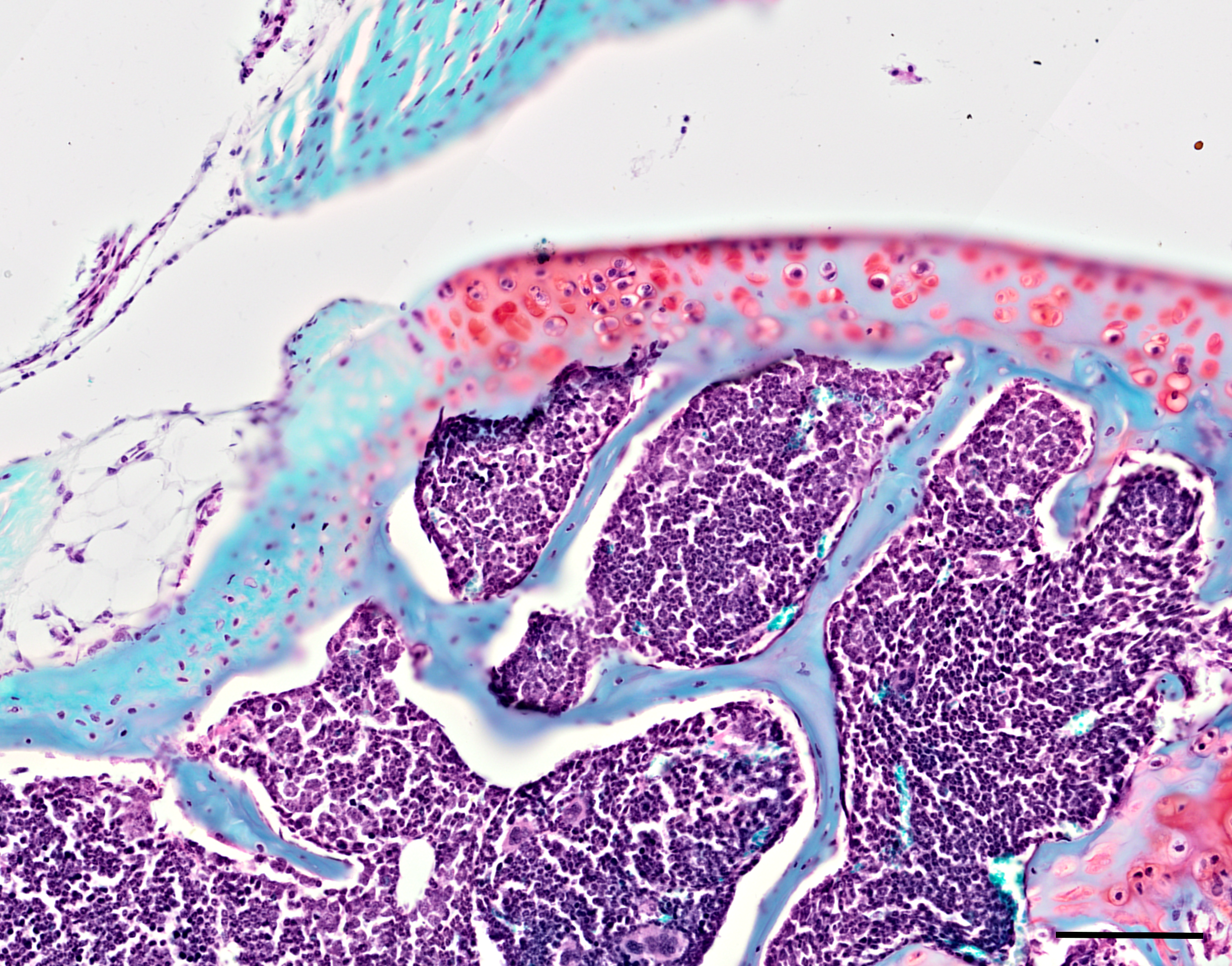

Supplement: Supplementary file 3 — Source Data Fig. 3 [file 44319_2024_93_MOESM3_ESM.zip › Figure3/3E/DTA_AC.tif]

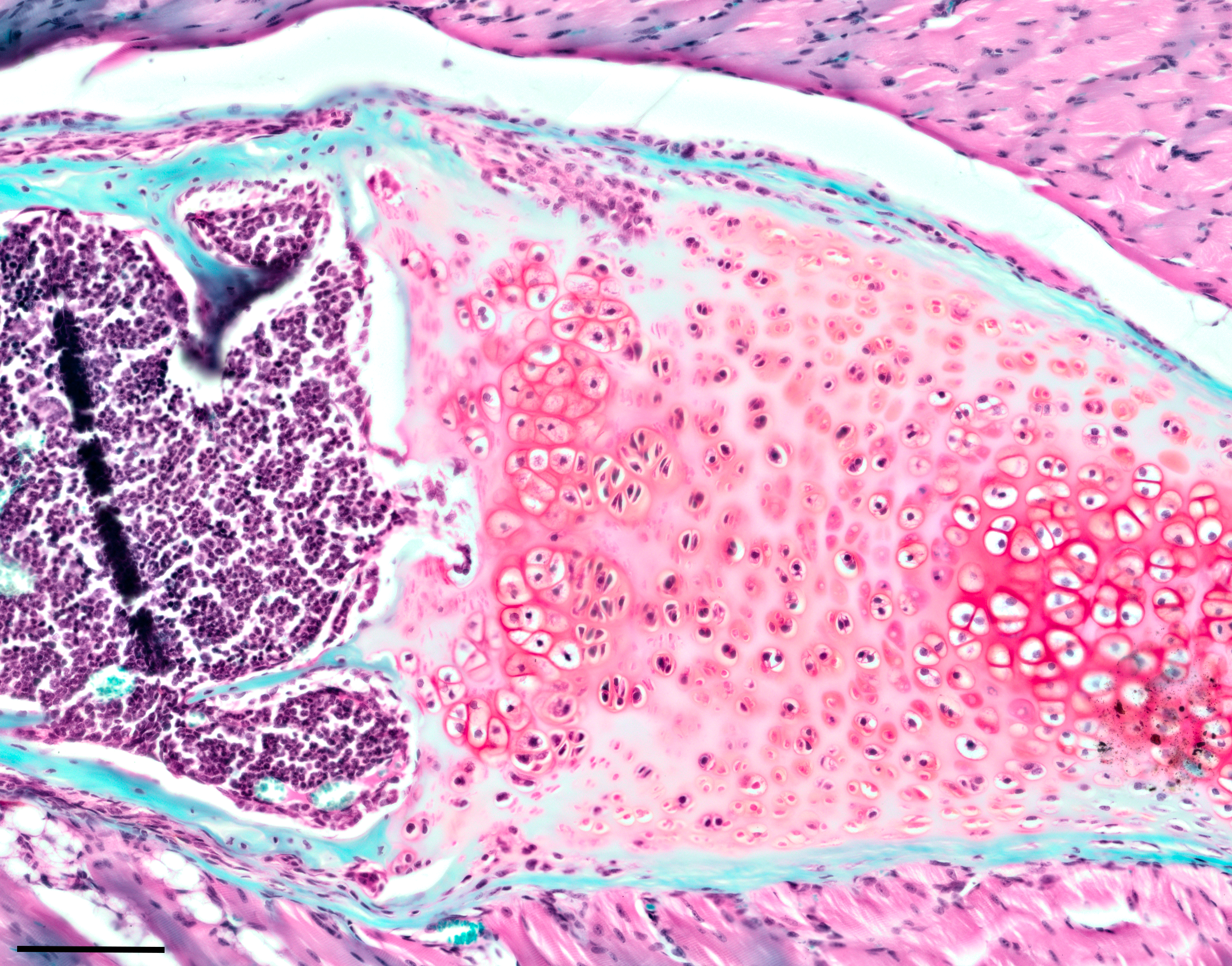

Supplement: Supplementary file 3 — Source Data Fig. 3 [file 44319_2024_93_MOESM3_ESM.zip › Figure3/3E/DTA_CC.tif]

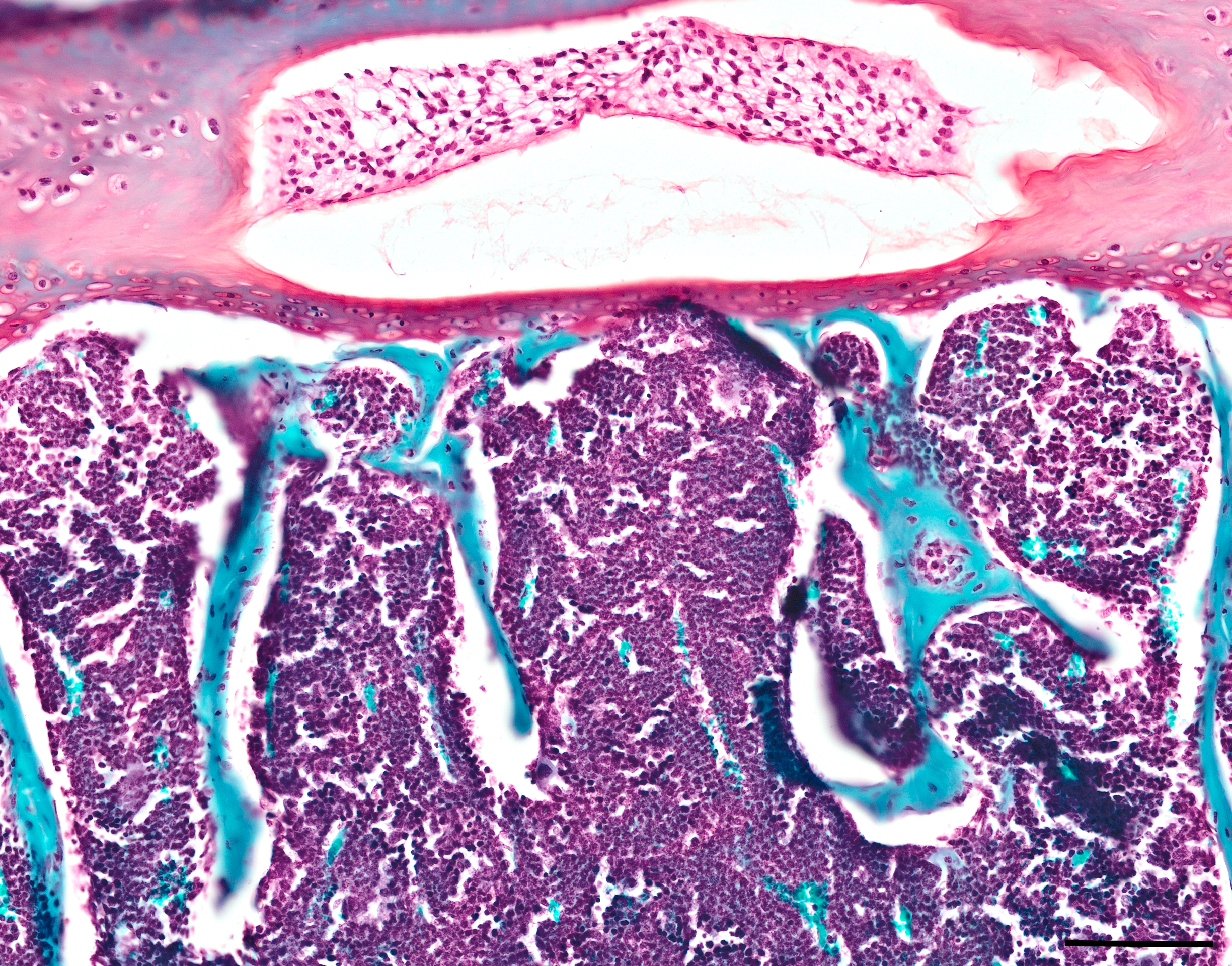

Supplement: Supplementary file 3 — Source Data Fig. 3 [file 44319_2024_93_MOESM3_ESM.zip › Figure3/3E/DTA_SP.tif]

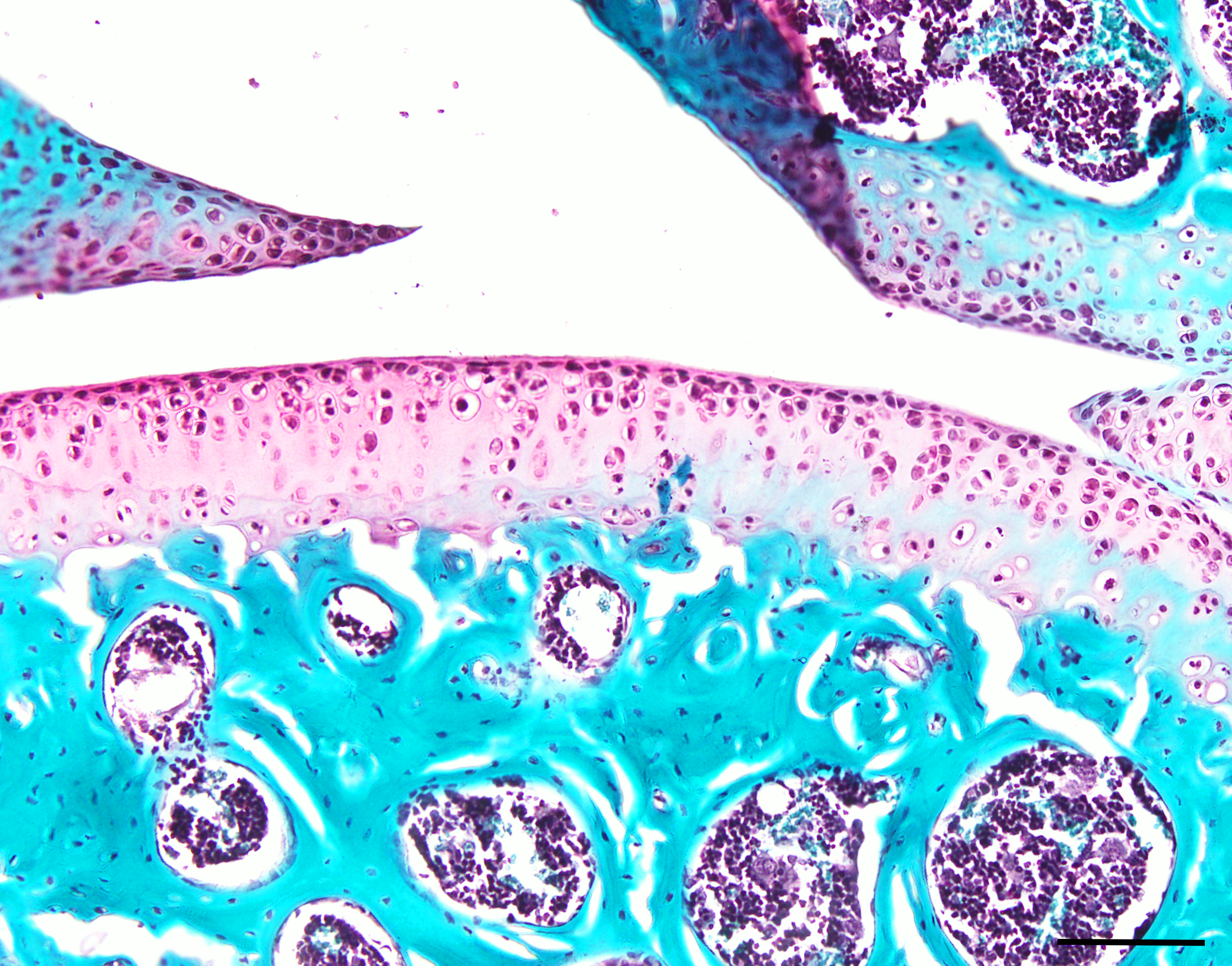

Supplement: Supplementary file 3 — Source Data Fig. 3 [file 44319_2024_93_MOESM3_ESM.zip › Figure3/3E/WT_AC-2.tif]

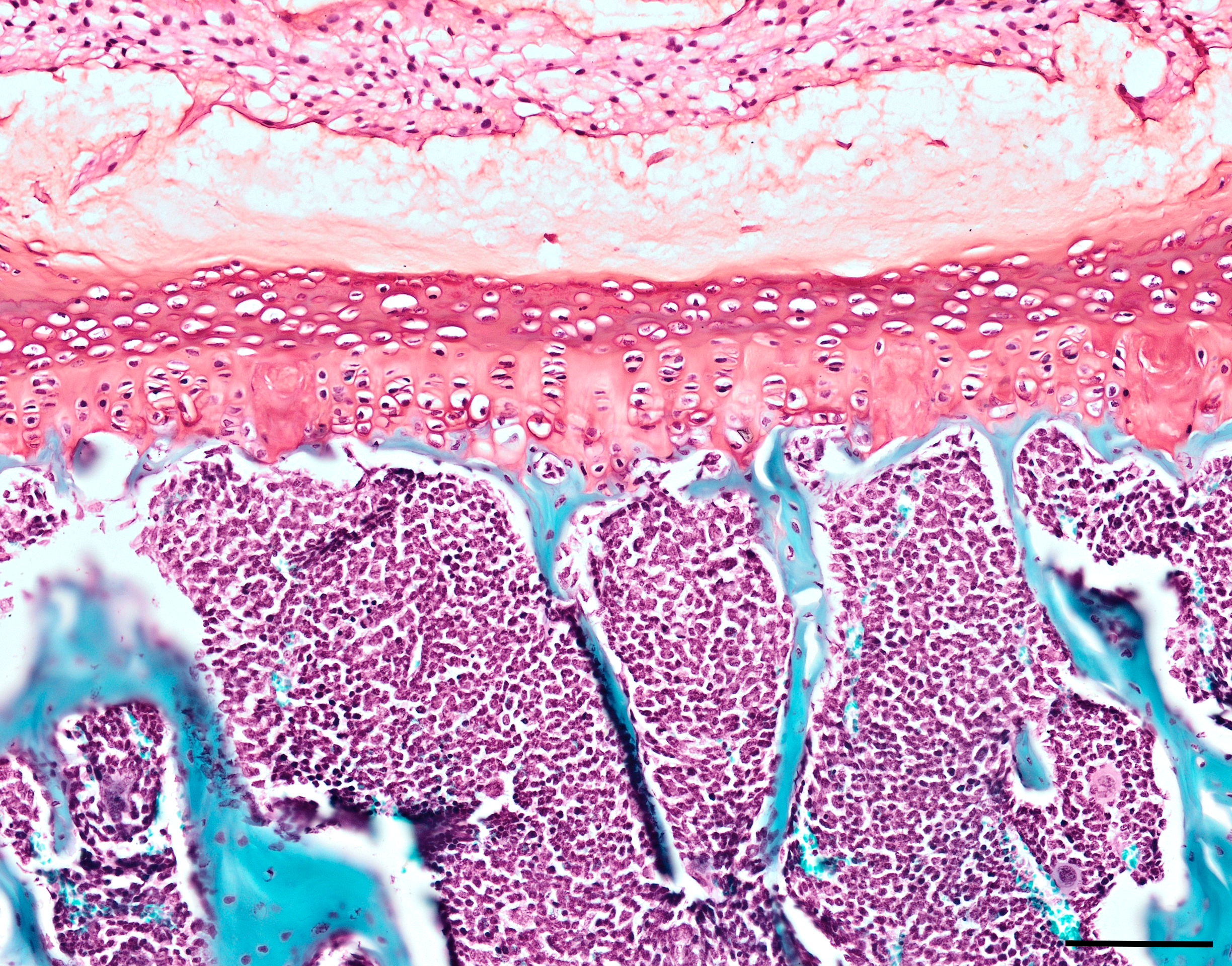

Supplement: Supplementary file 3 — Source Data Fig. 3 [file 44319_2024_93_MOESM3_ESM.zip › Figure3/3E/WT_SP.tif]

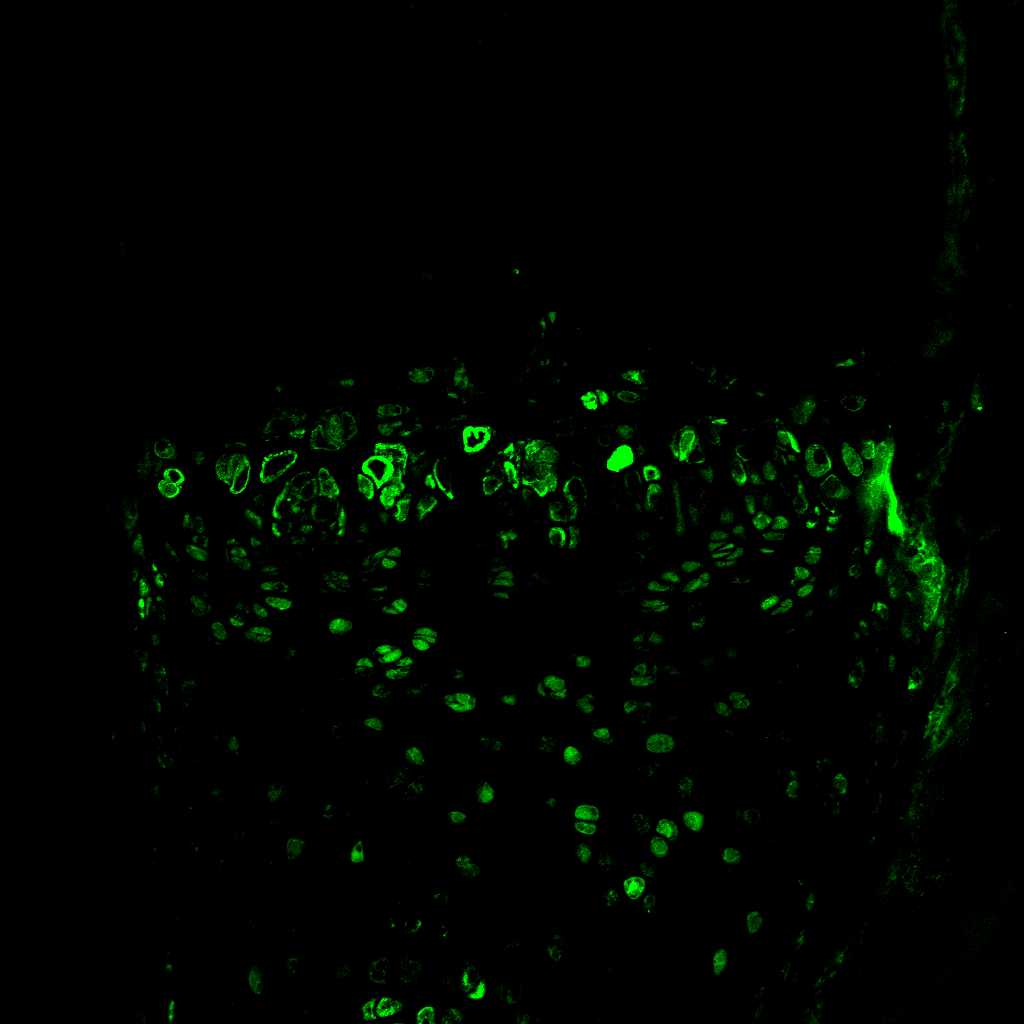

Supplement: Supplementary file 3 — Source Data Fig. 3 [file 44319_2024_93_MOESM3_ESM.zip › Figure3/3F/TM1M_1M_DTA_CC_20X_acan_green.tif]

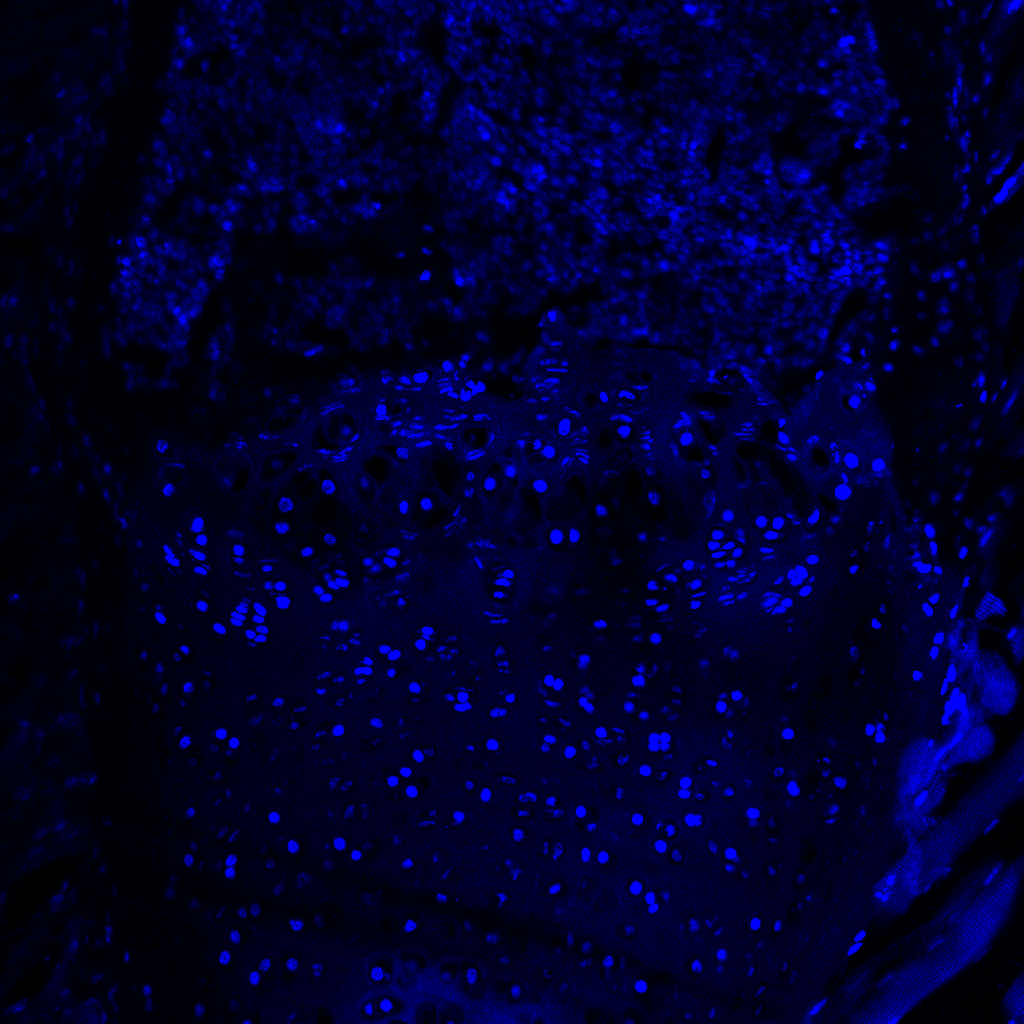

Supplement: Supplementary file 3 — Source Data Fig. 3 [file 44319_2024_93_MOESM3_ESM.zip › Figure3/3F/TM1M_1M_DTA_CC_20X_dapi_blue.tif]

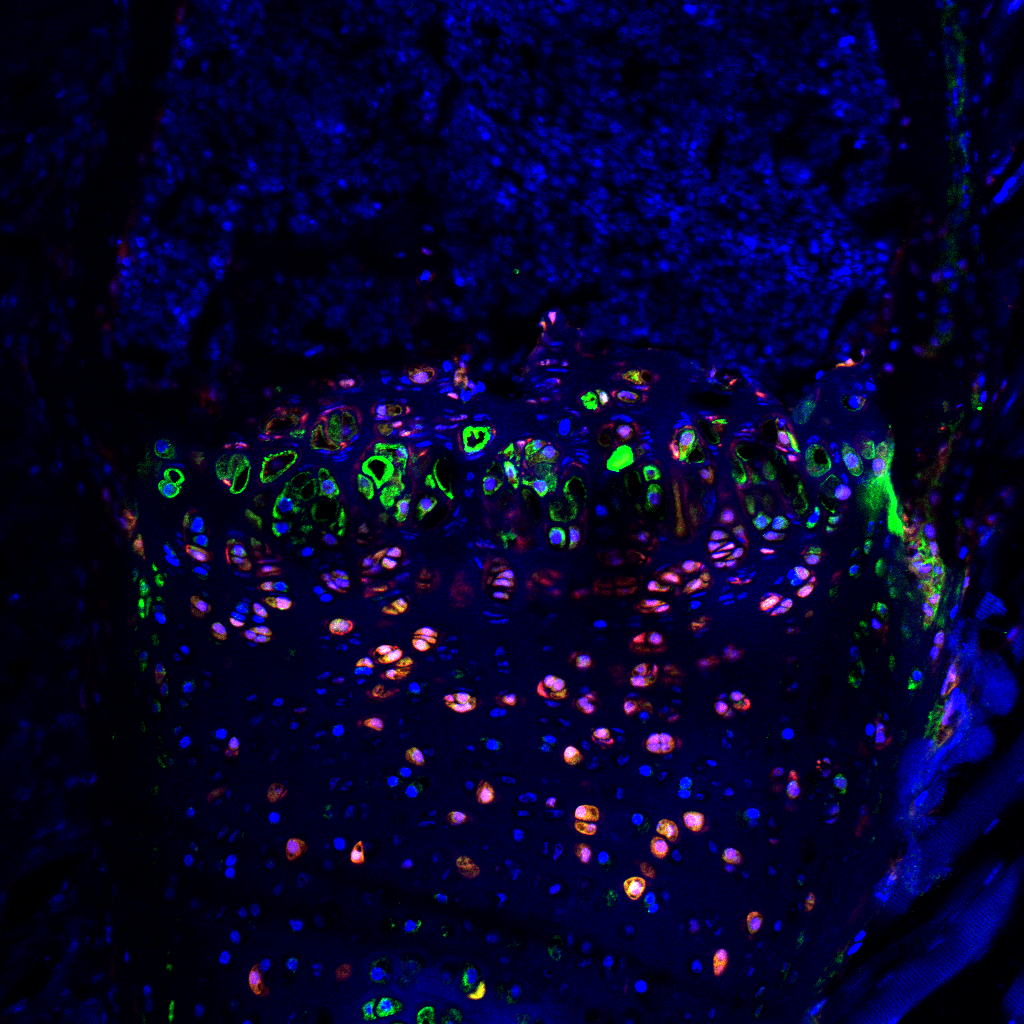

Supplement: Supplementary file 3 — Source Data Fig. 3 [file 44319_2024_93_MOESM3_ESM.zip › Figure3/3F/TM1M_1M_DTA_CC_20X_merge.tif]

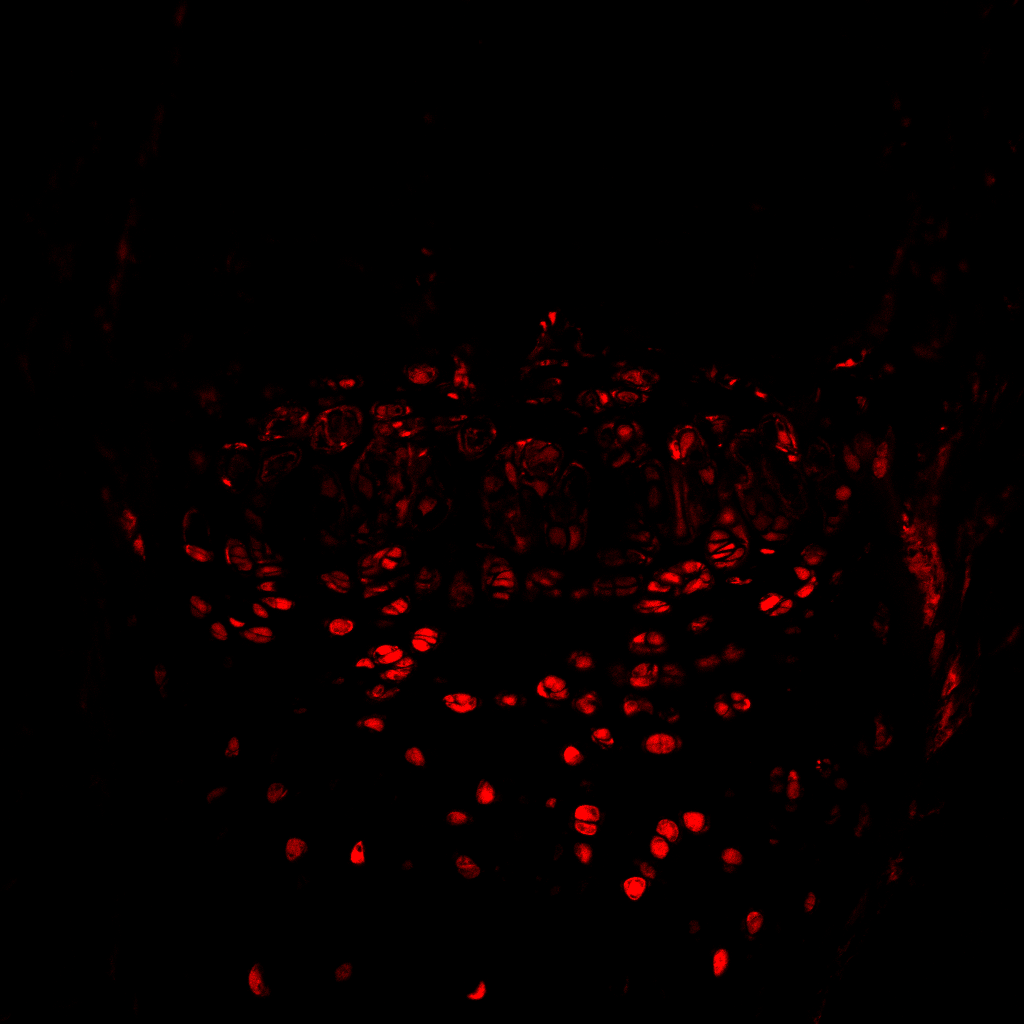

Supplement: Supplementary file 3 — Source Data Fig. 3 [file 44319_2024_93_MOESM3_ESM.zip › Figure3/3F/TM1M_1M_DTA_CC_20X_td_red.tif]

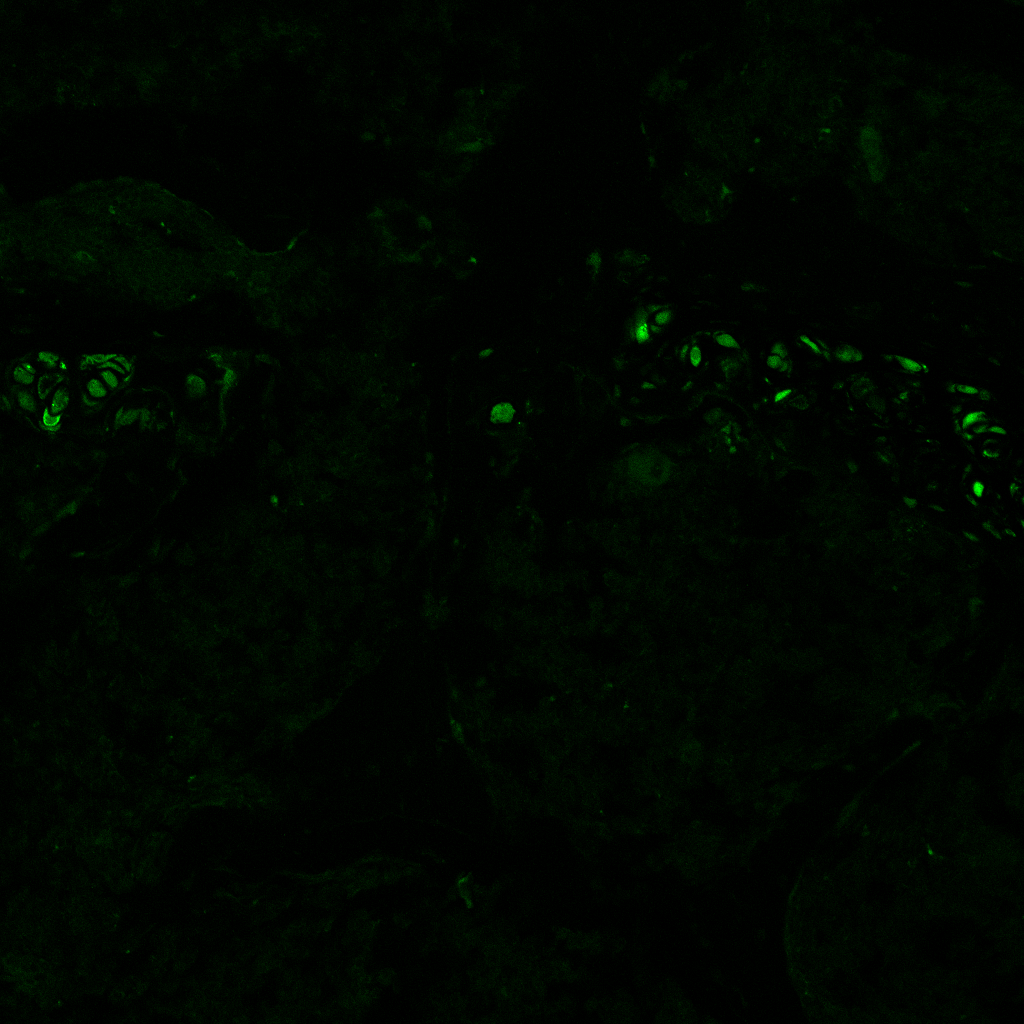

Supplement: Supplementary file 3 — Source Data Fig. 3 [file 44319_2024_93_MOESM3_ESM.zip › Figure3/3F/TM1M_1M_DTA_GP_20X_acan_green.tif]

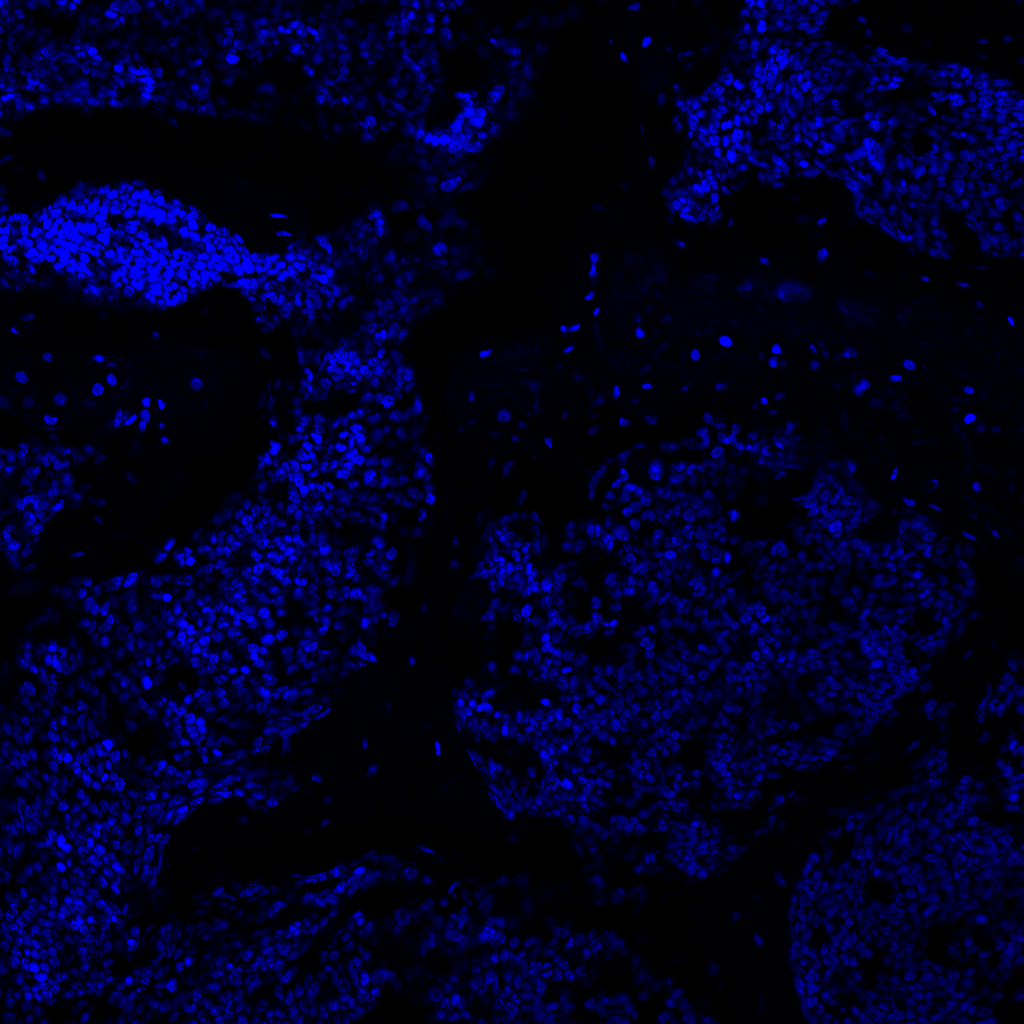

Supplement: Supplementary file 3 — Source Data Fig. 3 [file 44319_2024_93_MOESM3_ESM.zip › Figure3/3F/TM1M_1M_DTA_GP_20X_dapi_blue.tif]

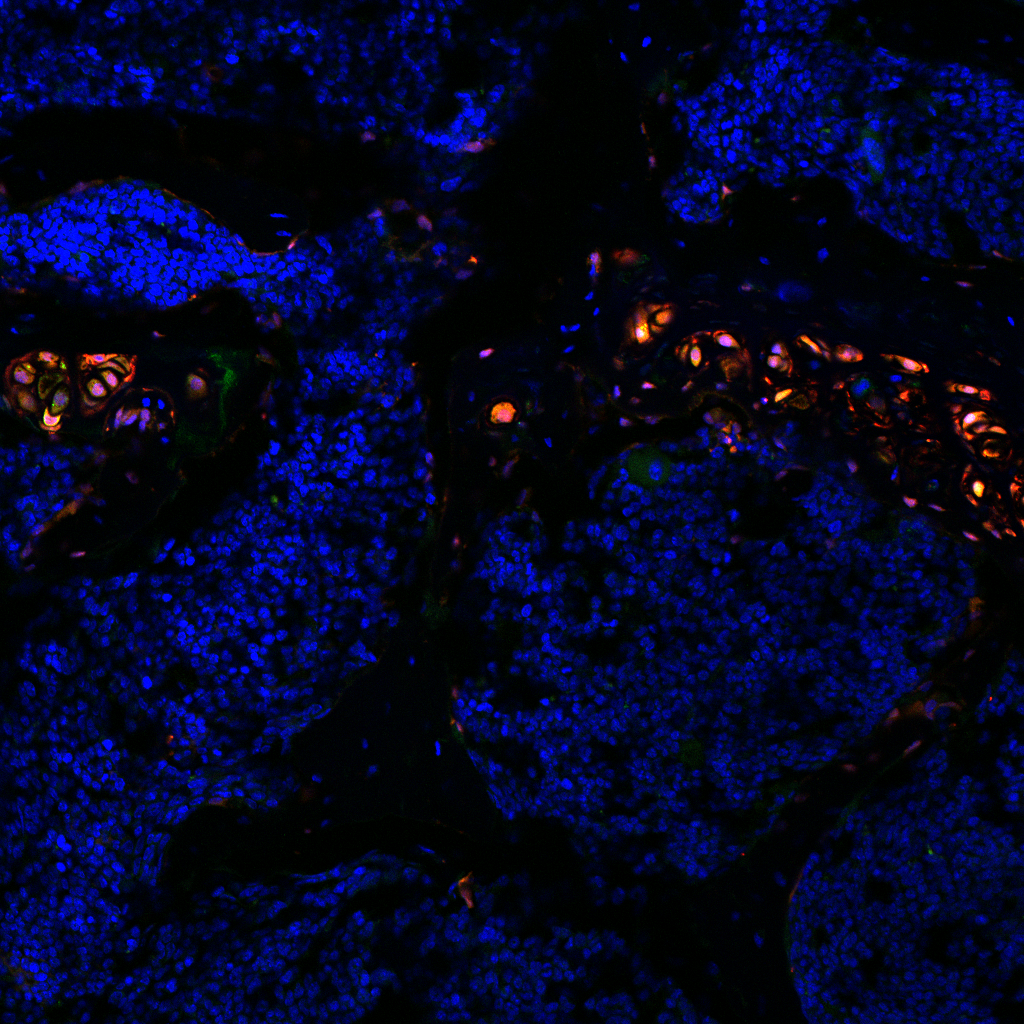

Supplement: Supplementary file 3 — Source Data Fig. 3 [file 44319_2024_93_MOESM3_ESM.zip › Figure3/3F/TM1M_1M_DTA_GP_20X_merge.tif]

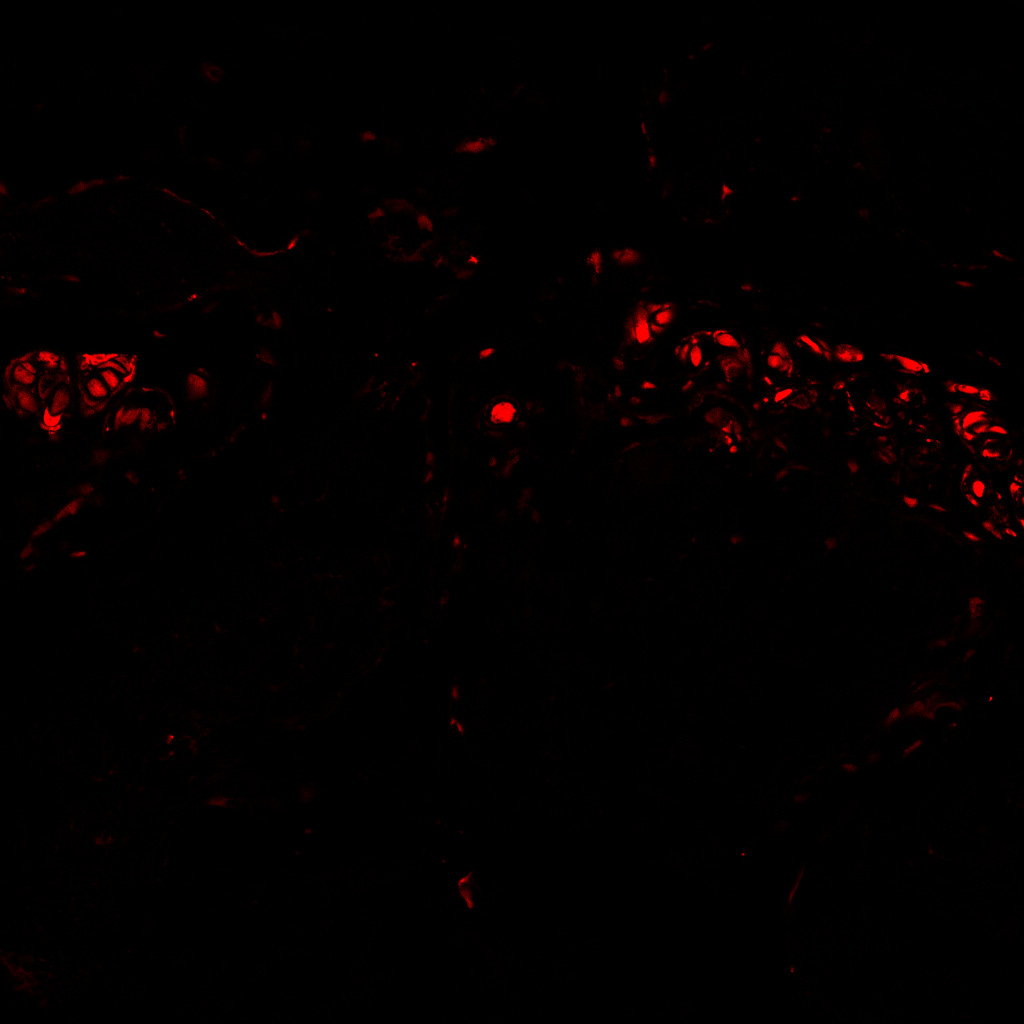

Supplement: Supplementary file 3 — Source Data Fig. 3 [file 44319_2024_93_MOESM3_ESM.zip › Figure3/3F/TM1M_1M_DTA_GP_20X_td_red.tif]

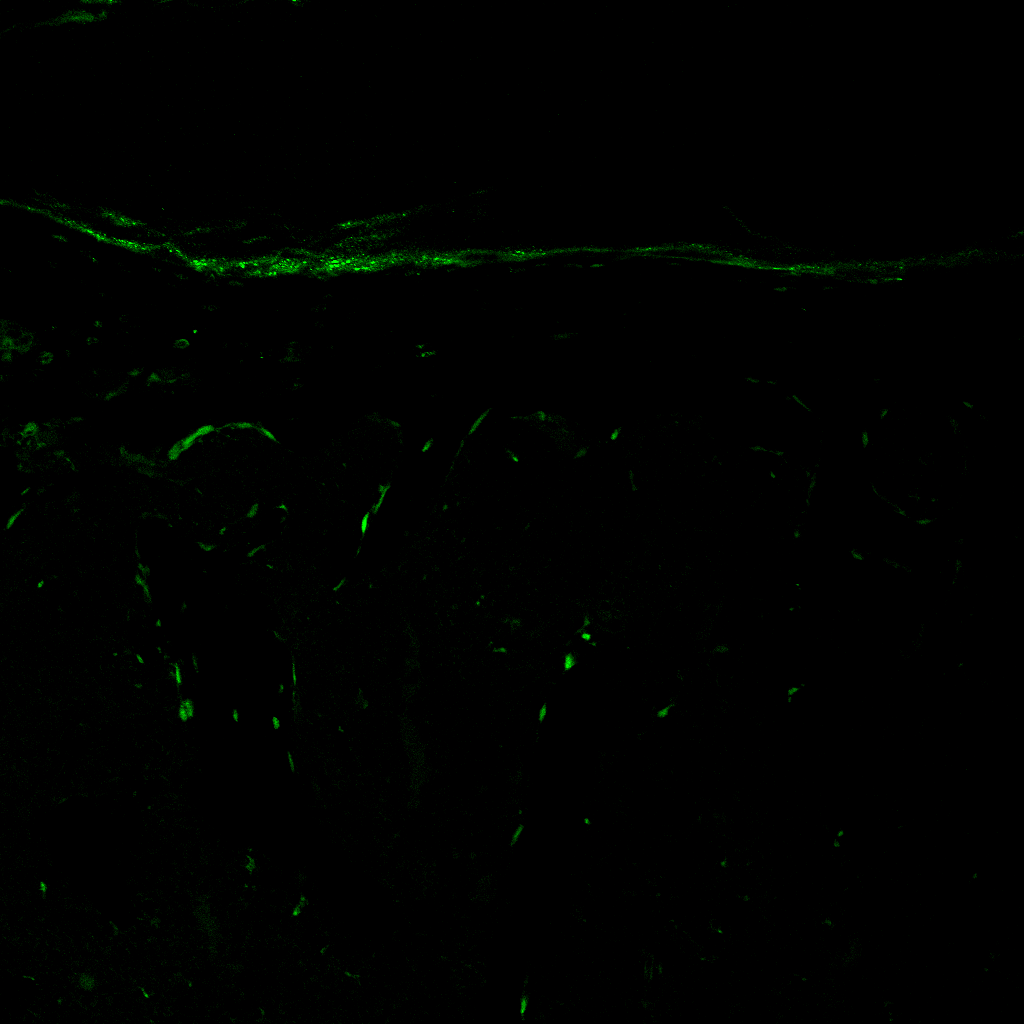

Supplement: Supplementary file 3 — Source Data Fig. 3 [file 44319_2024_93_MOESM3_ESM.zip › Figure3/3F/TM1M_1M_DTA_SP_20X_acan_green.tif]

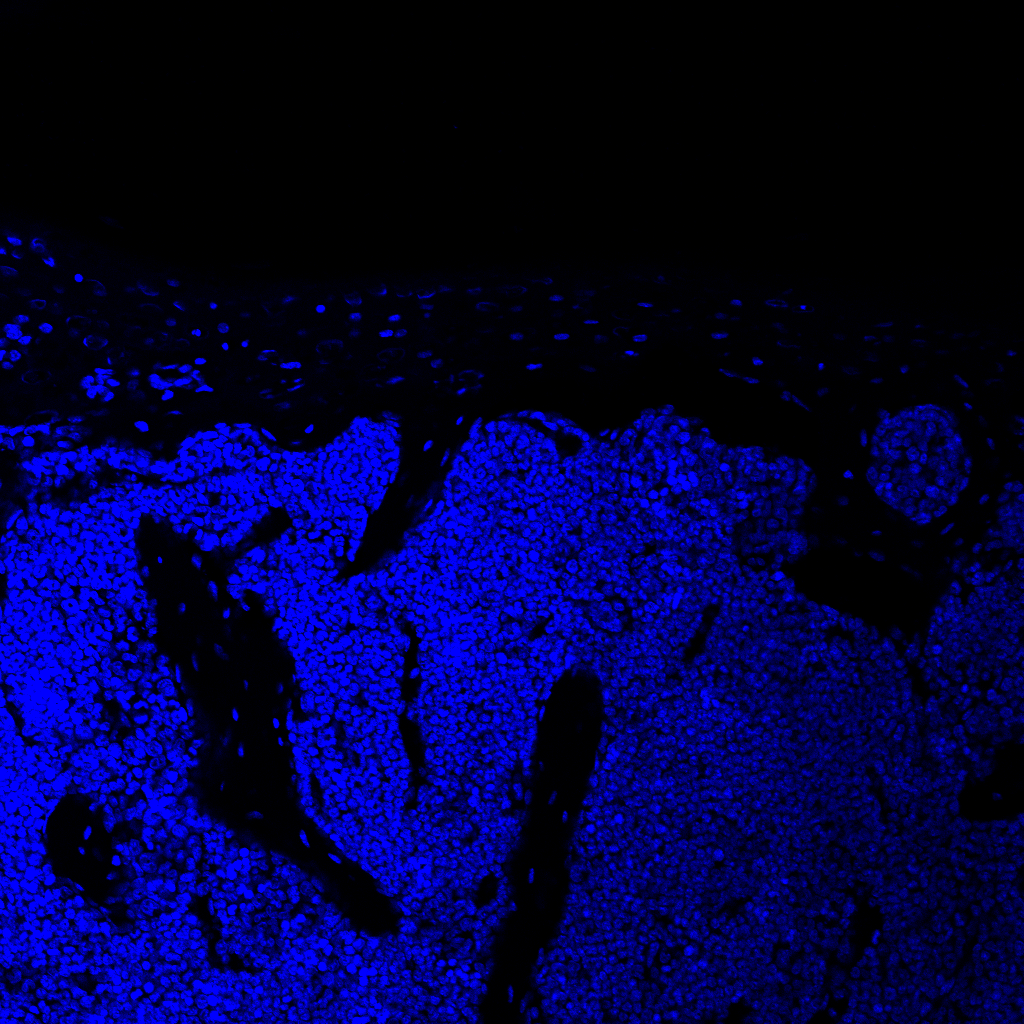

Supplement: Supplementary file 3 — Source Data Fig. 3 [file 44319_2024_93_MOESM3_ESM.zip › Figure3/3F/TM1M_1M_DTA_SP_20X_dapi_blue.tif]

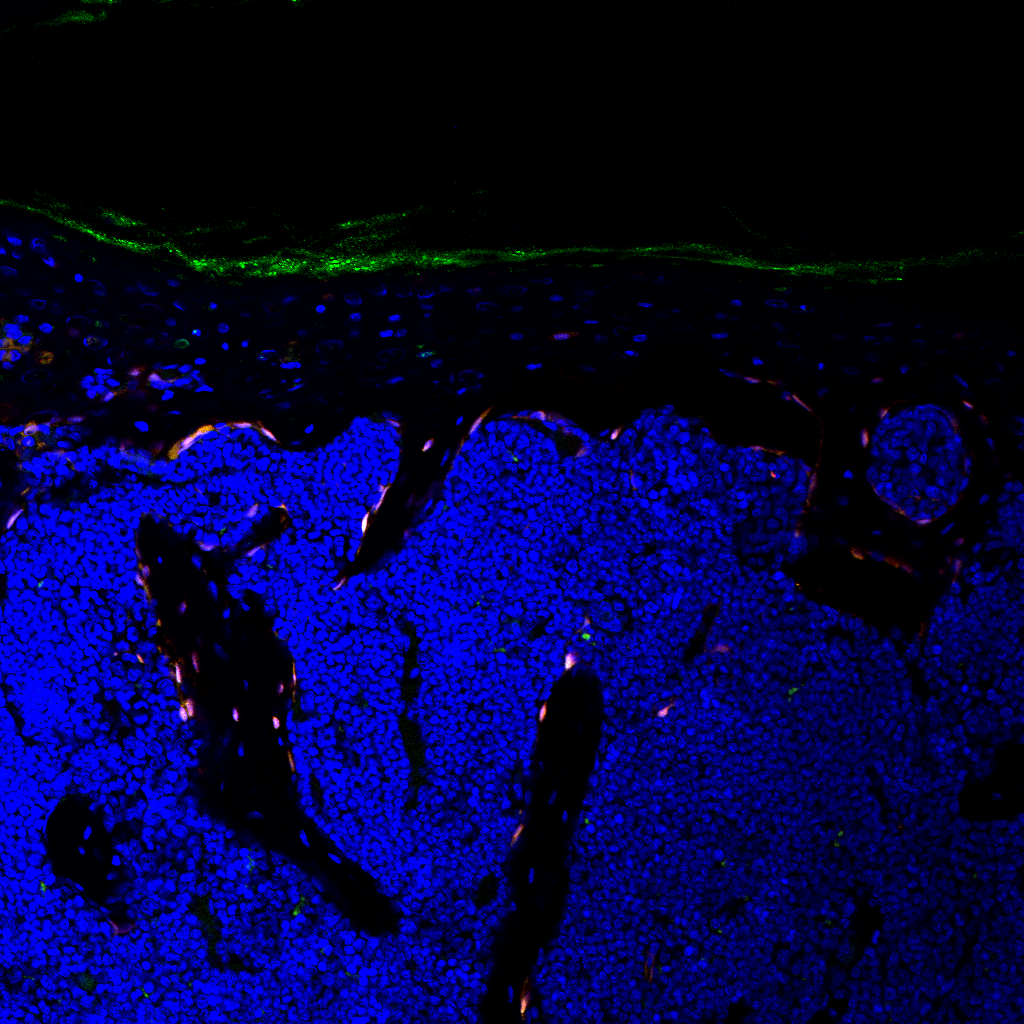

Supplement: Supplementary file 3 — Source Data Fig. 3 [file 44319_2024_93_MOESM3_ESM.zip › Figure3/3F/TM1M_1M_DTA_SP_20X_merge.tif]

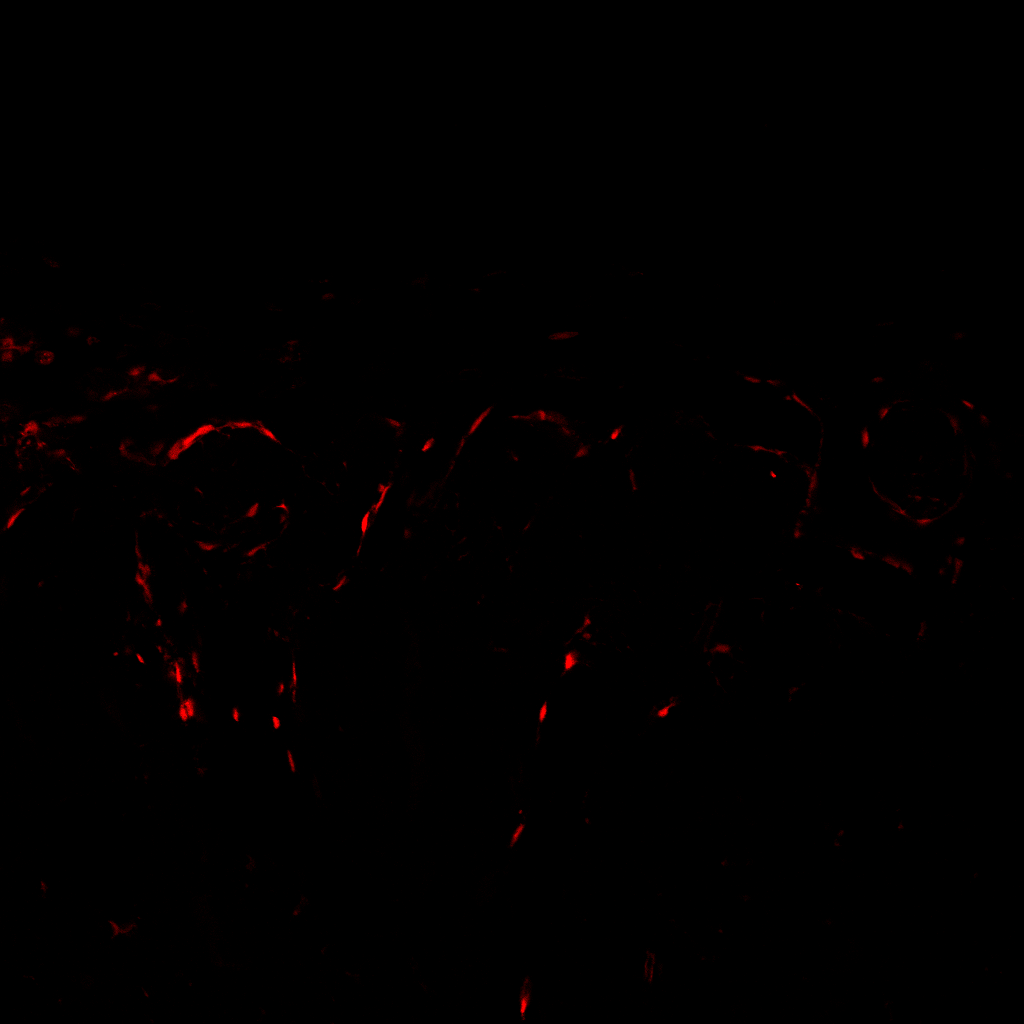

Supplement: Supplementary file 3 — Source Data Fig. 3 [file 44319_2024_93_MOESM3_ESM.zip › Figure3/3F/TM1M_1M_DTA_SP_20X_td_red.tif]

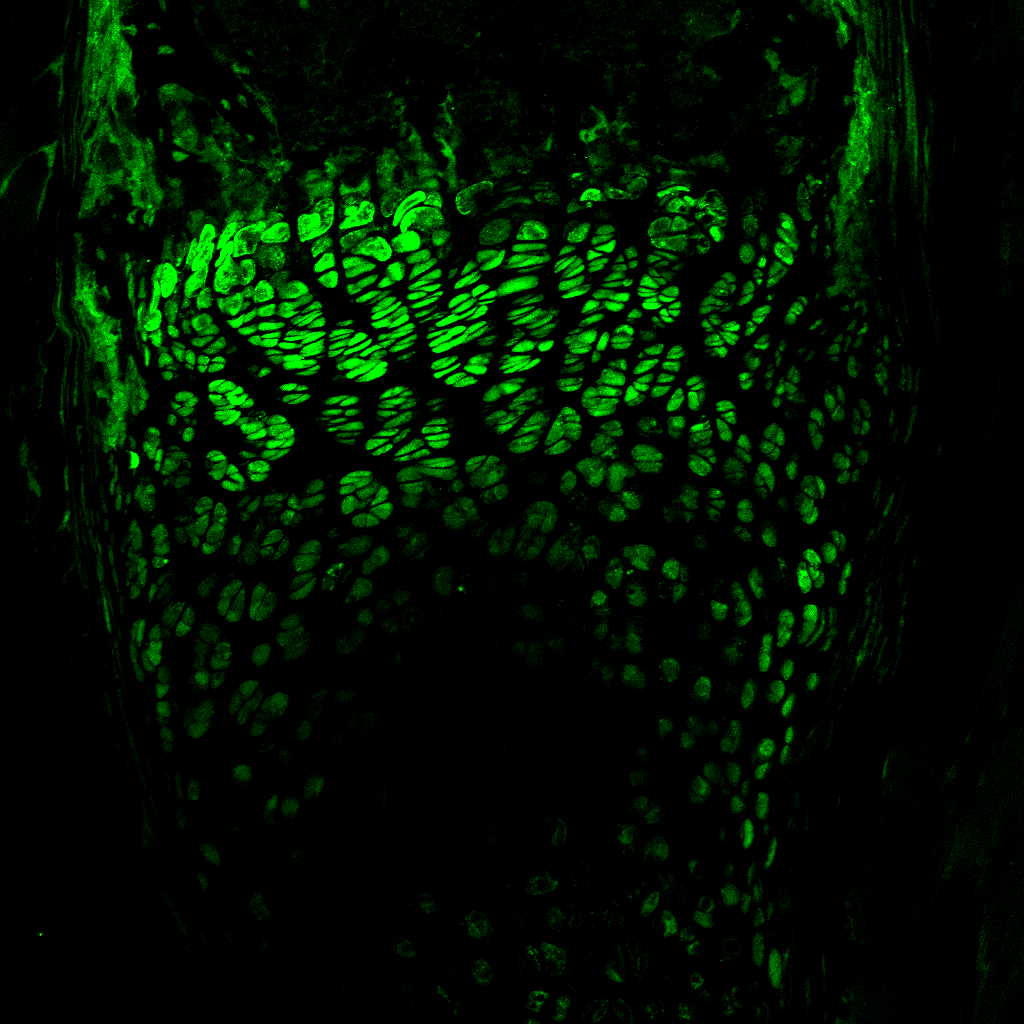

Supplement: Supplementary file 3 — Source Data Fig. 3 [file 44319_2024_93_MOESM3_ESM.zip › Figure3/3F/TM1M_1M_WT_CC_20X_acan_green.tif]

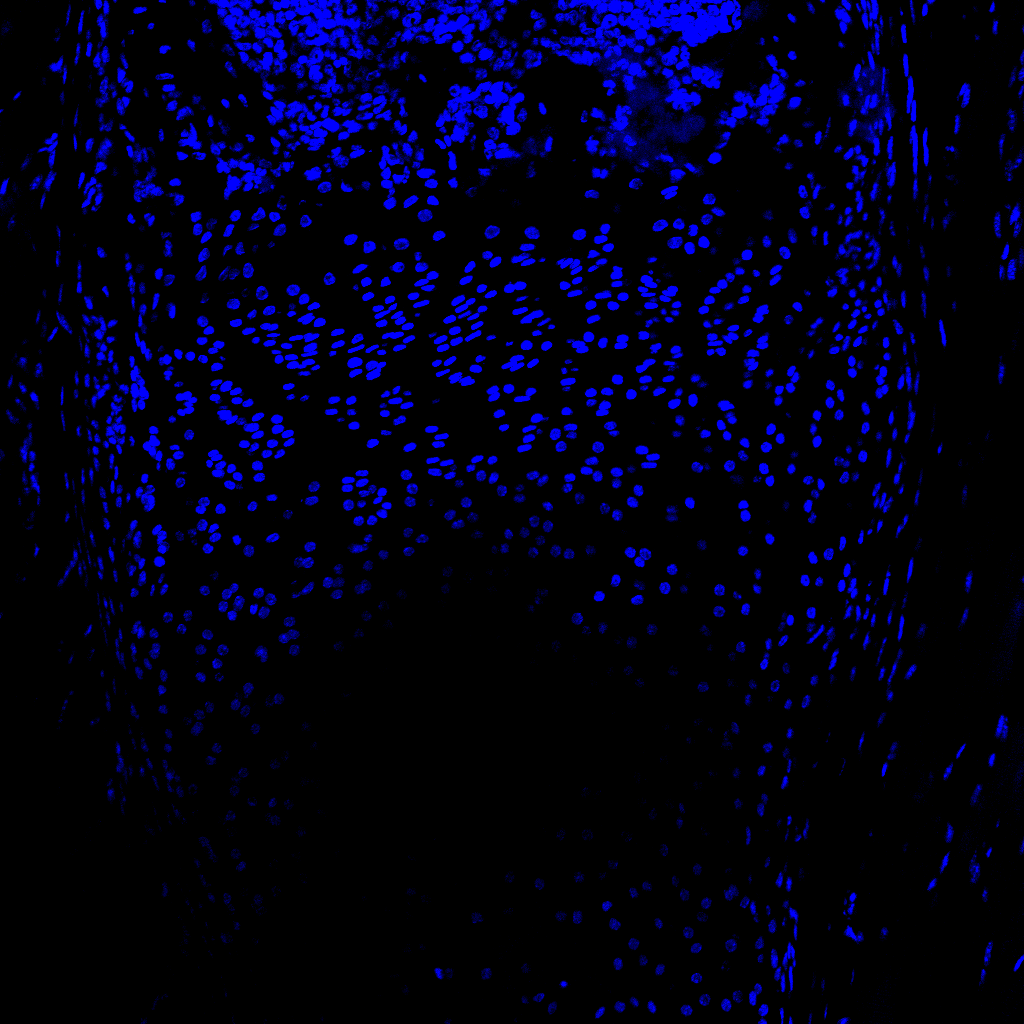

Supplement: Supplementary file 3 — Source Data Fig. 3 [file 44319_2024_93_MOESM3_ESM.zip › Figure3/3F/TM1M_1M_WT_CC_20X_dapi_blue.tif]

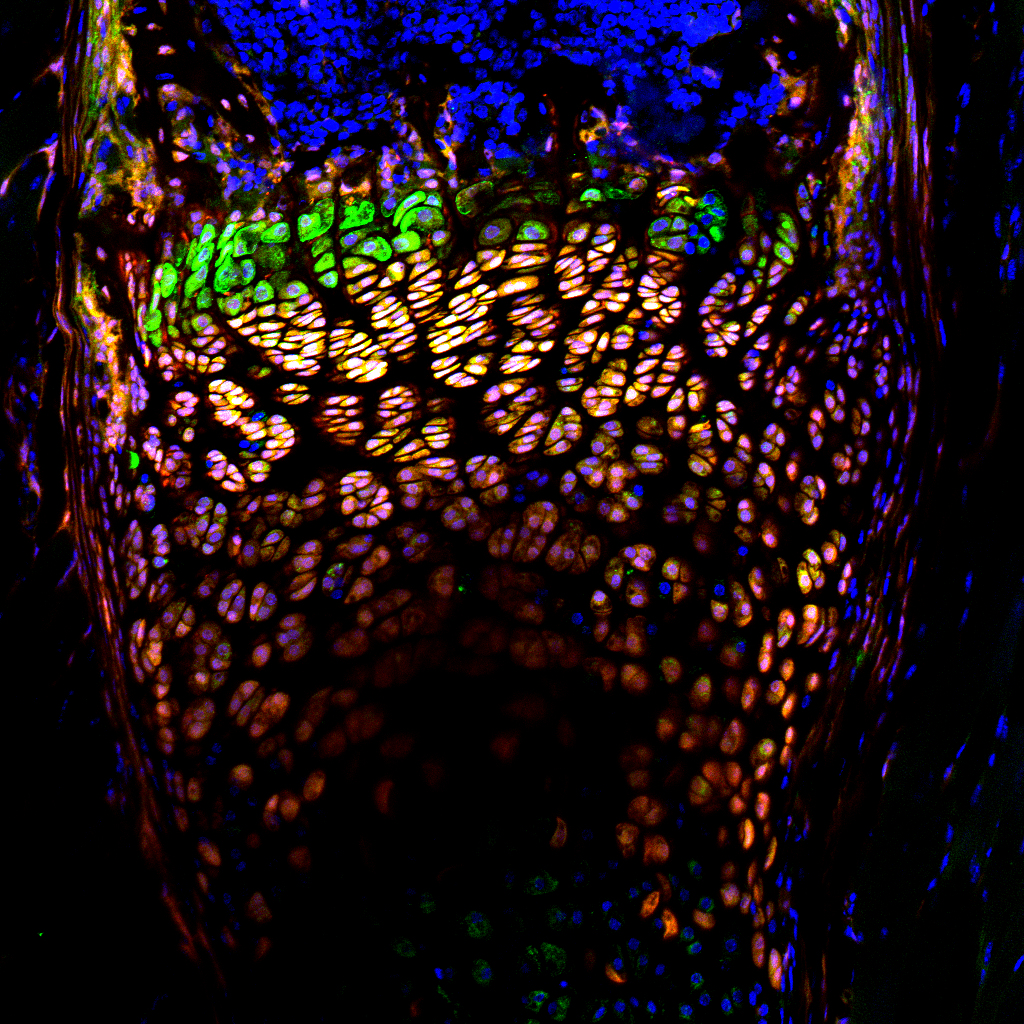

Supplement: Supplementary file 3 — Source Data Fig. 3 [file 44319_2024_93_MOESM3_ESM.zip › Figure3/3F/TM1M_1M_WT_CC_20X_merge.tif]

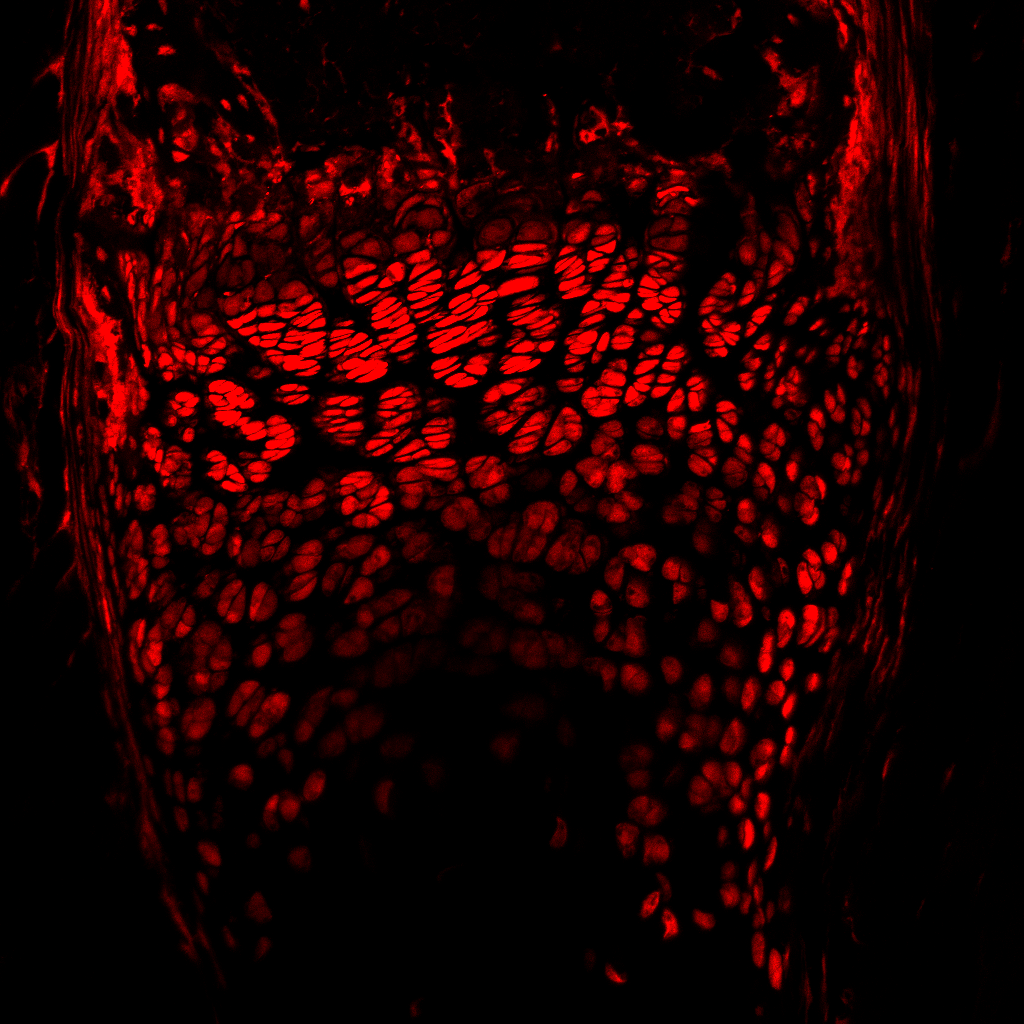

Supplement: Supplementary file 3 — Source Data Fig. 3 [file 44319_2024_93_MOESM3_ESM.zip › Figure3/3F/TM1M_1M_WT_CC_20X_td_red.tif]

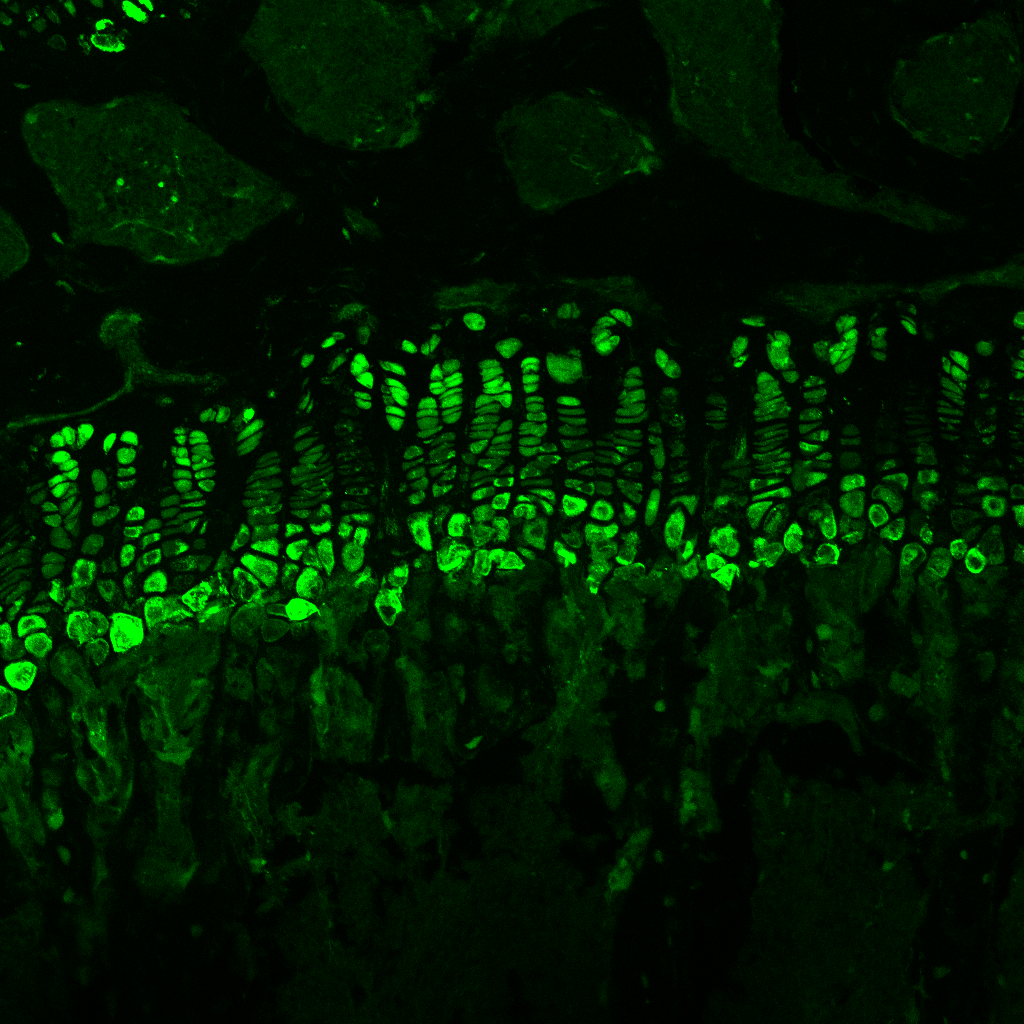

Supplement: Supplementary file 3 — Source Data Fig. 3 [file 44319_2024_93_MOESM3_ESM.zip › Figure3/3F/TM1M_1M_WT_GP_20X_acan_green.tif]

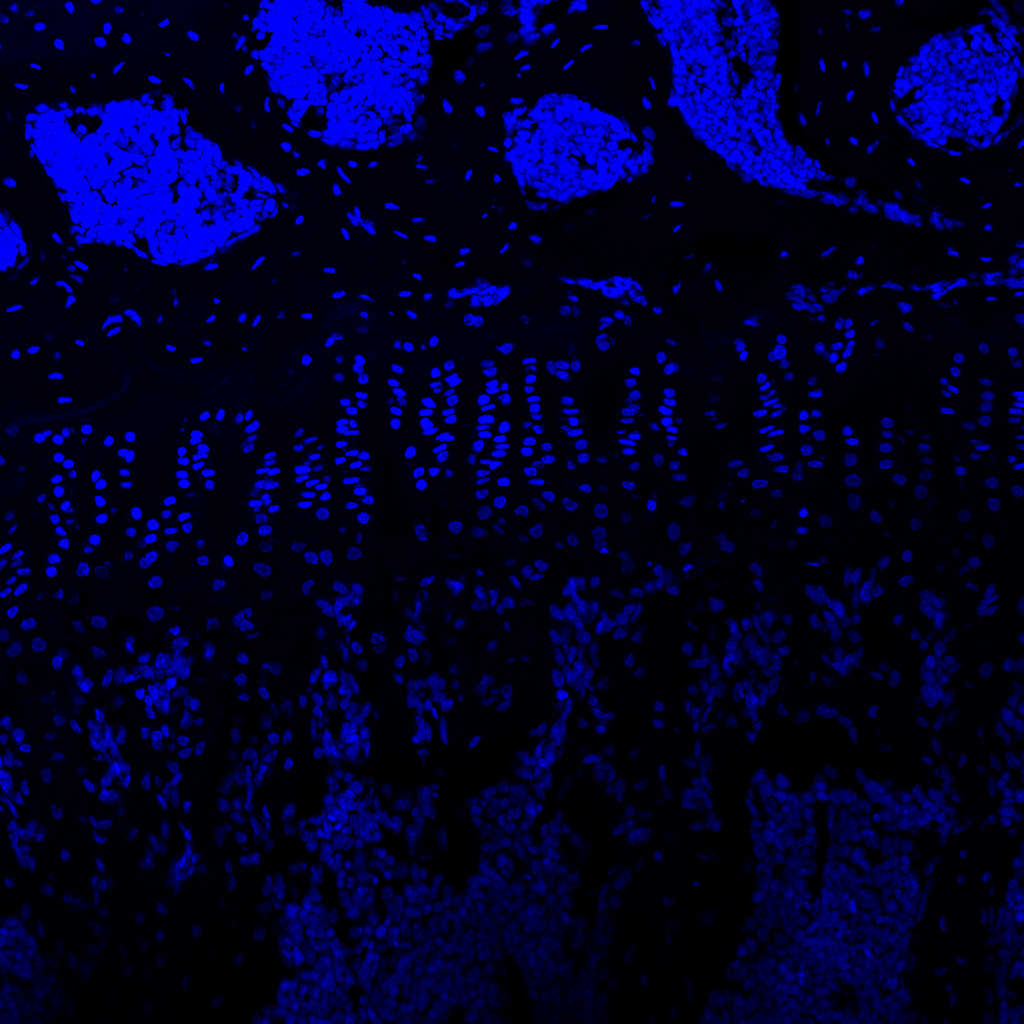

Supplement: Supplementary file 3 — Source Data Fig. 3 [file 44319_2024_93_MOESM3_ESM.zip › Figure3/3F/TM1M_1M_WT_GP_20X_dapi_blue.tif]

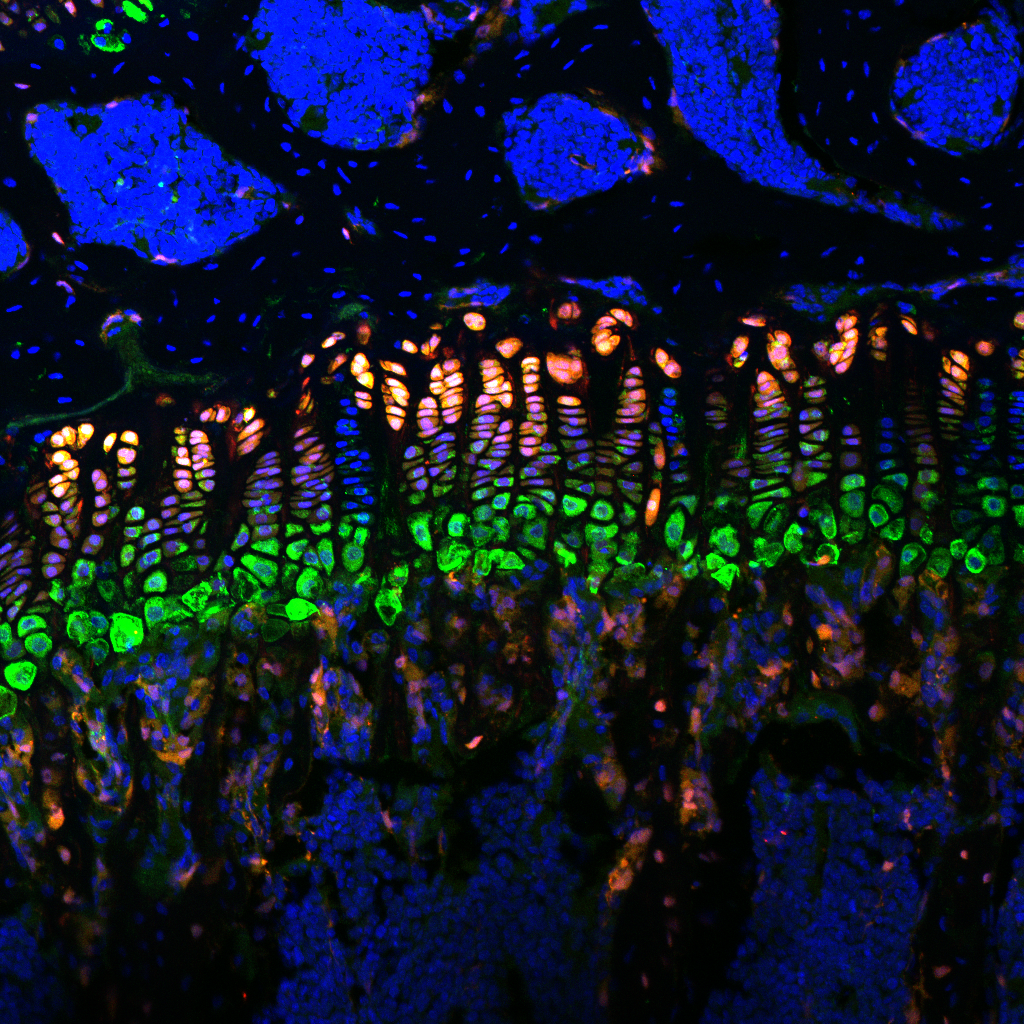

Supplement: Supplementary file 3 — Source Data Fig. 3 [file 44319_2024_93_MOESM3_ESM.zip › Figure3/3F/TM1M_1M_WT_GP_20X_merge.tif]

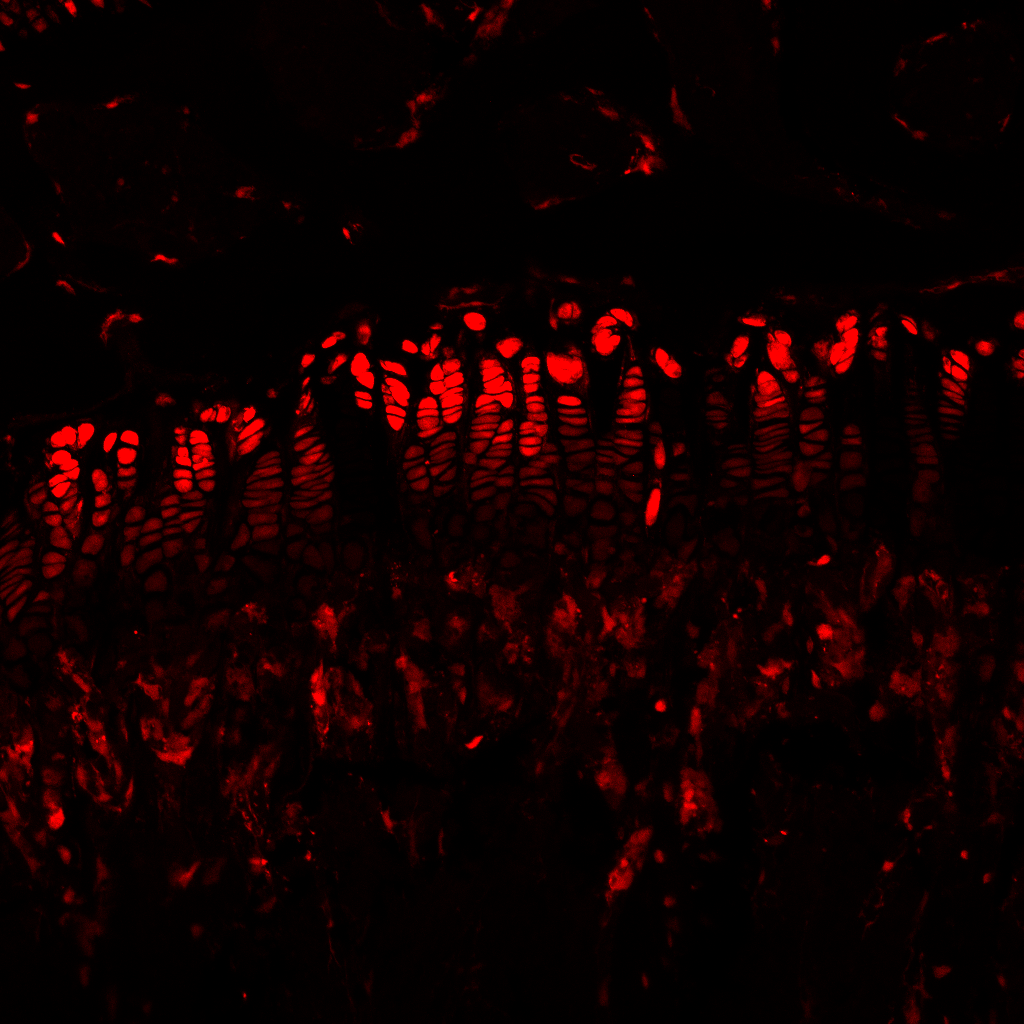

Supplement: Supplementary file 3 — Source Data Fig. 3 [file 44319_2024_93_MOESM3_ESM.zip › Figure3/3F/TM1M_1M_WT_GP_20X_td_red.tif]

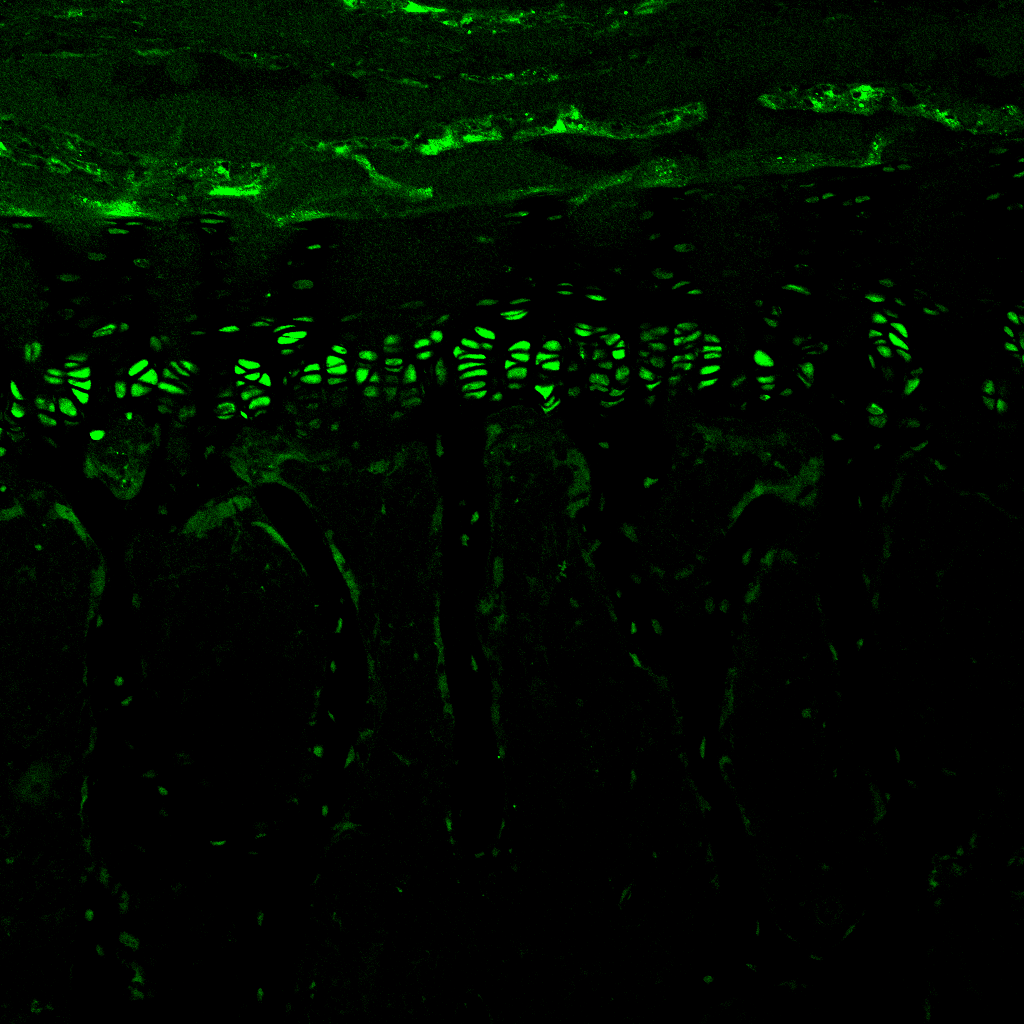

Supplement: Supplementary file 3 — Source Data Fig. 3 [file 44319_2024_93_MOESM3_ESM.zip › Figure3/3F/TM1M_1M_WT_SP_20X_acan_green.tif]

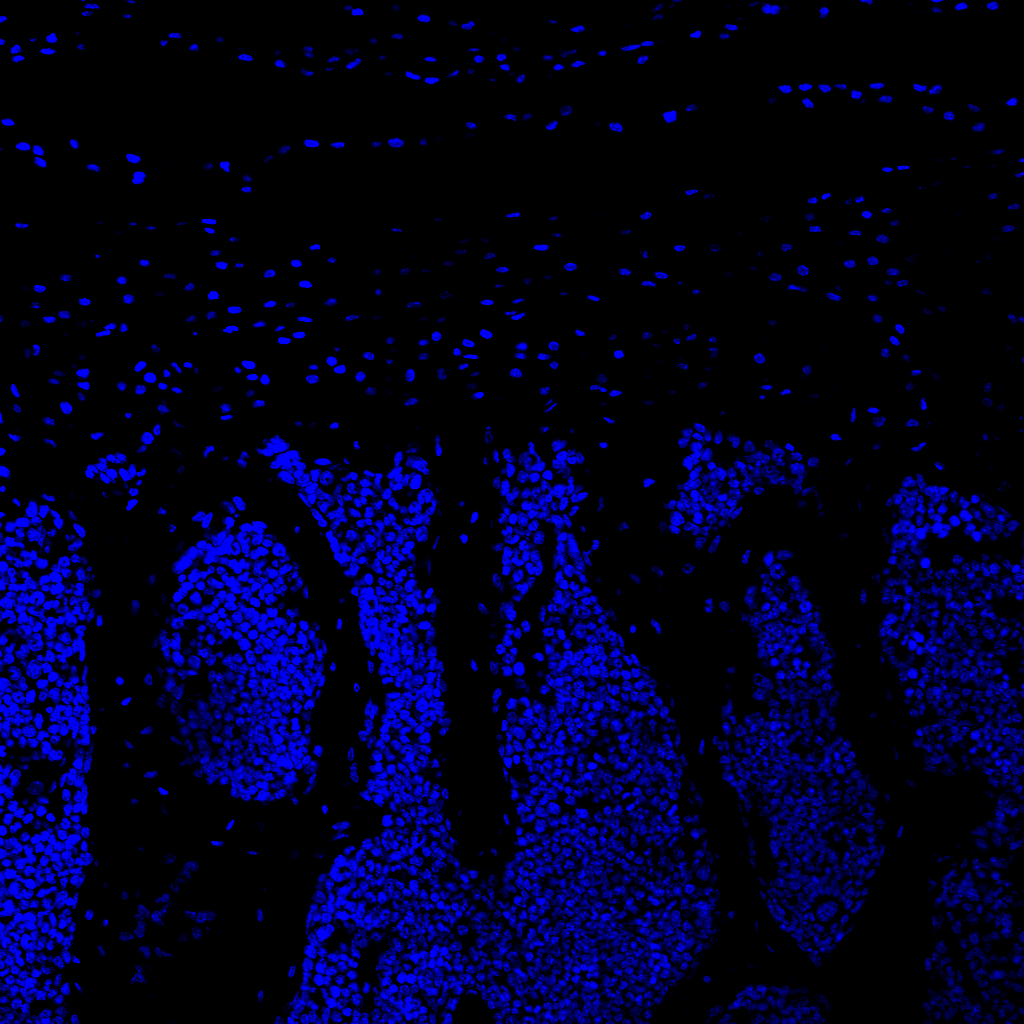

Supplement: Supplementary file 3 — Source Data Fig. 3 [file 44319_2024_93_MOESM3_ESM.zip › Figure3/3F/TM1M_1M_WT_SP_20X_dapi_blue.tif]

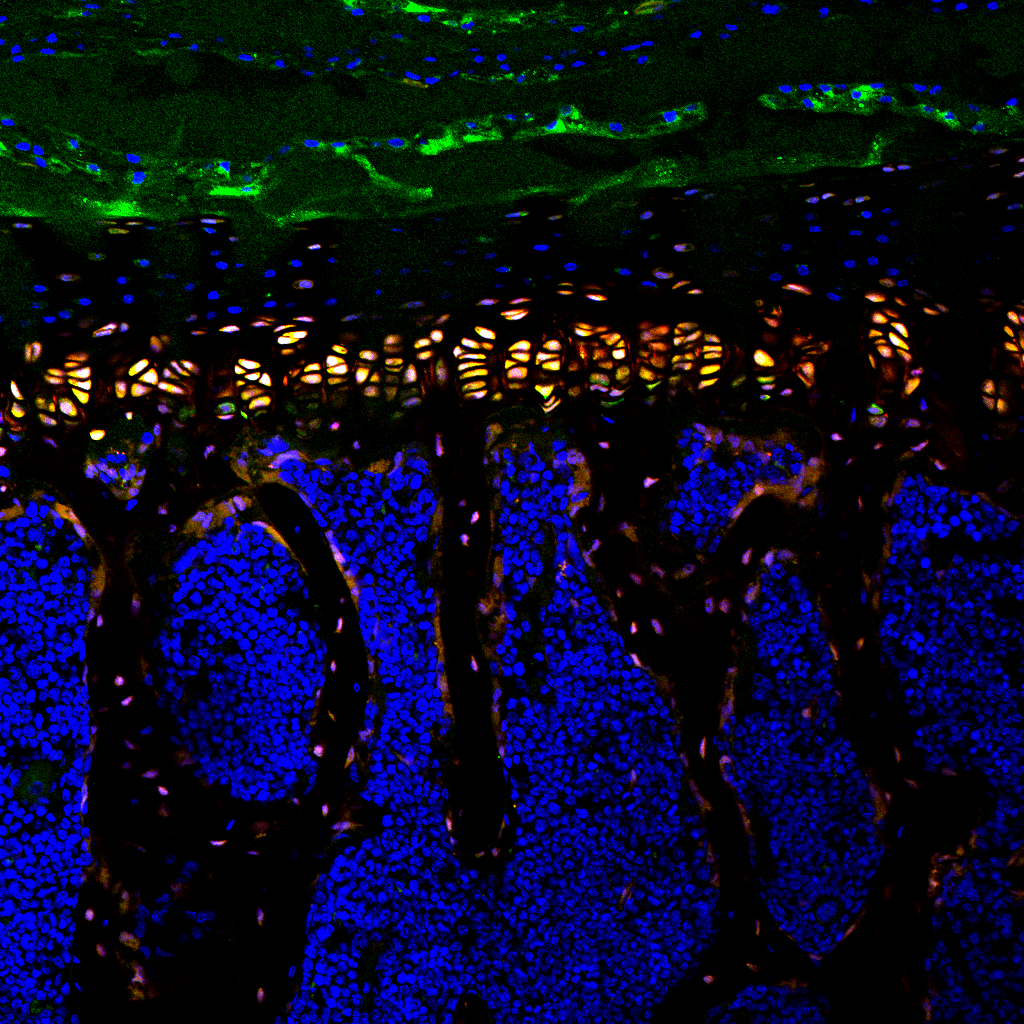

Supplement: Supplementary file 3 — Source Data Fig. 3 [file 44319_2024_93_MOESM3_ESM.zip › Figure3/3F/TM1M_1M_WT_SP_20X_merge.tif]

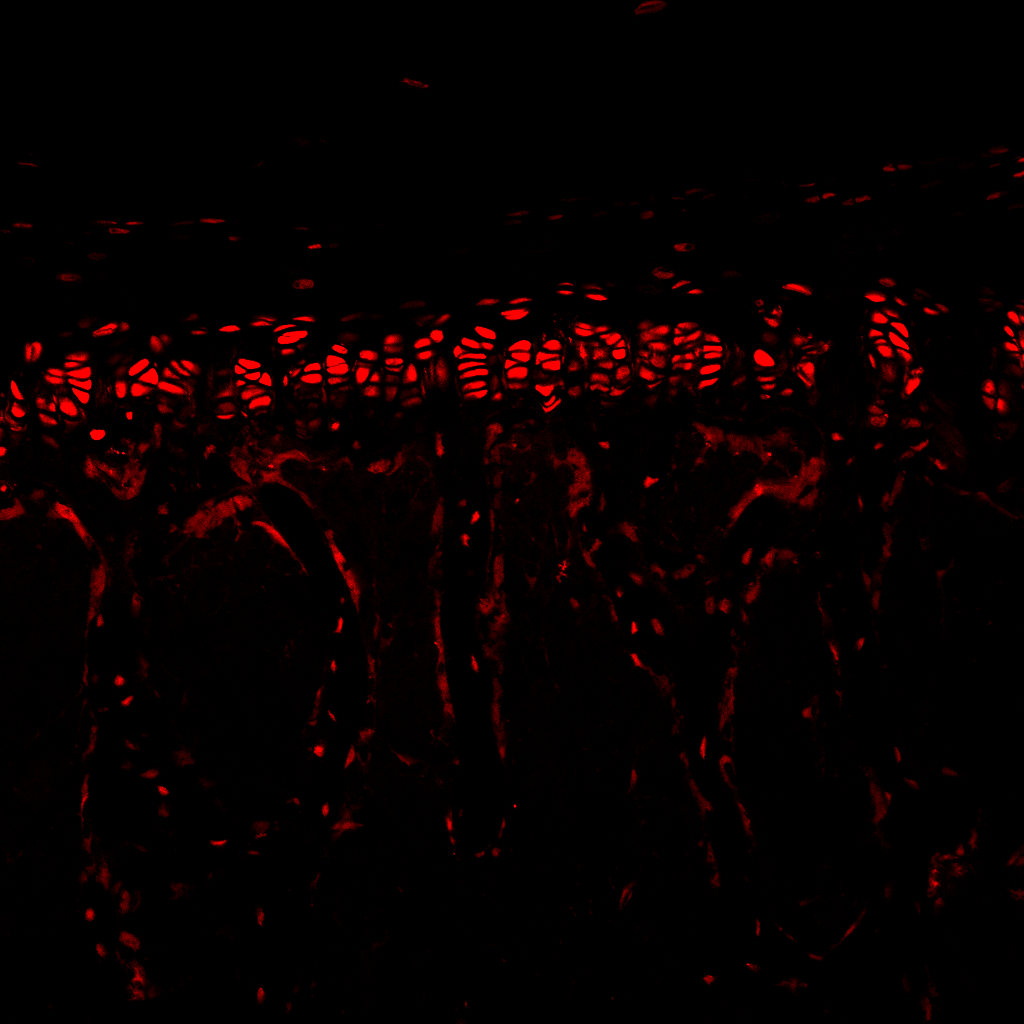

Supplement: Supplementary file 3 — Source Data Fig. 3 [file 44319_2024_93_MOESM3_ESM.zip › Figure3/3F/TM1M_1M_WT_SP_20X_td_red.tif]

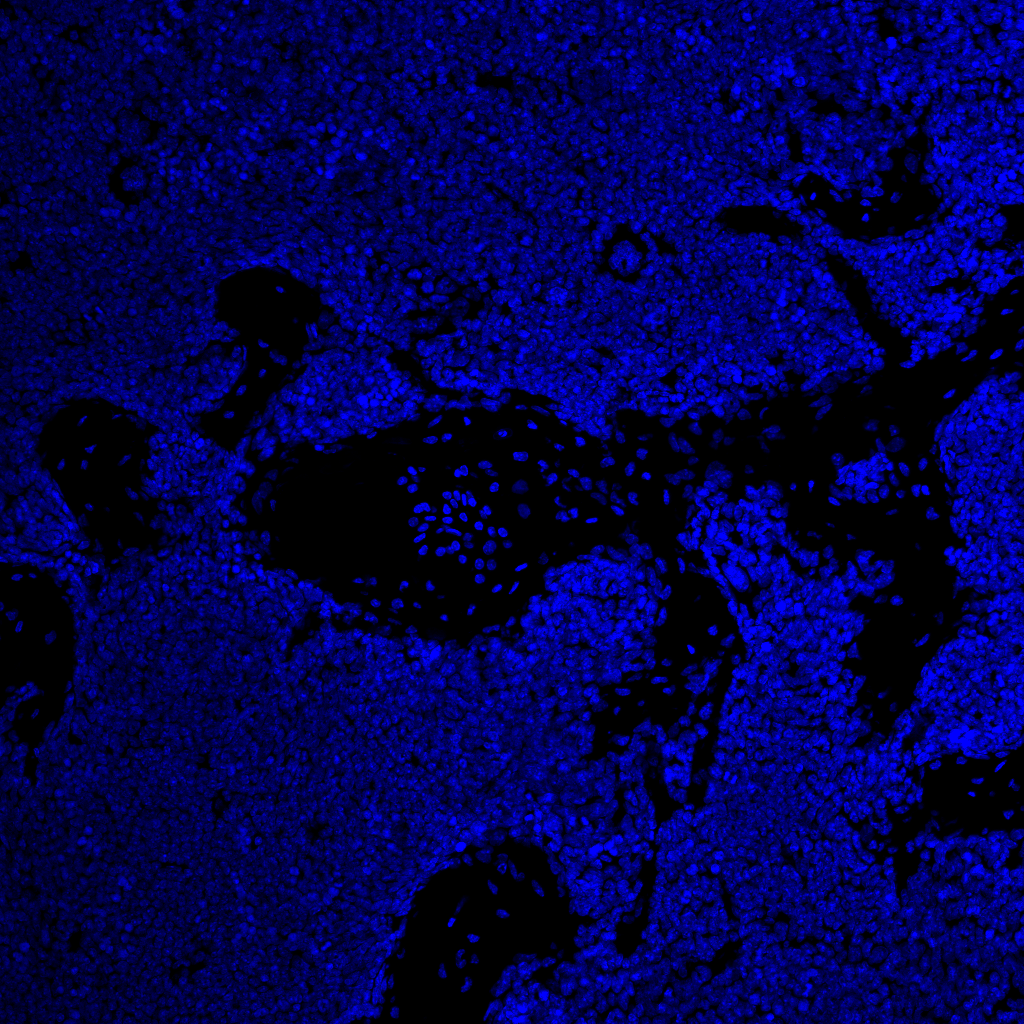

Supplement: Supplementary file 3 — Source Data Fig. 3 [file 44319_2024_93_MOESM3_ESM.zip › Figure3/3H/TM1M_1M_DTA GP 20X_dapi_blue.tif]

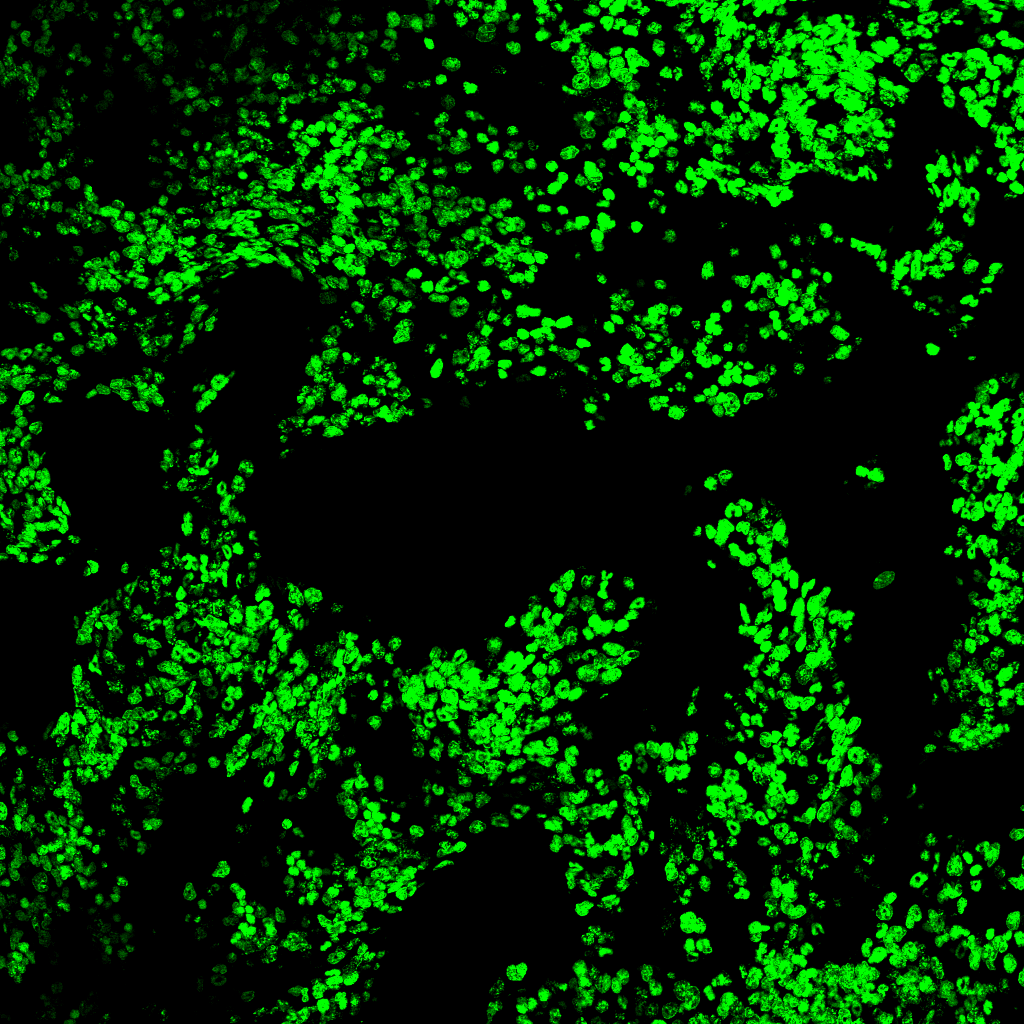

Supplement: Supplementary file 3 — Source Data Fig. 3 [file 44319_2024_93_MOESM3_ESM.zip › Figure3/3H/TM1M_1M_DTA GP 20X_edu_green.tif]

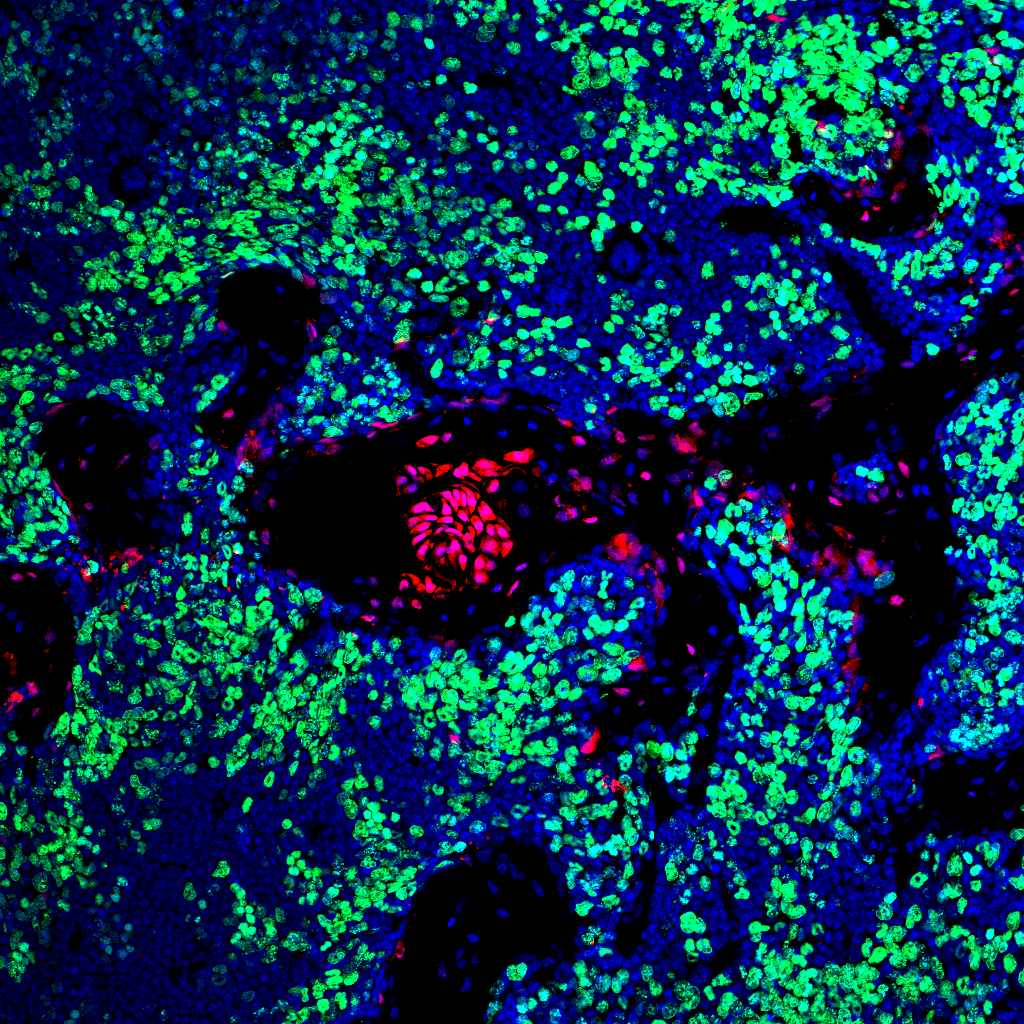

Supplement: Supplementary file 3 — Source Data Fig. 3 [file 44319_2024_93_MOESM3_ESM.zip › Figure3/3H/TM1M_1M_DTA GP 20X_merge.tif]

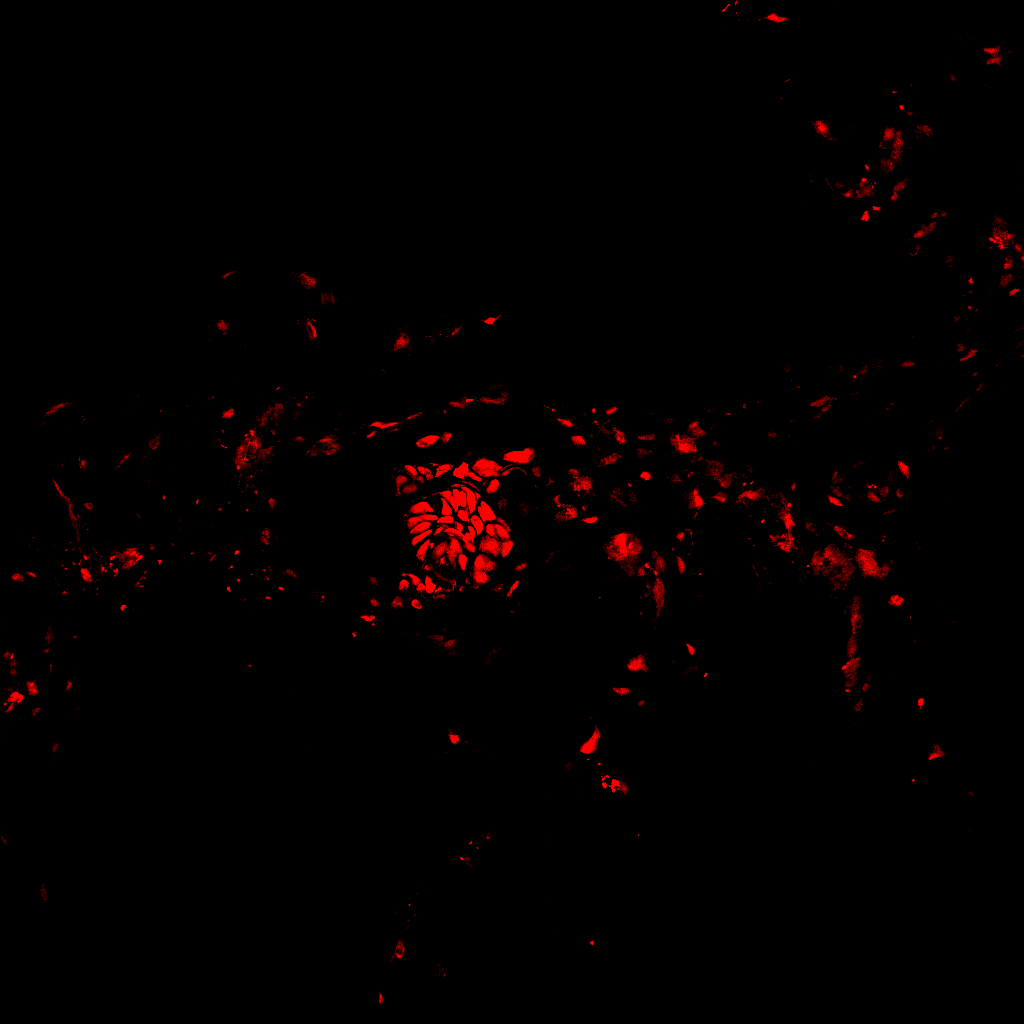

Supplement: Supplementary file 3 — Source Data Fig. 3 [file 44319_2024_93_MOESM3_ESM.zip › Figure3/3H/TM1M_1M_DTA GP 20X_td_red.tif]

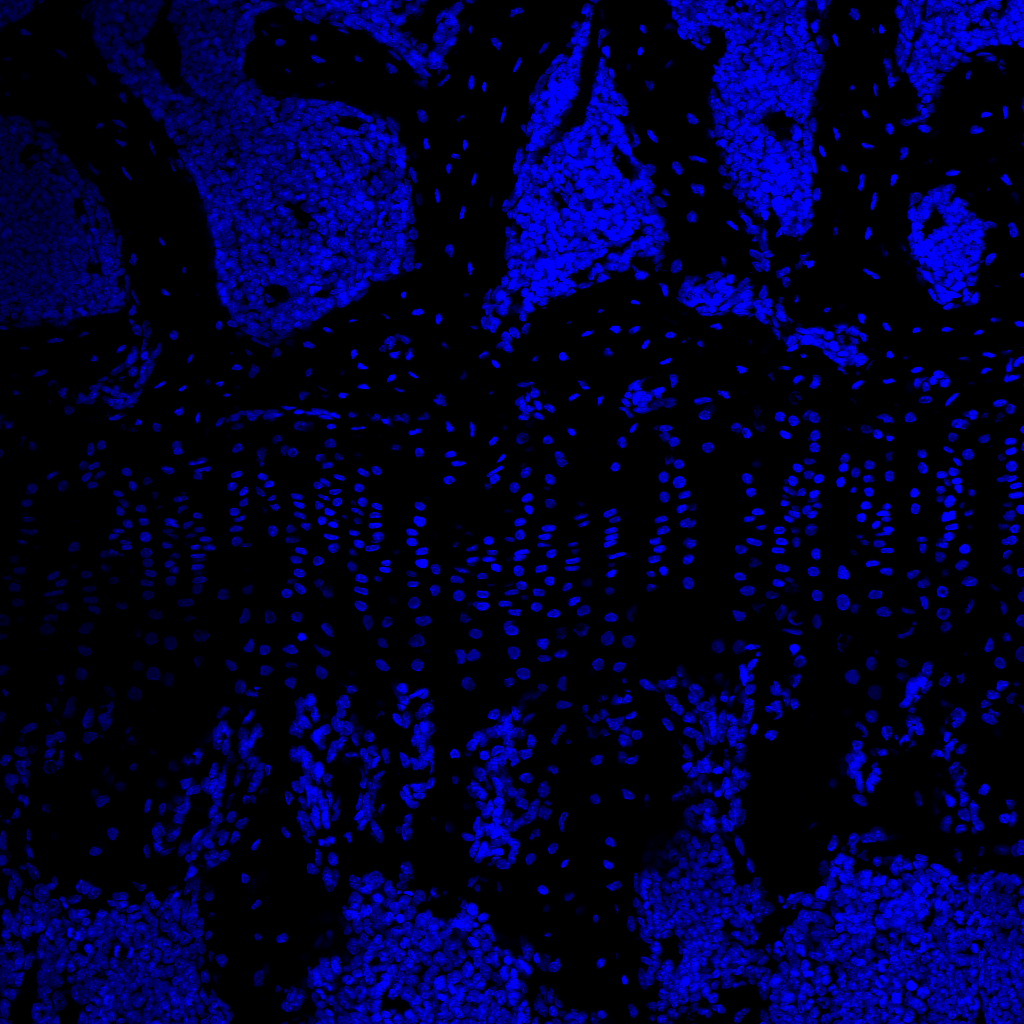

Supplement: Supplementary file 3 — Source Data Fig. 3 [file 44319_2024_93_MOESM3_ESM.zip › Figure3/3H/TM1M_1M_WT GP 20X_dapi_blue.tif]

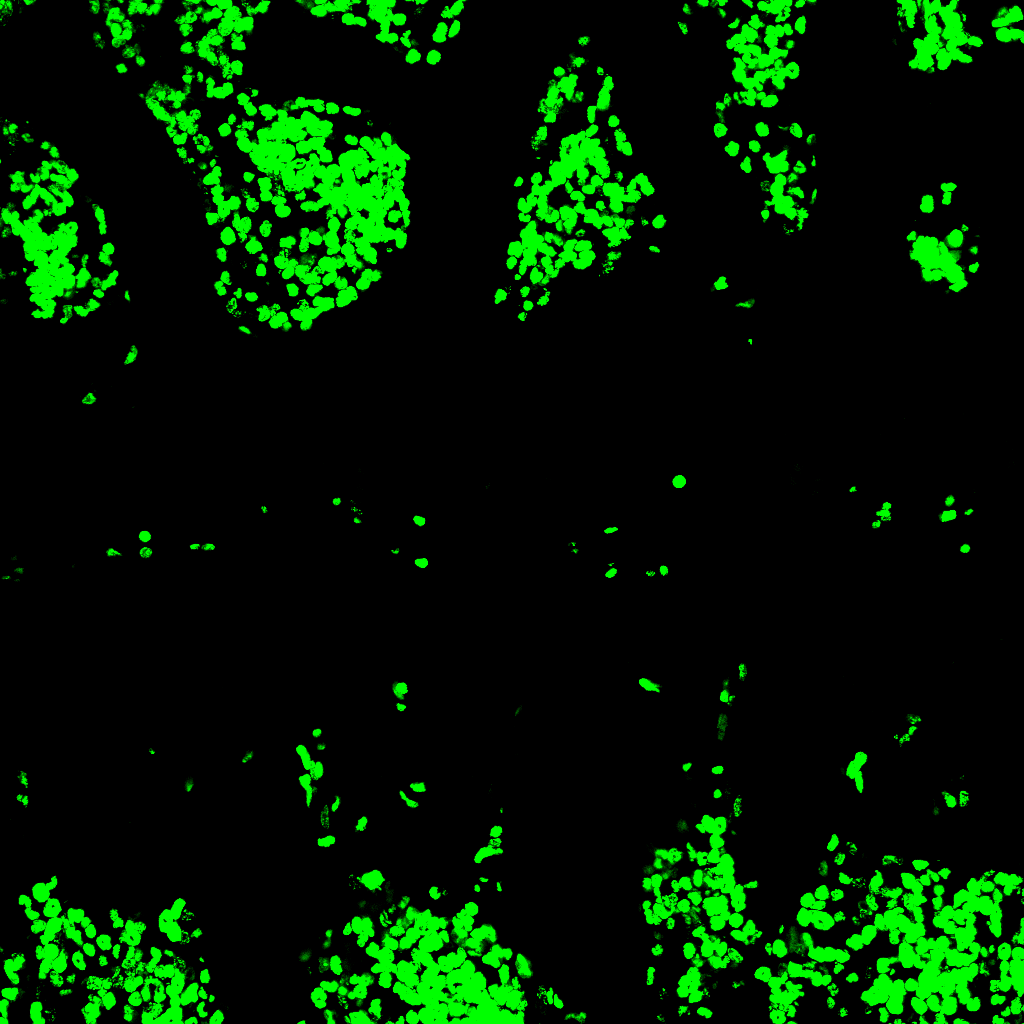

Supplement: Supplementary file 3 — Source Data Fig. 3 [file 44319_2024_93_MOESM3_ESM.zip › Figure3/3H/TM1M_1M_WT GP 20X_edu_green.tif]

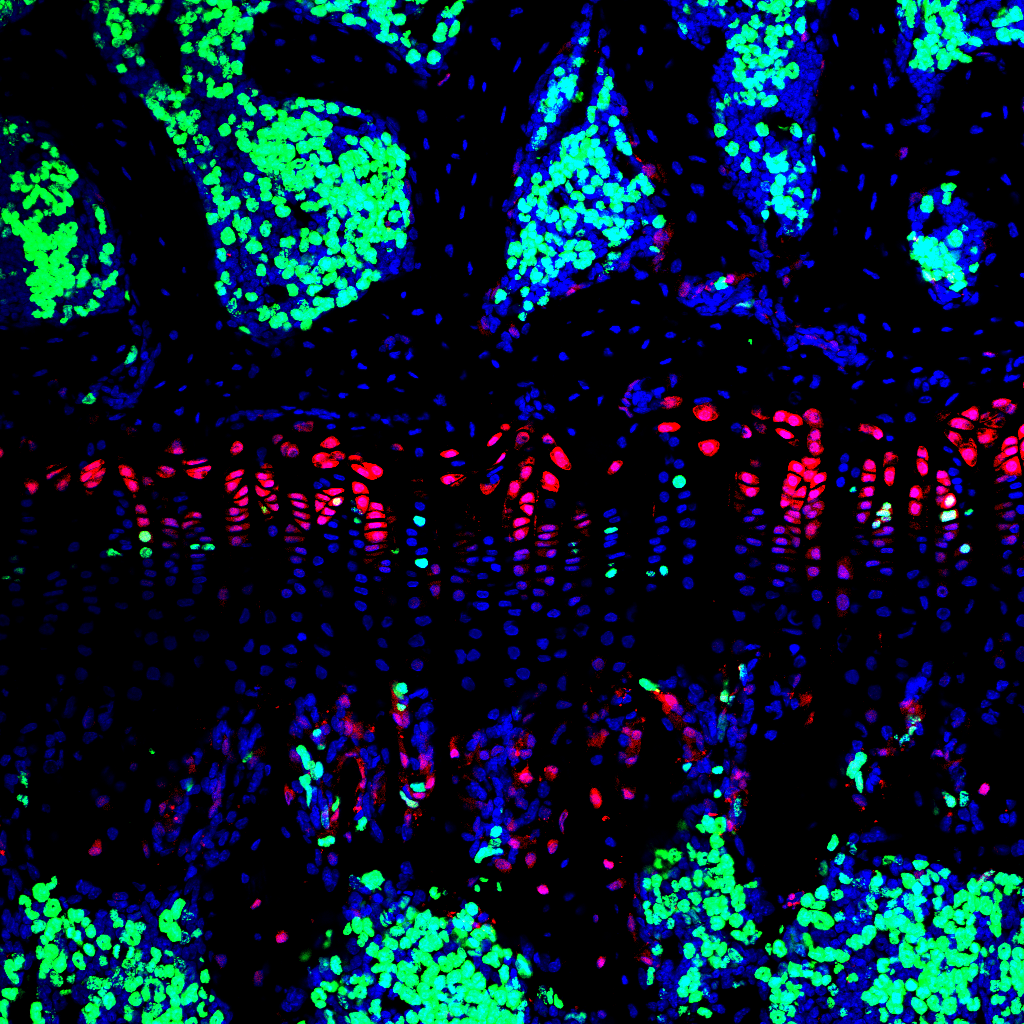

Supplement: Supplementary file 3 — Source Data Fig. 3 [file 44319_2024_93_MOESM3_ESM.zip › Figure3/3H/TM1M_1M_WT GP 20X_merge.tif]
