# Supplementary figures and images for: Gli1 labels progenitors during chondrogenesis in postnatal mice (part 2 of 3)
Source: EMBO Rep. 2024 Feb 26;25(4):12. doi: 10.1038/s44319-024-00093-x (PMC11014955; doi:10.1038/s44319-024-00093-x)

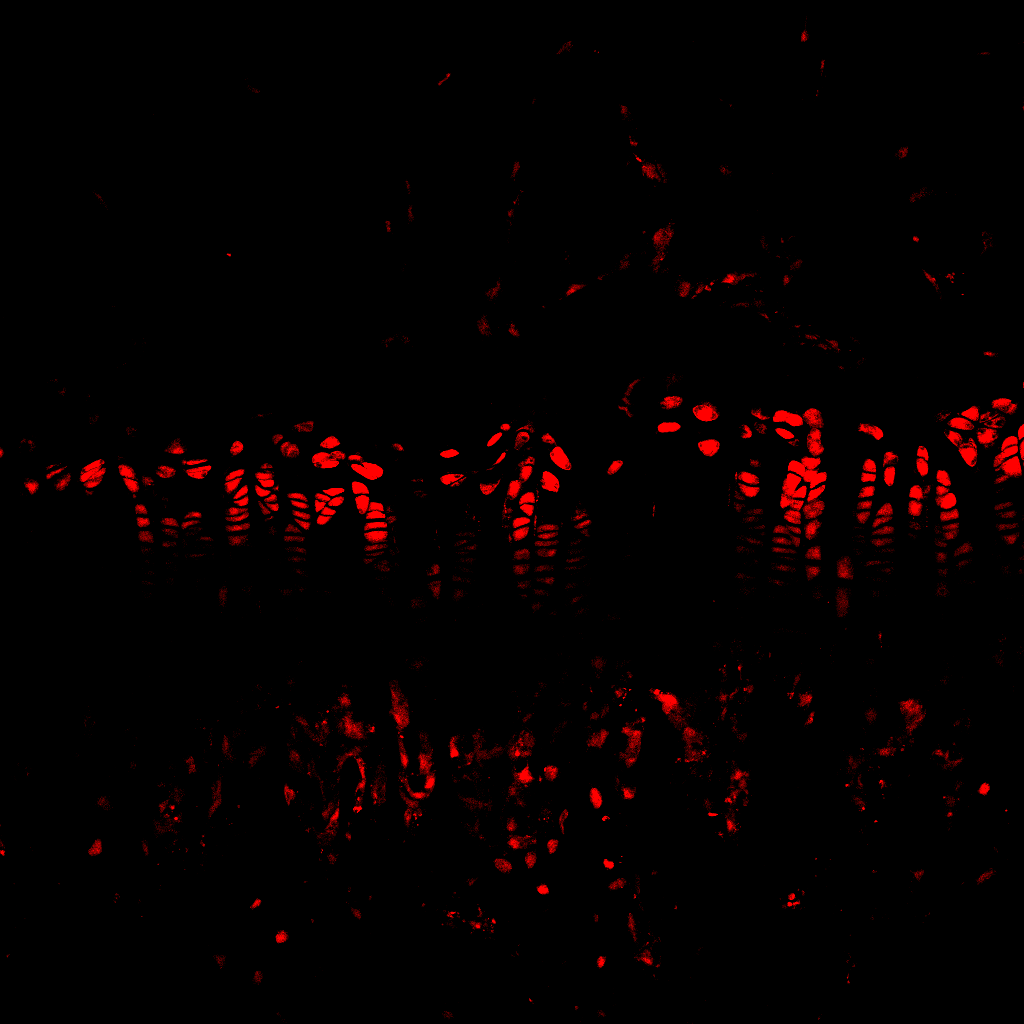

Supplement: Supplementary file 3 — Source Data Fig. 3 [file 44319_2024_93_MOESM3_ESM.zip › Figure3/3H/TM1M_1M_WT GP 20X_td_red.tif]

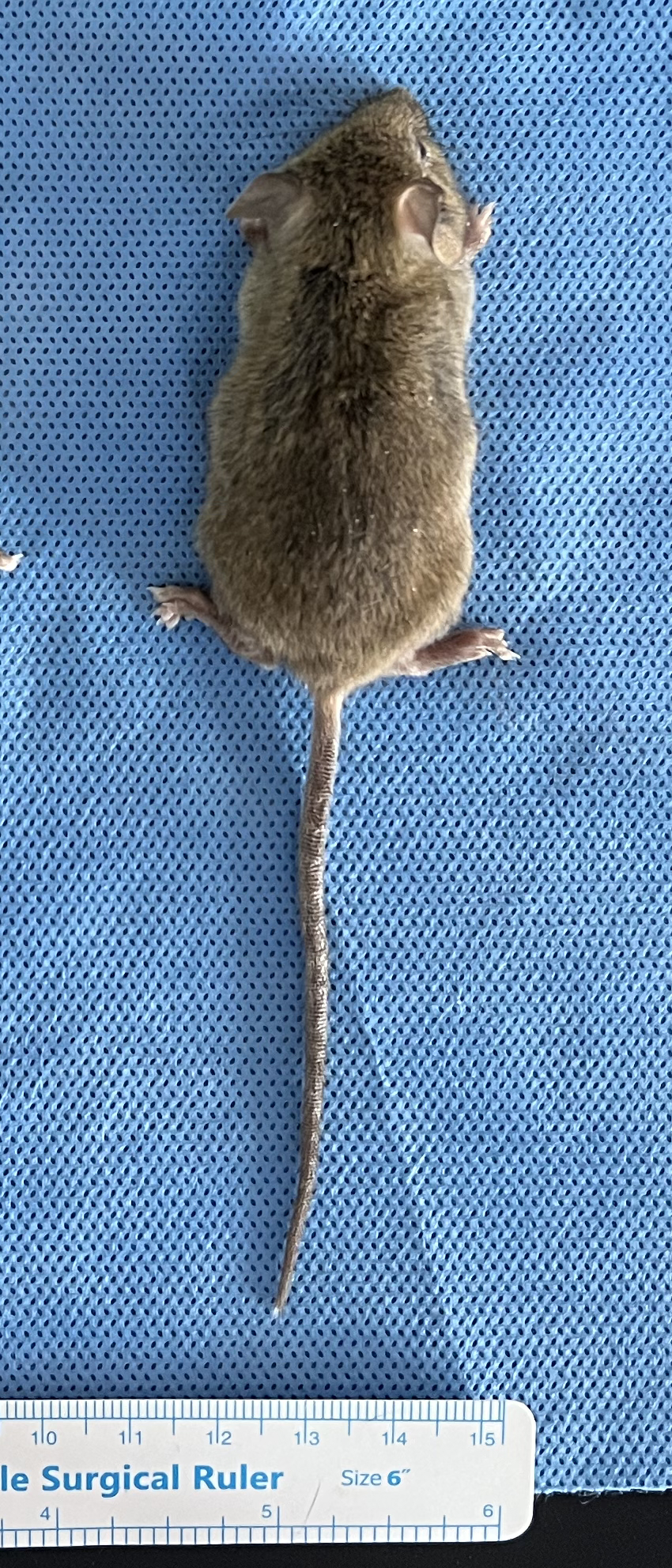

Supplement: Supplementary file 4 — Source Data Fig. 4 [file 44319_2024_93_MOESM4_ESM.zip › Figure4/4A/GDC.tif]

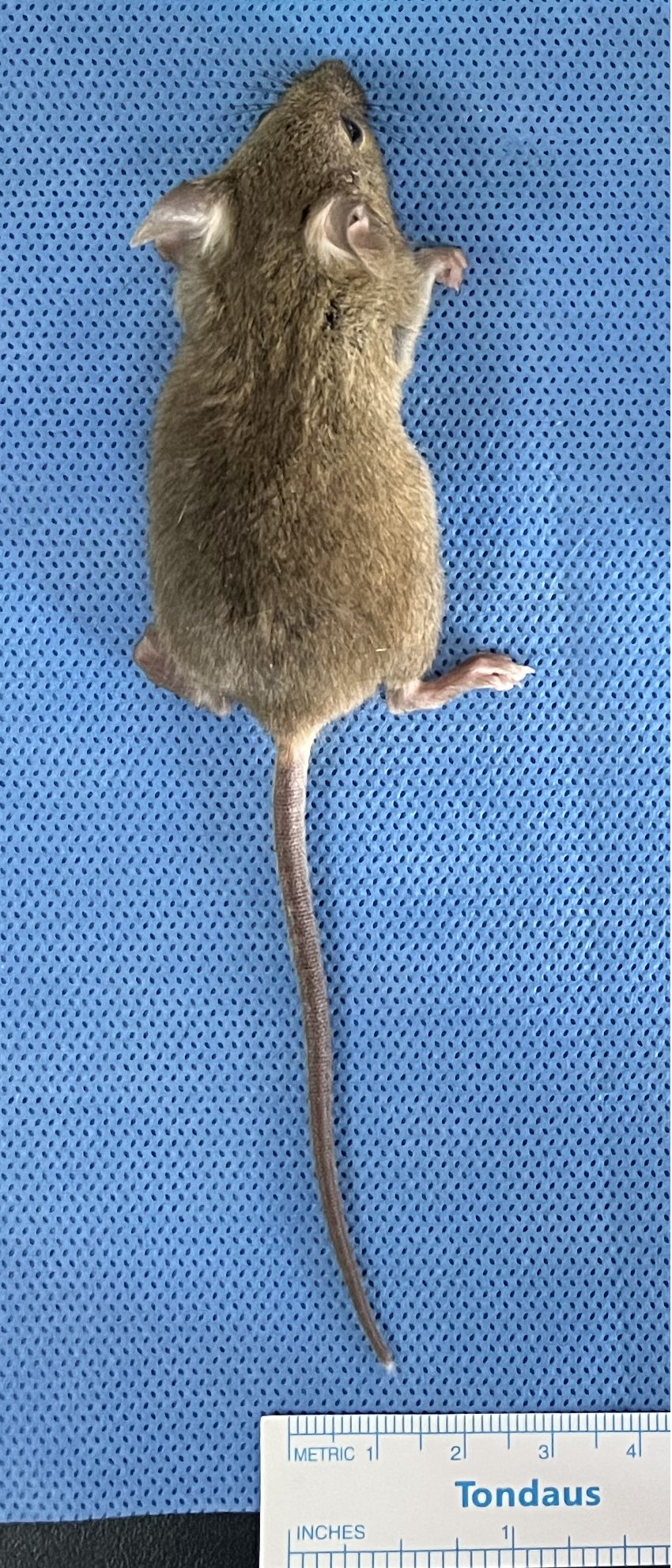

Supplement: Supplementary file 4 — Source Data Fig. 4 [file 44319_2024_93_MOESM4_ESM.zip › Figure4/4A/Veh.tif]

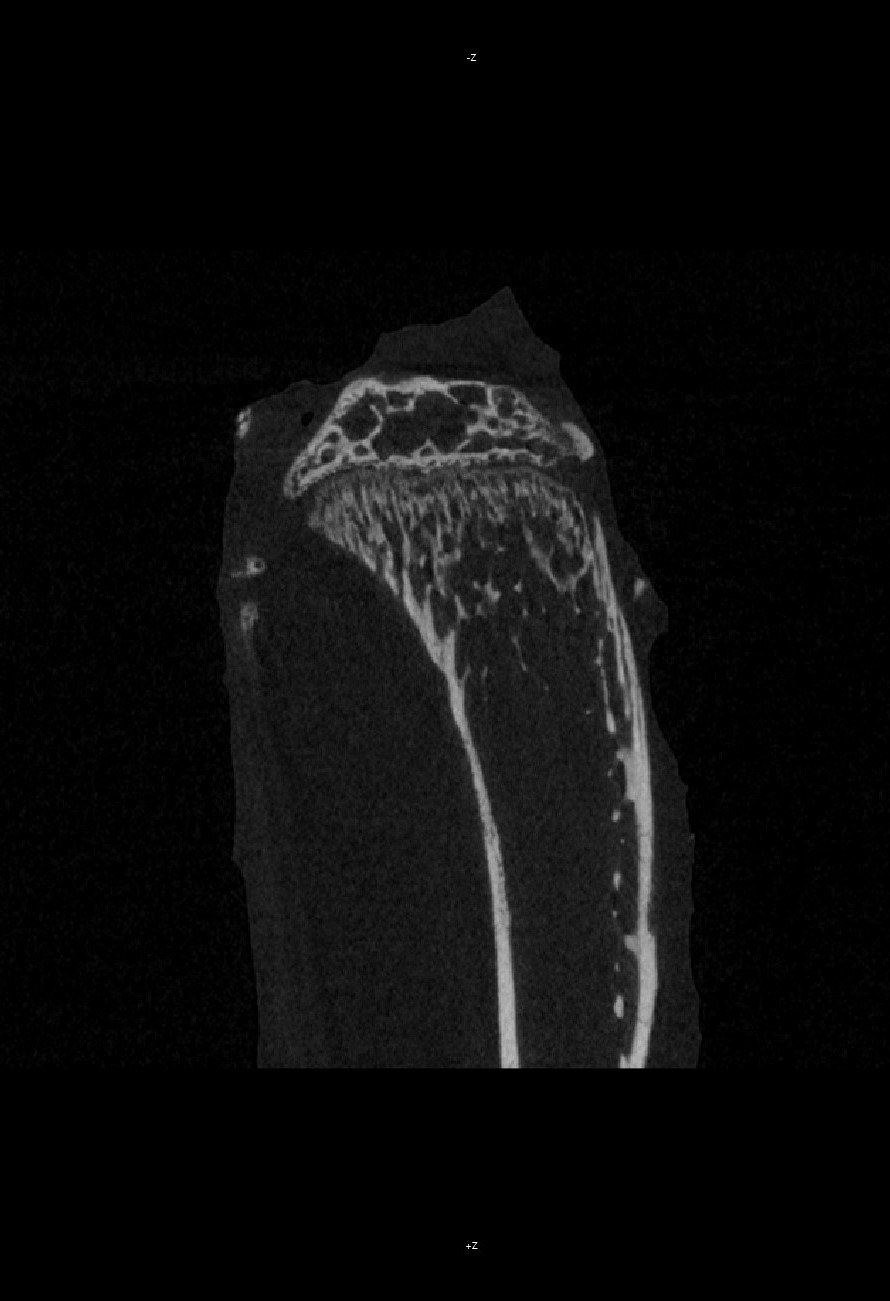

Supplement: Supplementary file 4 — Source Data Fig. 4 [file 44319_2024_93_MOESM4_ESM.zip › Figure4/4B/GDC.jpg]

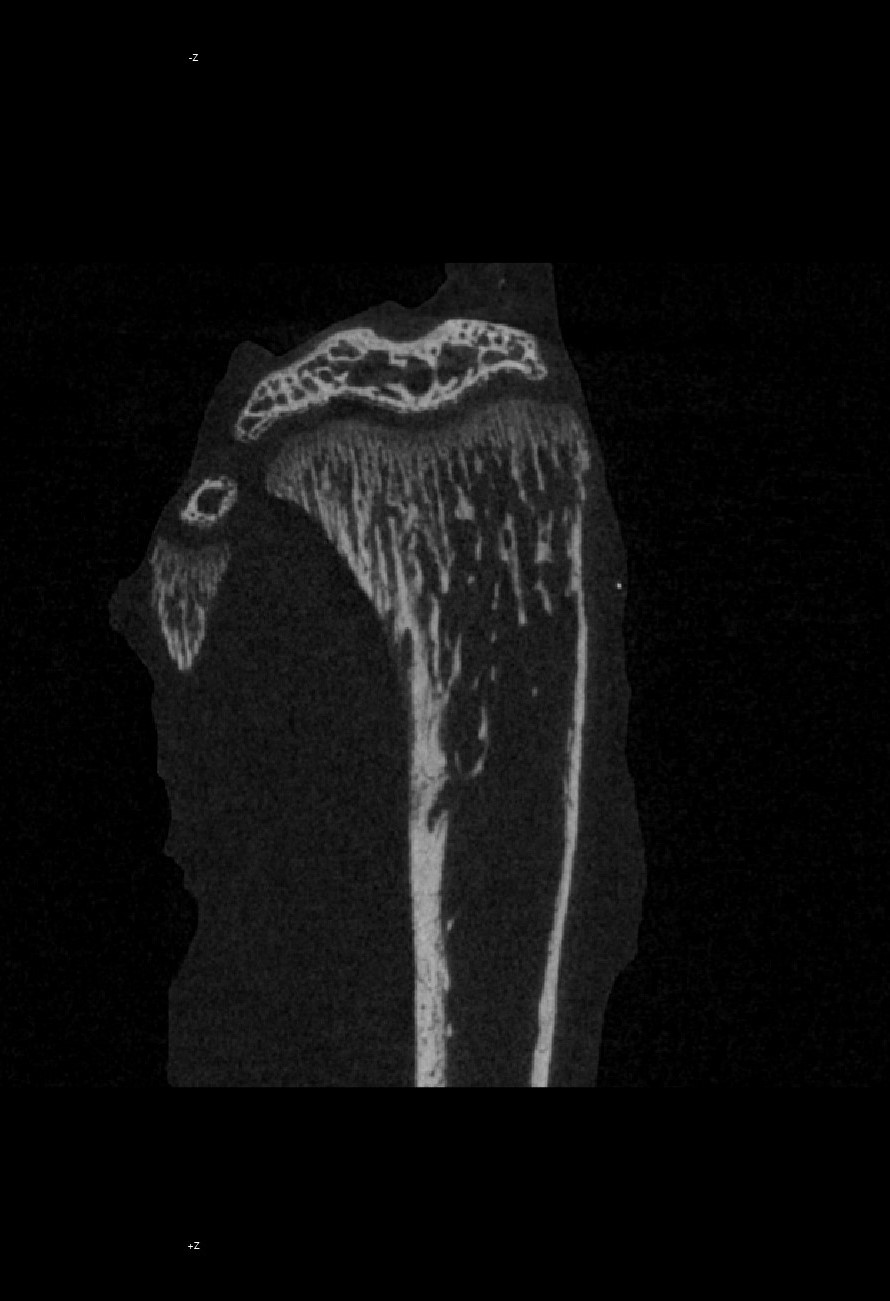

Supplement: Supplementary file 4 — Source Data Fig. 4 [file 44319_2024_93_MOESM4_ESM.zip › Figure4/4B/Veh.jpg]

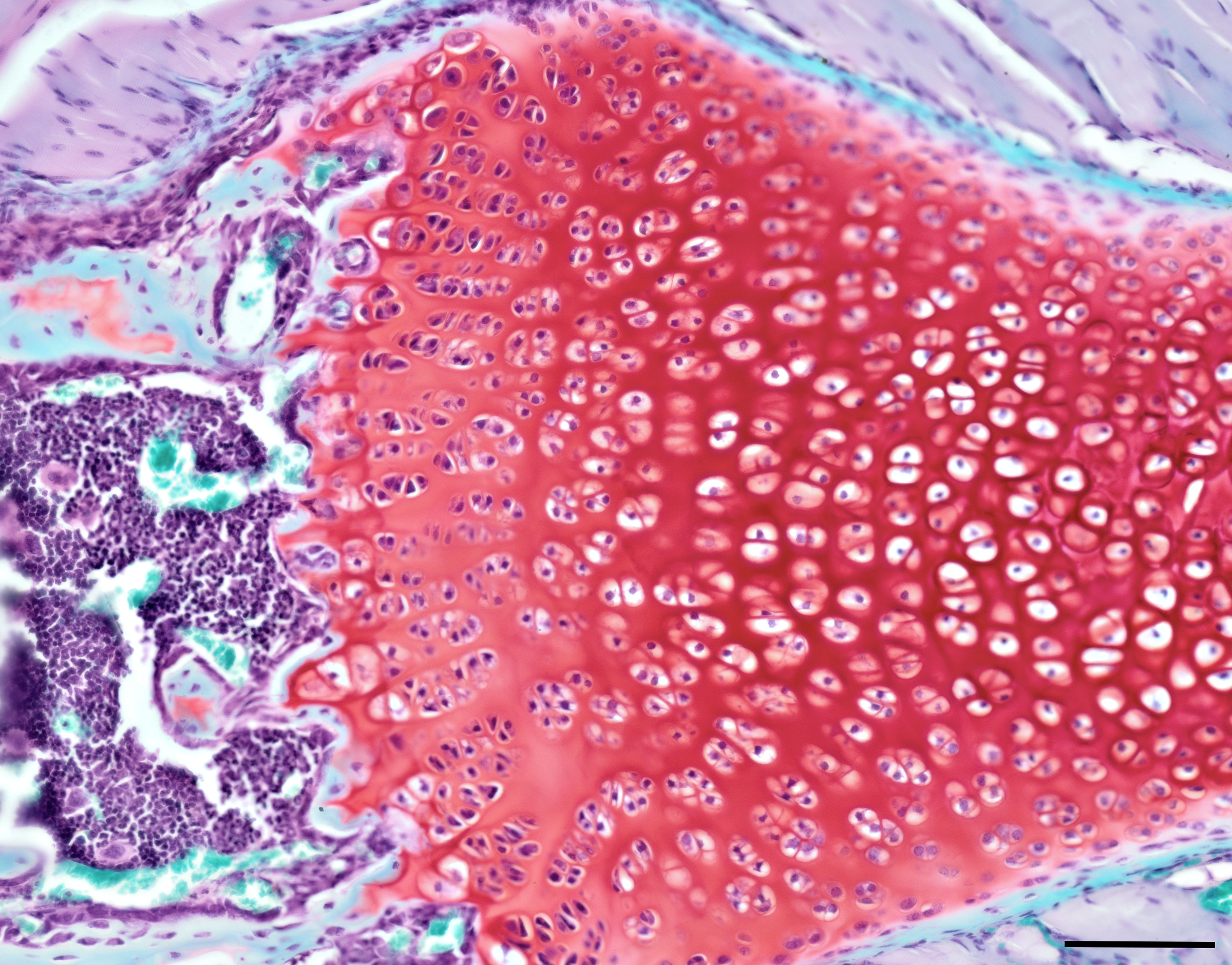

Supplement: Supplementary file 4 — Source Data Fig. 4 [file 44319_2024_93_MOESM4_ESM.zip › Figure4/4C/GDC_CC.tif]

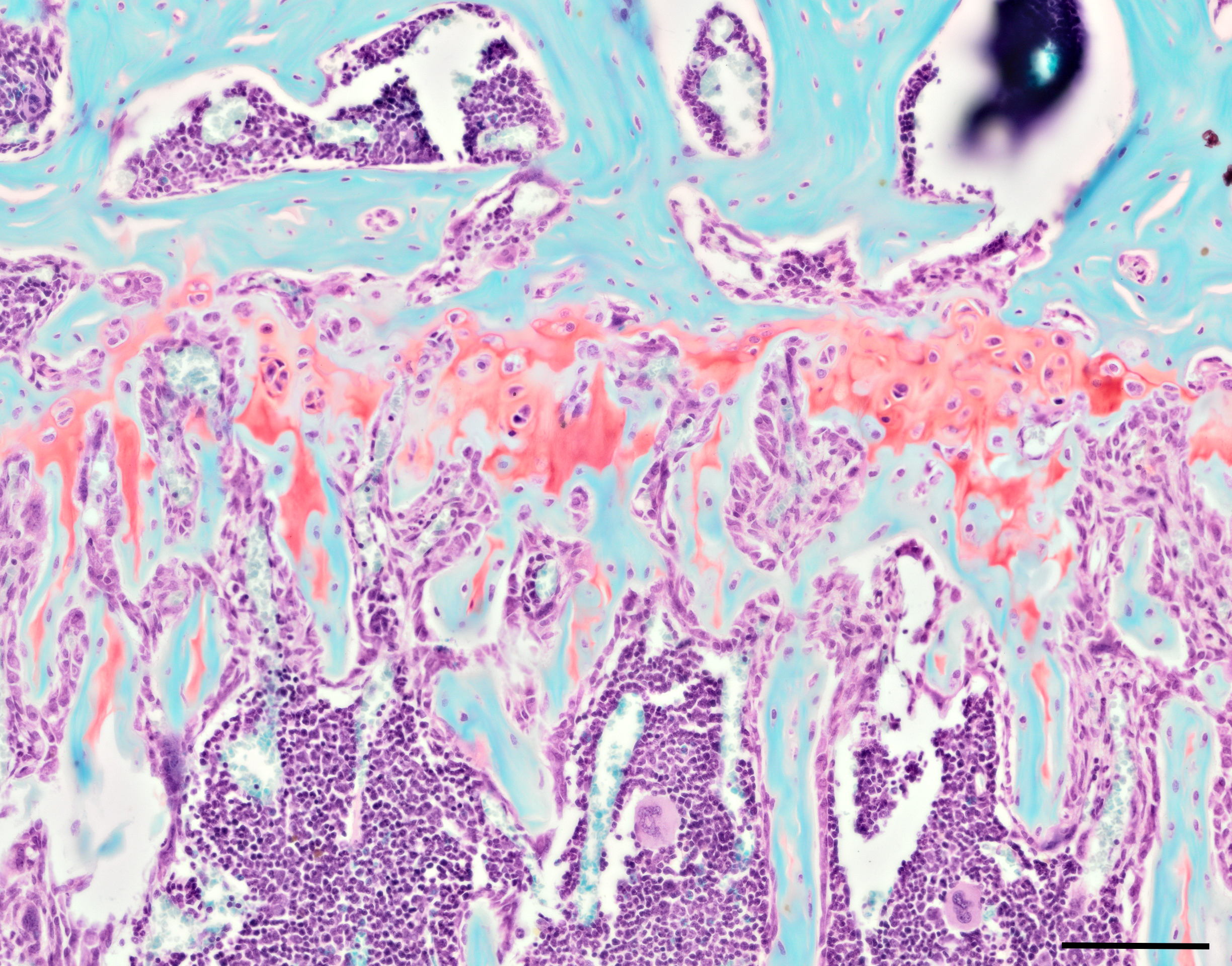

Supplement: Supplementary file 4 — Source Data Fig. 4 [file 44319_2024_93_MOESM4_ESM.zip › Figure4/4C/GDC_GP.tif]

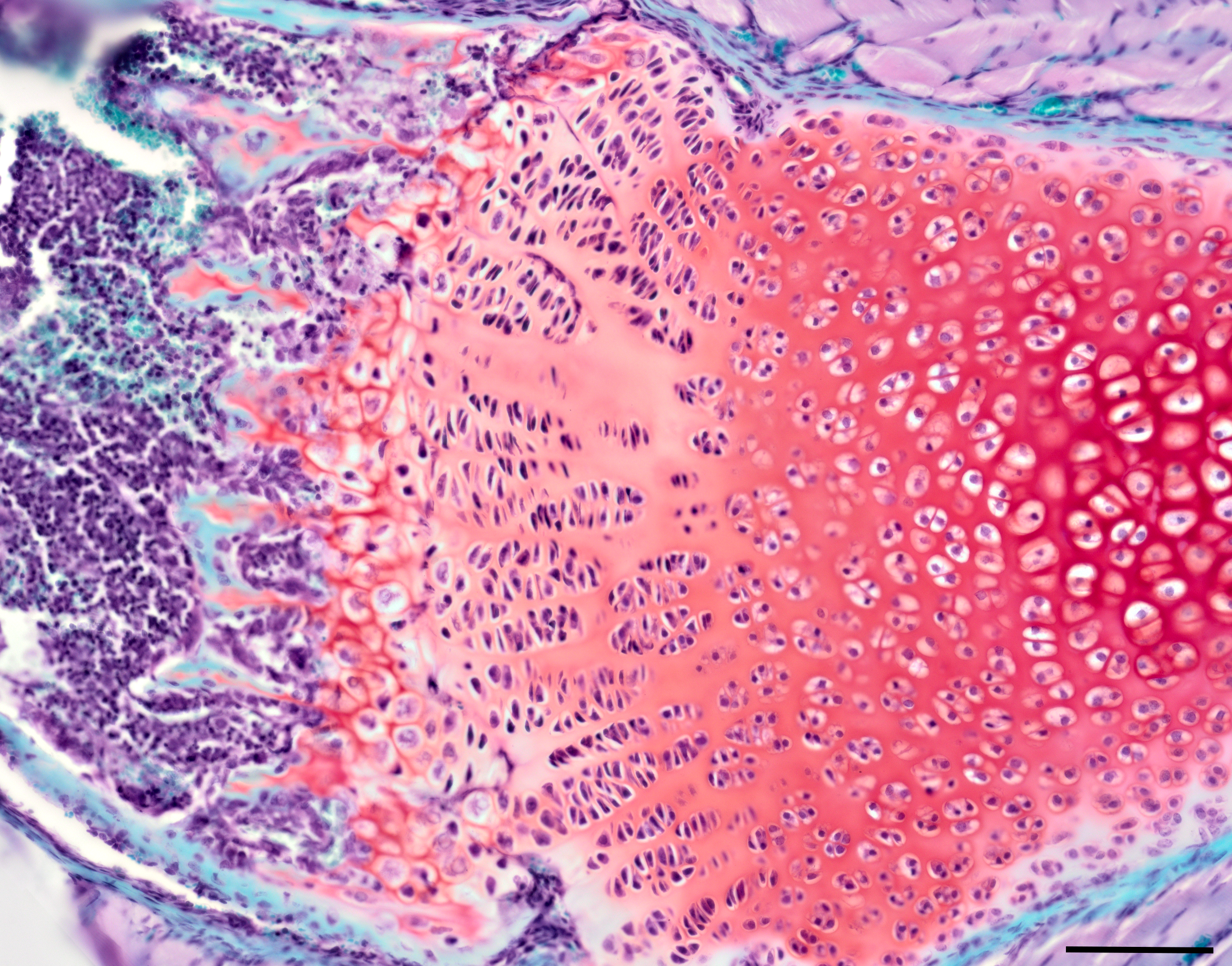

Supplement: Supplementary file 4 — Source Data Fig. 4 [file 44319_2024_93_MOESM4_ESM.zip › Figure4/4C/Veh_CC.tif]

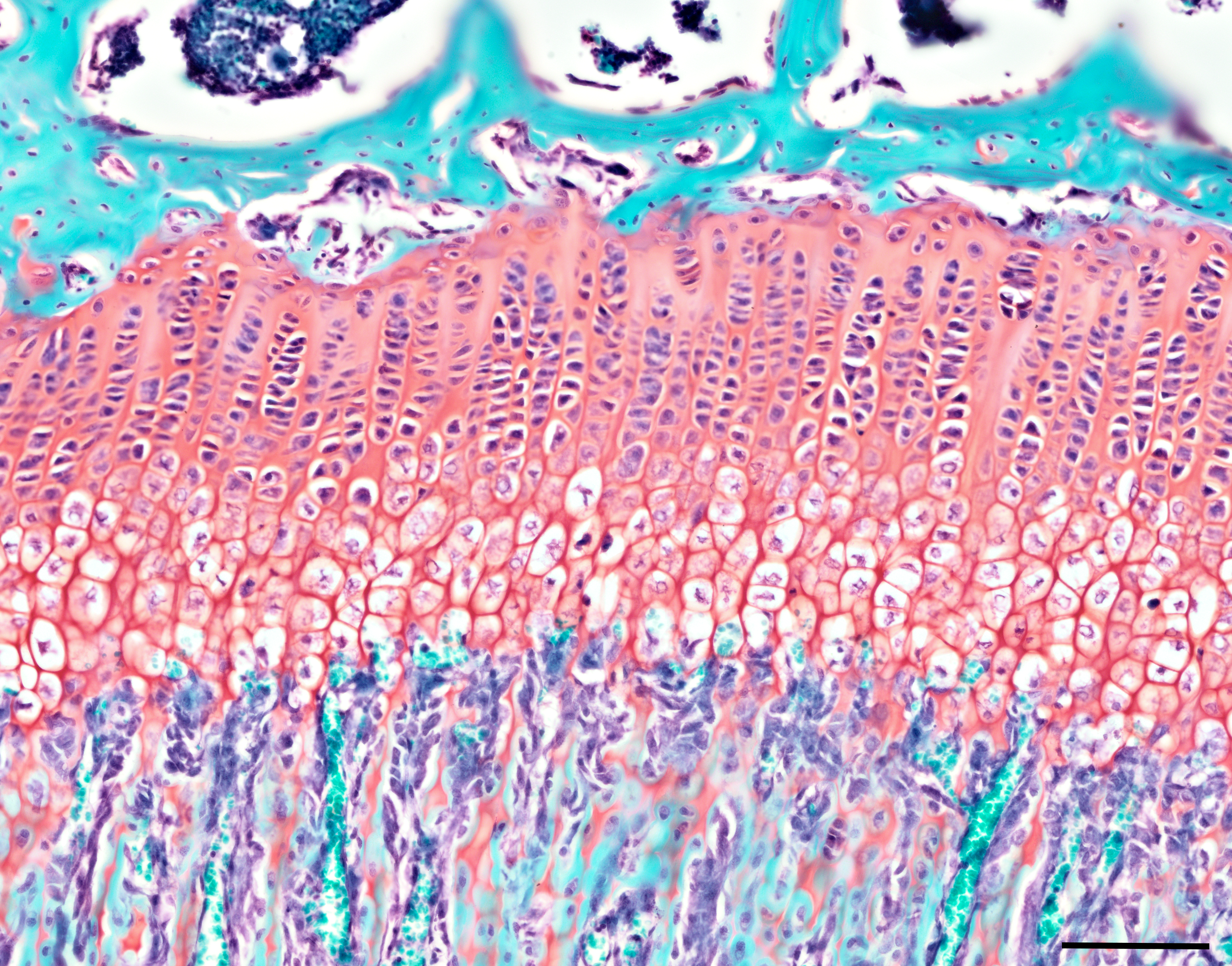

Supplement: Supplementary file 4 — Source Data Fig. 4 [file 44319_2024_93_MOESM4_ESM.zip › Figure4/4C/Veh_GP.tif]

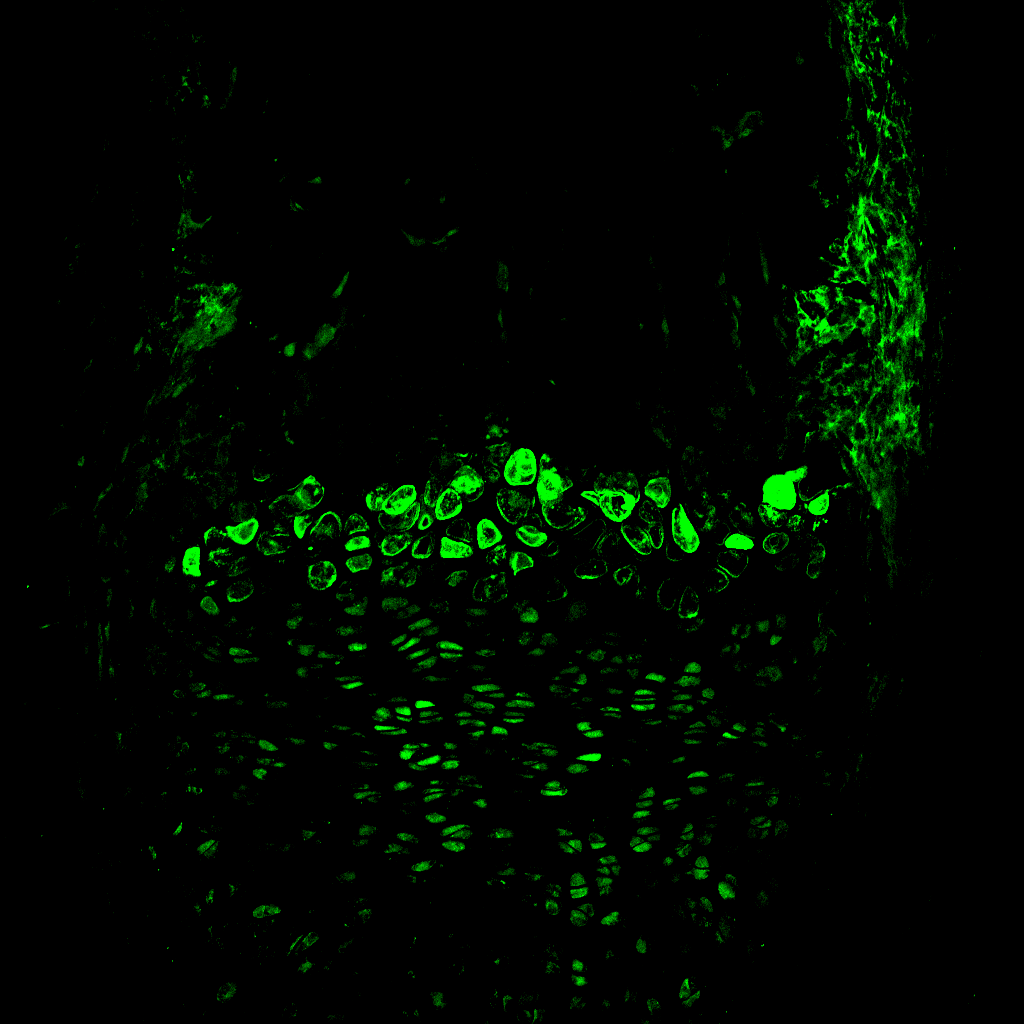

Supplement: Supplementary file 4 — Source Data Fig. 4 [file 44319_2024_93_MOESM4_ESM.zip › Figure4/4D/GDC_20X_CC_acan_green.tif]

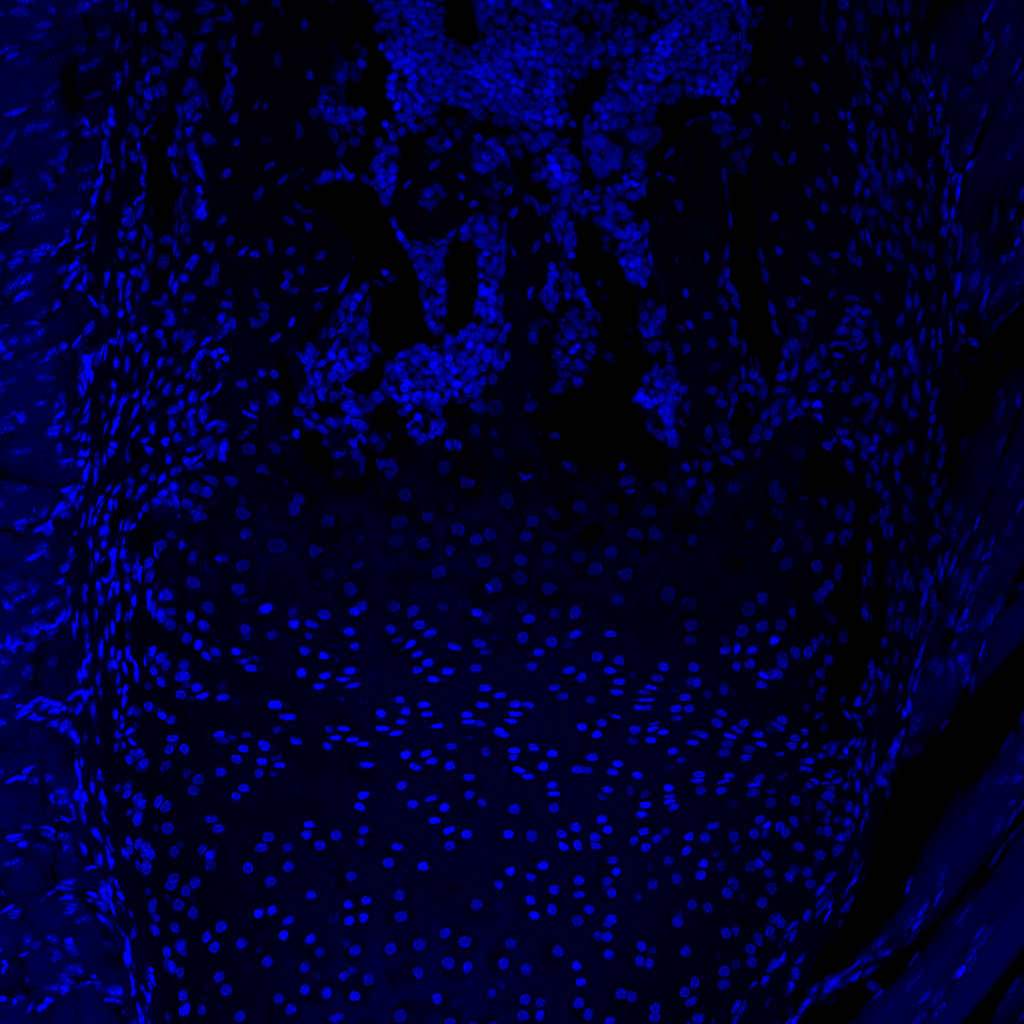

Supplement: Supplementary file 4 — Source Data Fig. 4 [file 44319_2024_93_MOESM4_ESM.zip › Figure4/4D/GDC_20X_CC_dapi_blue.tif]

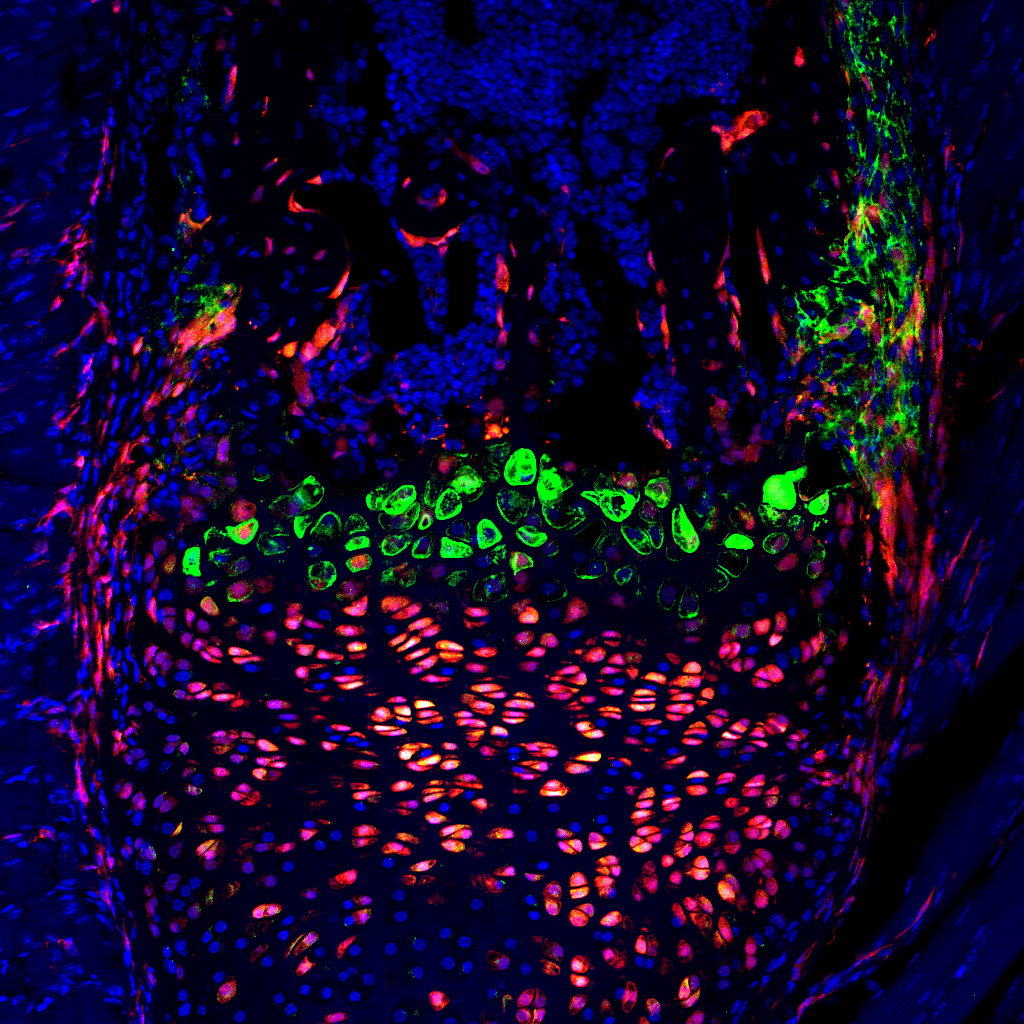

Supplement: Supplementary file 4 — Source Data Fig. 4 [file 44319_2024_93_MOESM4_ESM.zip › Figure4/4D/GDC_20X_CC_merge.tif]

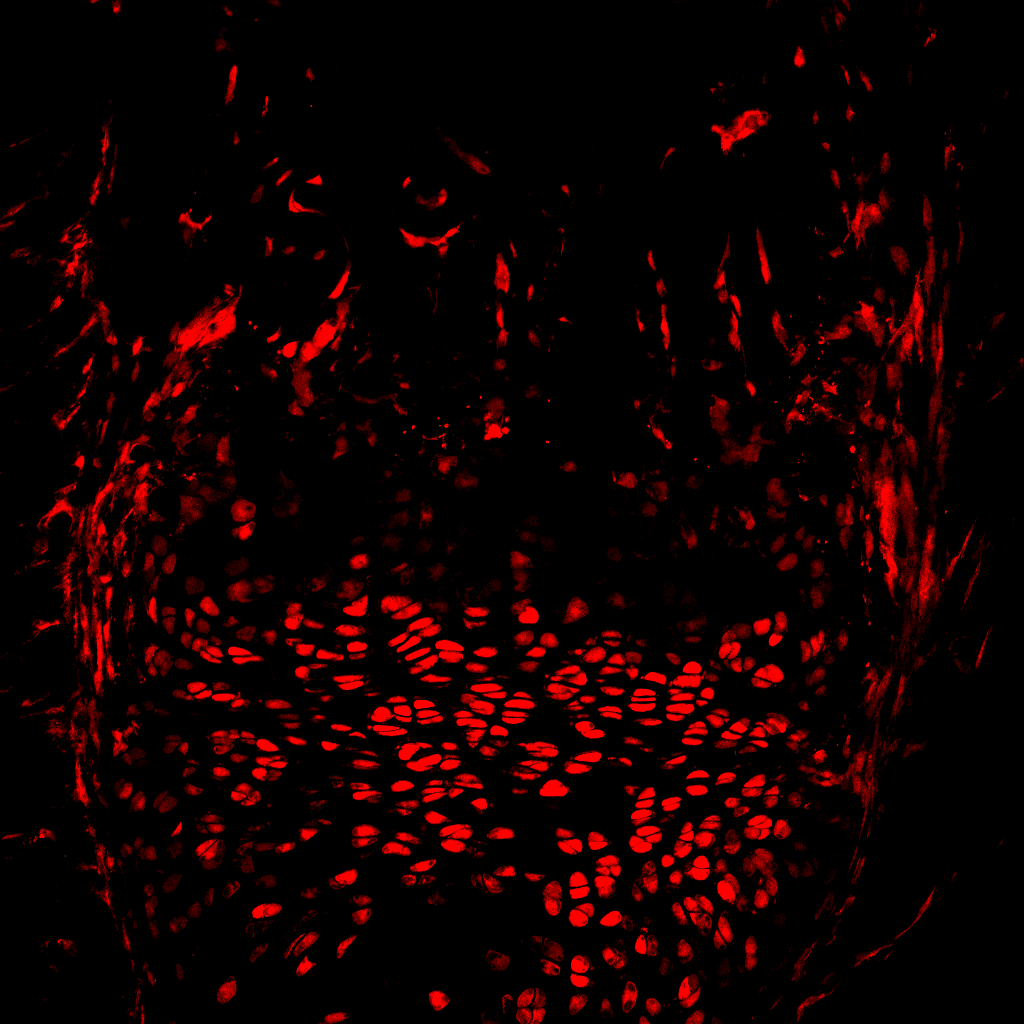

Supplement: Supplementary file 4 — Source Data Fig. 4 [file 44319_2024_93_MOESM4_ESM.zip › Figure4/4D/GDC_20X_CC_td_red.tif]

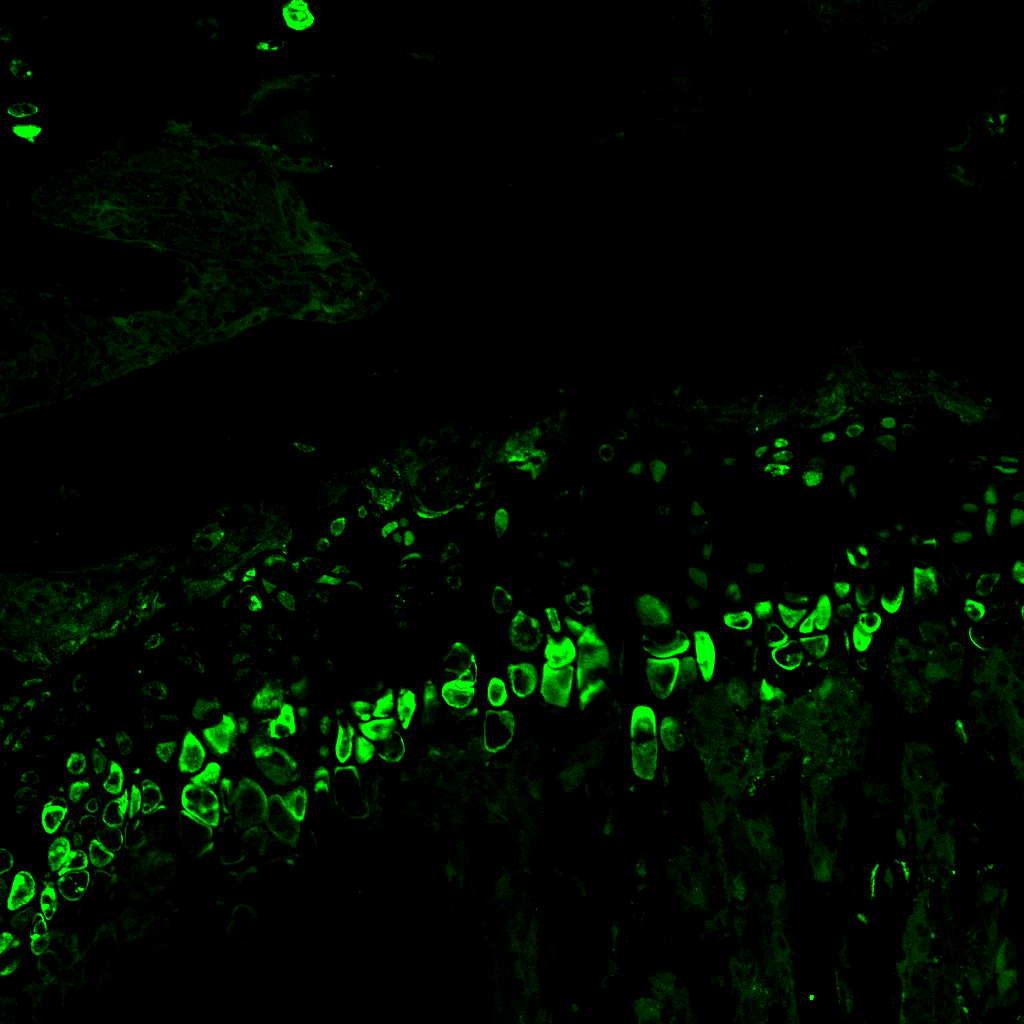

Supplement: Supplementary file 4 — Source Data Fig. 4 [file 44319_2024_93_MOESM4_ESM.zip › Figure4/4D/GDC_20X_GP_acan_green.tif]

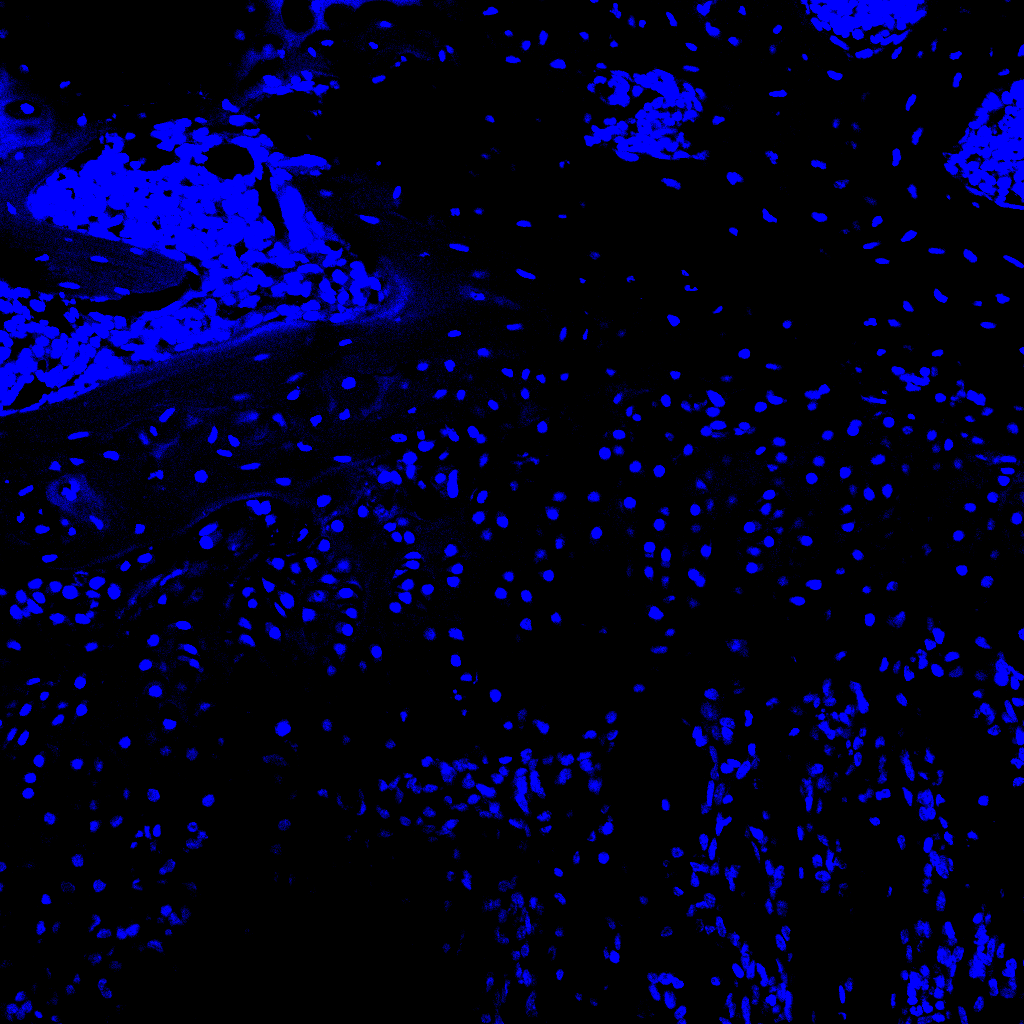

Supplement: Supplementary file 4 — Source Data Fig. 4 [file 44319_2024_93_MOESM4_ESM.zip › Figure4/4D/GDC_20X_GP_dapi_blue.tif]

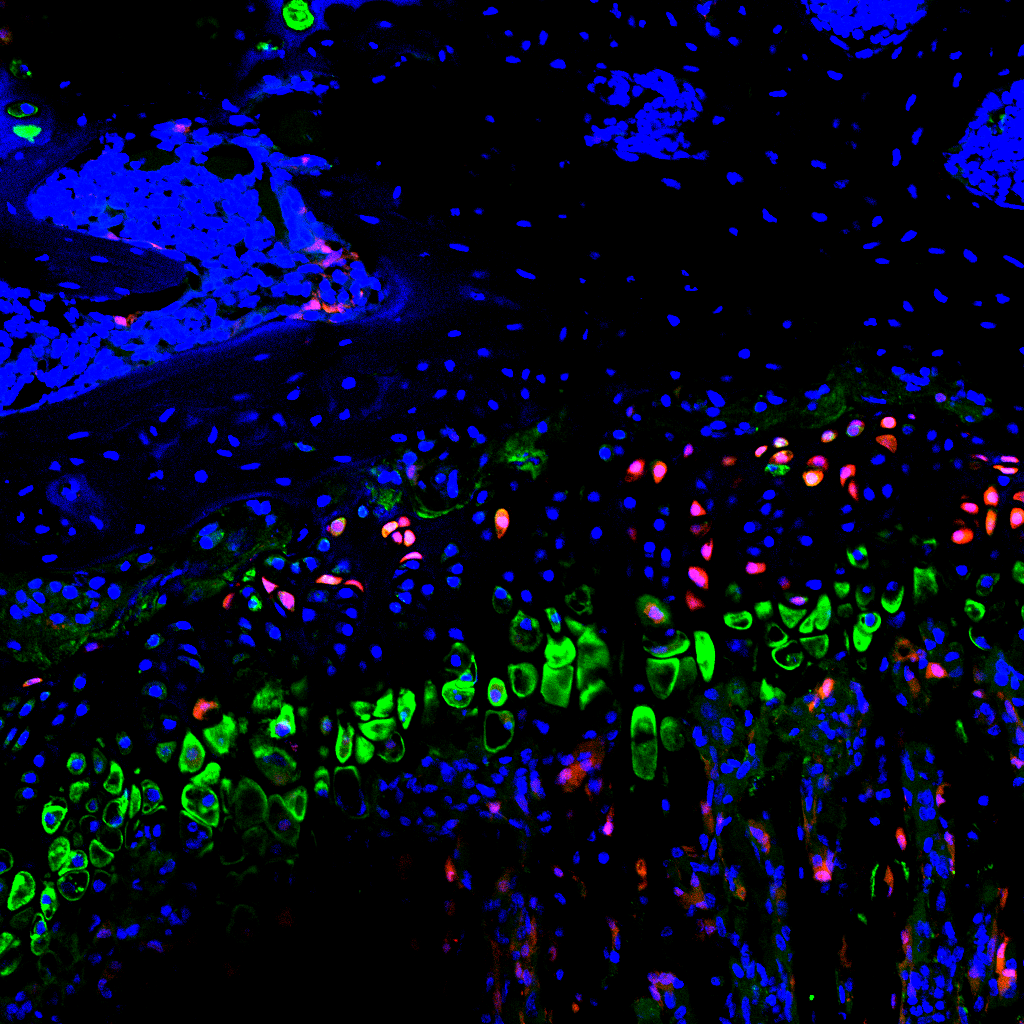

Supplement: Supplementary file 4 — Source Data Fig. 4 [file 44319_2024_93_MOESM4_ESM.zip › Figure4/4D/GDC_20X_GP_merge.tif]

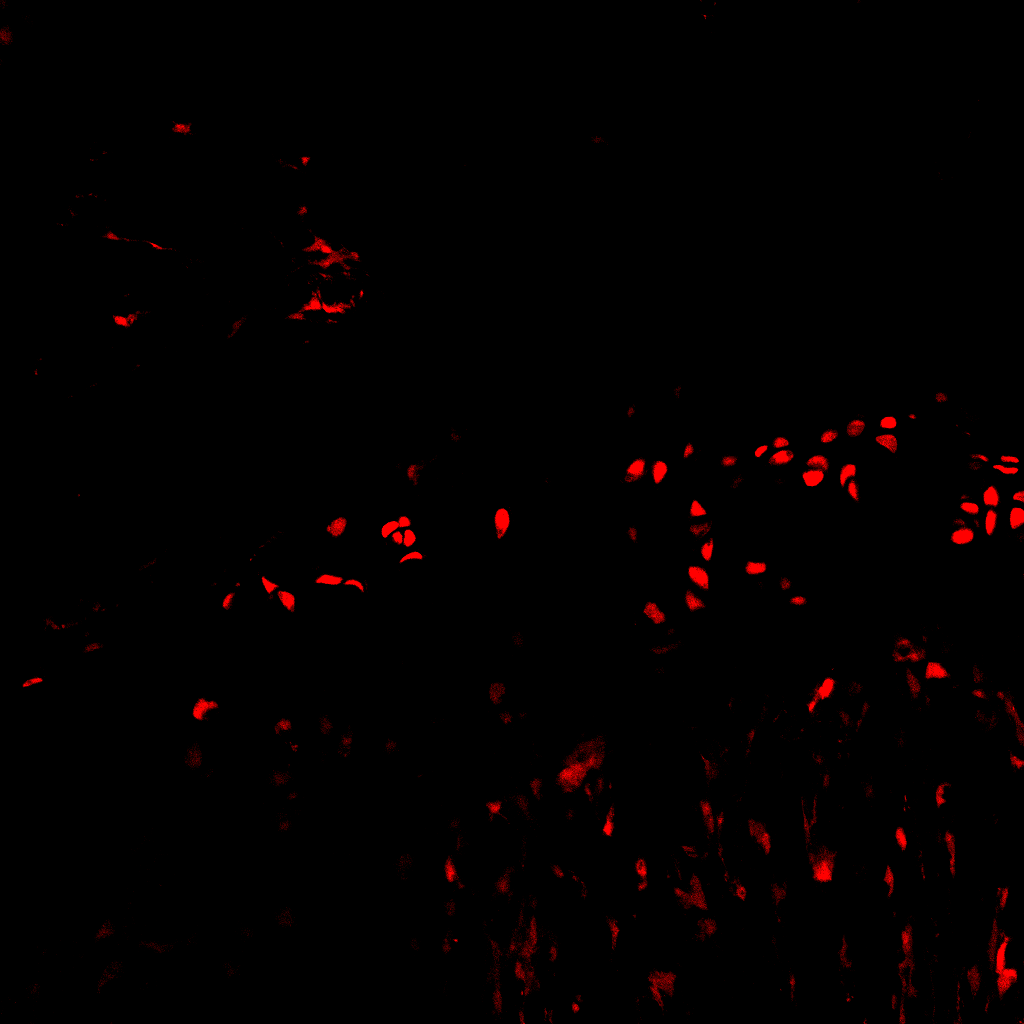

Supplement: Supplementary file 4 — Source Data Fig. 4 [file 44319_2024_93_MOESM4_ESM.zip › Figure4/4D/GDC_20X_GP_td_red.tif]

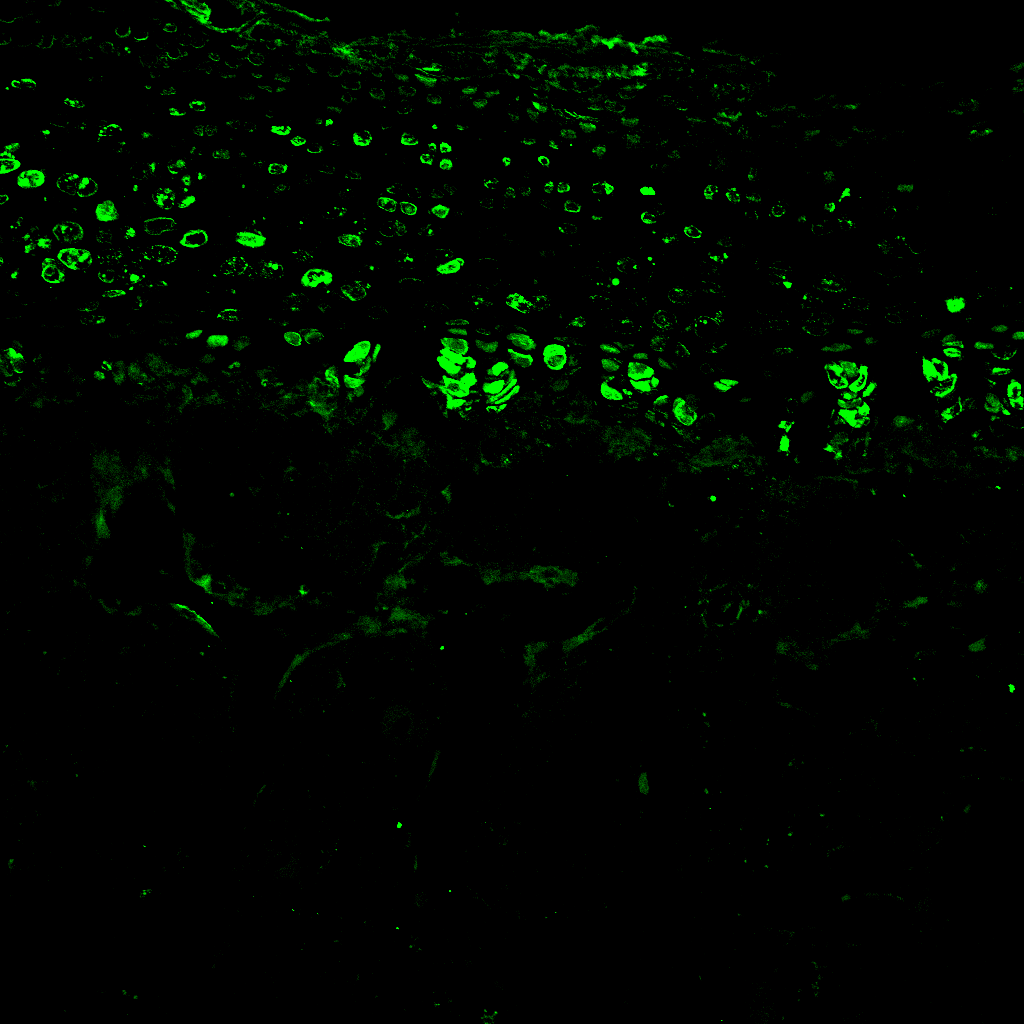

Supplement: Supplementary file 4 — Source Data Fig. 4 [file 44319_2024_93_MOESM4_ESM.zip › Figure4/4D/GDC_20X_VB_acan_green.tif]

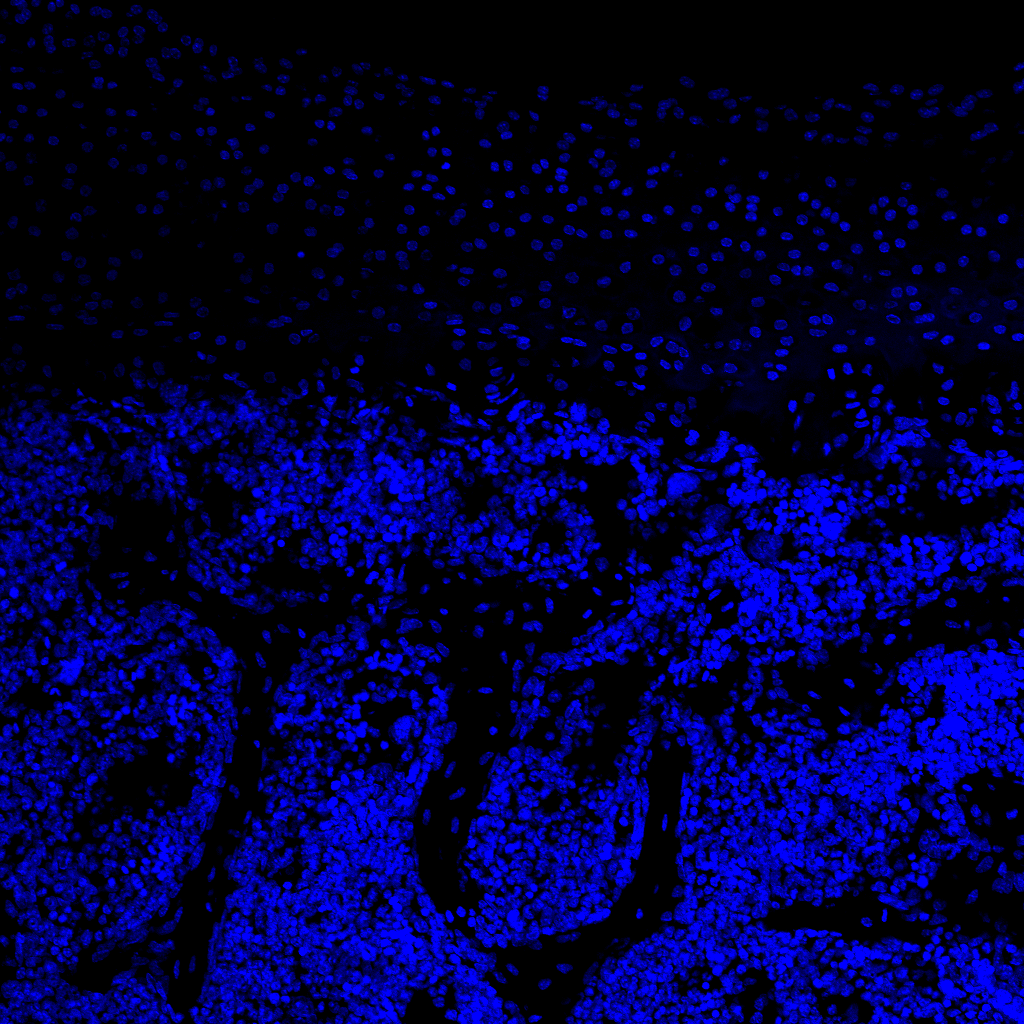

Supplement: Supplementary file 4 — Source Data Fig. 4 [file 44319_2024_93_MOESM4_ESM.zip › Figure4/4D/GDC_20X_VB_dapi_blue.tif]

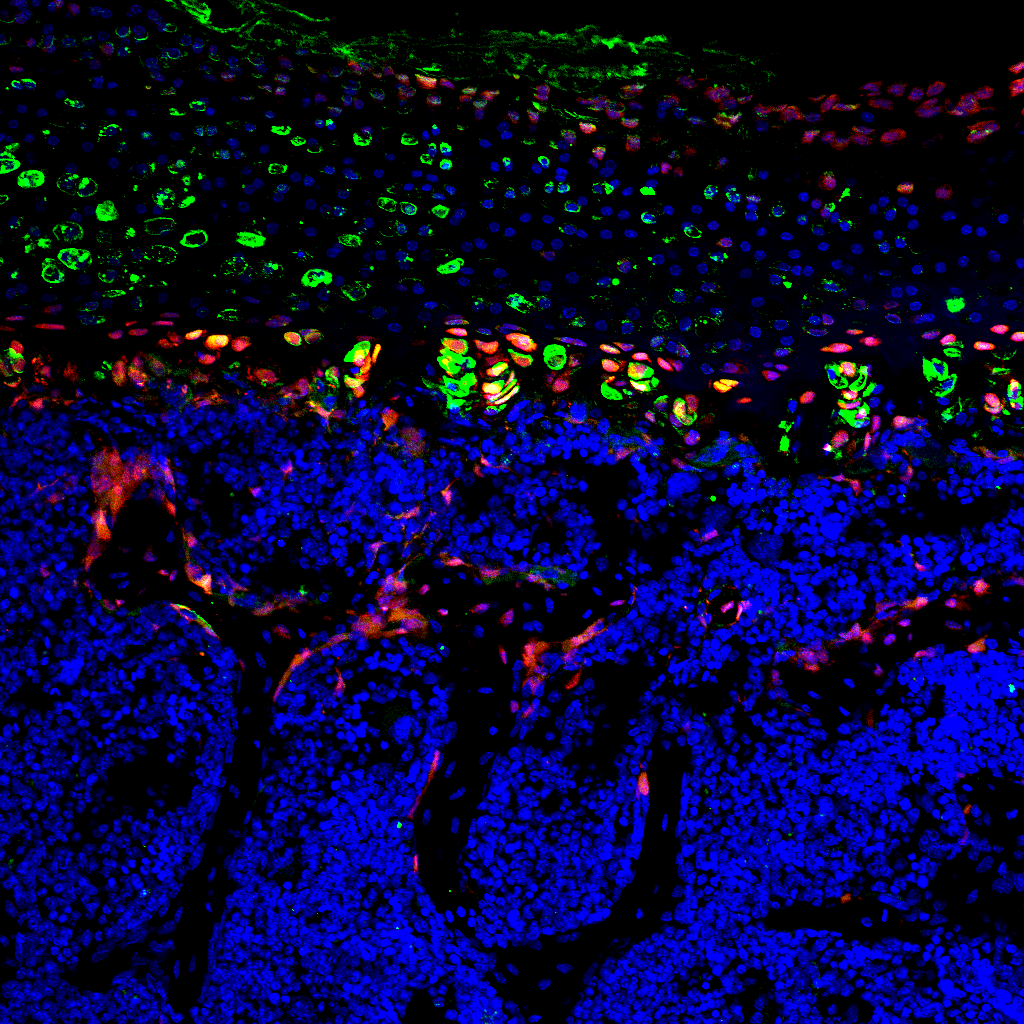

Supplement: Supplementary file 4 — Source Data Fig. 4 [file 44319_2024_93_MOESM4_ESM.zip › Figure4/4D/GDC_20X_VB_merge.tif]

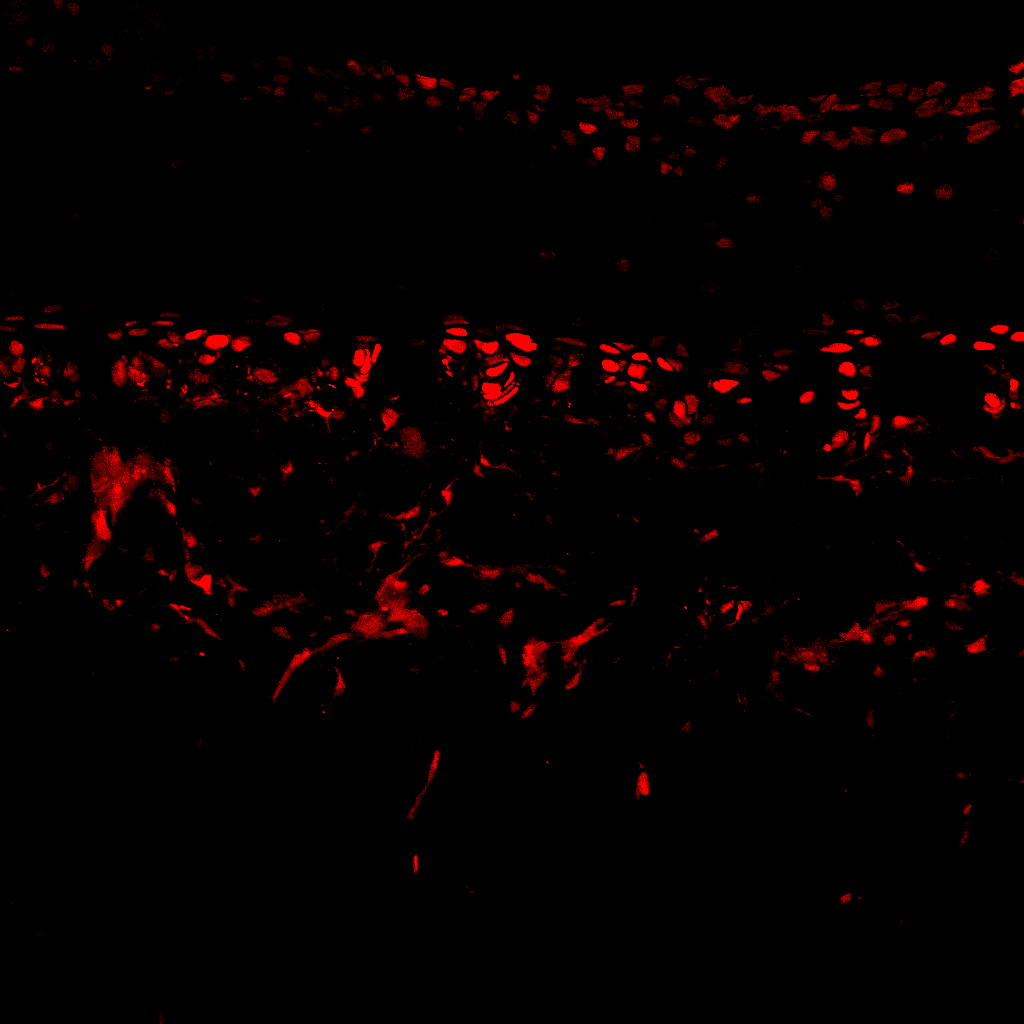

Supplement: Supplementary file 4 — Source Data Fig. 4 [file 44319_2024_93_MOESM4_ESM.zip › Figure4/4D/GDC_20X_VB_td_red.tif]

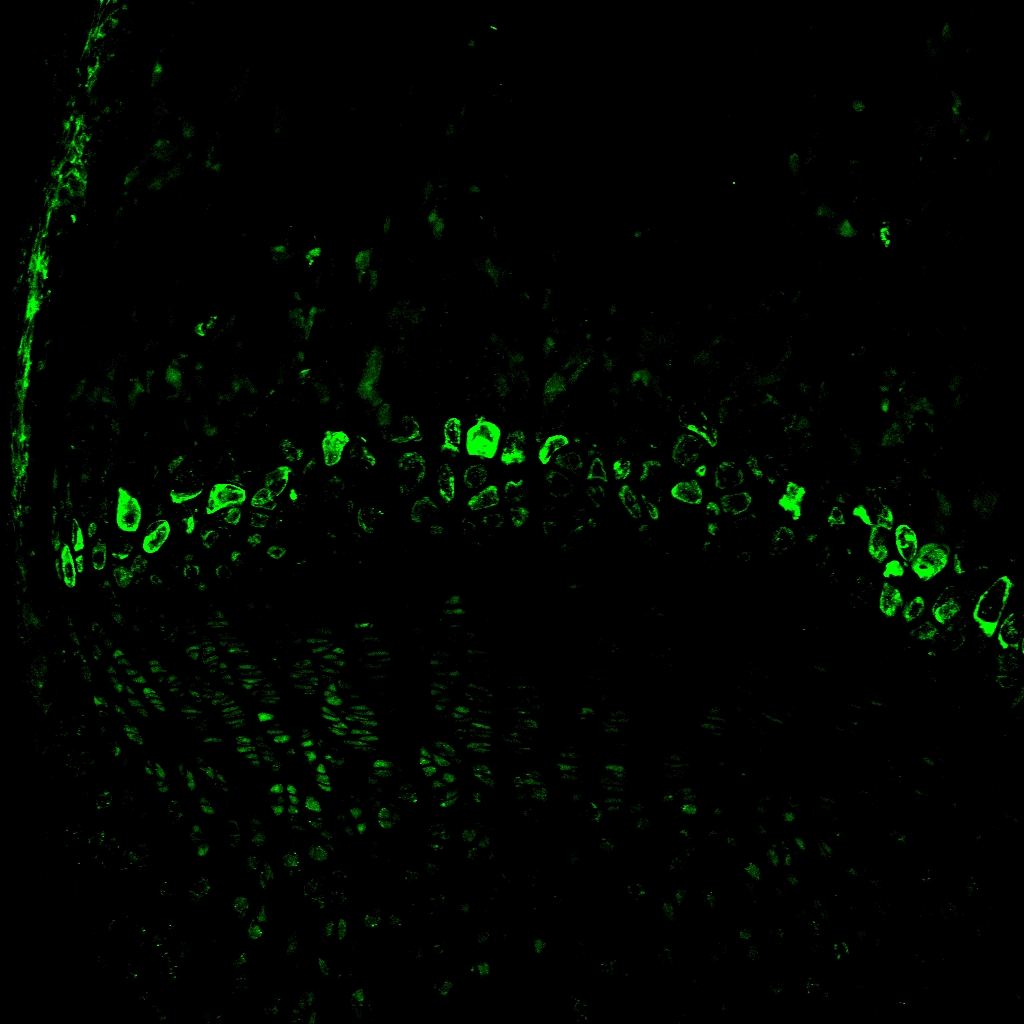

Supplement: Supplementary file 4 — Source Data Fig. 4 [file 44319_2024_93_MOESM4_ESM.zip › Figure4/4D/Veh_20X_CC_acan_green.tif]

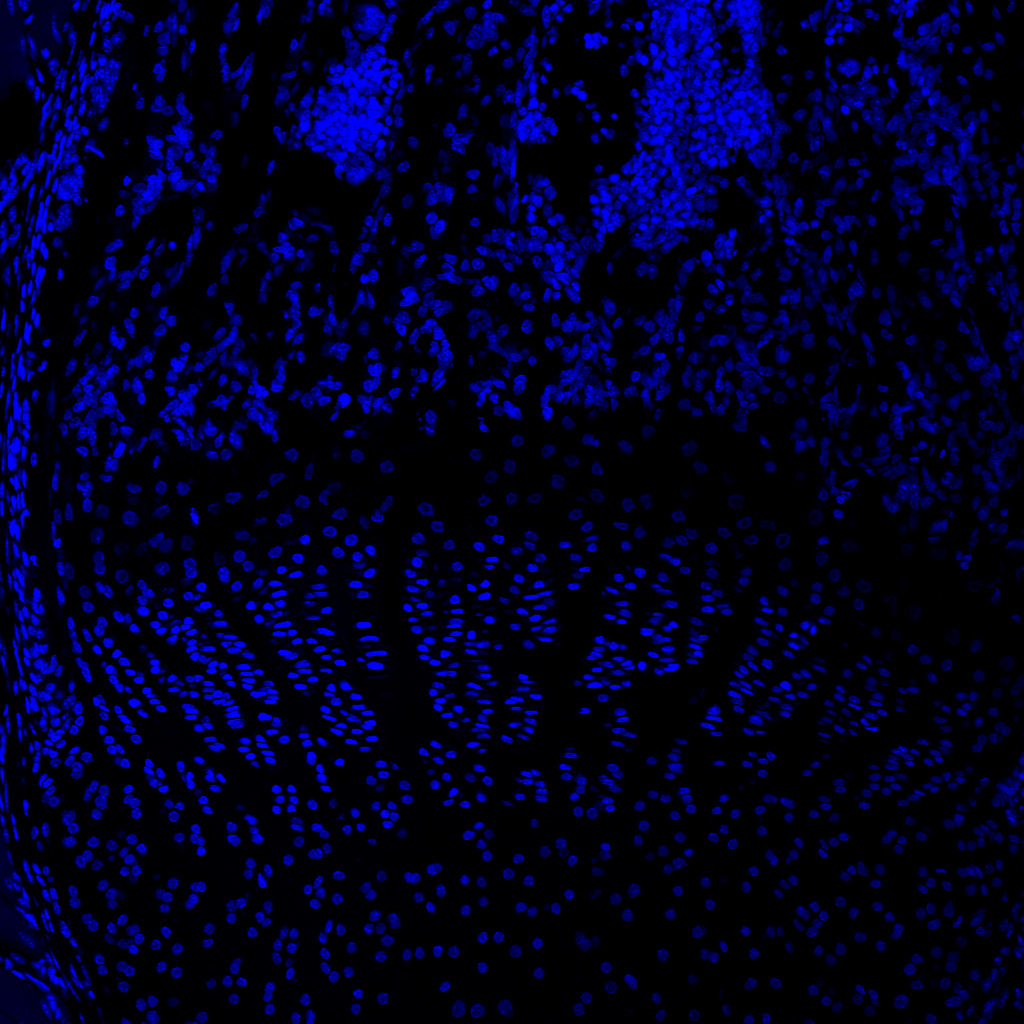

Supplement: Supplementary file 4 — Source Data Fig. 4 [file 44319_2024_93_MOESM4_ESM.zip › Figure4/4D/Veh_20X_CC_dapi_blue.tif]

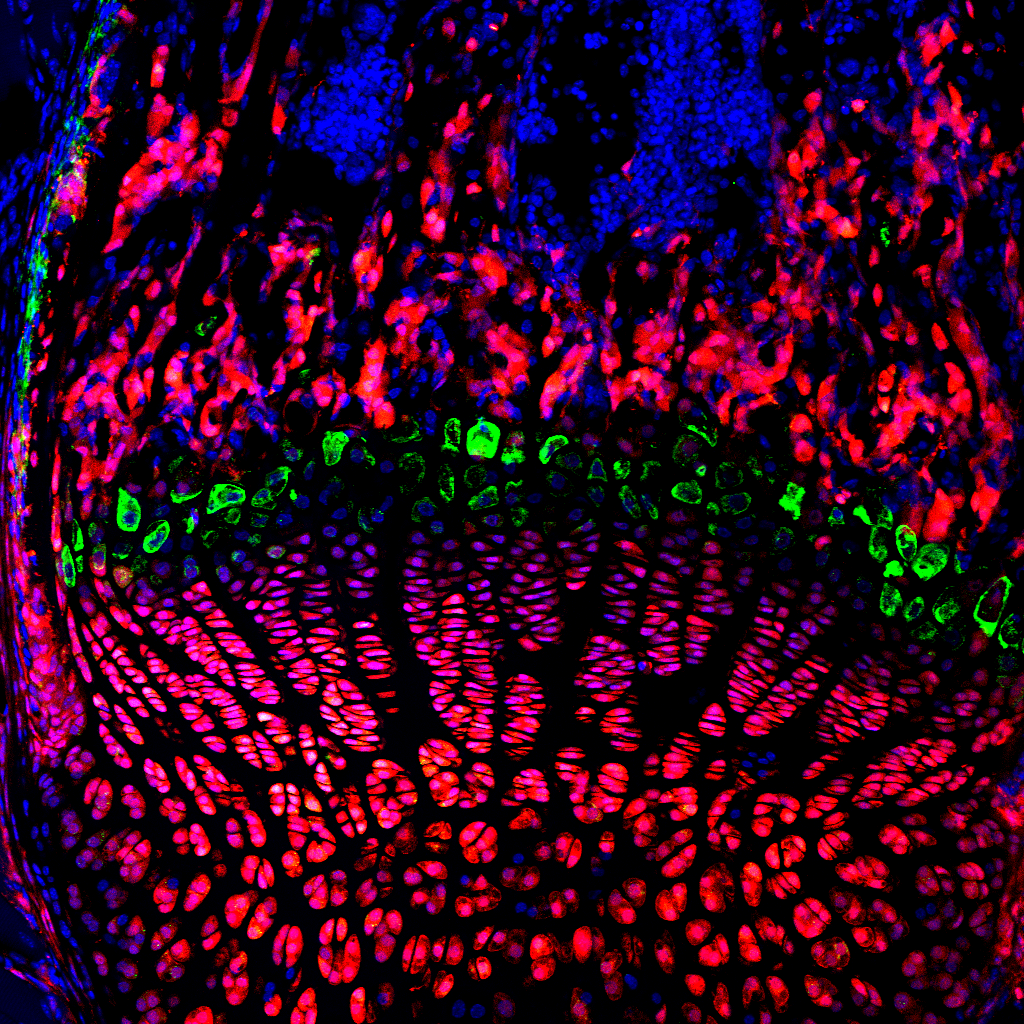

Supplement: Supplementary file 4 — Source Data Fig. 4 [file 44319_2024_93_MOESM4_ESM.zip › Figure4/4D/Veh_20X_CC_merge.tif]

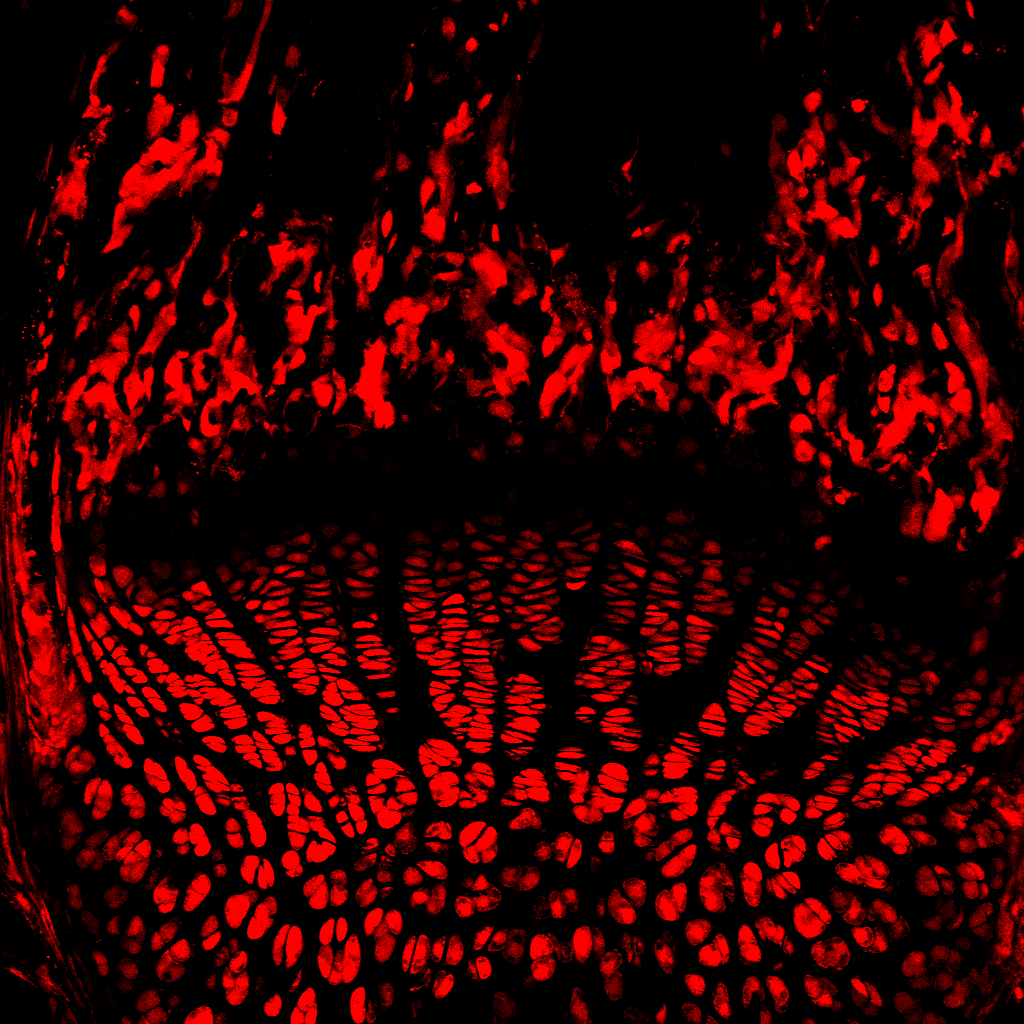

Supplement: Supplementary file 4 — Source Data Fig. 4 [file 44319_2024_93_MOESM4_ESM.zip › Figure4/4D/Veh_20X_CC_td_red.tif]

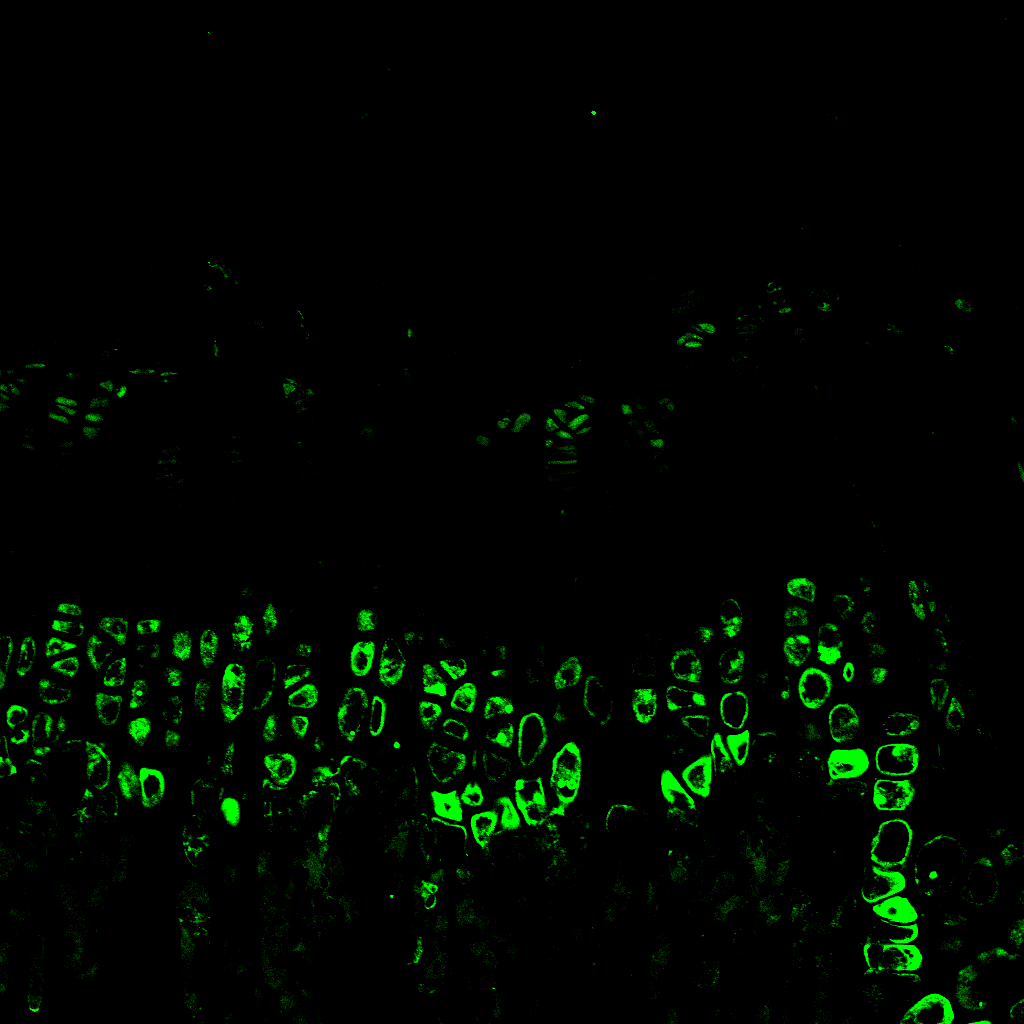

Supplement: Supplementary file 4 — Source Data Fig. 4 [file 44319_2024_93_MOESM4_ESM.zip › Figure4/4D/Veh_20X_GP_acan_green.tif]

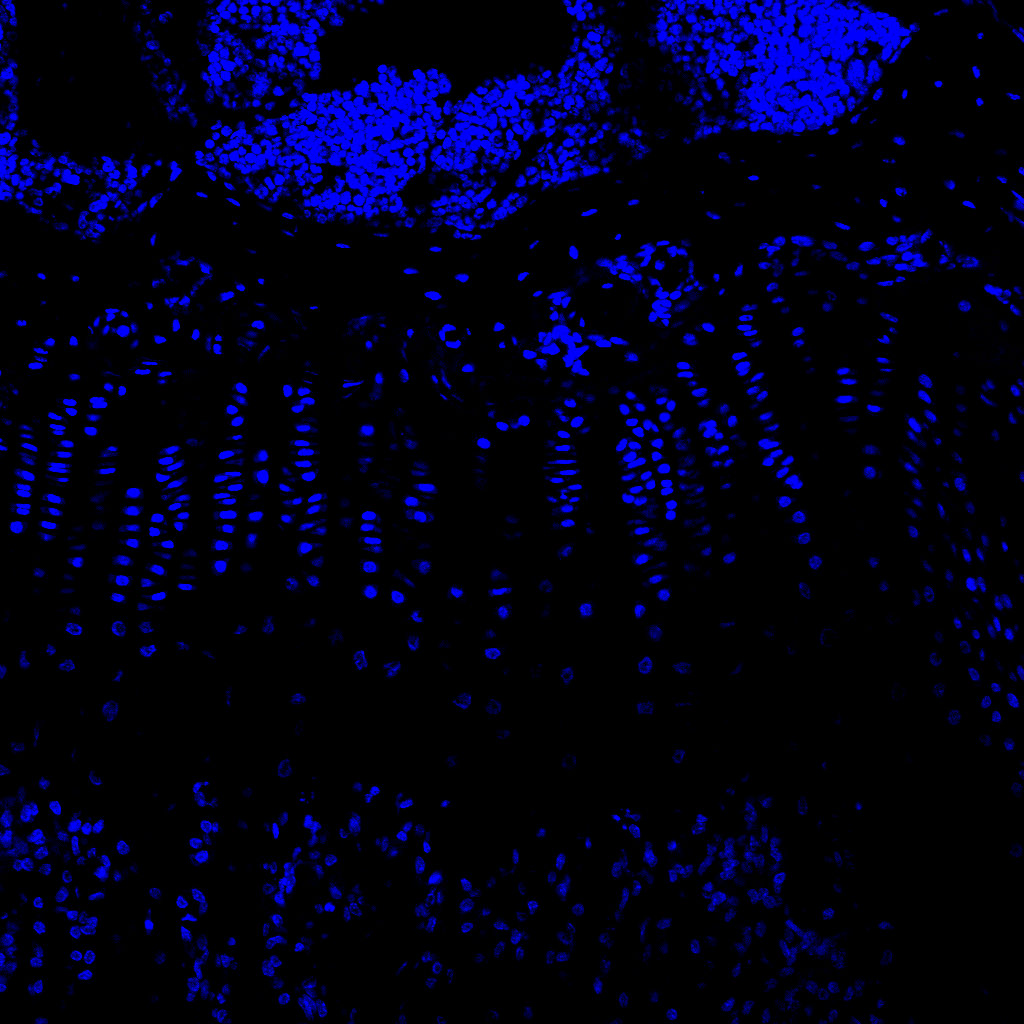

Supplement: Supplementary file 4 — Source Data Fig. 4 [file 44319_2024_93_MOESM4_ESM.zip › Figure4/4D/Veh_20X_GP_dapi_blue.tif]

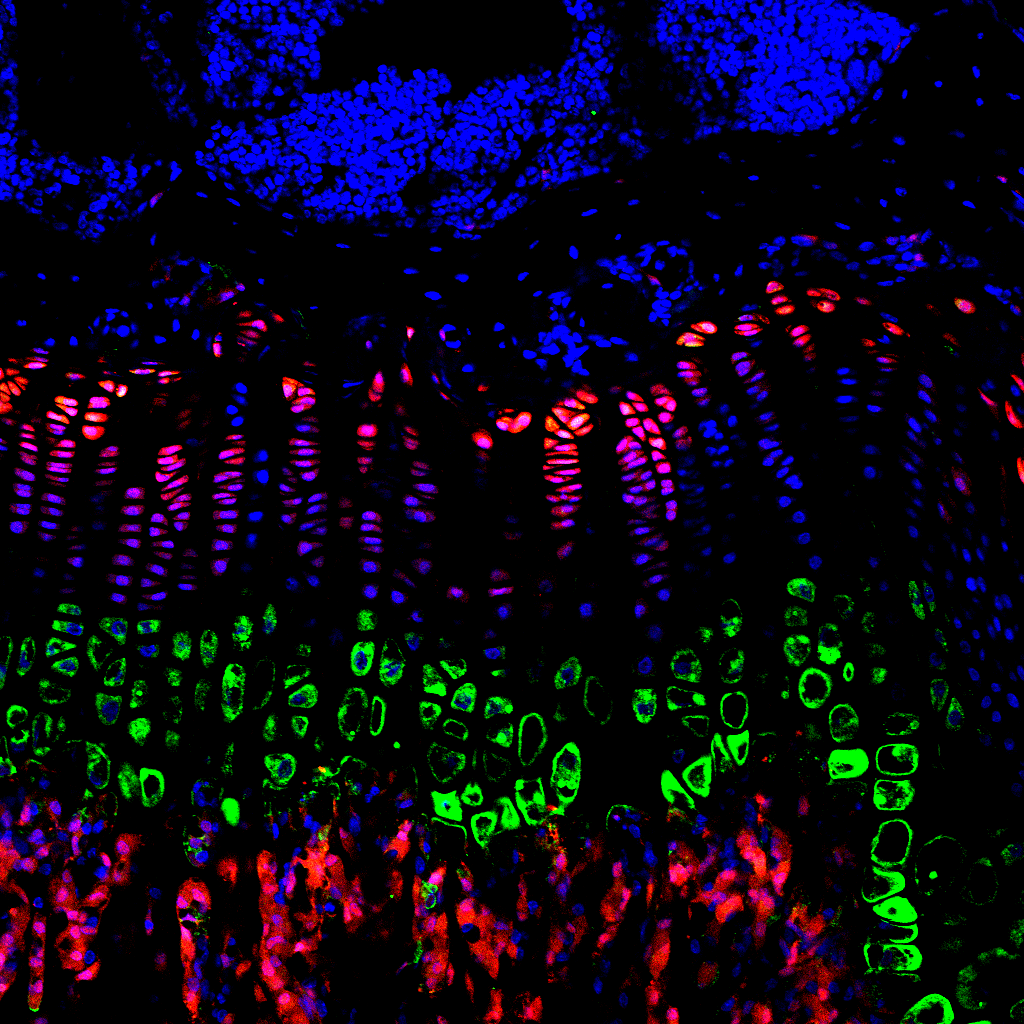

Supplement: Supplementary file 4 — Source Data Fig. 4 [file 44319_2024_93_MOESM4_ESM.zip › Figure4/4D/Veh_20X_GP_merge.tif]

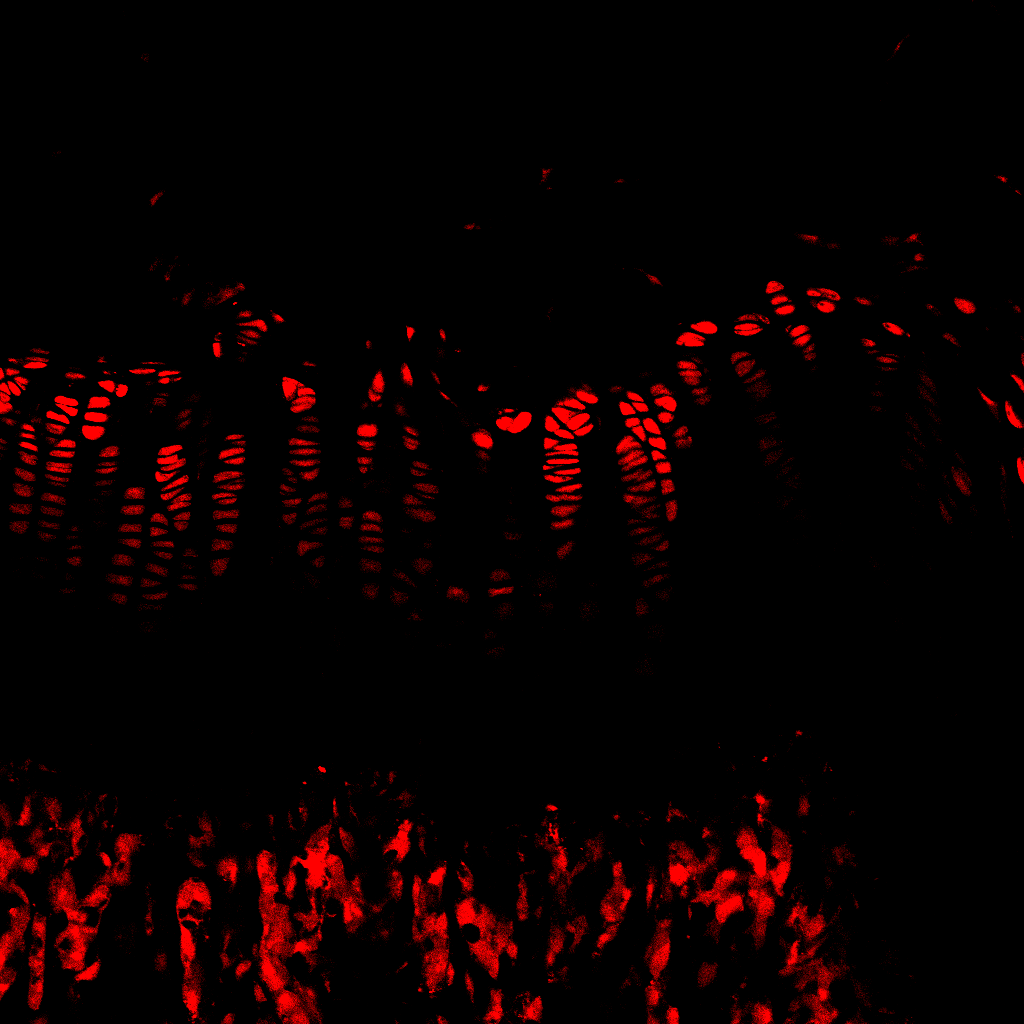

Supplement: Supplementary file 4 — Source Data Fig. 4 [file 44319_2024_93_MOESM4_ESM.zip › Figure4/4D/Veh_20X_GP_td_red.tif]

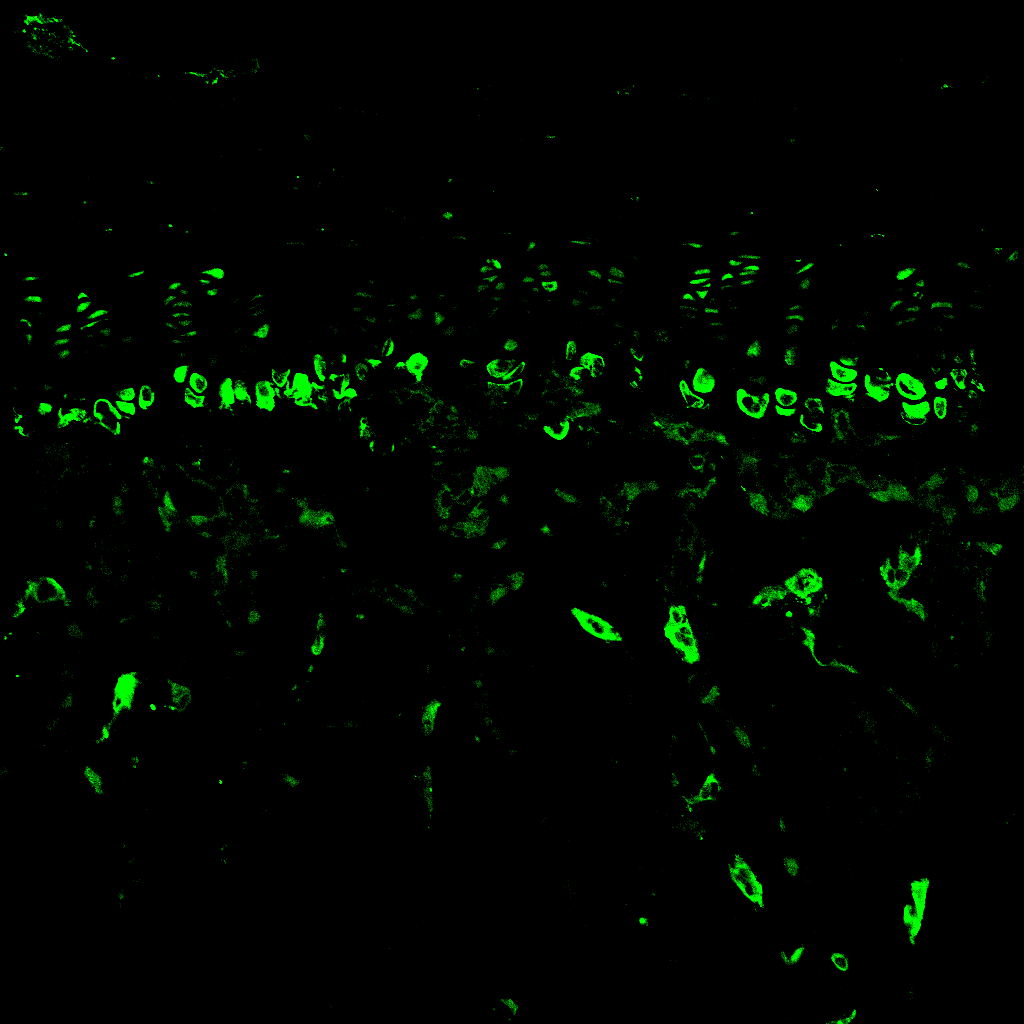

Supplement: Supplementary file 4 — Source Data Fig. 4 [file 44319_2024_93_MOESM4_ESM.zip › Figure4/4D/Veh_20X_VB_acan_green.tif]

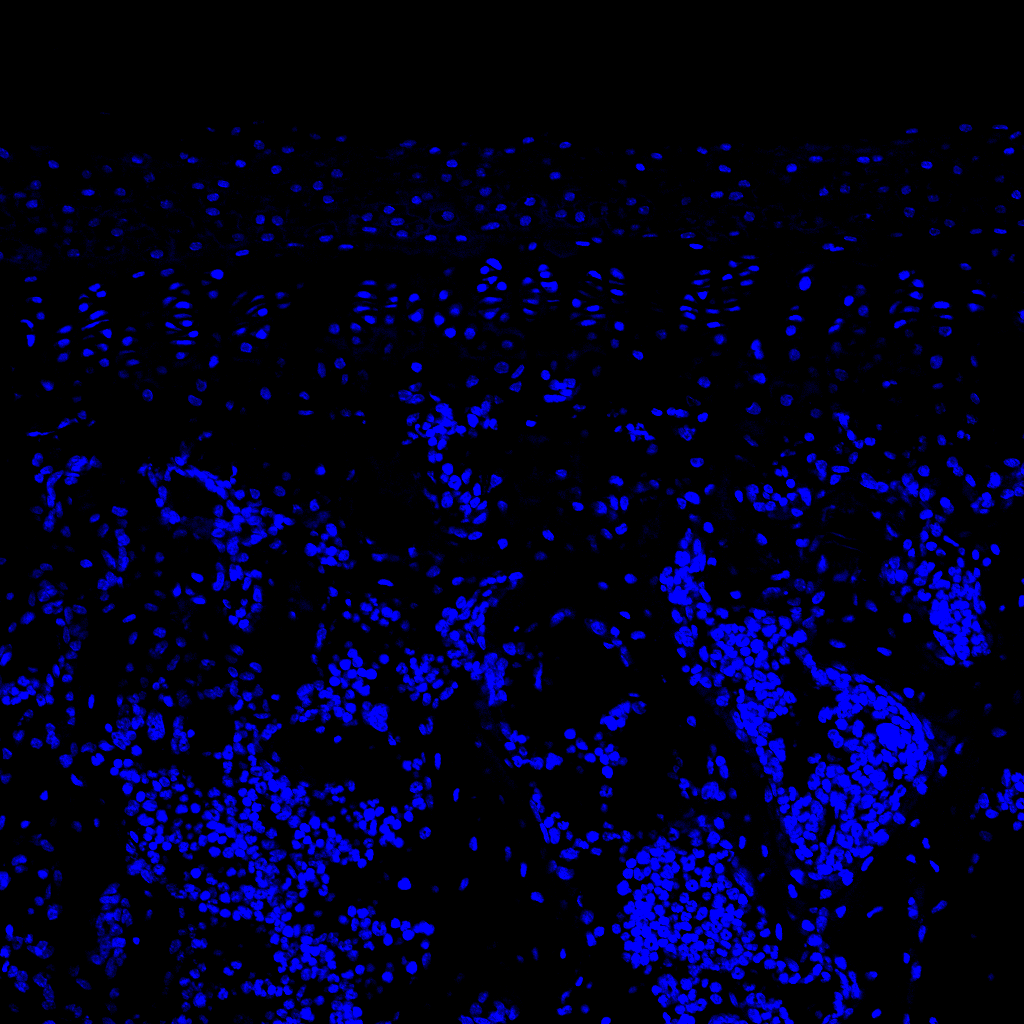

Supplement: Supplementary file 4 — Source Data Fig. 4 [file 44319_2024_93_MOESM4_ESM.zip › Figure4/4D/Veh_20X_VB_dapi_blue.tif]

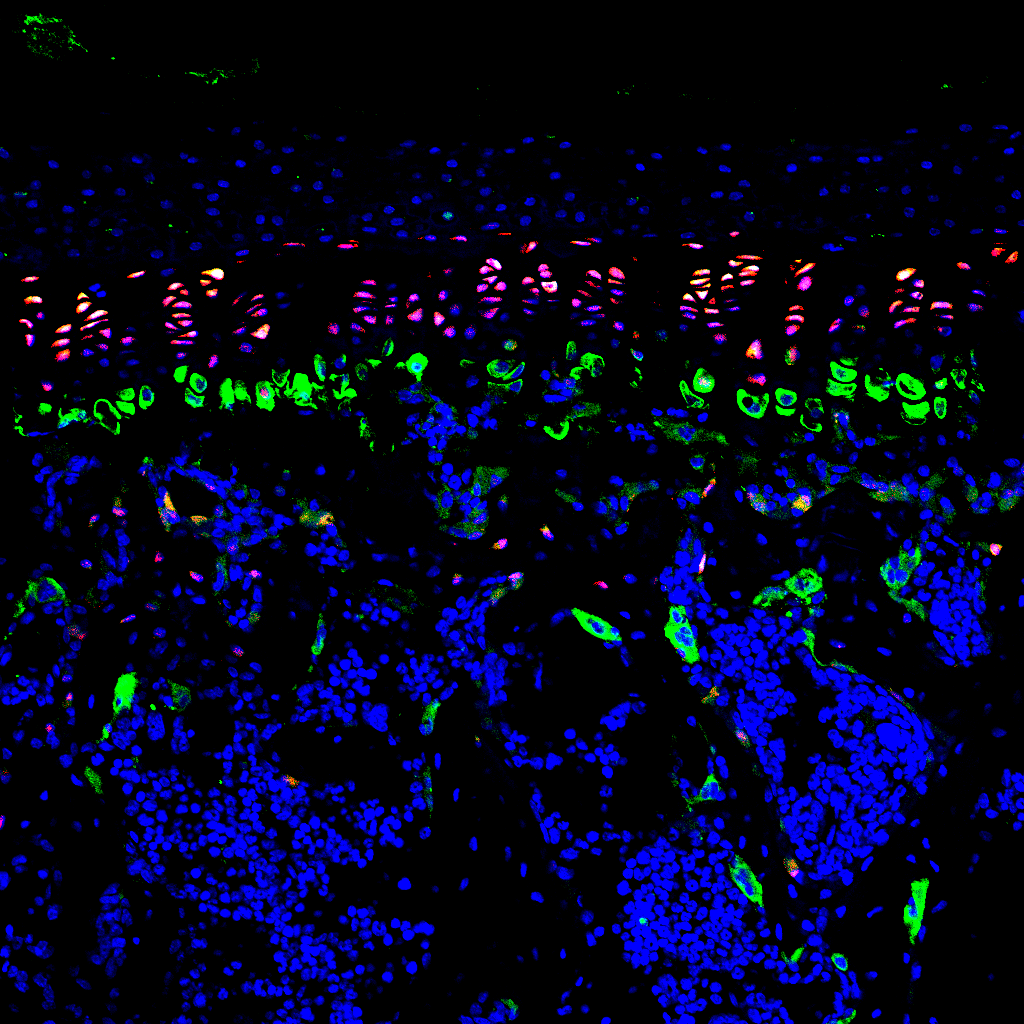

Supplement: Supplementary file 4 — Source Data Fig. 4 [file 44319_2024_93_MOESM4_ESM.zip › Figure4/4D/Veh_20X_VB_merge.tif]

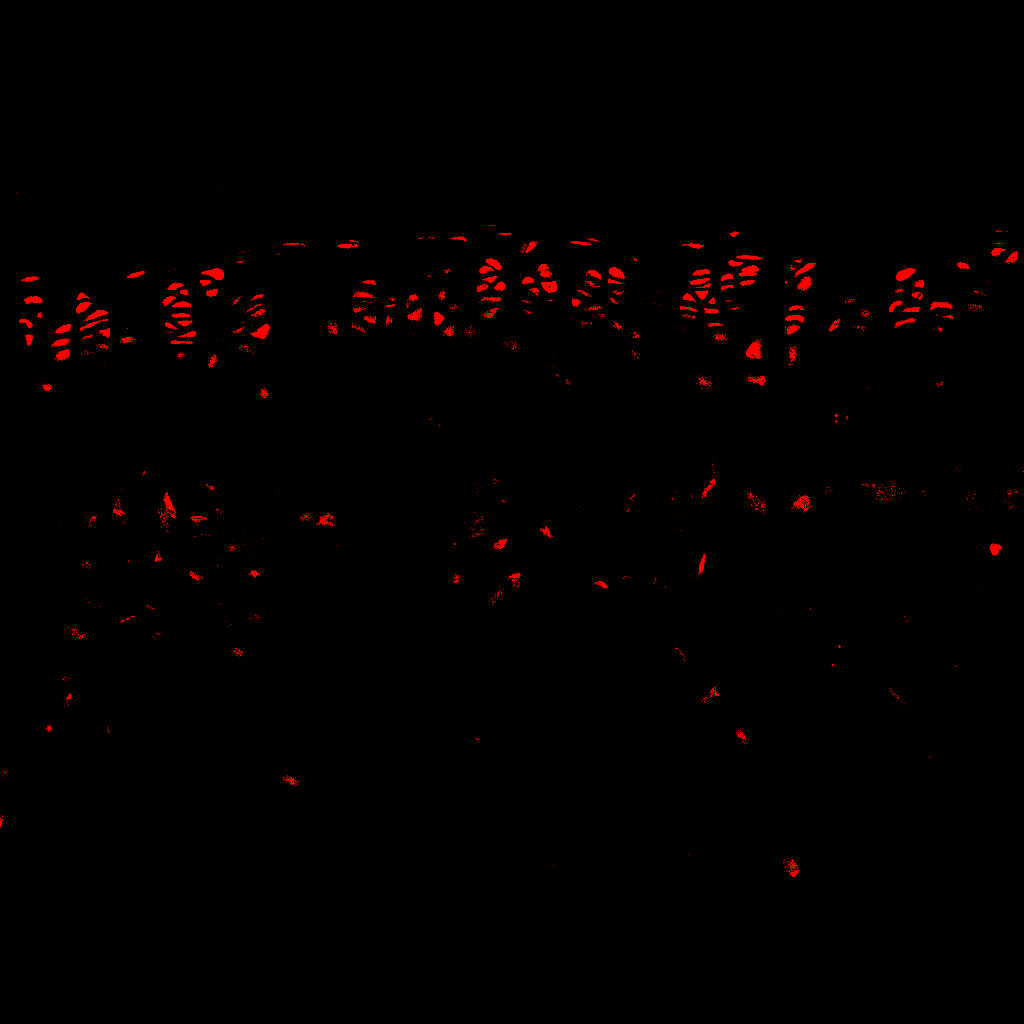

Supplement: Supplementary file 4 — Source Data Fig. 4 [file 44319_2024_93_MOESM4_ESM.zip › Figure4/4D/Veh_20X_VB_td_red.tif]

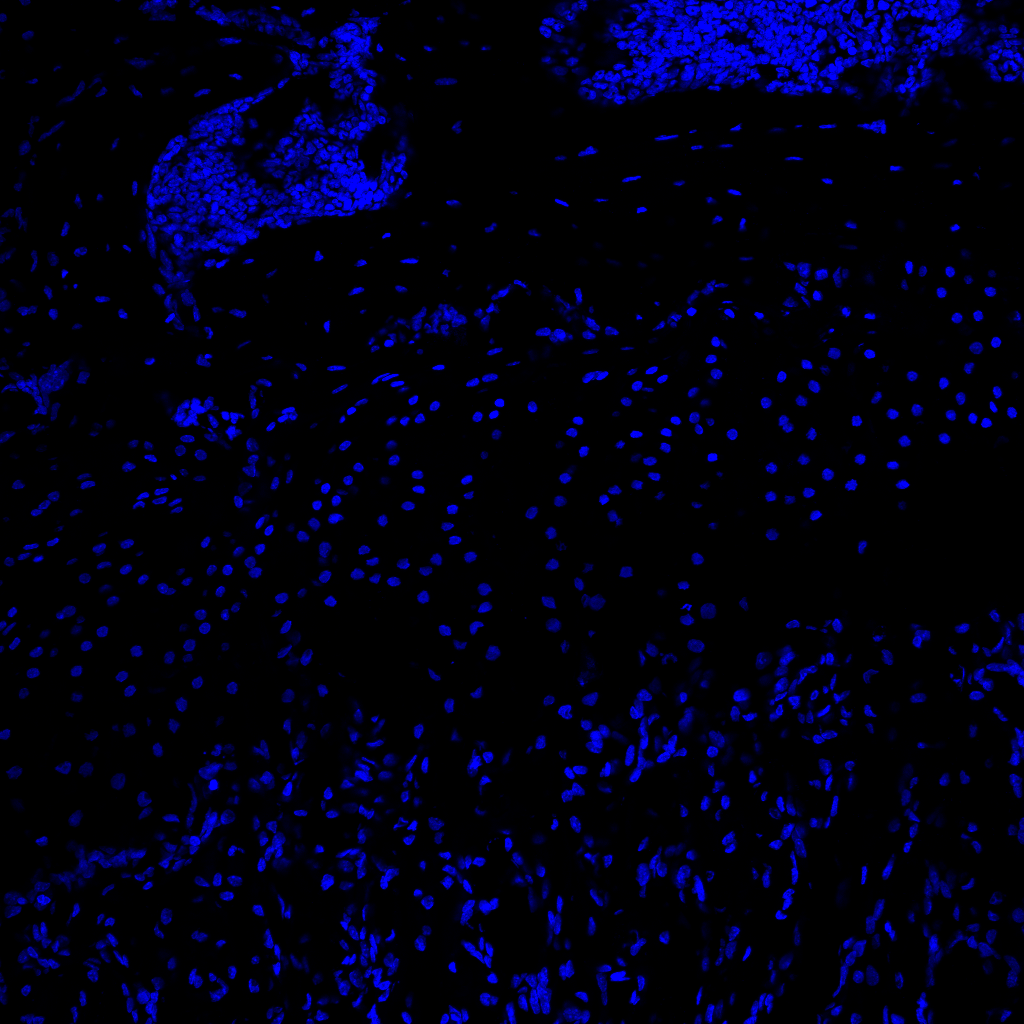

Supplement: Supplementary file 4 — Source Data Fig. 4 [file 44319_2024_93_MOESM4_ESM.zip › Figure4/4H/GDC_GP_20X_dapi_blue.tif]

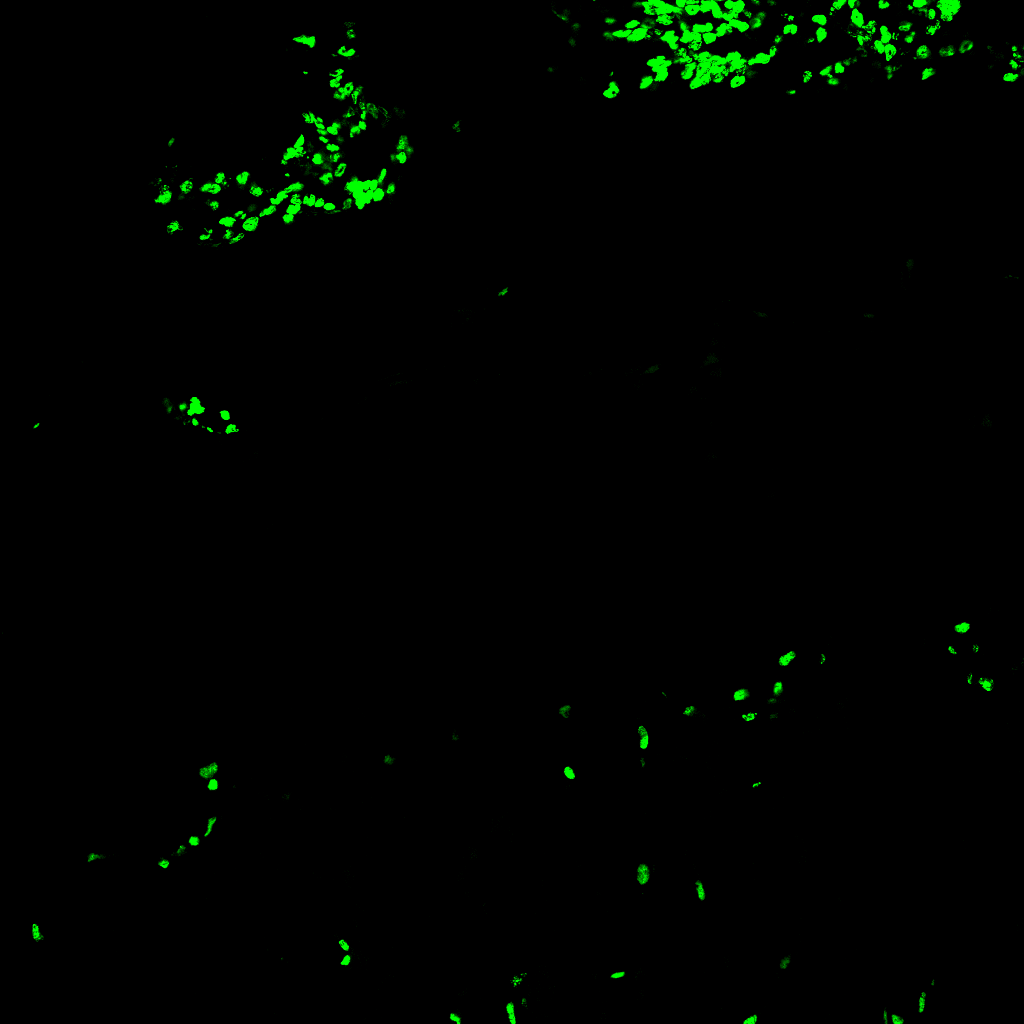

Supplement: Supplementary file 4 — Source Data Fig. 4 [file 44319_2024_93_MOESM4_ESM.zip › Figure4/4H/GDC_GP_20X_edu_green.tif]

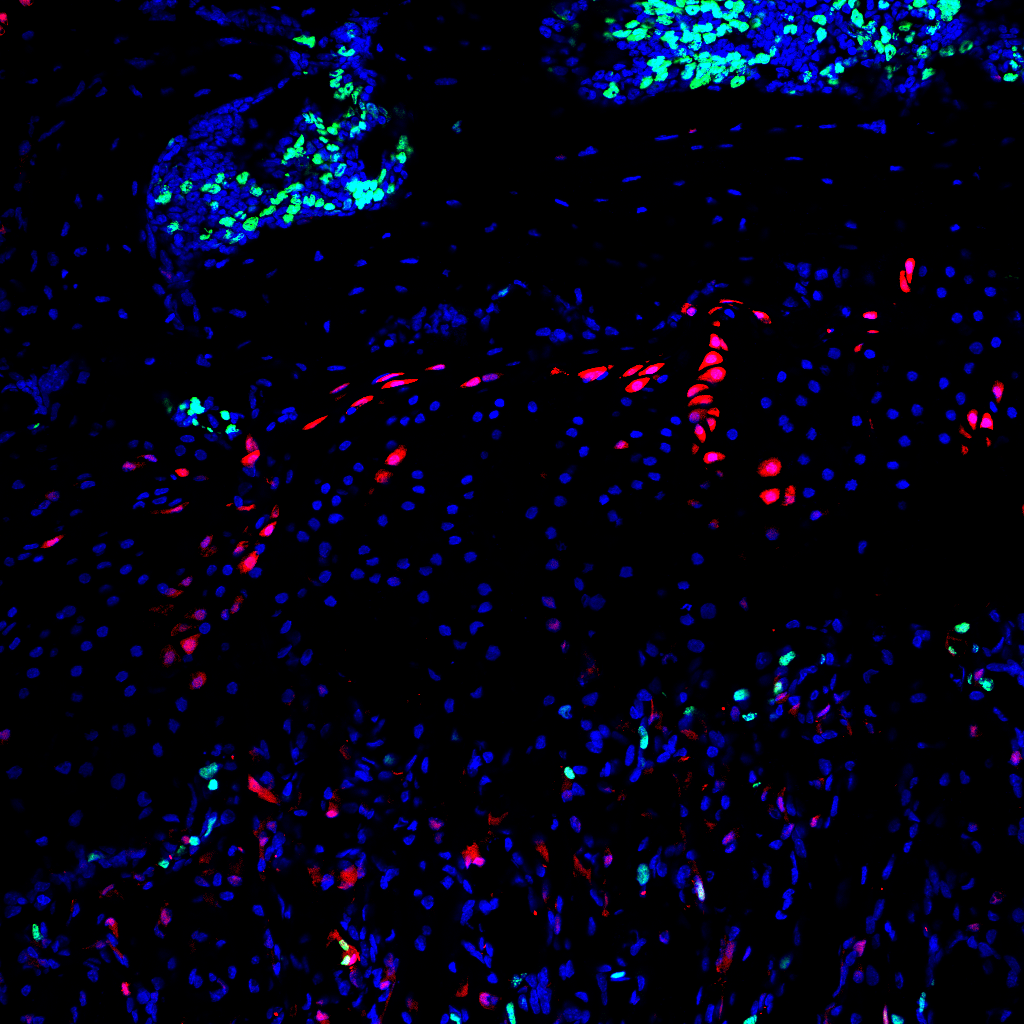

Supplement: Supplementary file 4 — Source Data Fig. 4 [file 44319_2024_93_MOESM4_ESM.zip › Figure4/4H/GDC_GP_20X_merge.tif]

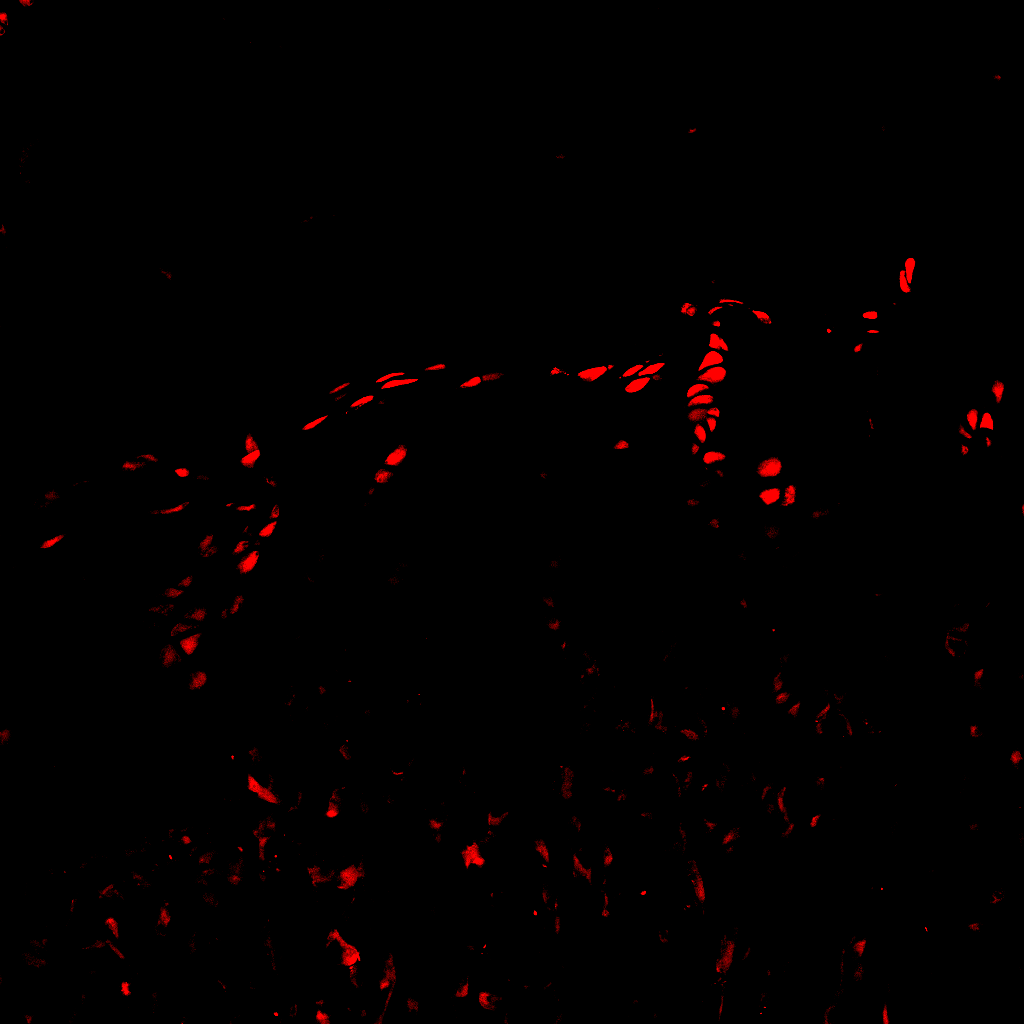

Supplement: Supplementary file 4 — Source Data Fig. 4 [file 44319_2024_93_MOESM4_ESM.zip › Figure4/4H/GDC_GP_20X_td_red.tif]

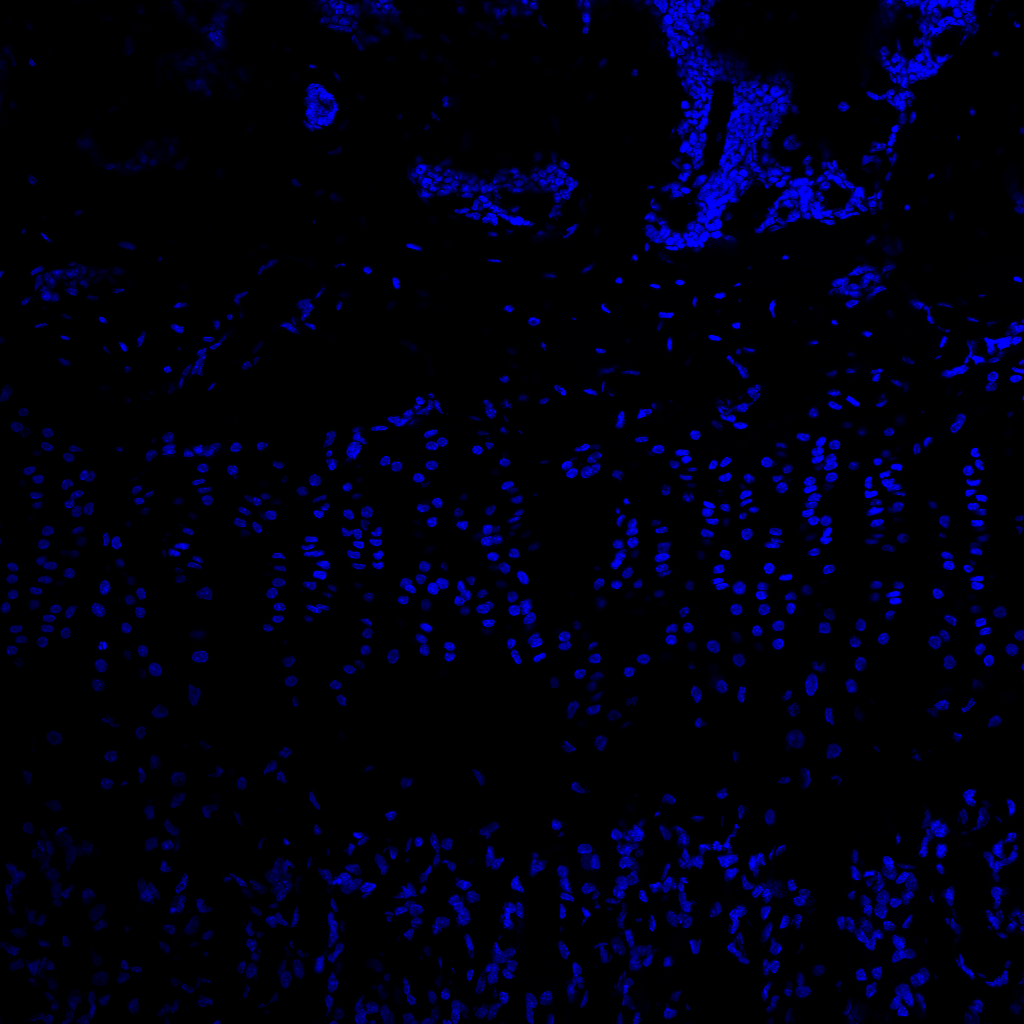

Supplement: Supplementary file 4 — Source Data Fig. 4 [file 44319_2024_93_MOESM4_ESM.zip › Figure4/4H/Veh_GP_20X_dapi_blue.tif]

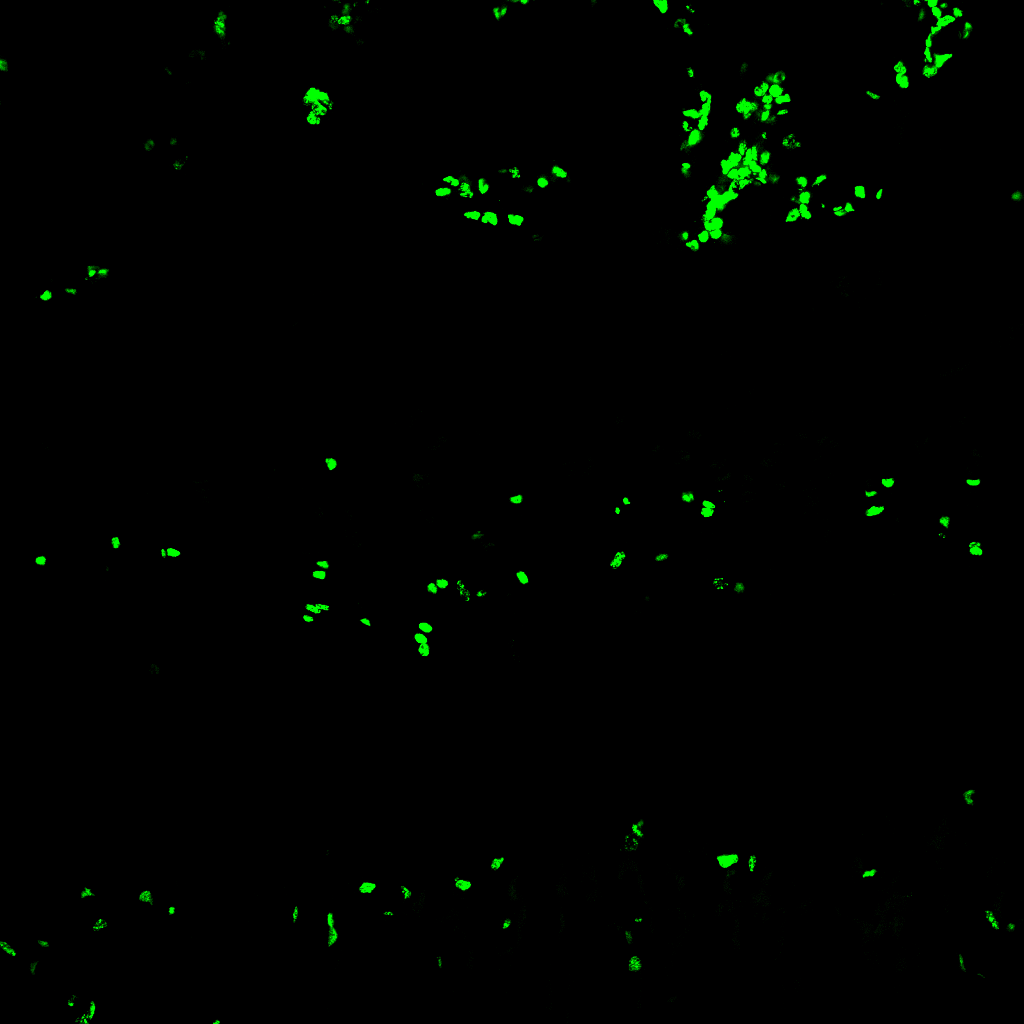

Supplement: Supplementary file 4 — Source Data Fig. 4 [file 44319_2024_93_MOESM4_ESM.zip › Figure4/4H/Veh_GP_20X_edu_green.tif]

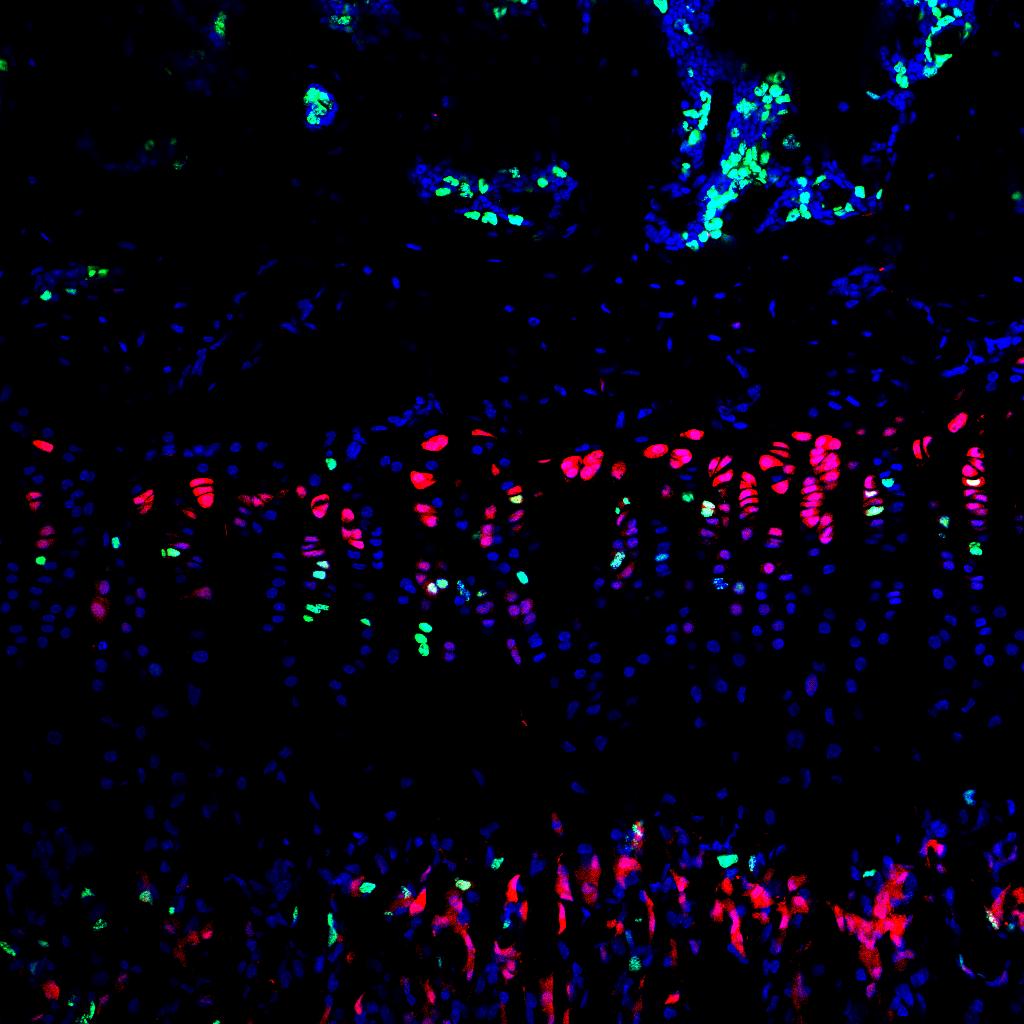

Supplement: Supplementary file 4 — Source Data Fig. 4 [file 44319_2024_93_MOESM4_ESM.zip › Figure4/4H/Veh_GP_20X_merge.tif]

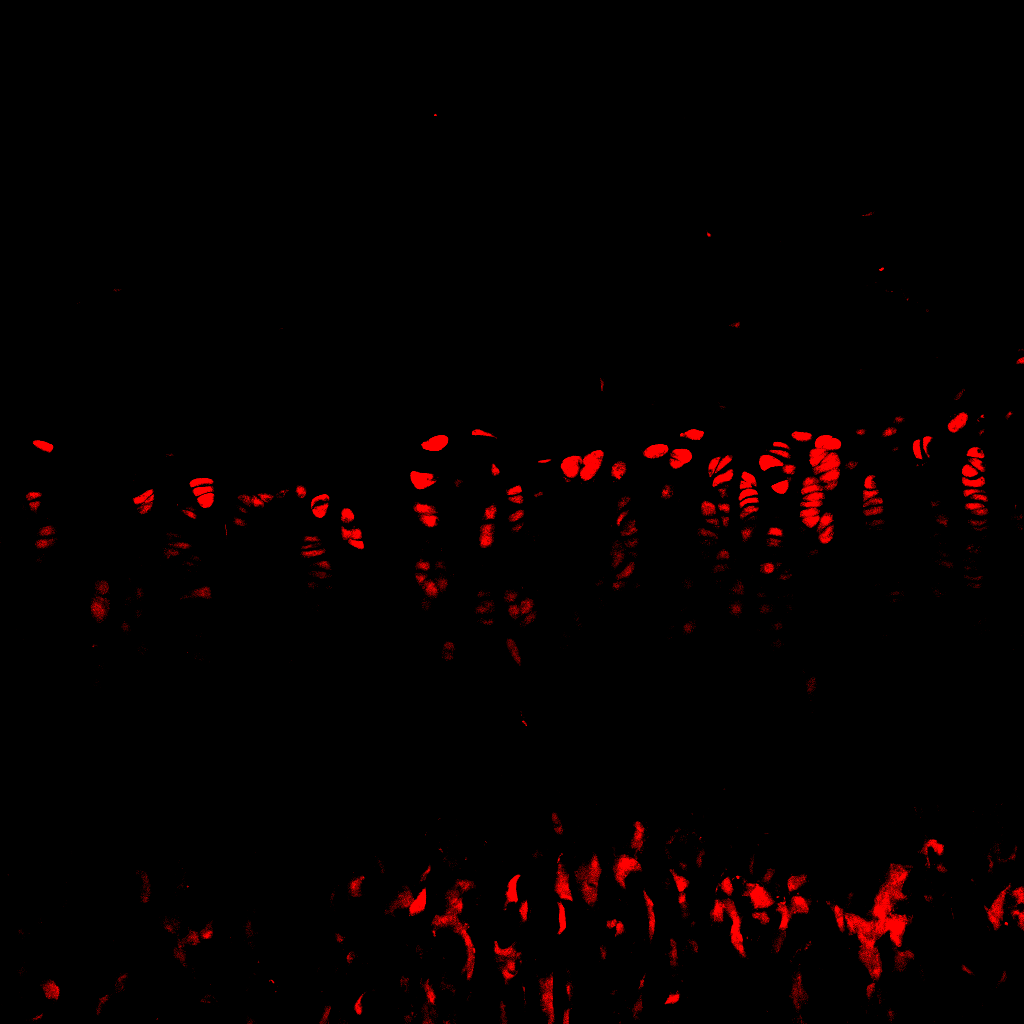

Supplement: Supplementary file 4 — Source Data Fig. 4 [file 44319_2024_93_MOESM4_ESM.zip › Figure4/4H/Veh_GP_20X_td_red.tif]

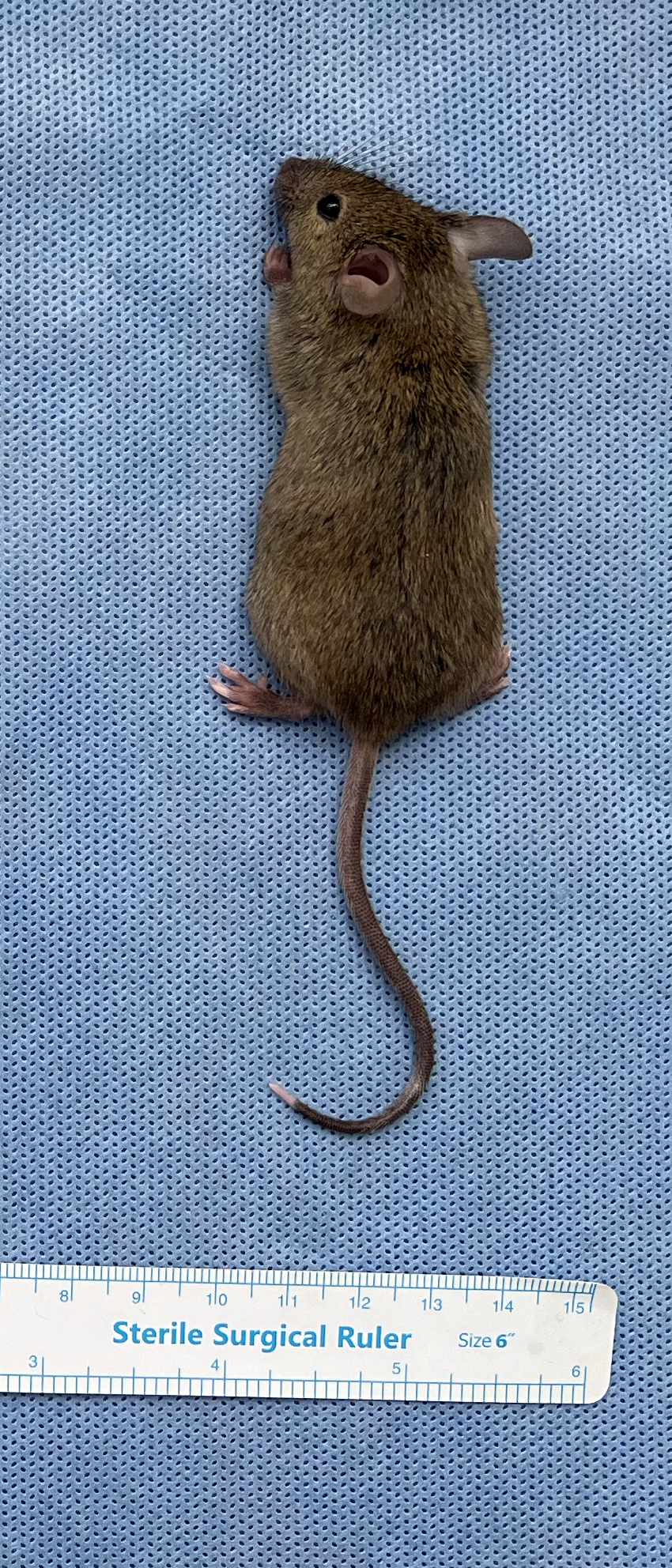

Supplement: Supplementary file 5 — Source Data Fig. 5 [file 44319_2024_93_MOESM5_ESM.zip › Figure5/5B/CKO.tif]

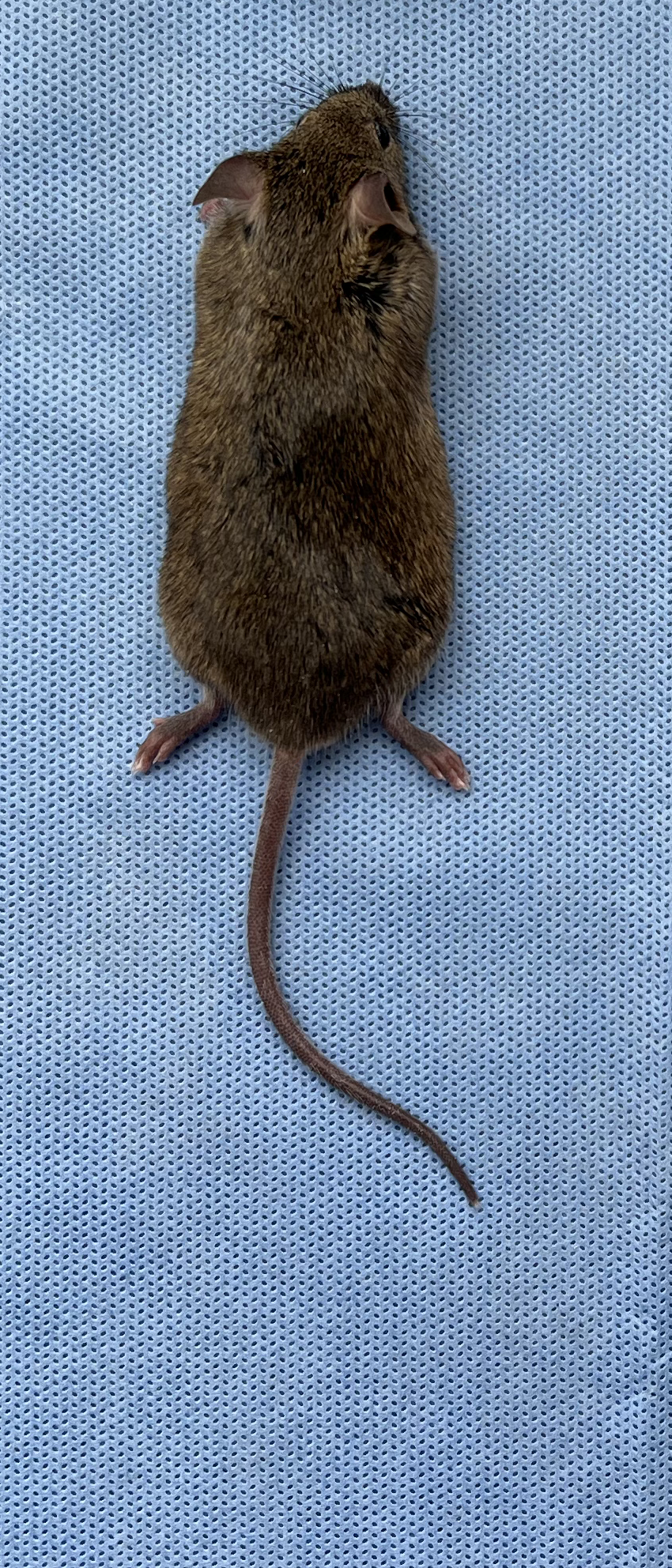

Supplement: Supplementary file 5 — Source Data Fig. 5 [file 44319_2024_93_MOESM5_ESM.zip › Figure5/5B/WT.tif]

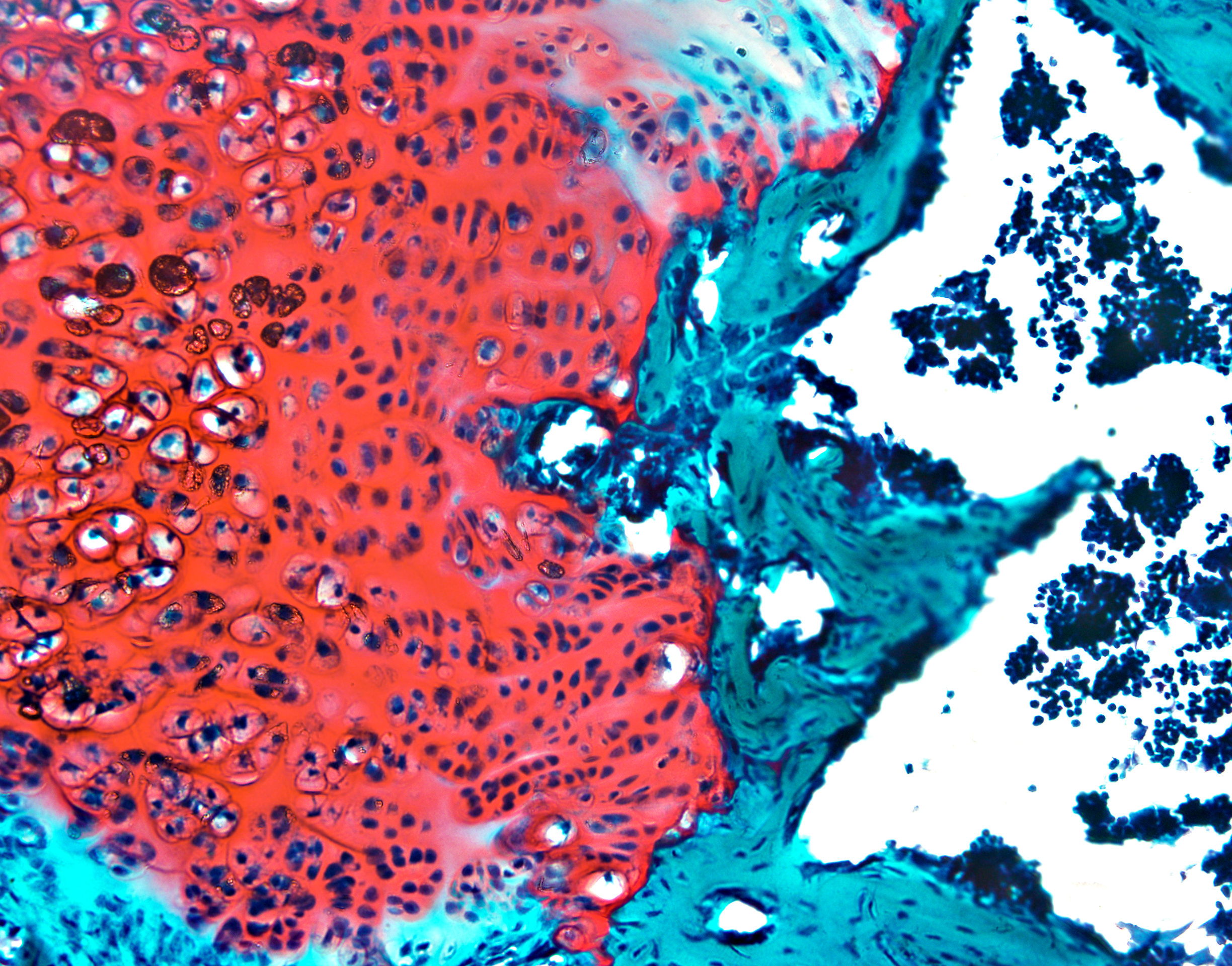

Supplement: Supplementary file 5 — Source Data Fig. 5 [file 44319_2024_93_MOESM5_ESM.zip › Figure5/5C/CKO_CC.tif]

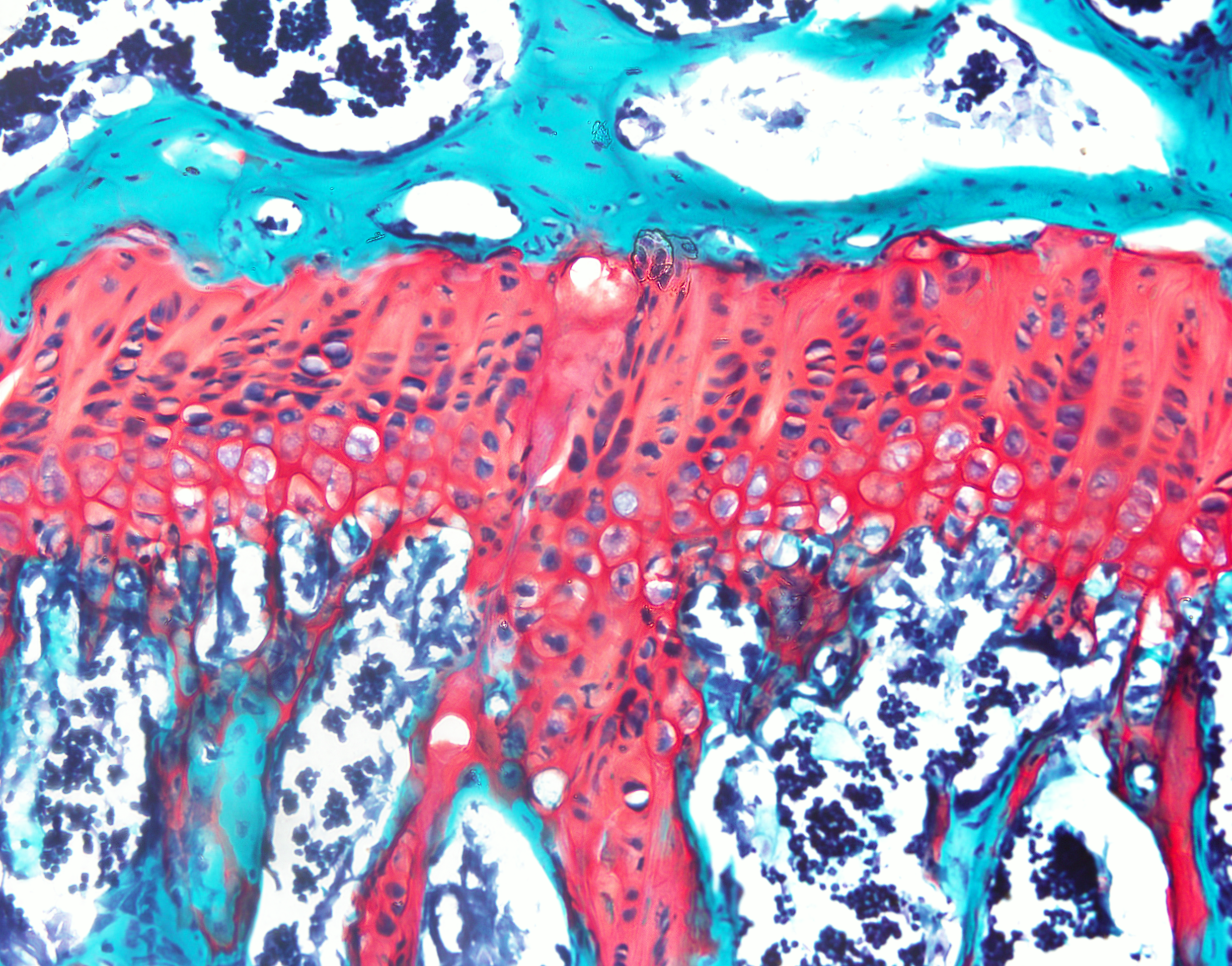

Supplement: Supplementary file 5 — Source Data Fig. 5 [file 44319_2024_93_MOESM5_ESM.zip › Figure5/5C/CKO_GP.tif]

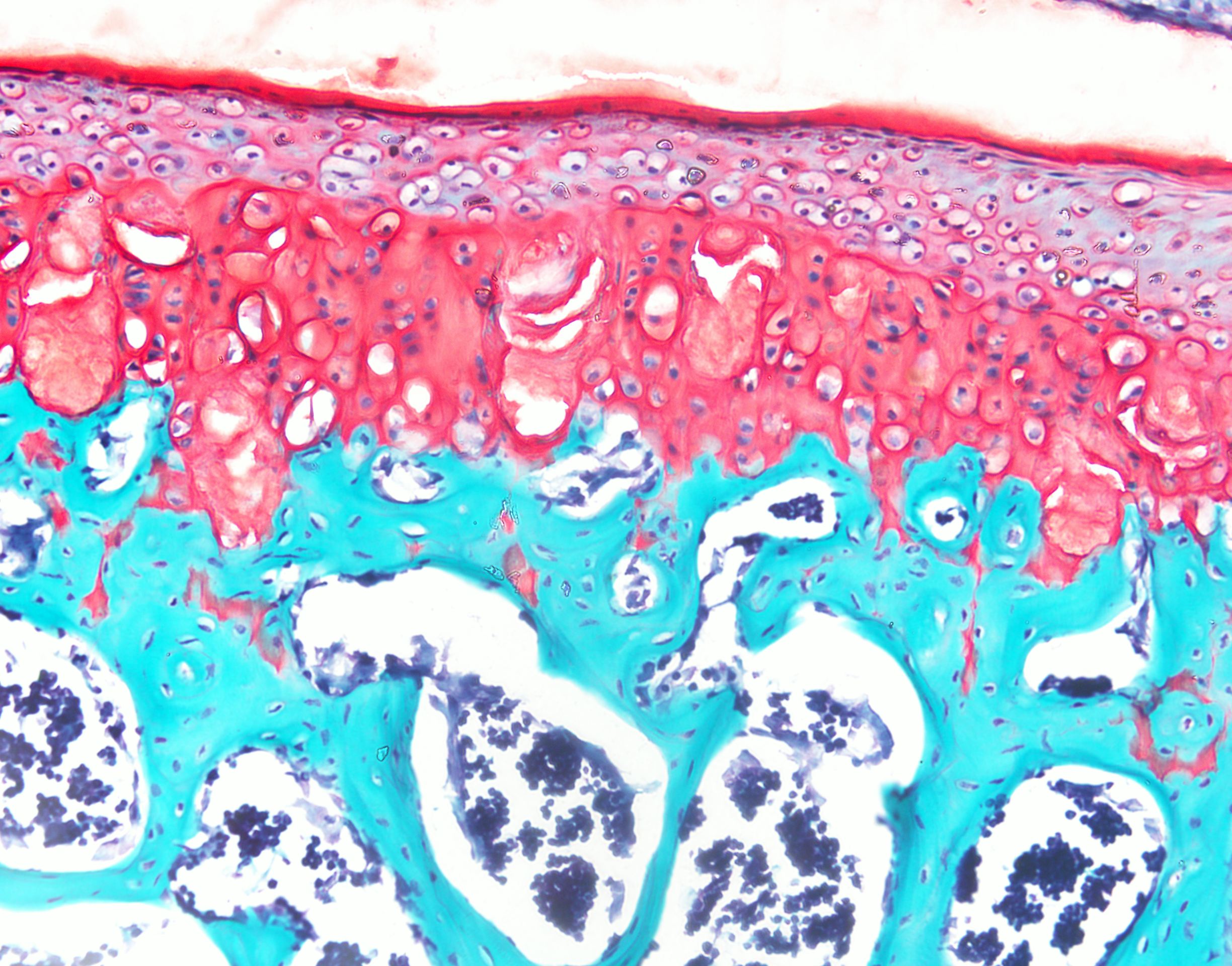

Supplement: Supplementary file 5 — Source Data Fig. 5 [file 44319_2024_93_MOESM5_ESM.zip › Figure5/5C/CKO_VB.tif]

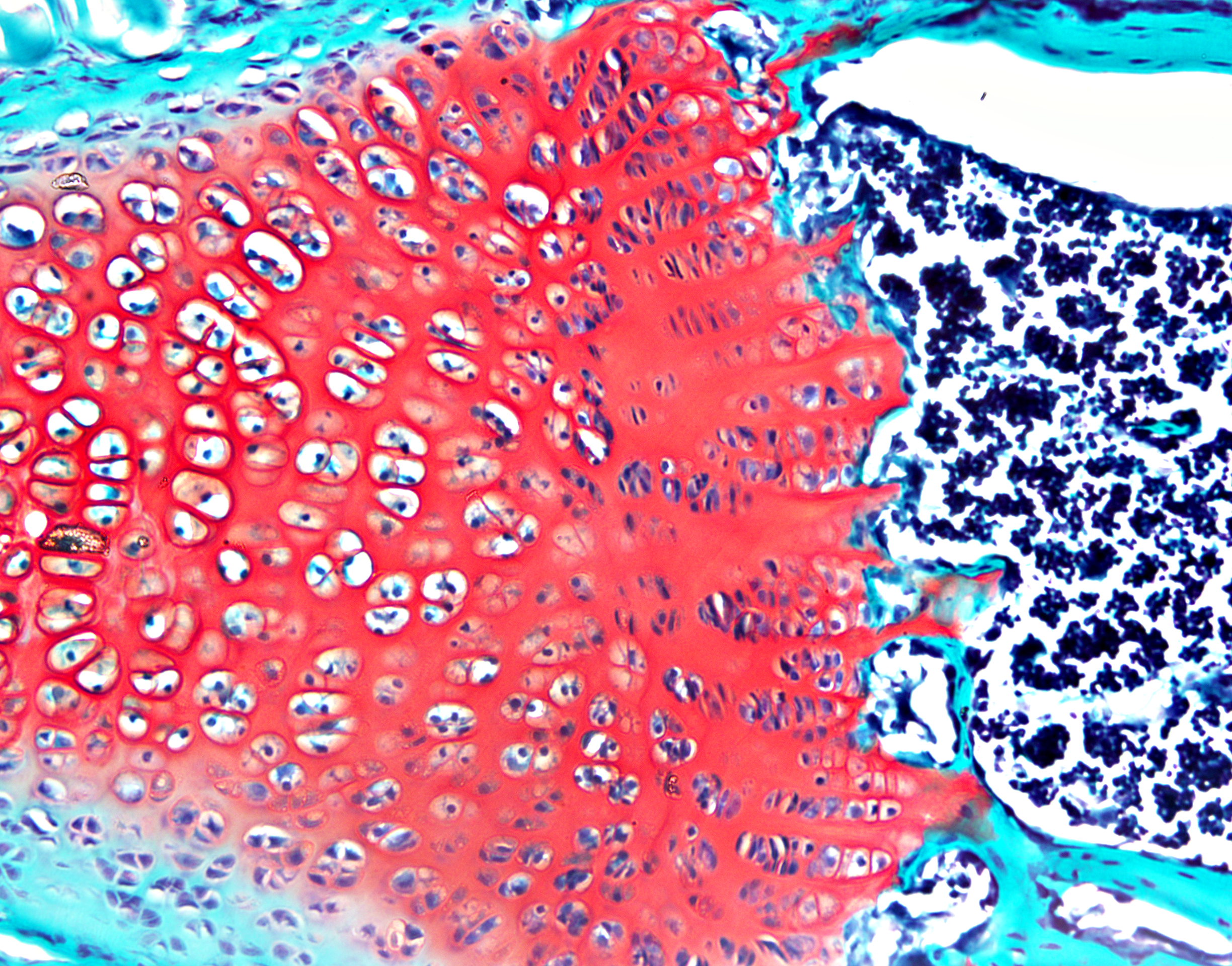

Supplement: Supplementary file 5 — Source Data Fig. 5 [file 44319_2024_93_MOESM5_ESM.zip › Figure5/5C/WT_CC.tif]

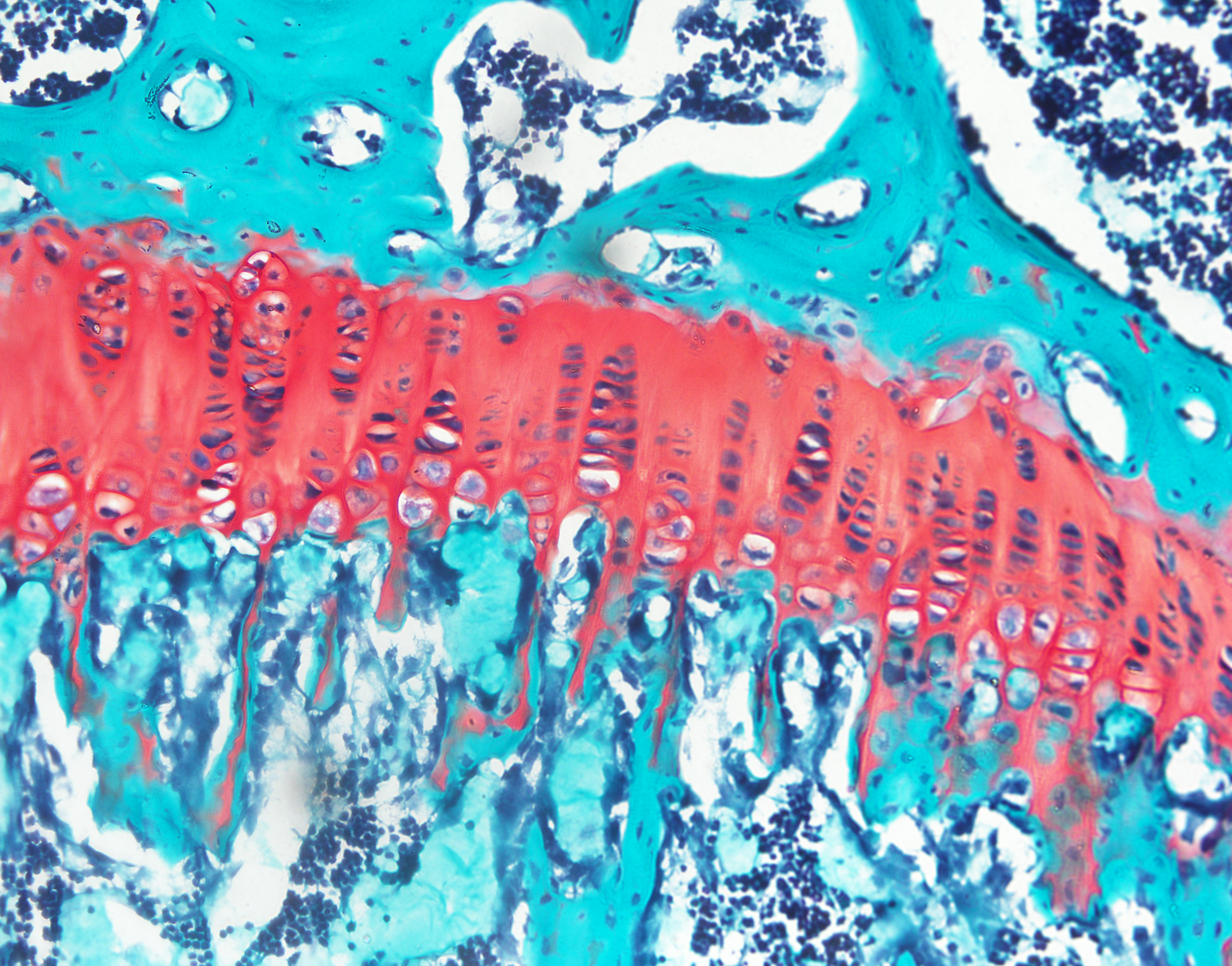

Supplement: Supplementary file 5 — Source Data Fig. 5 [file 44319_2024_93_MOESM5_ESM.zip › Figure5/5C/WT_GP.tif]

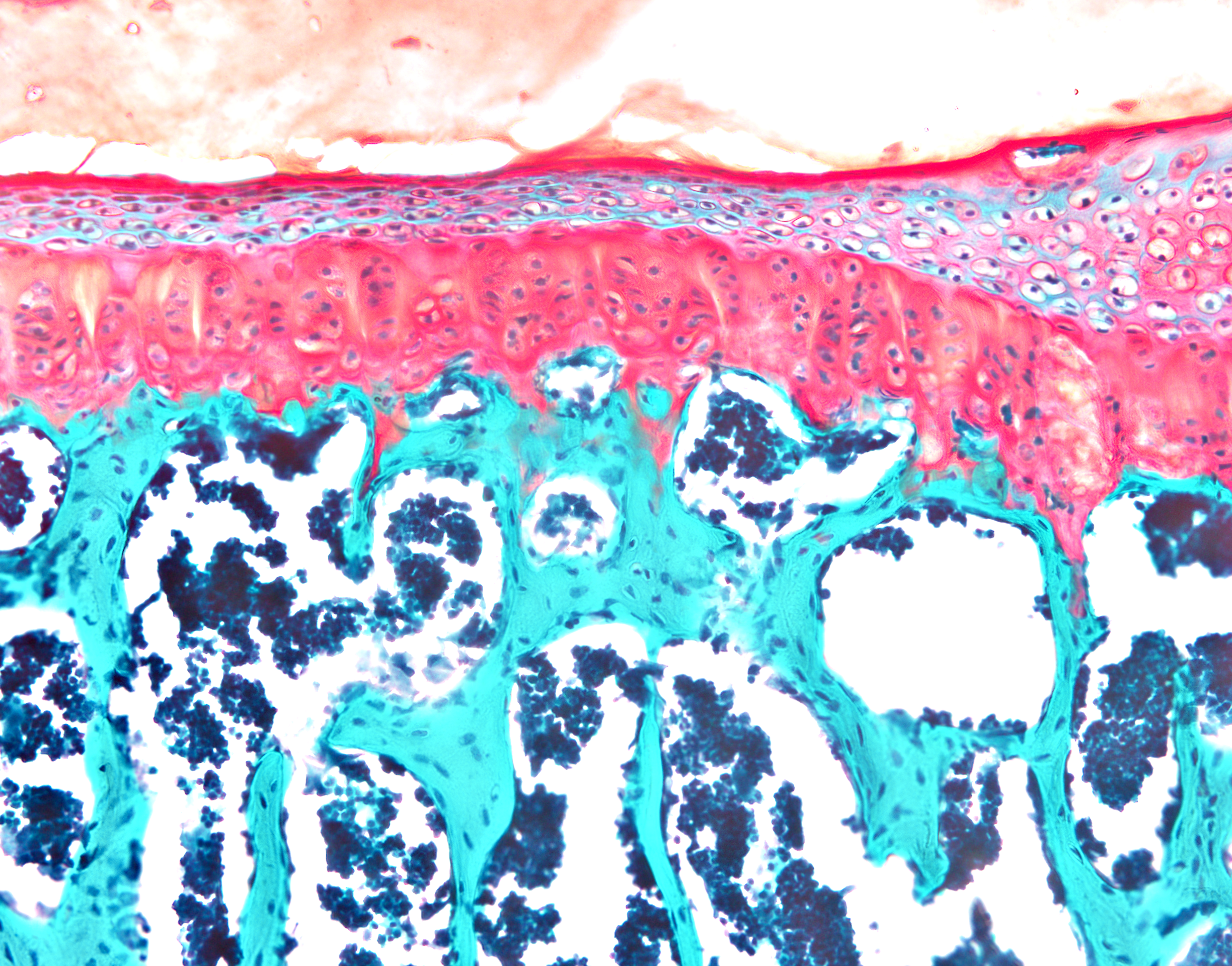

Supplement: Supplementary file 5 — Source Data Fig. 5 [file 44319_2024_93_MOESM5_ESM.zip › Figure5/5C/WT_VB.tif]

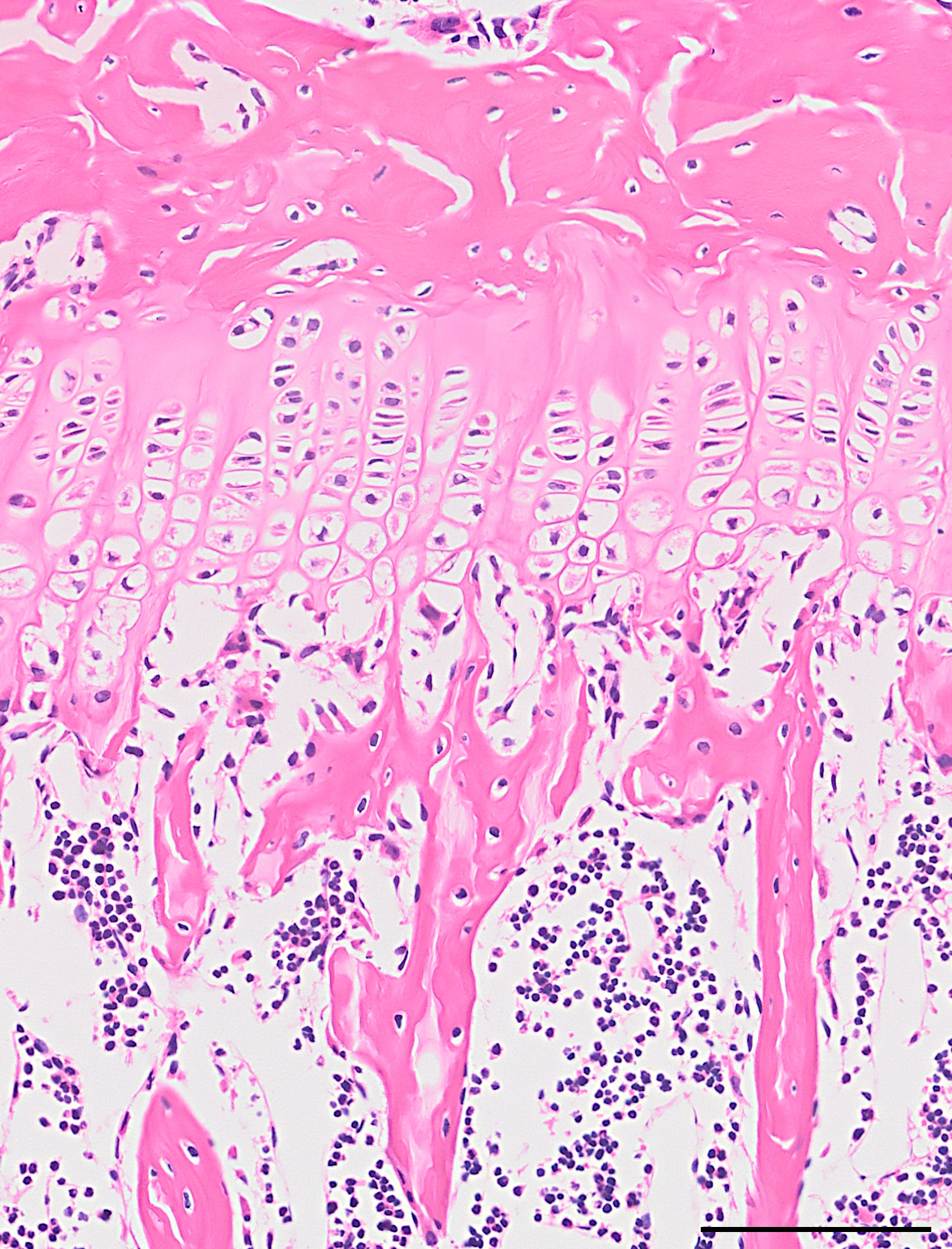

Supplement: Supplementary file 5 — Source Data Fig. 5 [file 44319_2024_93_MOESM5_ESM.zip › Figure5/5D/WT.tif]

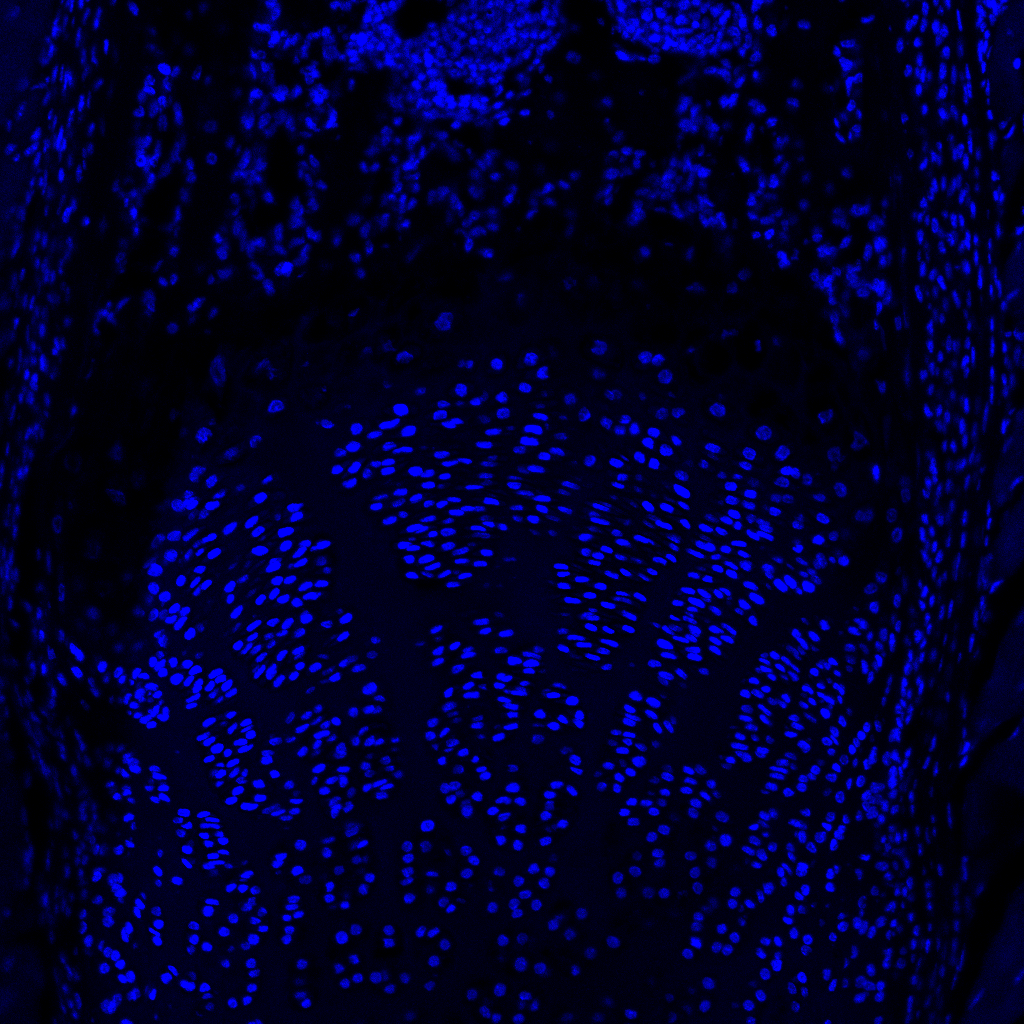

Supplement: Supplementary file 5 — Source Data Fig. 5 [file 44319_2024_93_MOESM5_ESM.zip › Figure5/5F/TM1M_1M_CKO_CC_dapi_blue.tif]

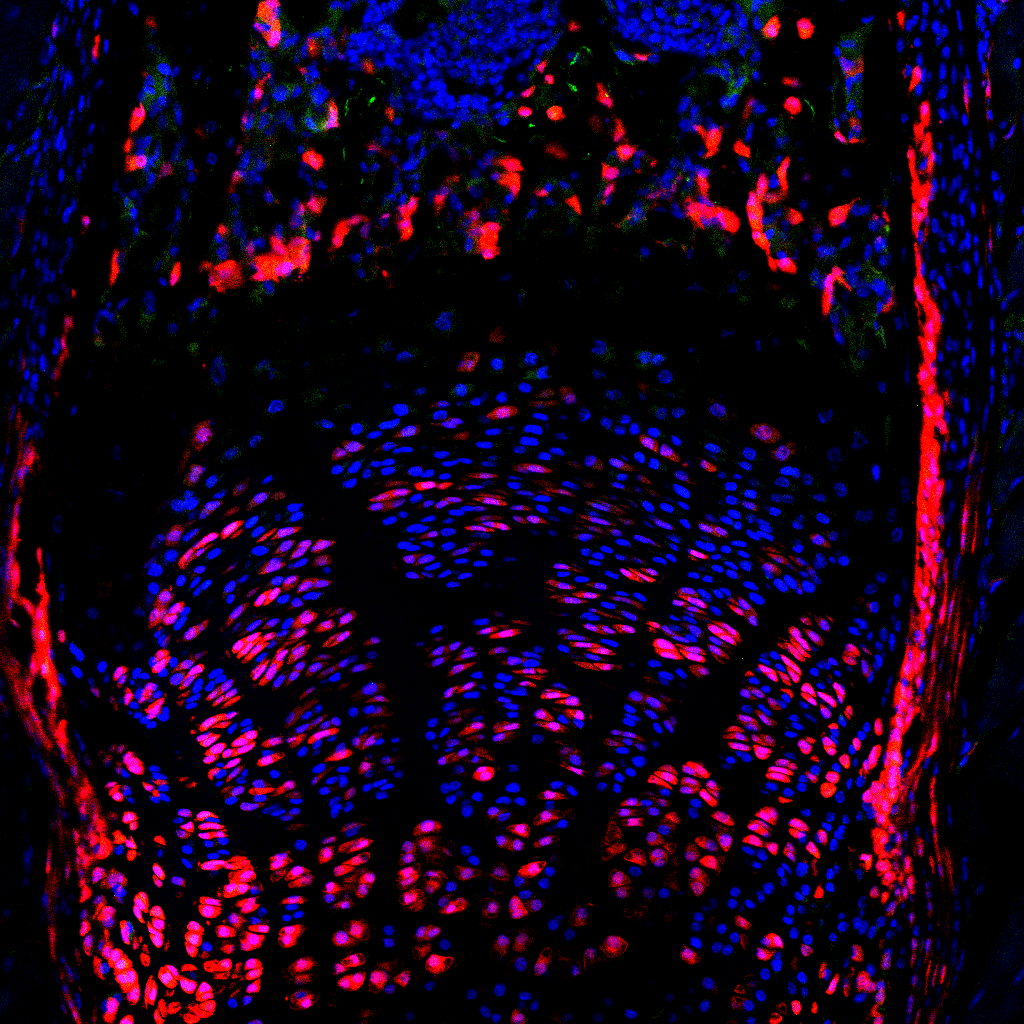

Supplement: Supplementary file 5 — Source Data Fig. 5 [file 44319_2024_93_MOESM5_ESM.zip › Figure5/5F/TM1M_1M_CKO_CC_merge.tif]

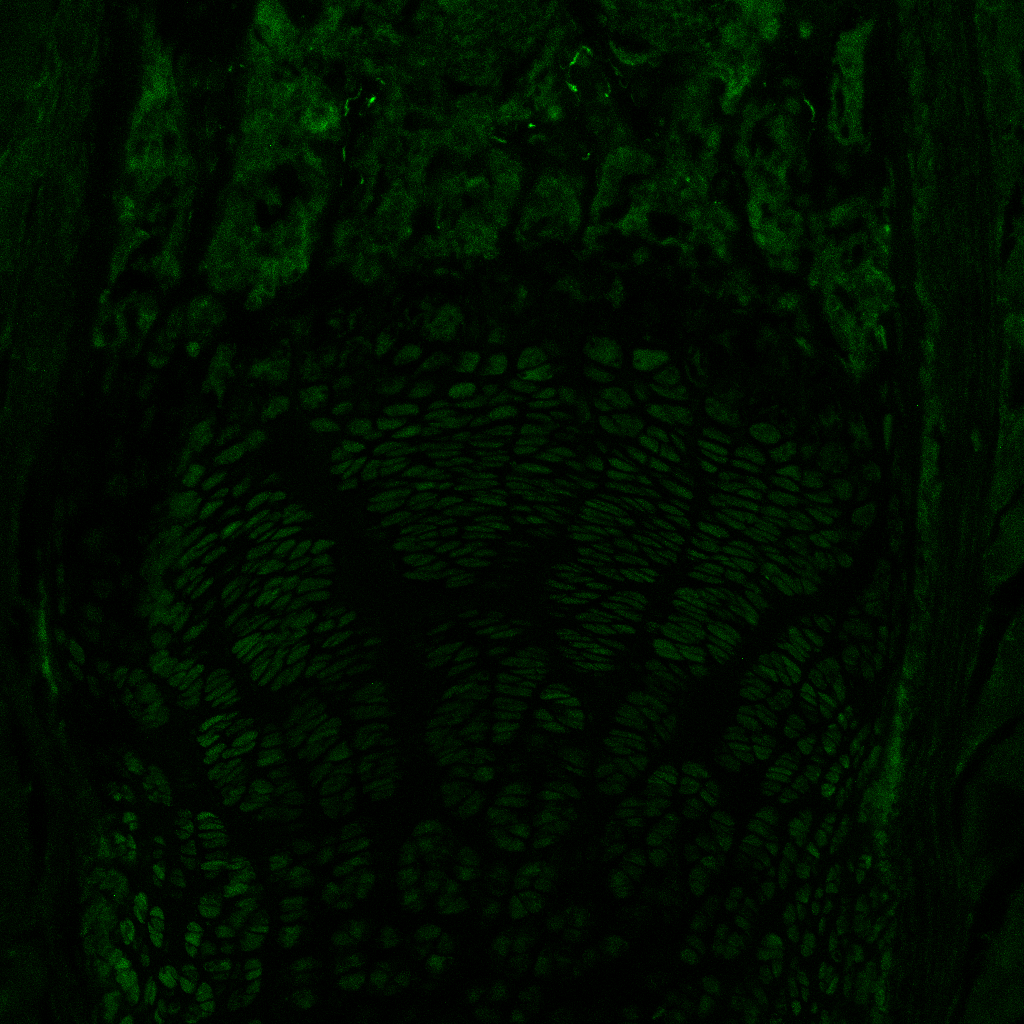

Supplement: Supplementary file 5 — Source Data Fig. 5 [file 44319_2024_93_MOESM5_ESM.zip › Figure5/5F/TM1M_1M_CKO_CC_psmad_green.tif]

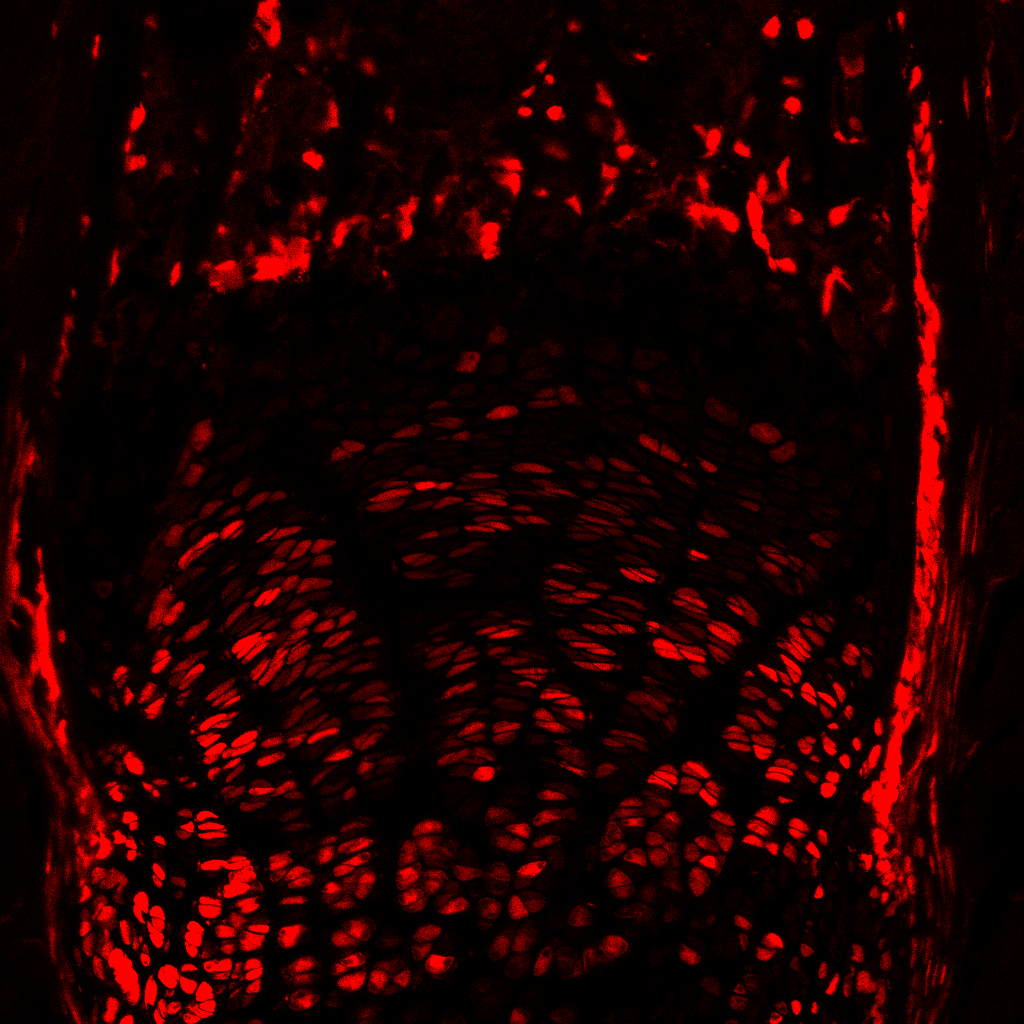

Supplement: Supplementary file 5 — Source Data Fig. 5 [file 44319_2024_93_MOESM5_ESM.zip › Figure5/5F/TM1M_1M_CKO_CC_td_red.tif]

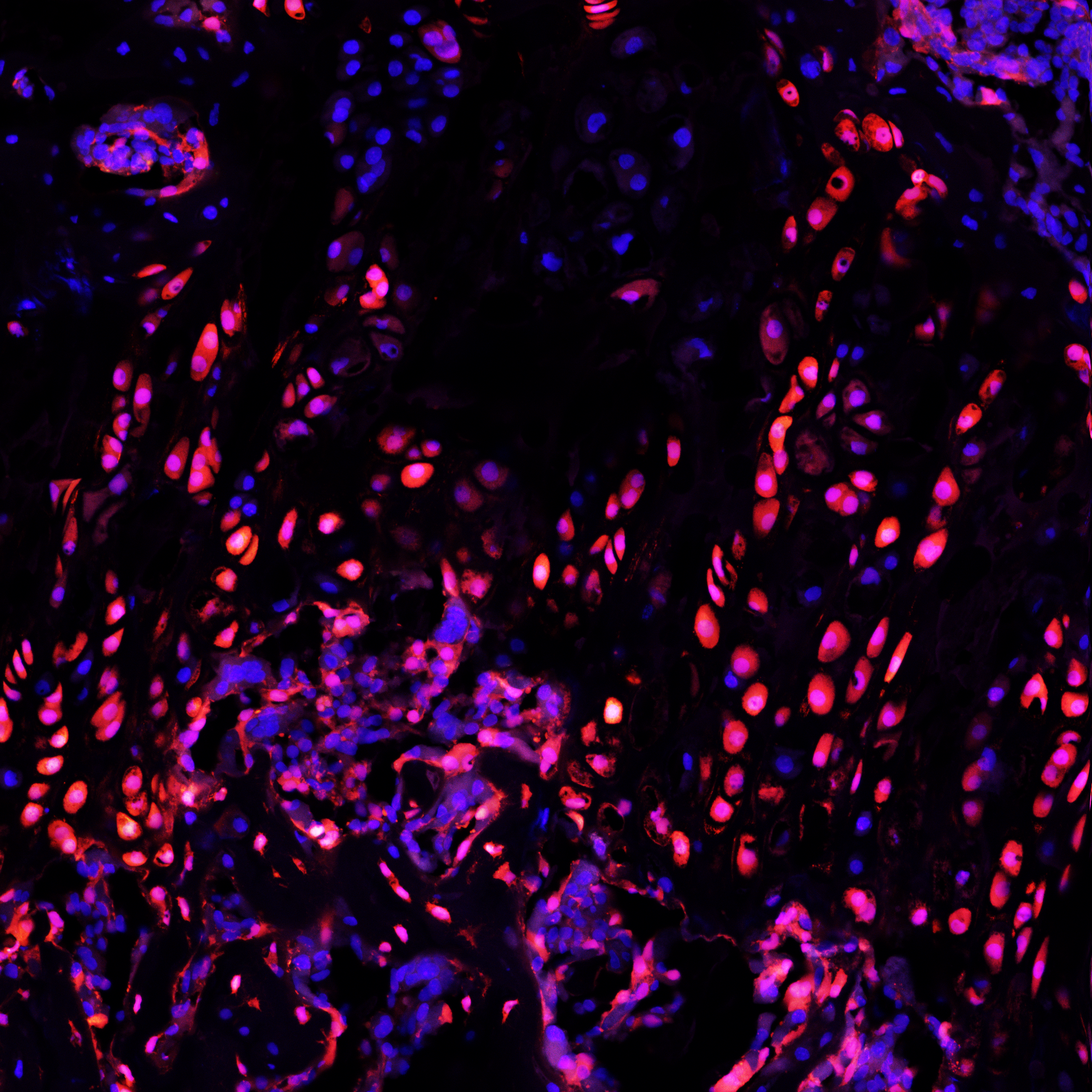

Supplement: Supplementary file 5 — Source Data Fig. 5 [file 44319_2024_93_MOESM5_ESM.zip › Figure5/5F/TM1M_1M_CKO_GP_merge.tif]

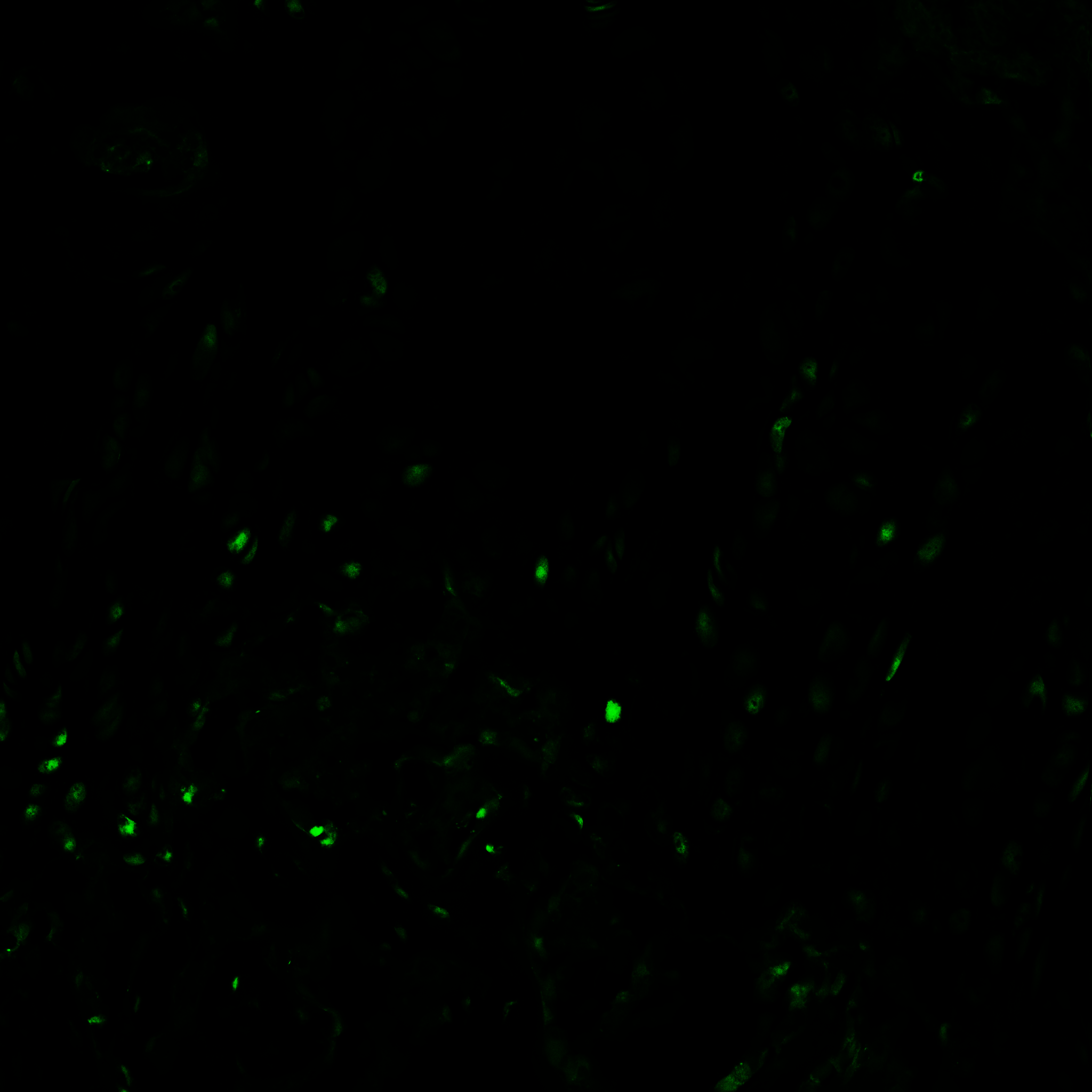

Supplement: Supplementary file 5 — Source Data Fig. 5 [file 44319_2024_93_MOESM5_ESM.zip › Figure5/5F/TM1M_1M_CKO_GP_psmad_green.tif]

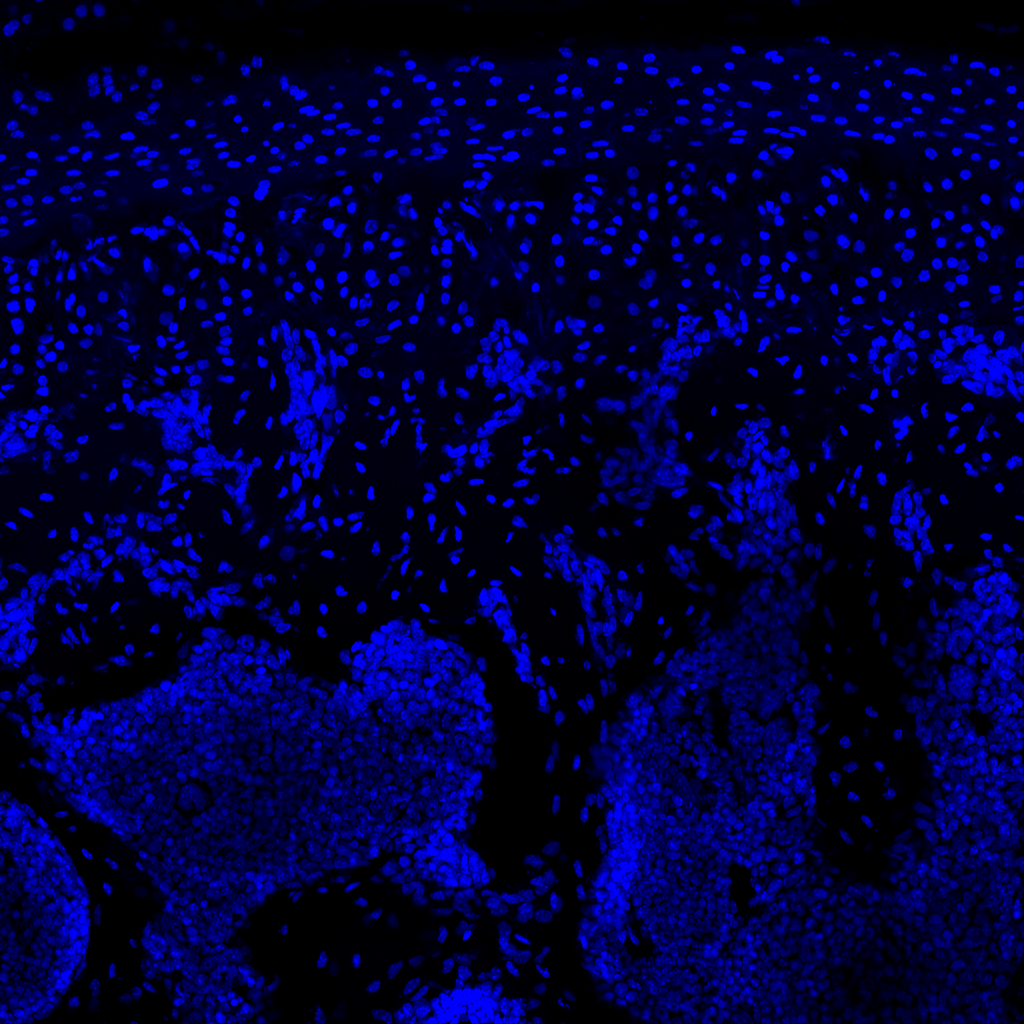

Supplement: Supplementary file 5 — Source Data Fig. 5 [file 44319_2024_93_MOESM5_ESM.zip › Figure5/5F/TM1M_1M_CKO_VB_dapi_blue.tif]

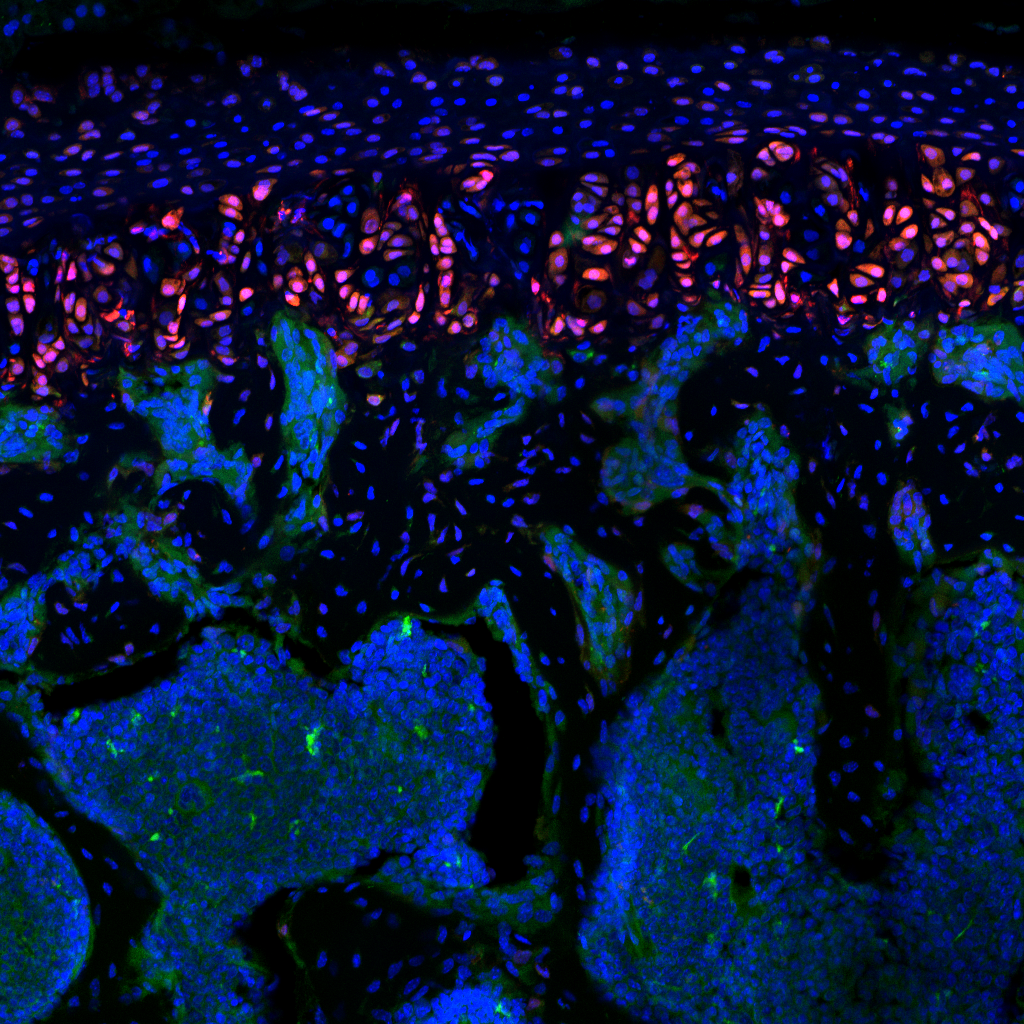

Supplement: Supplementary file 5 — Source Data Fig. 5 [file 44319_2024_93_MOESM5_ESM.zip › Figure5/5F/TM1M_1M_CKO_VB_merge_.tif]

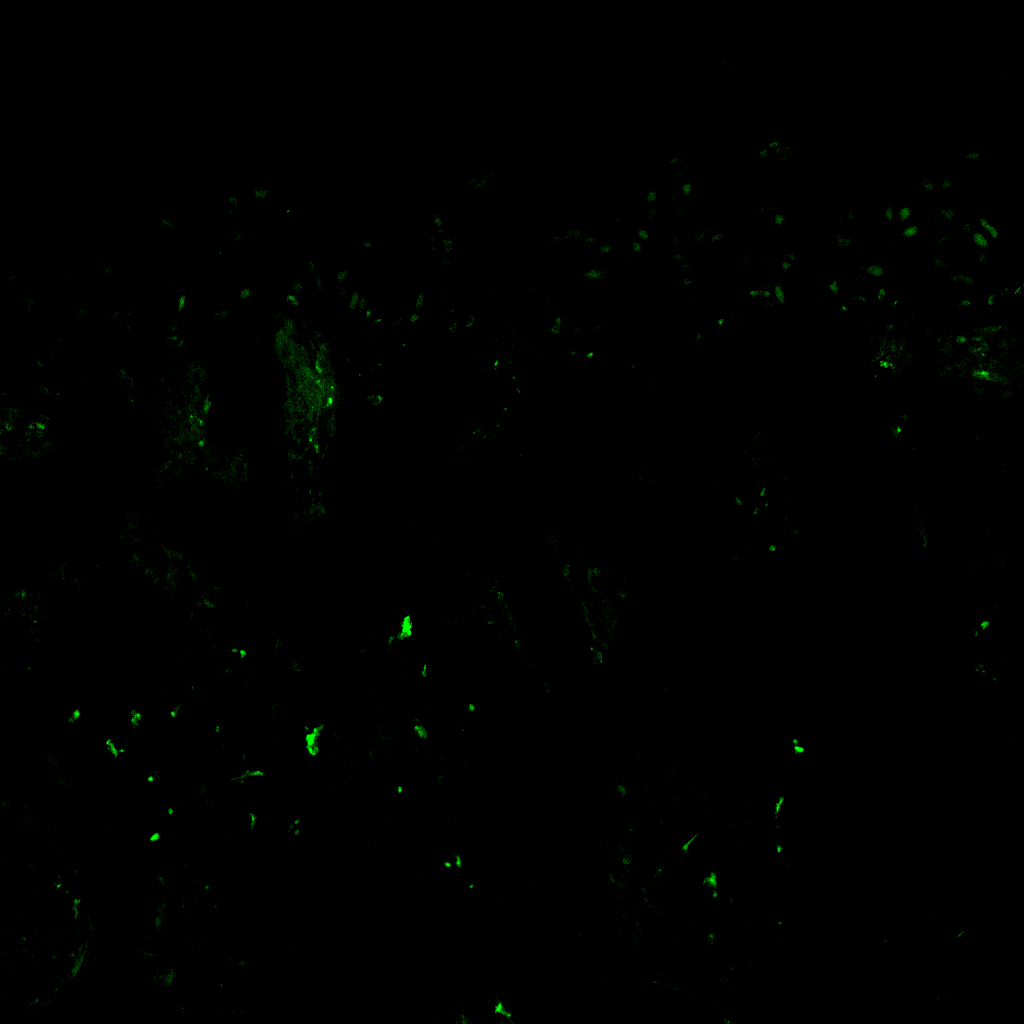

Supplement: Supplementary file 5 — Source Data Fig. 5 [file 44319_2024_93_MOESM5_ESM.zip › Figure5/5F/TM1M_1M_CKO_VB_psmad_green_.tif]

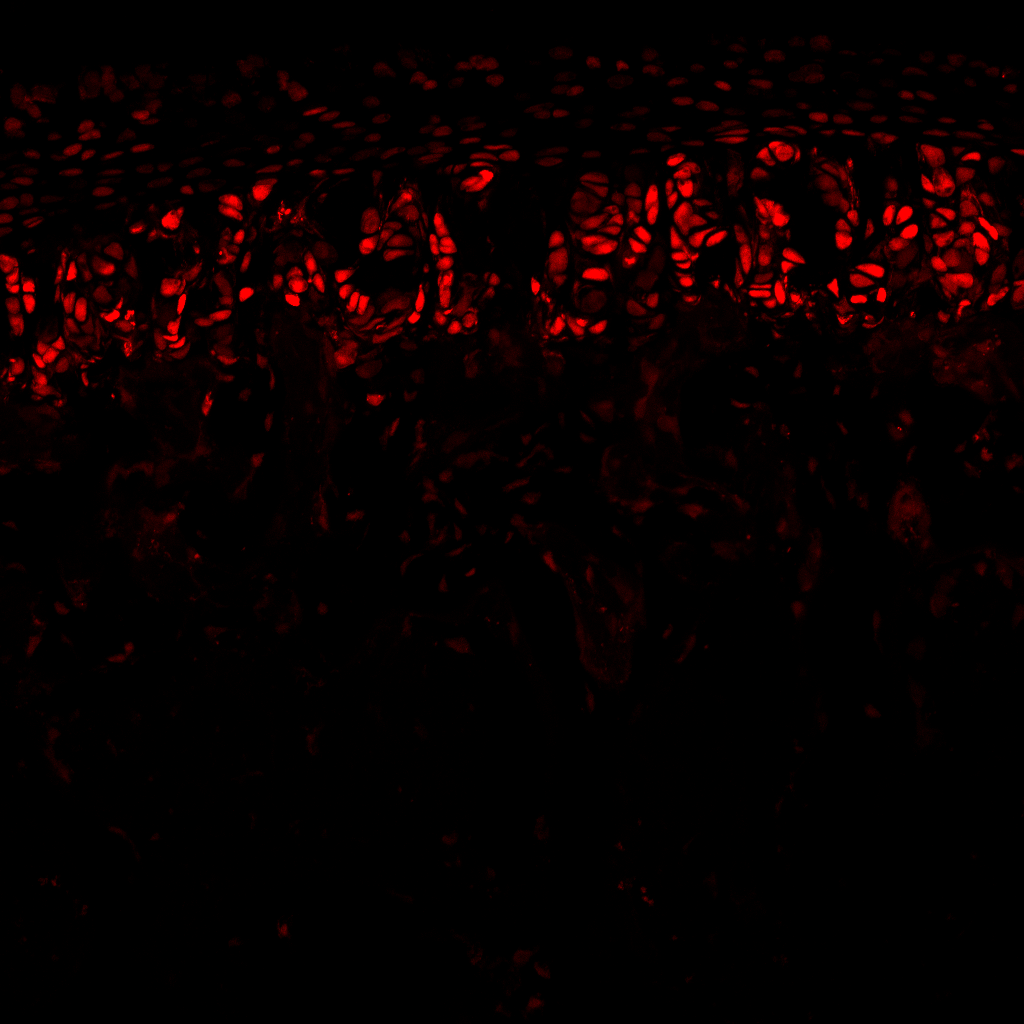

Supplement: Supplementary file 5 — Source Data Fig. 5 [file 44319_2024_93_MOESM5_ESM.zip › Figure5/5F/TM1M_1M_CKO_VB_td_red_.tif]

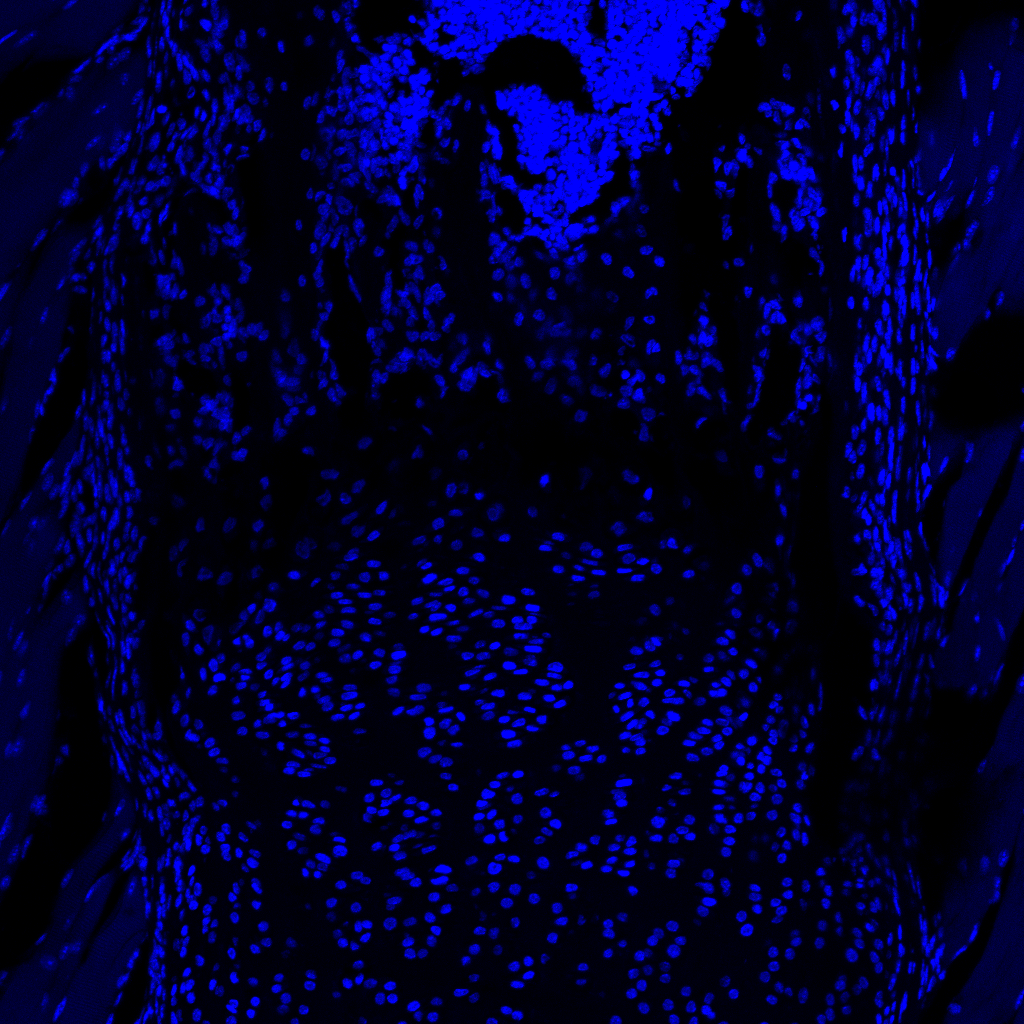

Supplement: Supplementary file 5 — Source Data Fig. 5 [file 44319_2024_93_MOESM5_ESM.zip › Figure5/5F/TM1M_1M_WT_CC_dapi_blue.tif]

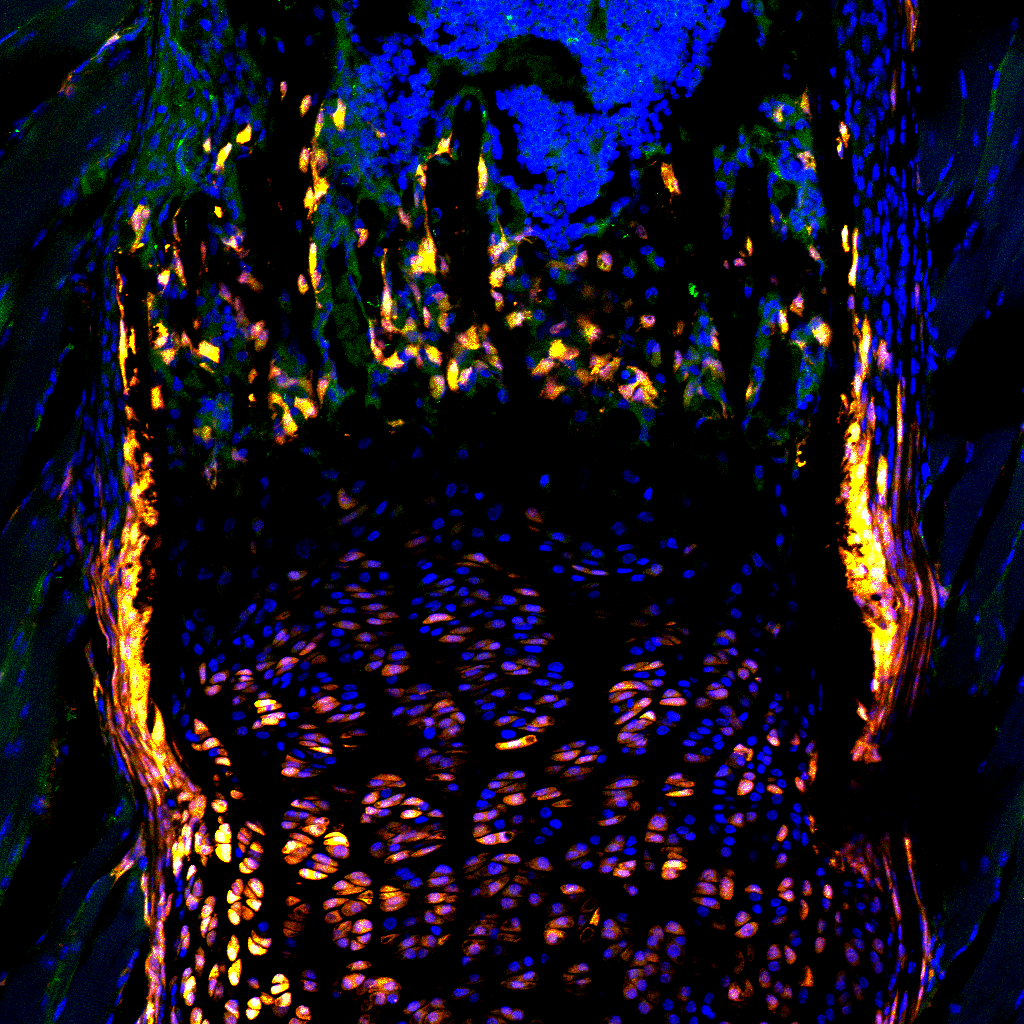

Supplement: Supplementary file 5 — Source Data Fig. 5 [file 44319_2024_93_MOESM5_ESM.zip › Figure5/5F/TM1M_1M_WT_CC_merge.tif]

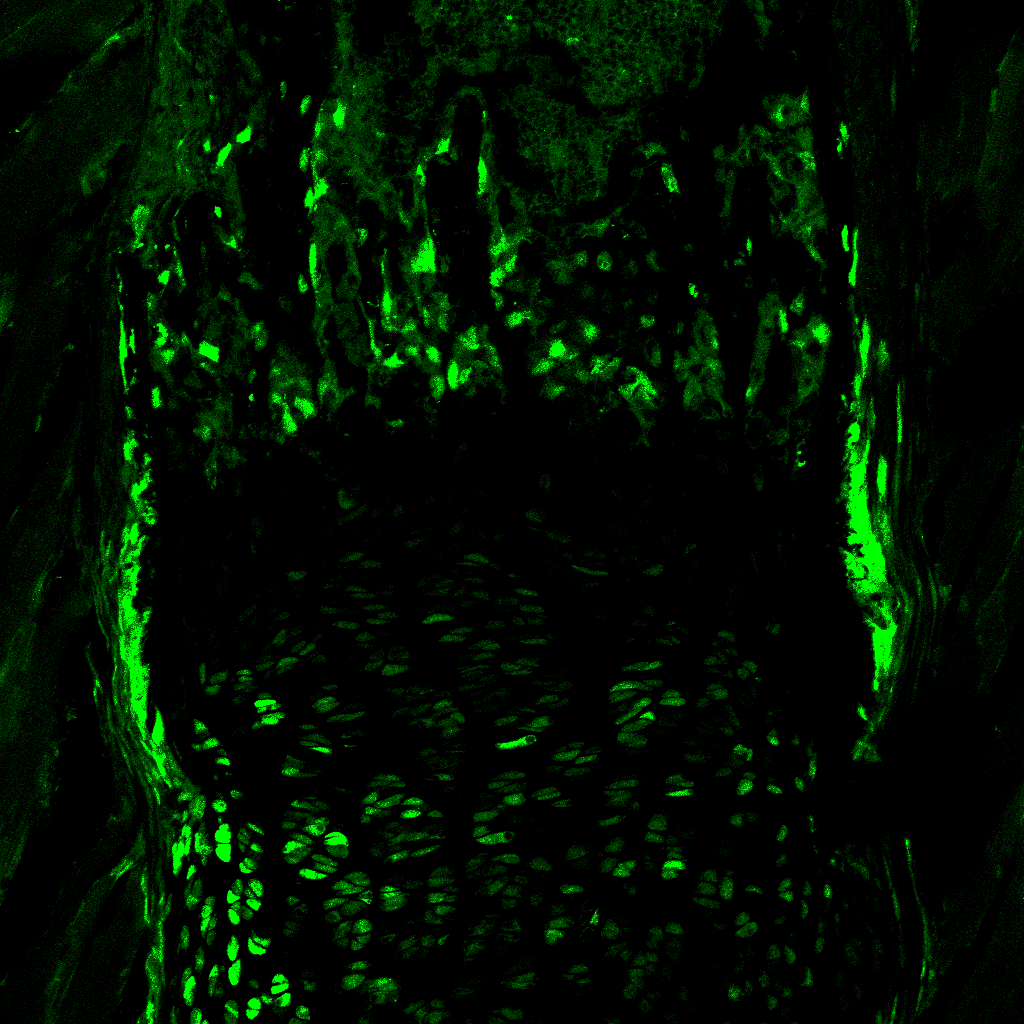

Supplement: Supplementary file 5 — Source Data Fig. 5 [file 44319_2024_93_MOESM5_ESM.zip › Figure5/5F/TM1M_1M_WT_CC_psmad_green.tif]

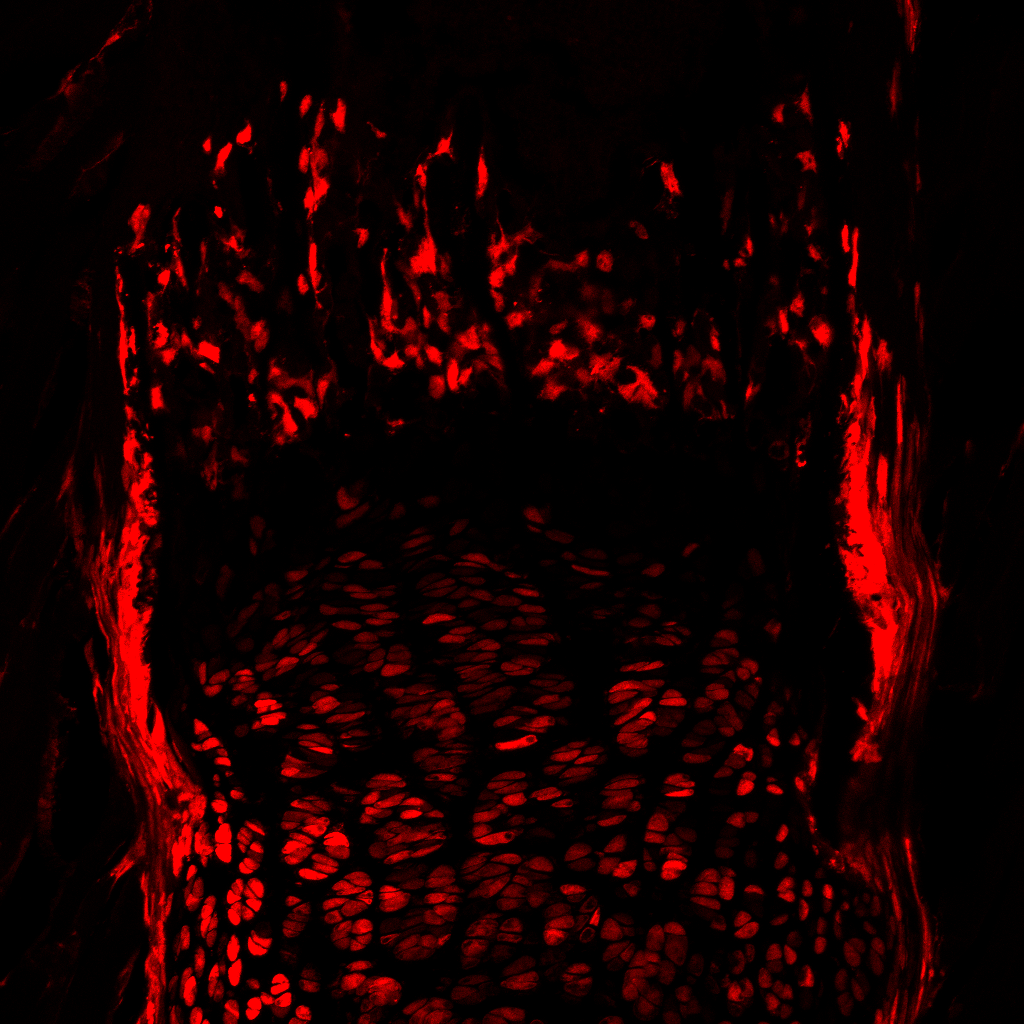

Supplement: Supplementary file 5 — Source Data Fig. 5 [file 44319_2024_93_MOESM5_ESM.zip › Figure5/5F/TM1M_1M_WT_CC_td_red.tif]

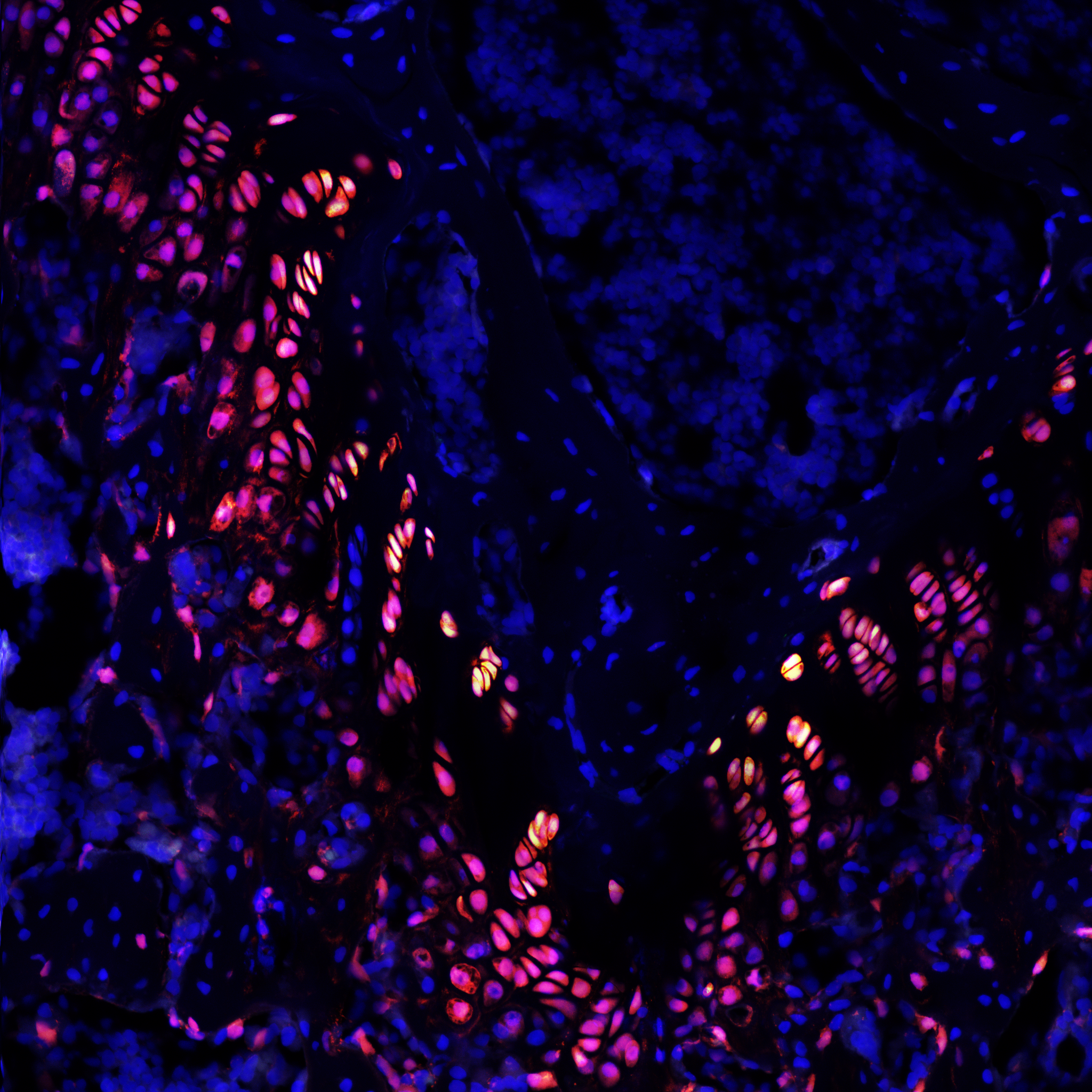

Supplement: Supplementary file 5 — Source Data Fig. 5 [file 44319_2024_93_MOESM5_ESM.zip › Figure5/5F/TM1M_1M_WT_GP_merge.tif]

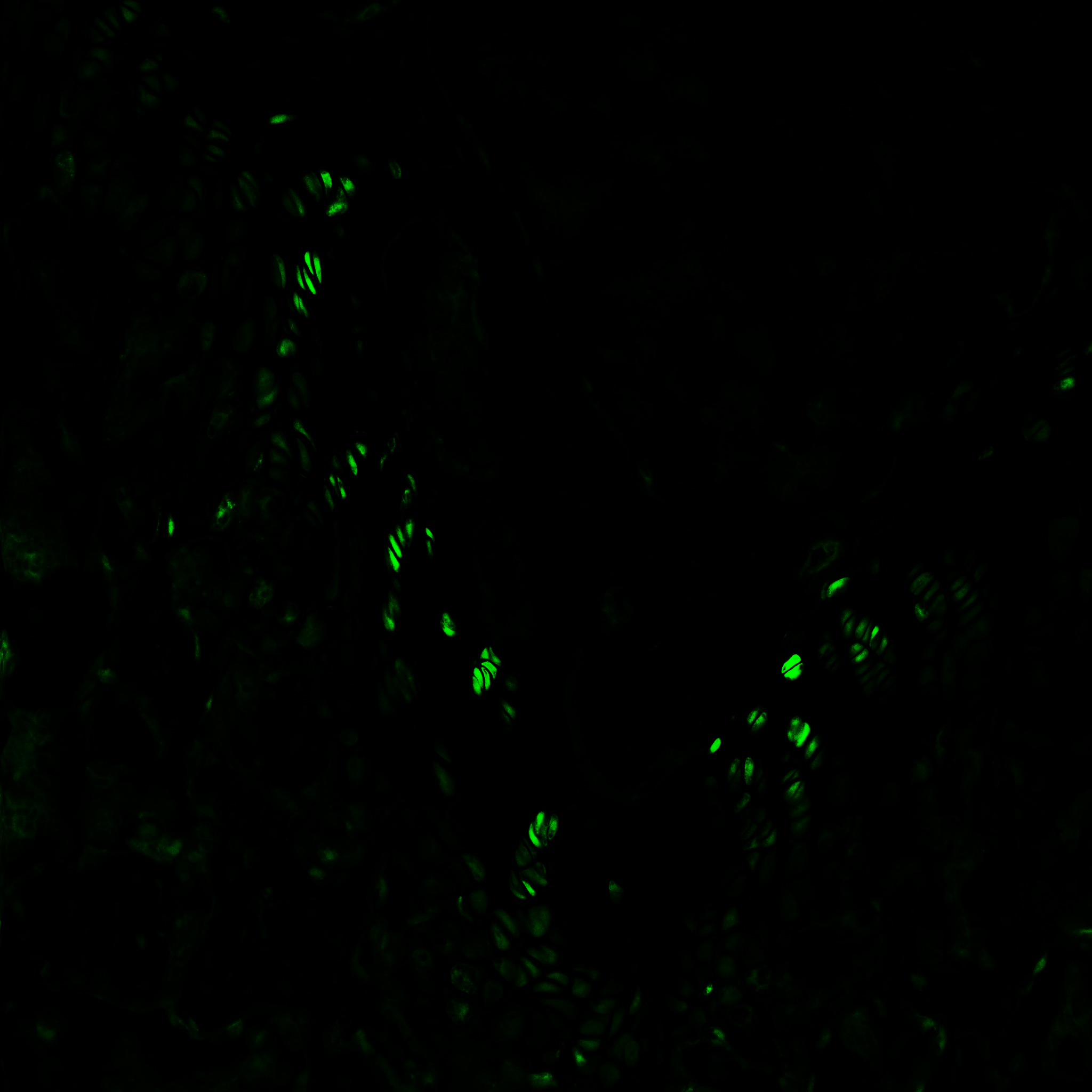

Supplement: Supplementary file 5 — Source Data Fig. 5 [file 44319_2024_93_MOESM5_ESM.zip › Figure5/5F/TM1M_1M_WT_GP_psmad_green.tif]

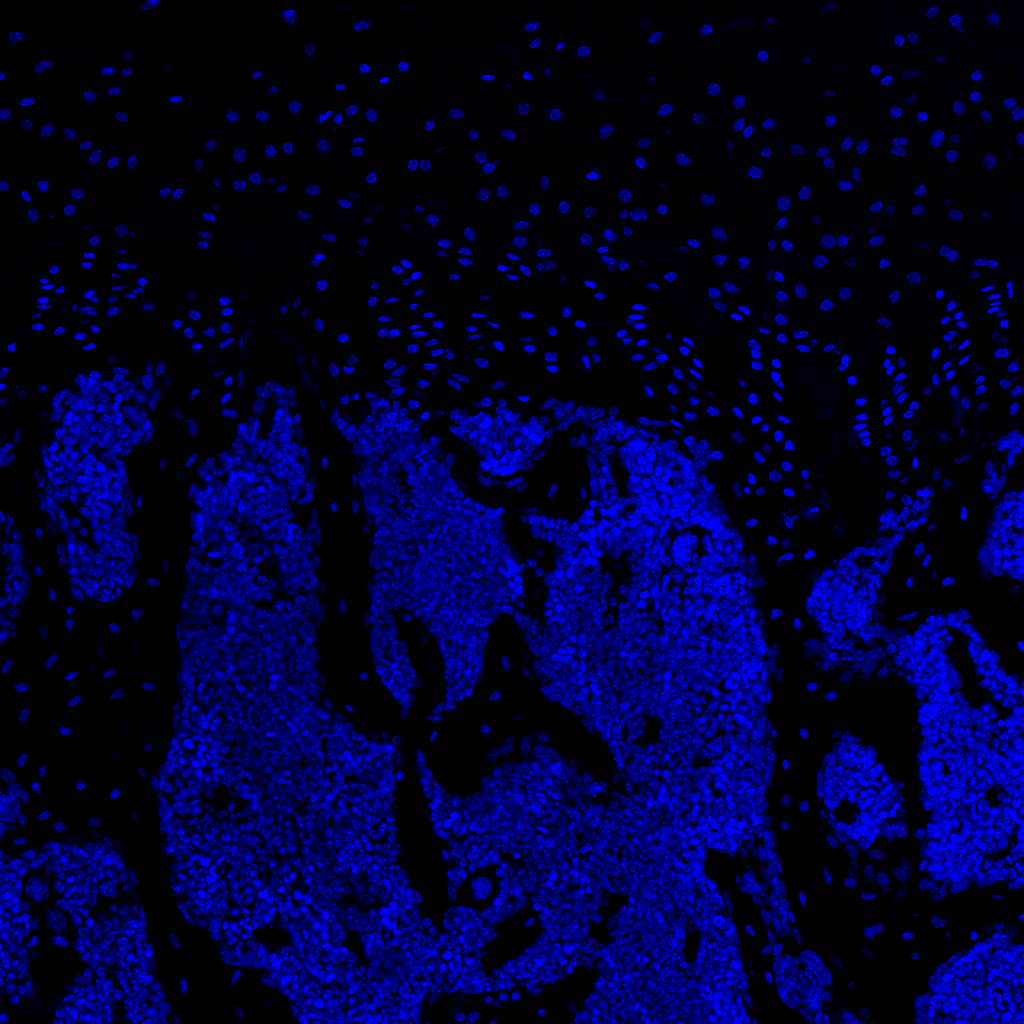

Supplement: Supplementary file 5 — Source Data Fig. 5 [file 44319_2024_93_MOESM5_ESM.zip › Figure5/5F/TM1M_1M_WT_VB_dapi_blue.tif]

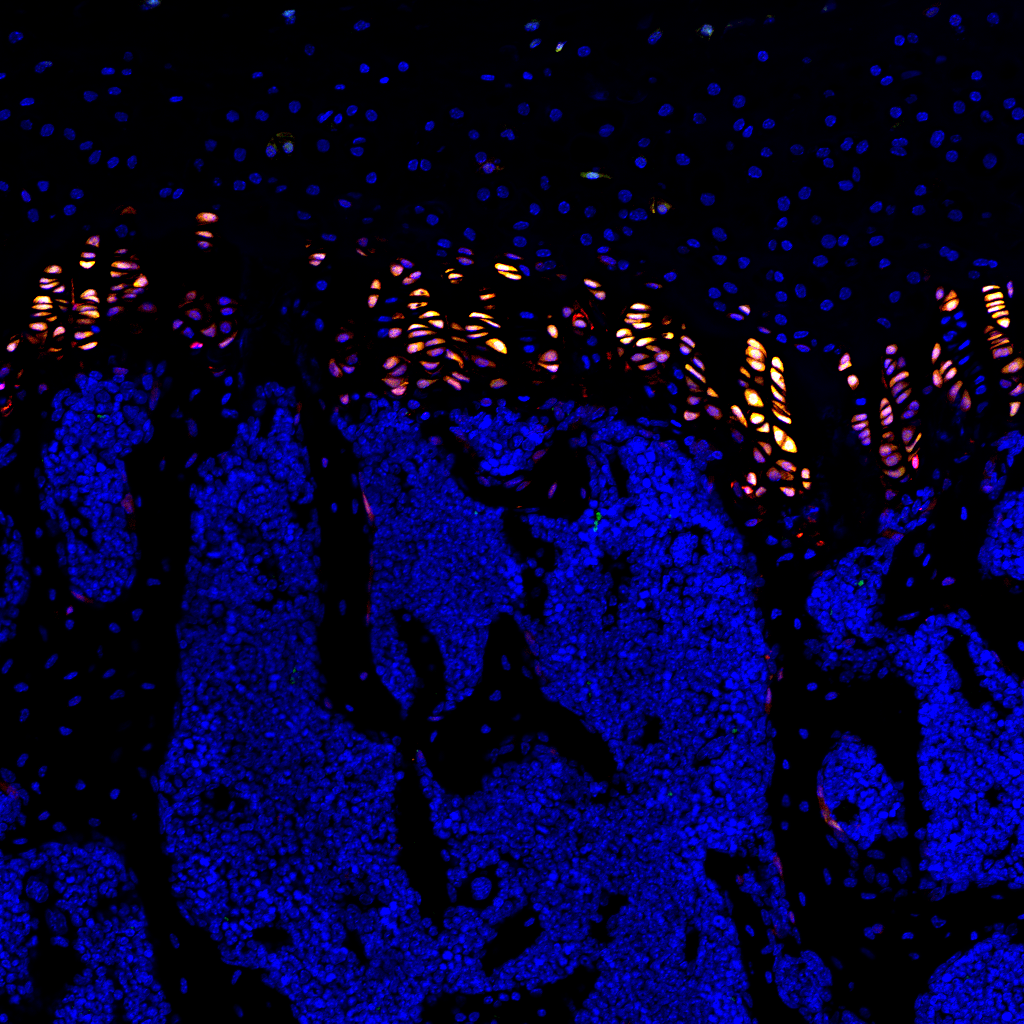

Supplement: Supplementary file 5 — Source Data Fig. 5 [file 44319_2024_93_MOESM5_ESM.zip › Figure5/5F/TM1M_1M_WT_VB_merge.tif]

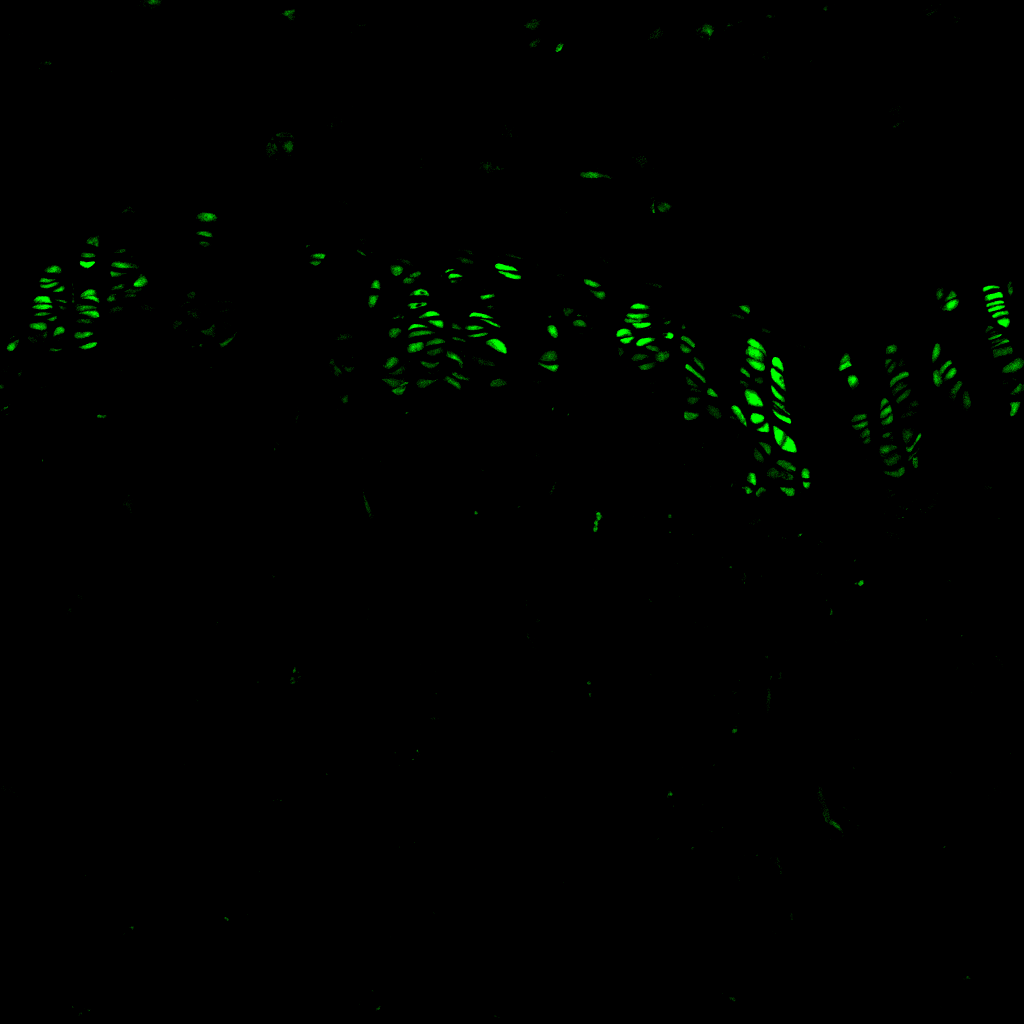

Supplement: Supplementary file 5 — Source Data Fig. 5 [file 44319_2024_93_MOESM5_ESM.zip › Figure5/5F/TM1M_1M_WT_VB_psmad_green.tif]

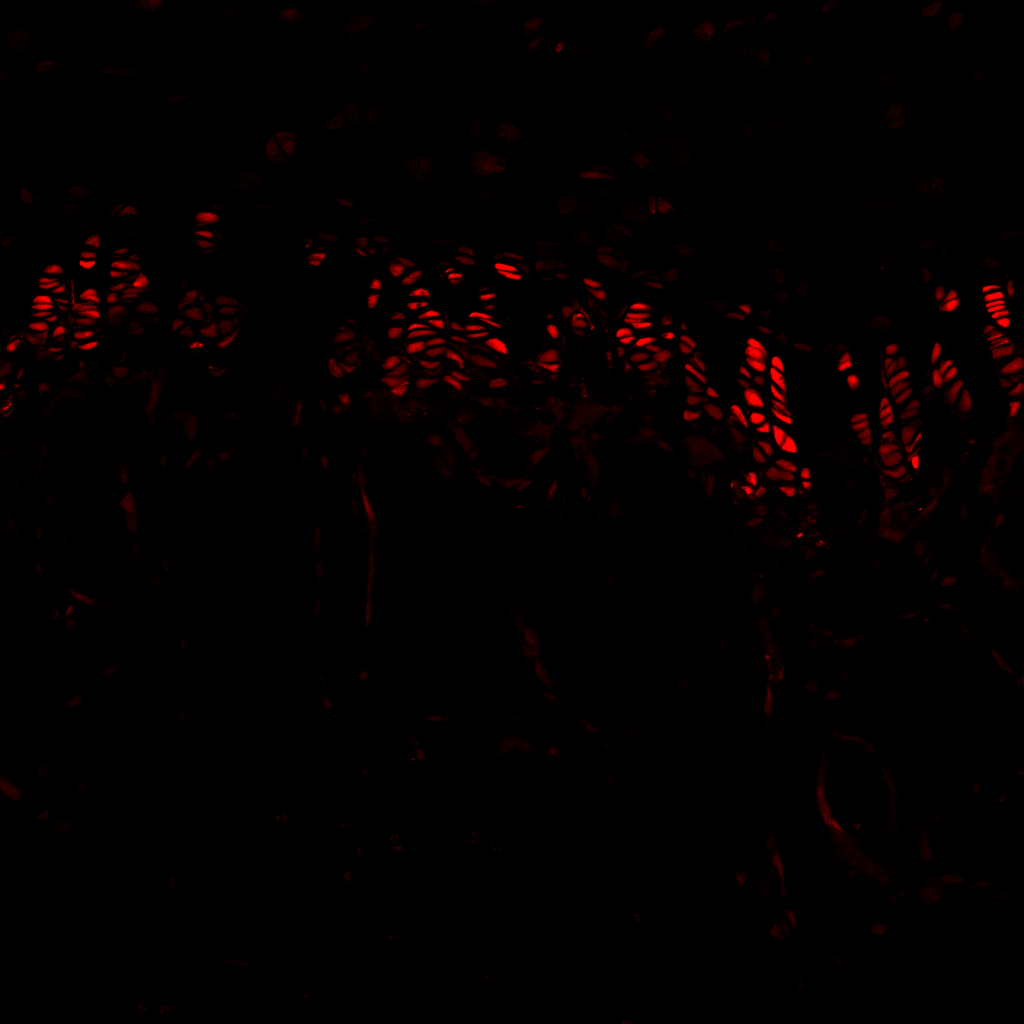

Supplement: Supplementary file 5 — Source Data Fig. 5 [file 44319_2024_93_MOESM5_ESM.zip › Figure5/5F/TM1M_1M_WT_VB_td_red.tif]

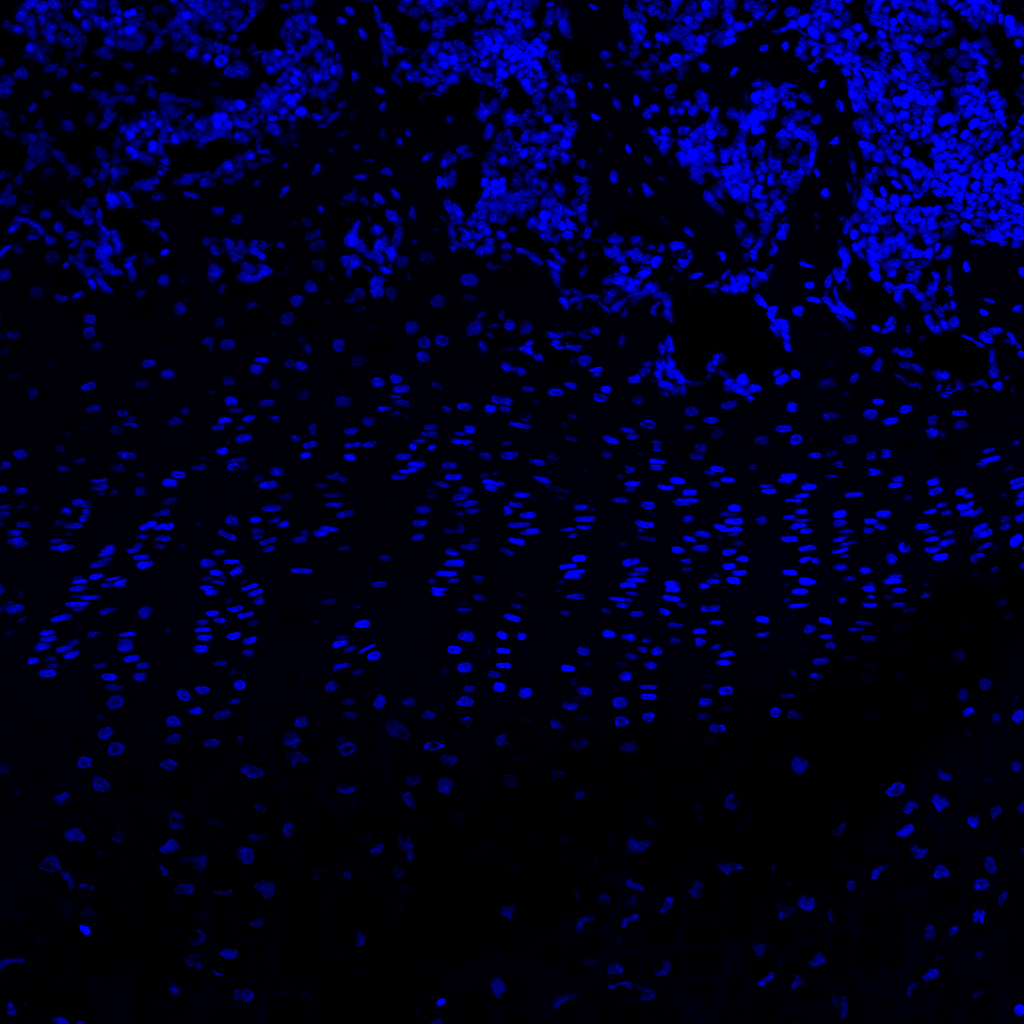

Supplement: Supplementary file 6 — Source Data Fig. 6 [file 44319_2024_93_MOESM6_ESM.zip › Figure6/6A/CKO_10D_GP_dapi_blue.tif]

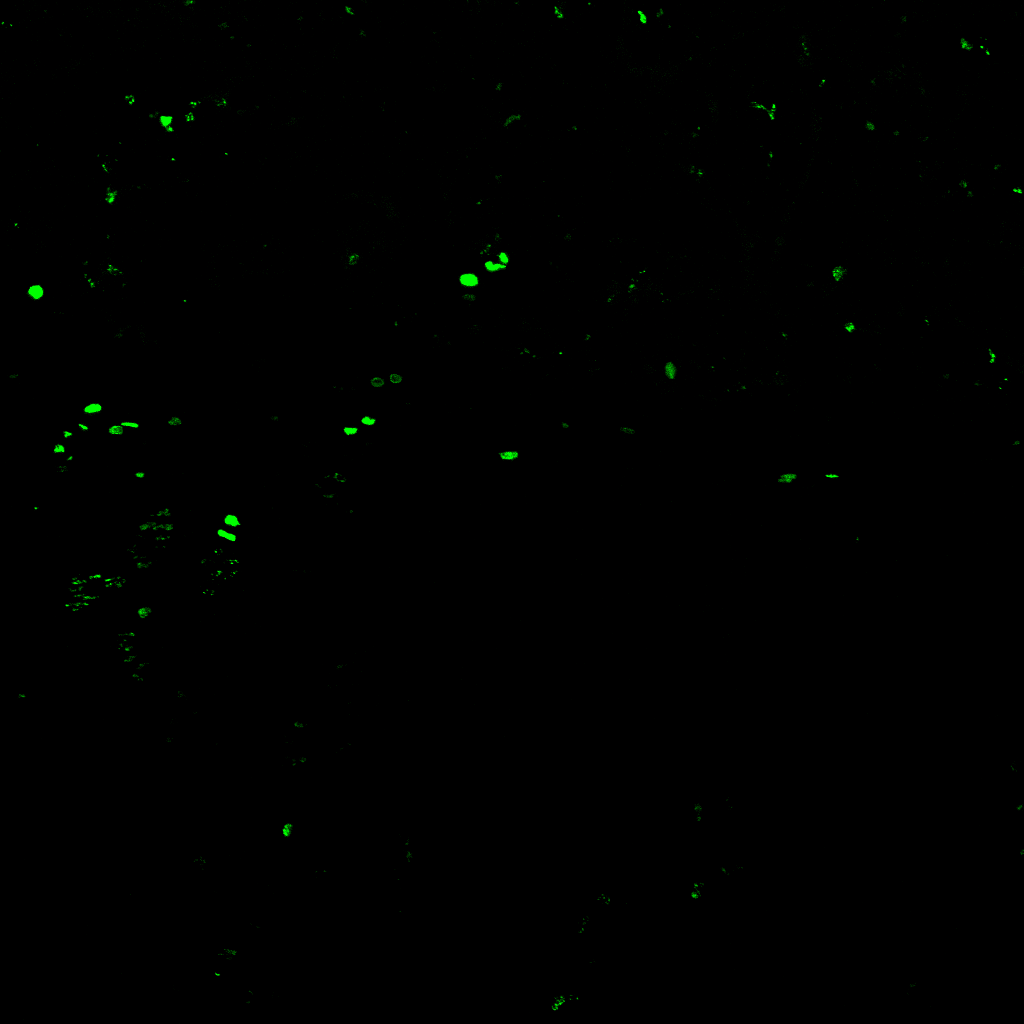

Supplement: Supplementary file 6 — Source Data Fig. 6 [file 44319_2024_93_MOESM6_ESM.zip › Figure6/6A/CKO_10D_GP_edu_green.tif]

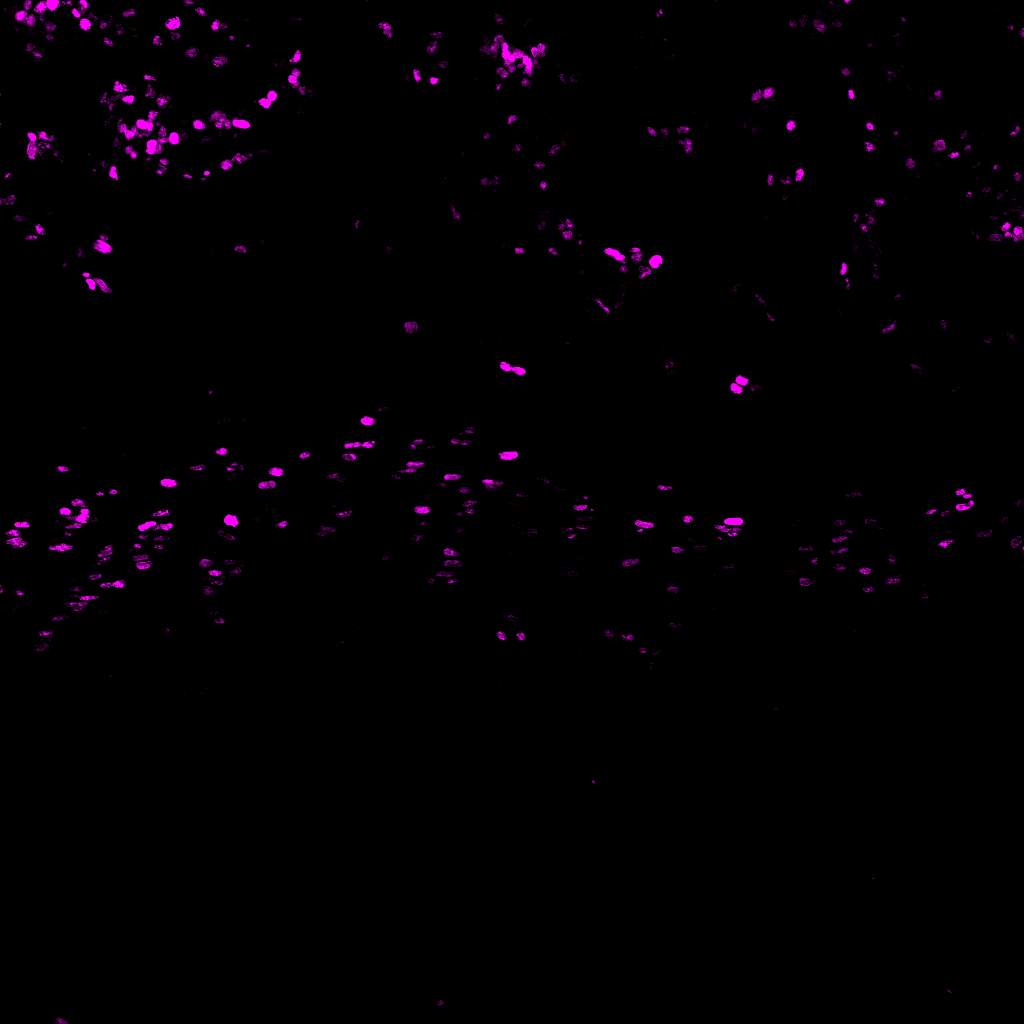

Supplement: Supplementary file 6 — Source Data Fig. 6 [file 44319_2024_93_MOESM6_ESM.zip › Figure6/6A/CKO_10D_GP_ki67_magenta.tif]

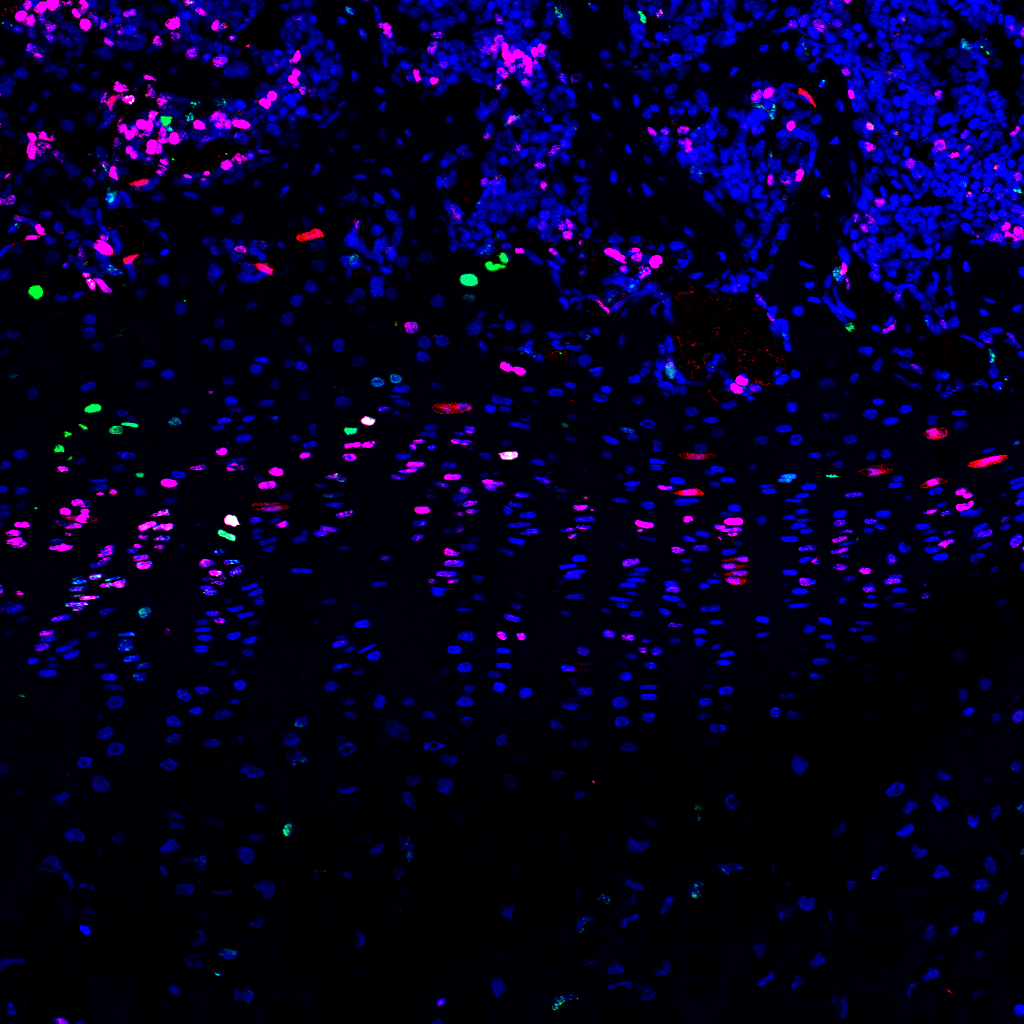

Supplement: Supplementary file 6 — Source Data Fig. 6 [file 44319_2024_93_MOESM6_ESM.zip › Figure6/6A/CKO_10D_GP_merge.tif]

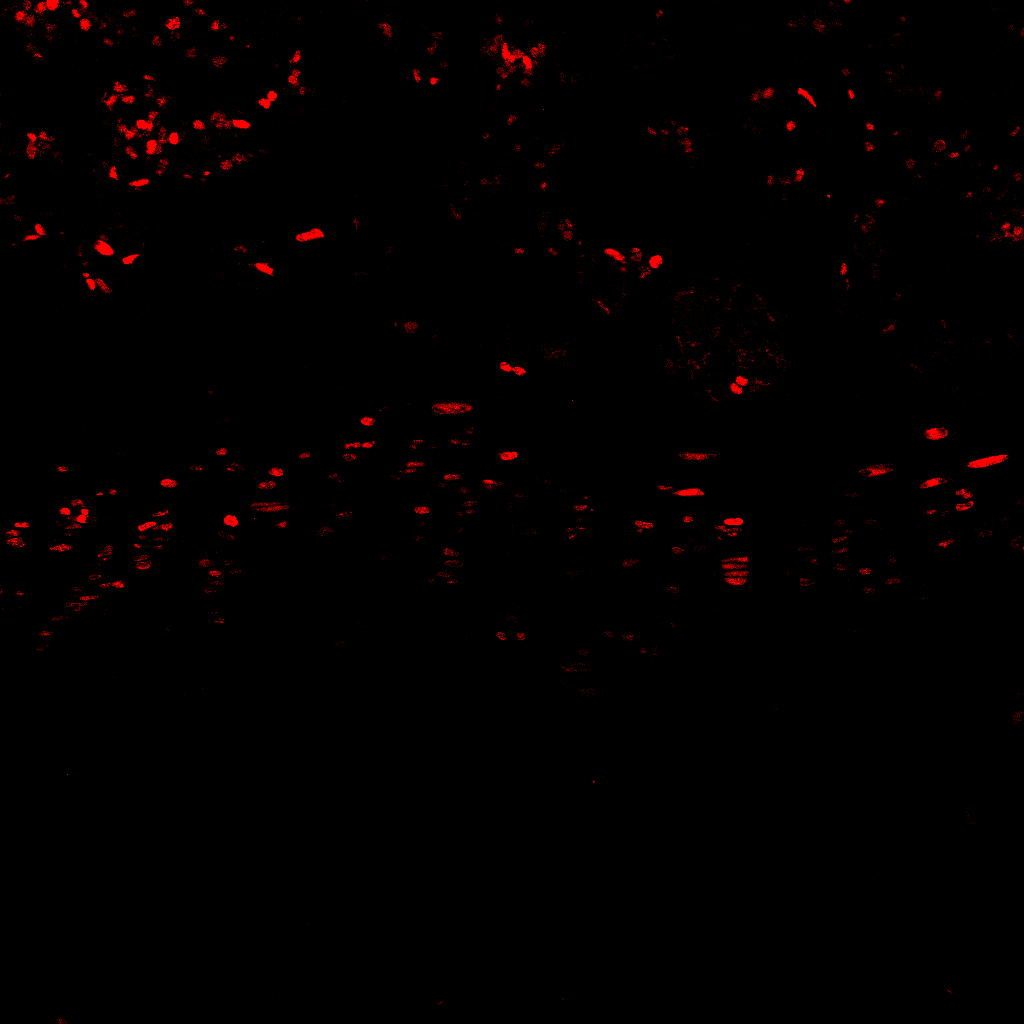

Supplement: Supplementary file 6 — Source Data Fig. 6 [file 44319_2024_93_MOESM6_ESM.zip › Figure6/6A/CKO_10D_GP_td_red.tif]

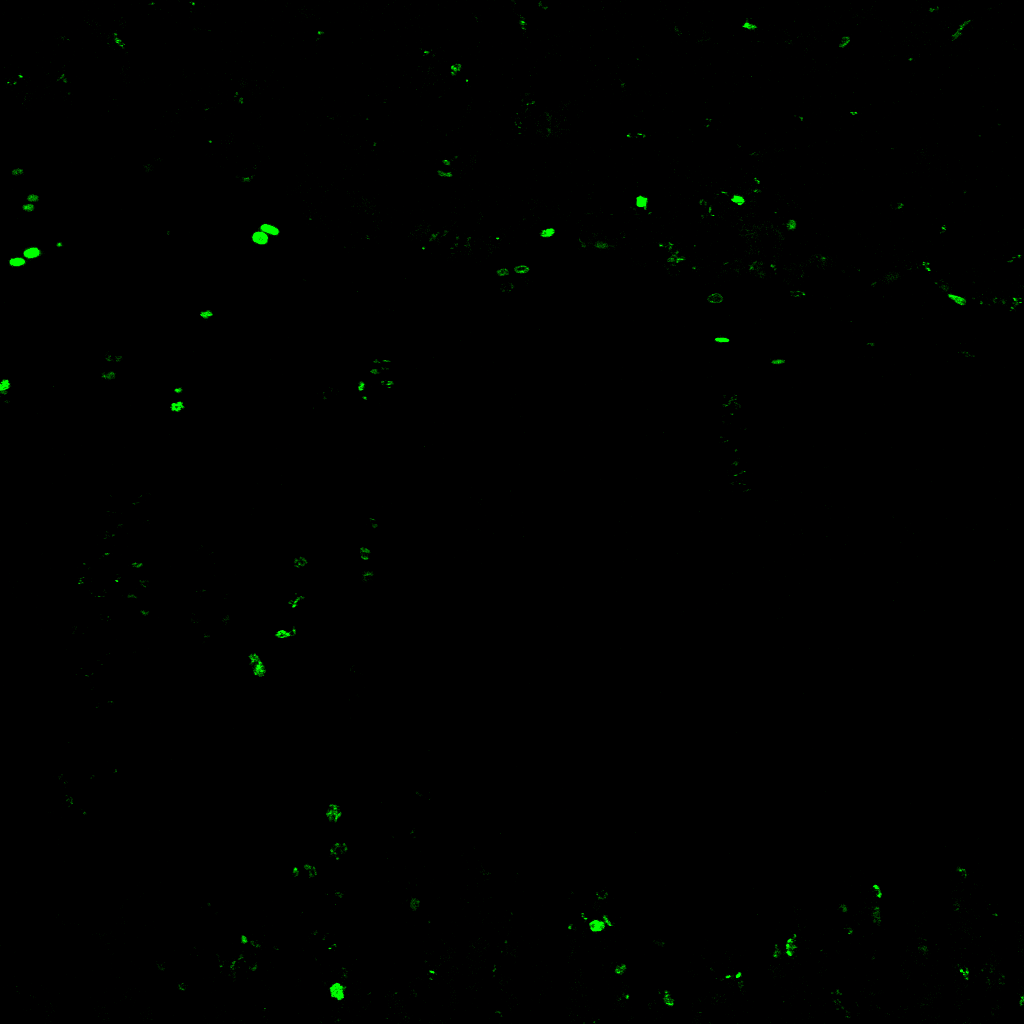

Supplement: Supplementary file 6 — Source Data Fig. 6 [file 44319_2024_93_MOESM6_ESM.zip › Figure6/6A/WT_10D__GP_edu_green.tif]

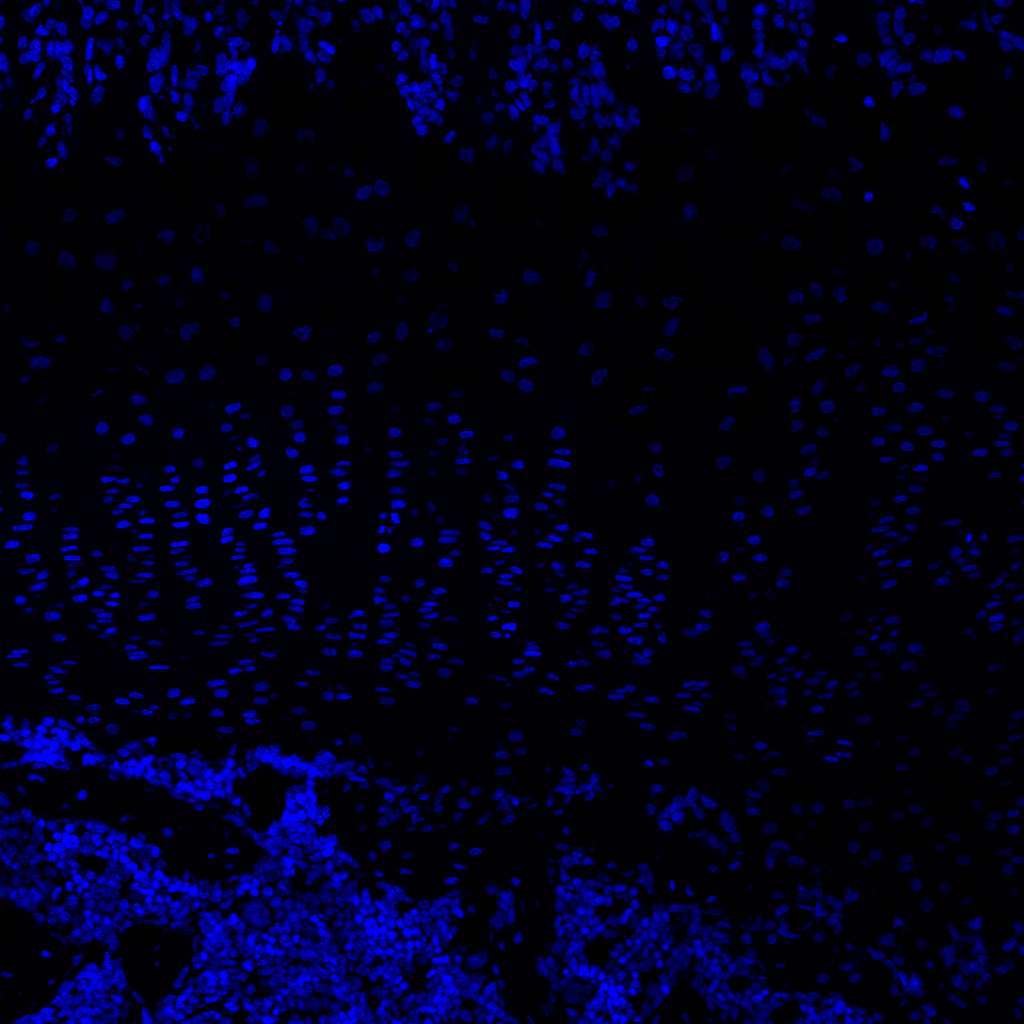

Supplement: Supplementary file 6 — Source Data Fig. 6 [file 44319_2024_93_MOESM6_ESM.zip › Figure6/6A/WT_10D_GP_dapi_blue.tif]

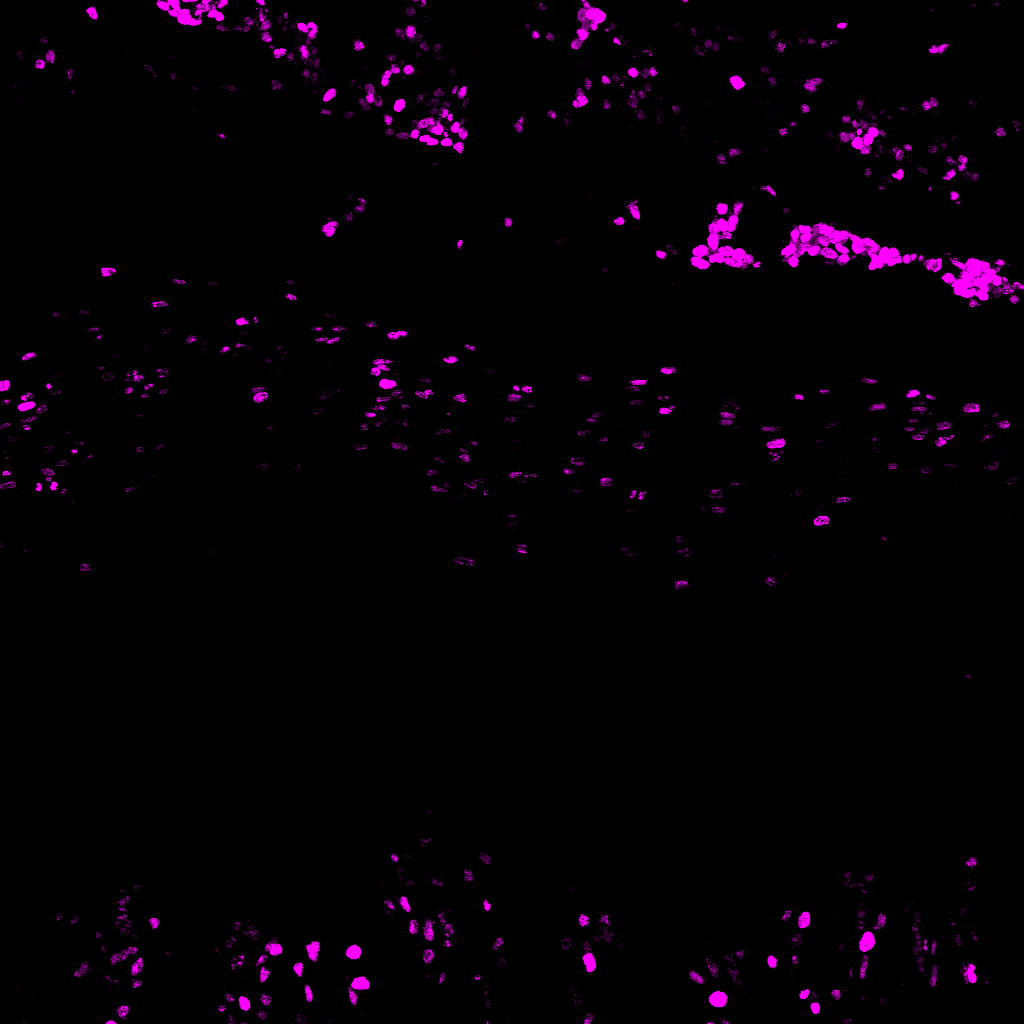

Supplement: Supplementary file 6 — Source Data Fig. 6 [file 44319_2024_93_MOESM6_ESM.zip › Figure6/6A/WT_10D_GP_ki67_magenta.tif]

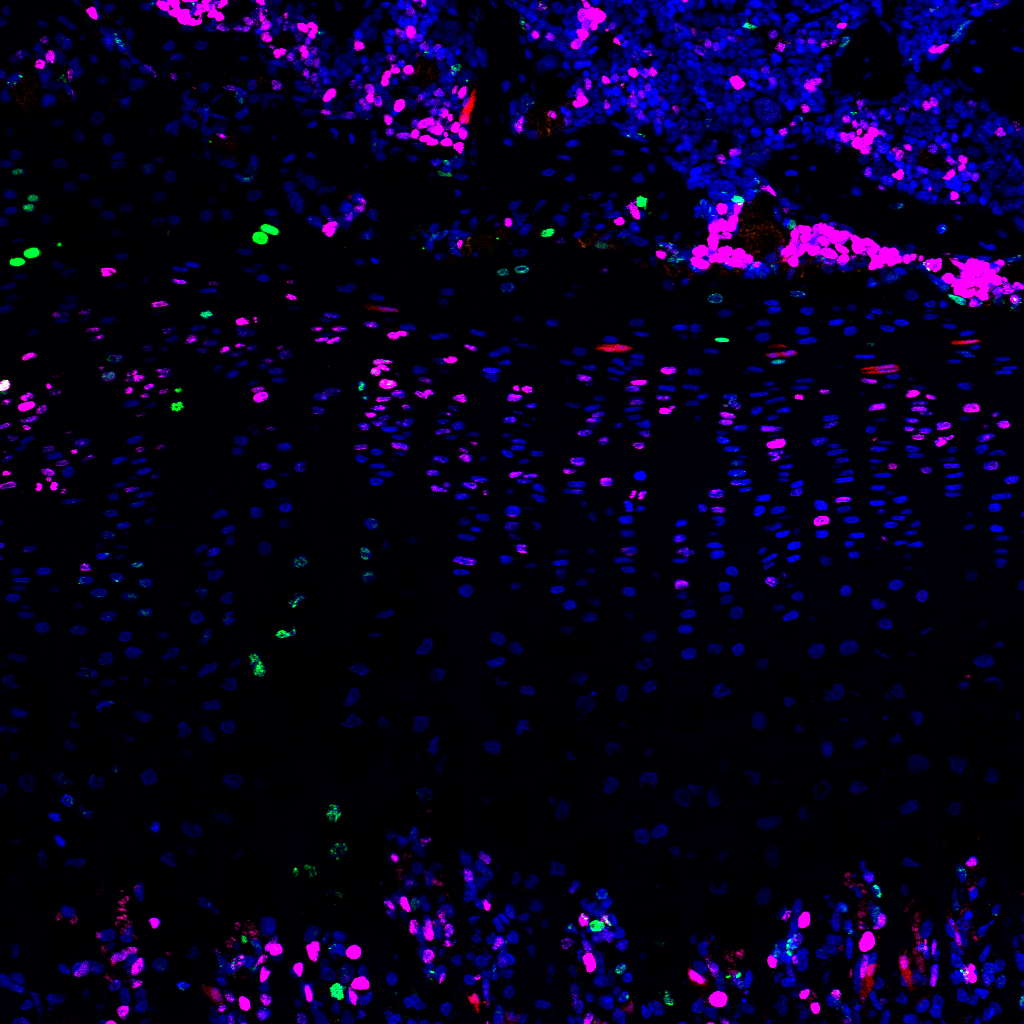

Supplement: Supplementary file 6 — Source Data Fig. 6 [file 44319_2024_93_MOESM6_ESM.zip › Figure6/6A/WT_10D_GP_merge.tif]

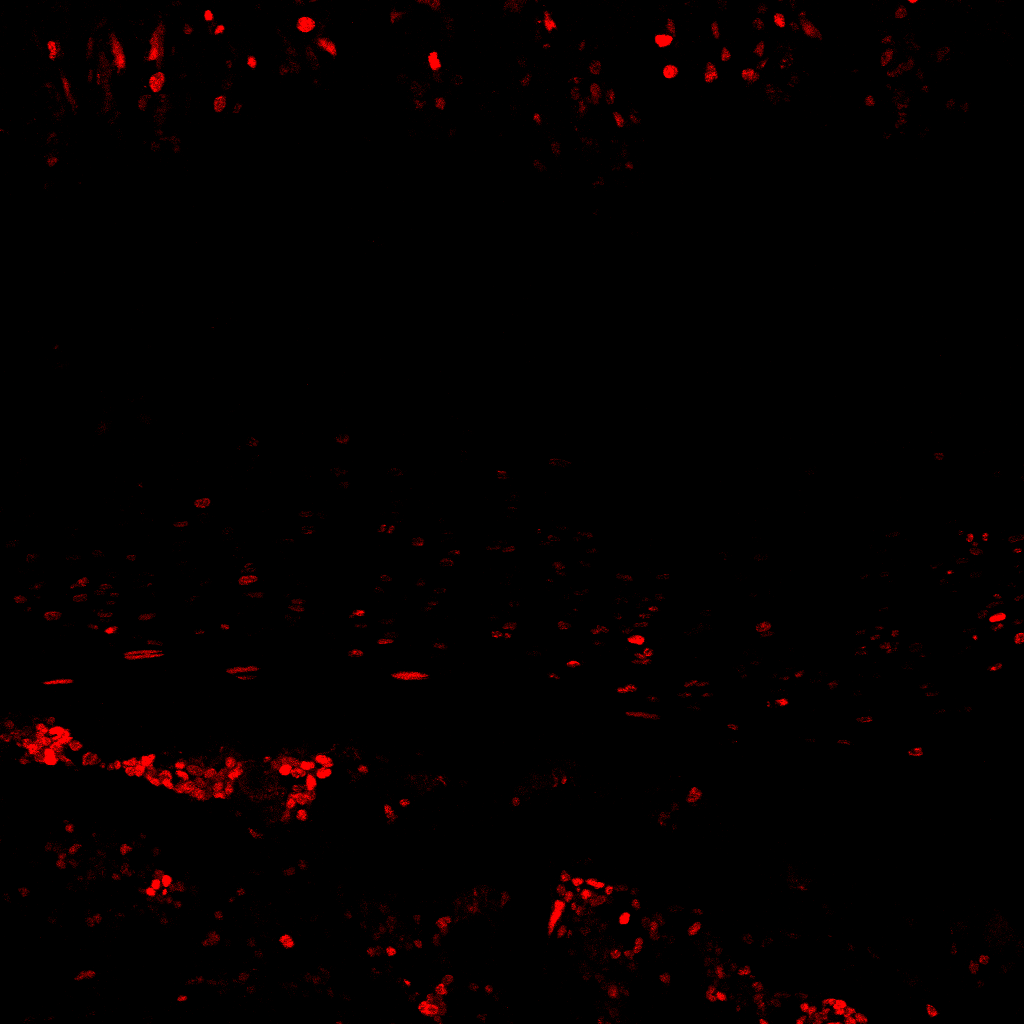

Supplement: Supplementary file 6 — Source Data Fig. 6 [file 44319_2024_93_MOESM6_ESM.zip › Figure6/6A/WT_10D_GP_td_red.tif]

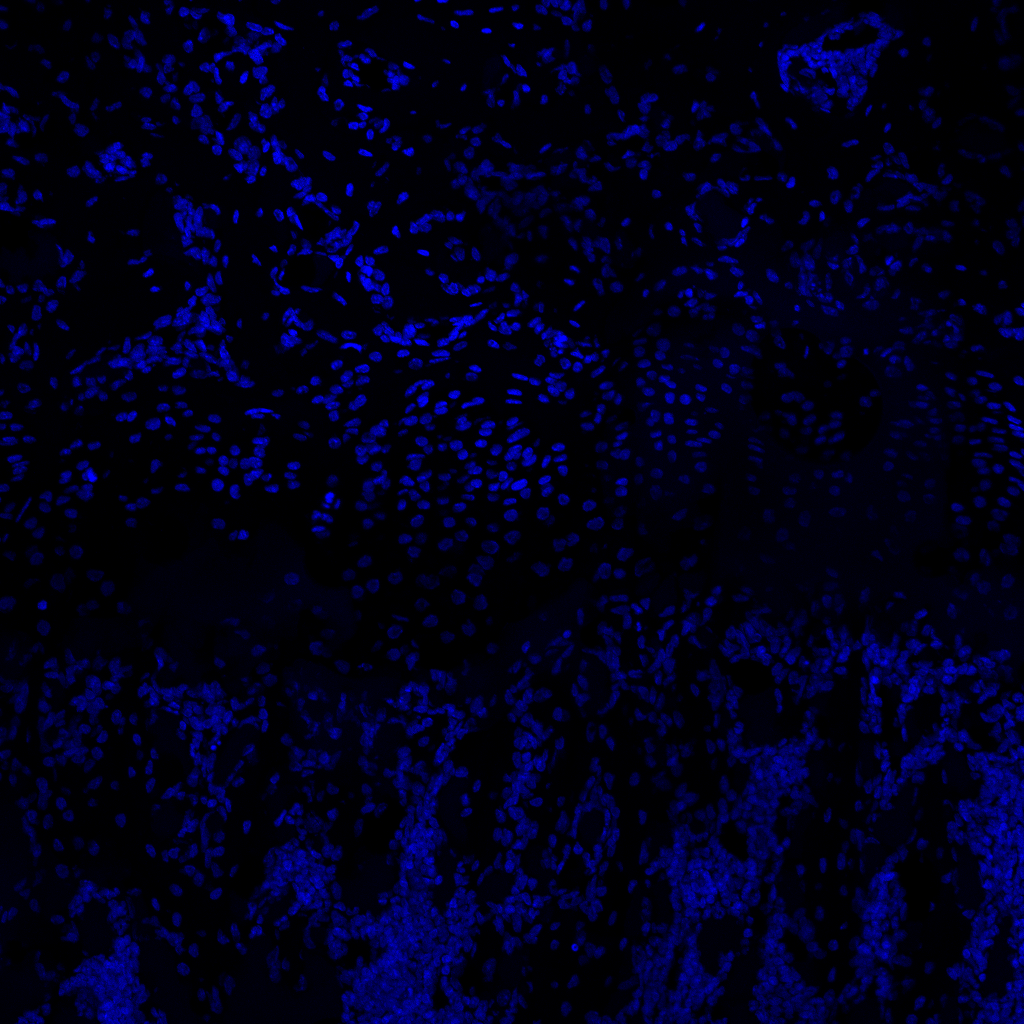

Supplement: Supplementary file 6 — Source Data Fig. 6 [file 44319_2024_93_MOESM6_ESM.zip › Figure6/6D/CKO_17D_GP_dapi_blue.tif]

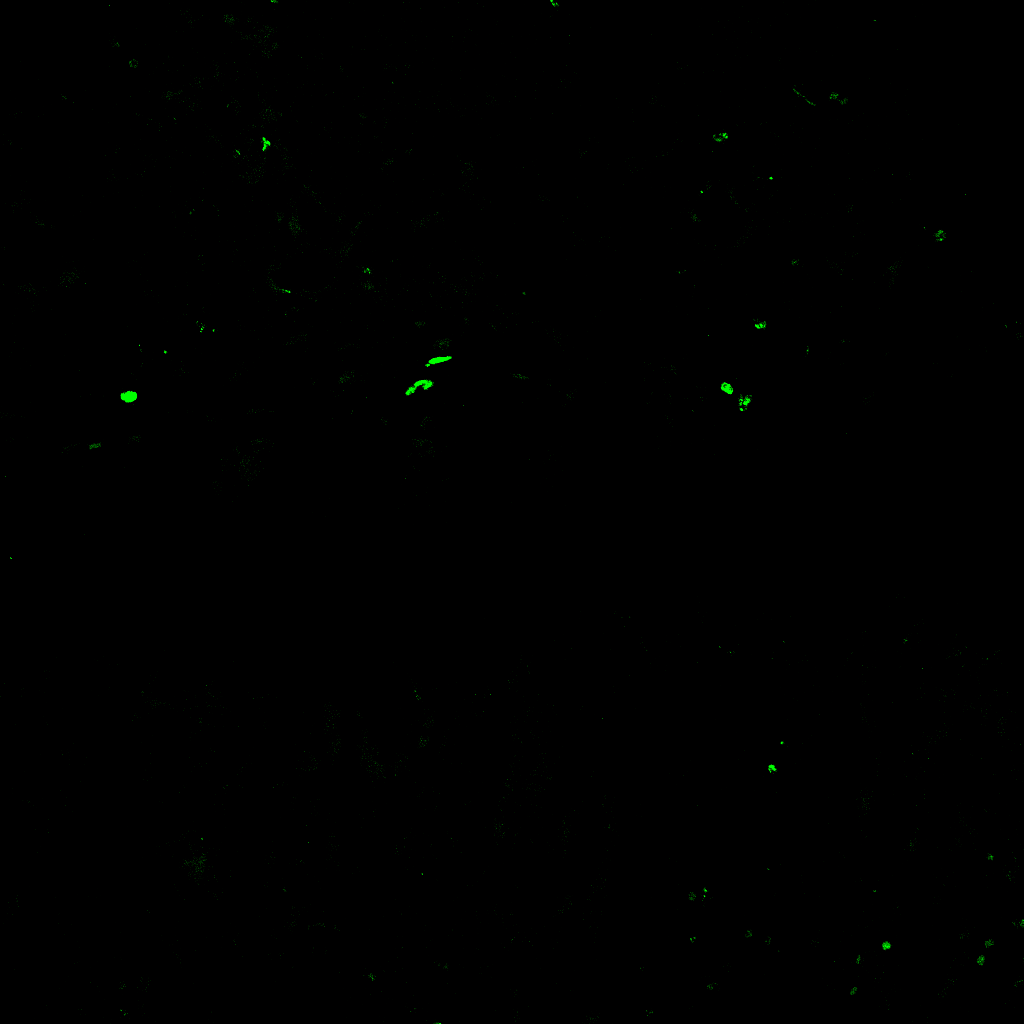

Supplement: Supplementary file 6 — Source Data Fig. 6 [file 44319_2024_93_MOESM6_ESM.zip › Figure6/6D/CKO_17D_GP_edu_green.tif]

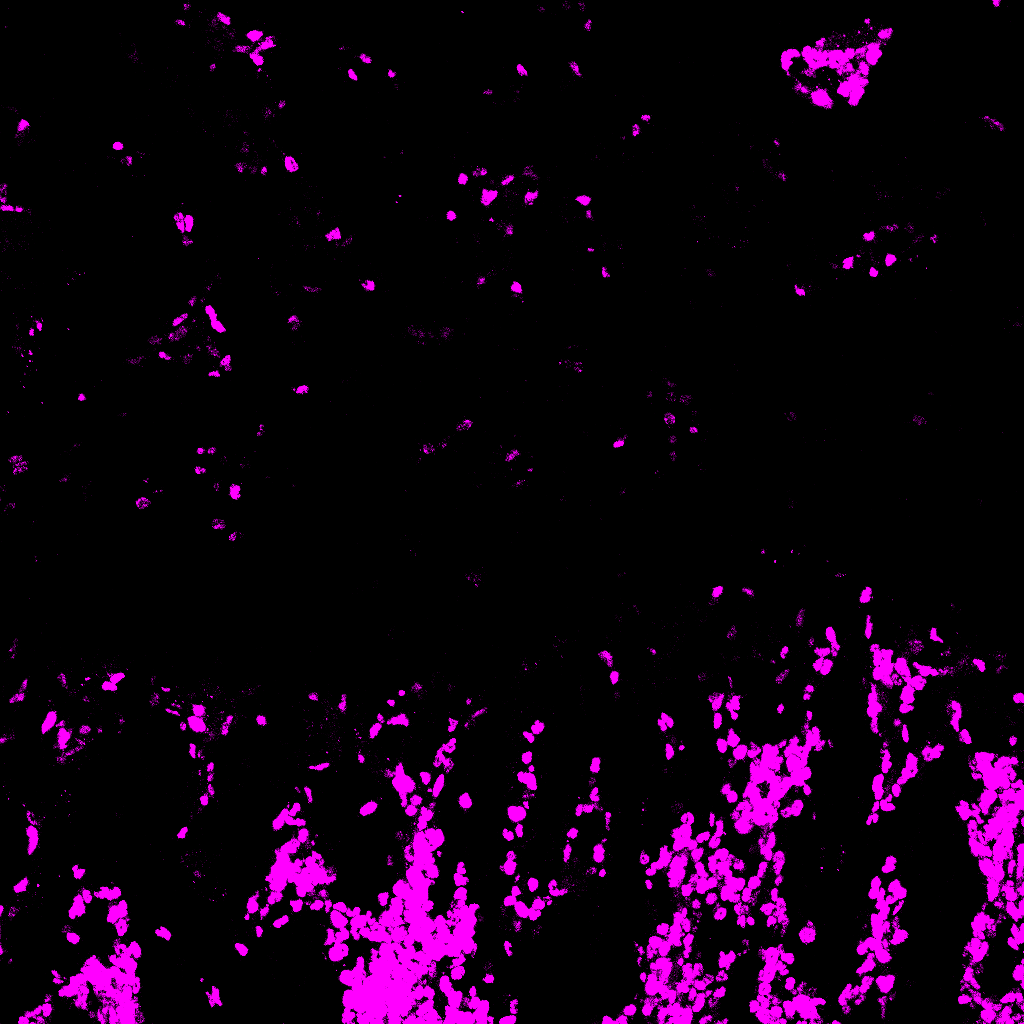

Supplement: Supplementary file 6 — Source Data Fig. 6 [file 44319_2024_93_MOESM6_ESM.zip › Figure6/6D/CKO_17D_GP_ki67_magenta.tif]

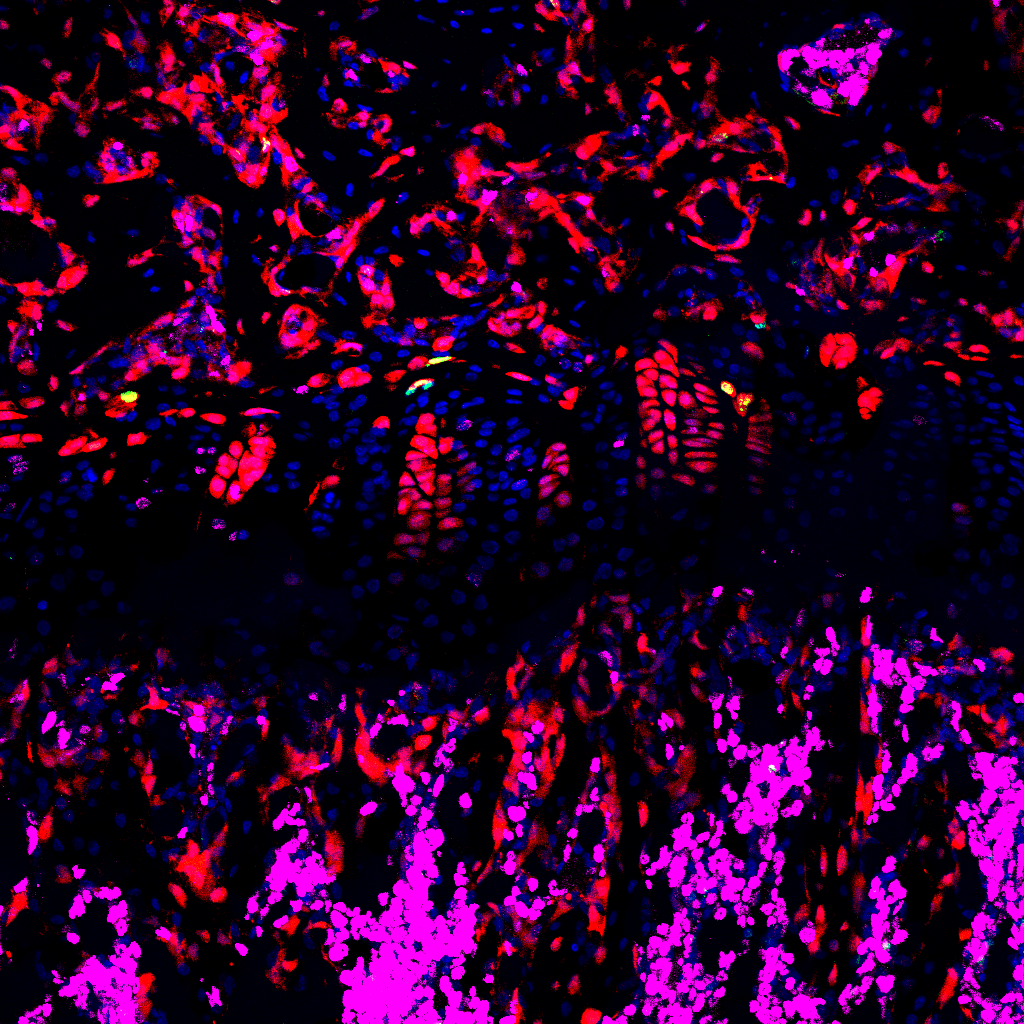

Supplement: Supplementary file 6 — Source Data Fig. 6 [file 44319_2024_93_MOESM6_ESM.zip › Figure6/6D/CKO_17D_GP_merge.tif]

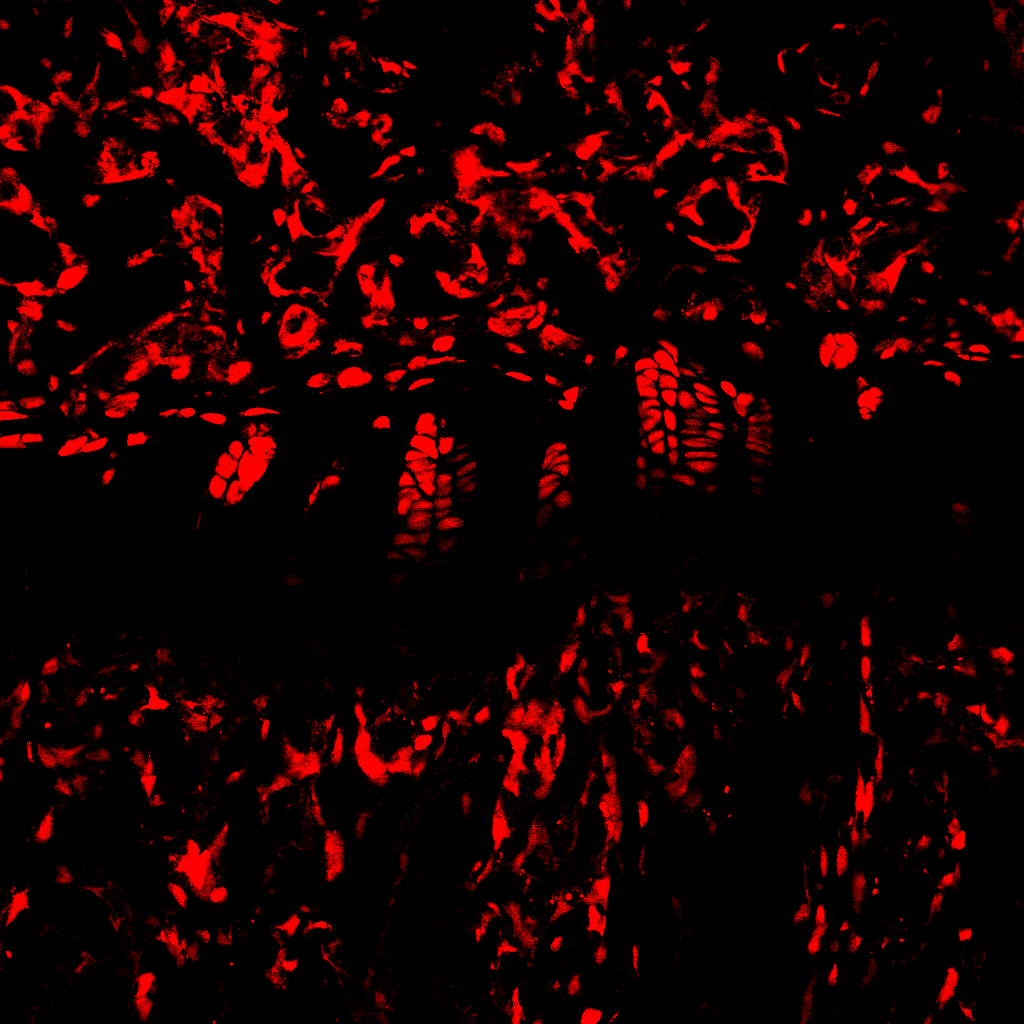

Supplement: Supplementary file 6 — Source Data Fig. 6 [file 44319_2024_93_MOESM6_ESM.zip › Figure6/6D/CKO_17D_GP_td_red.tif]

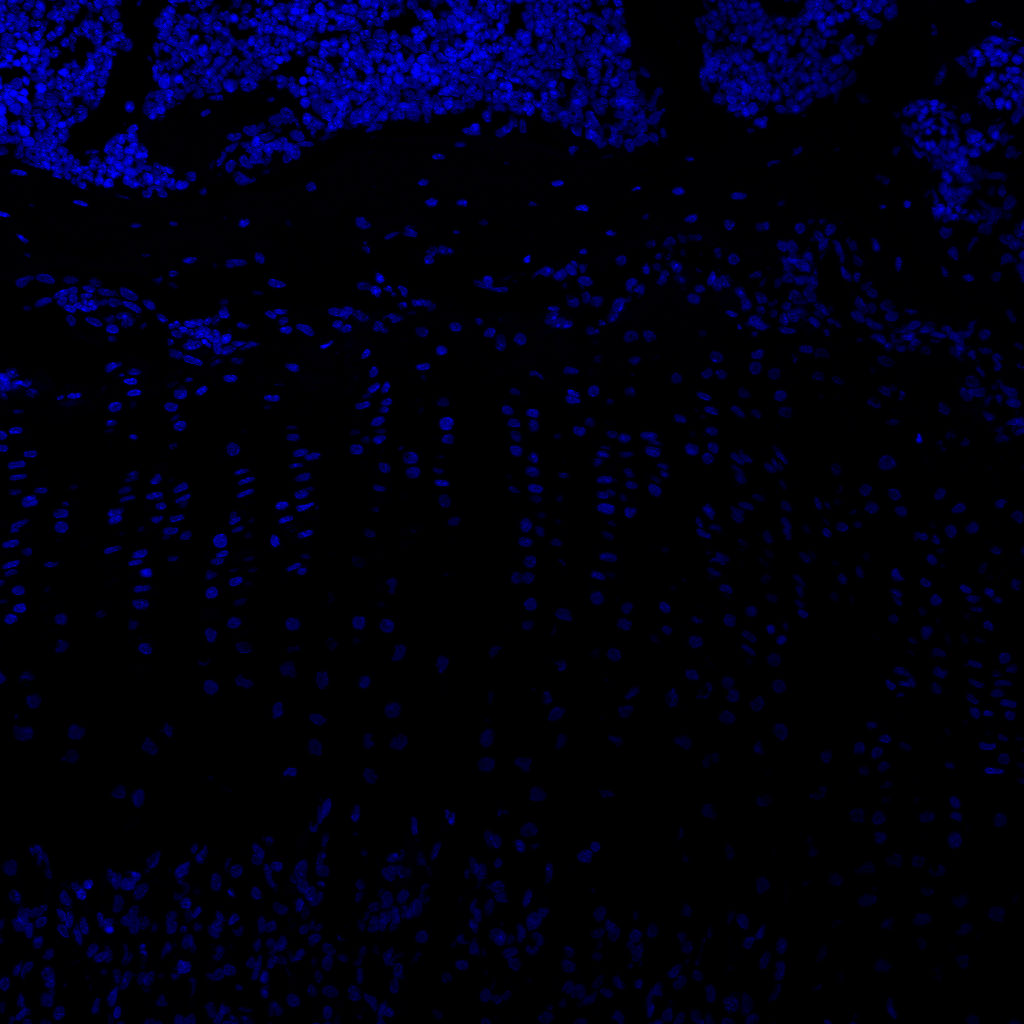

Supplement: Supplementary file 6 — Source Data Fig. 6 [file 44319_2024_93_MOESM6_ESM.zip › Figure6/6D/WT_17D_GP_dapi_blue.tif]

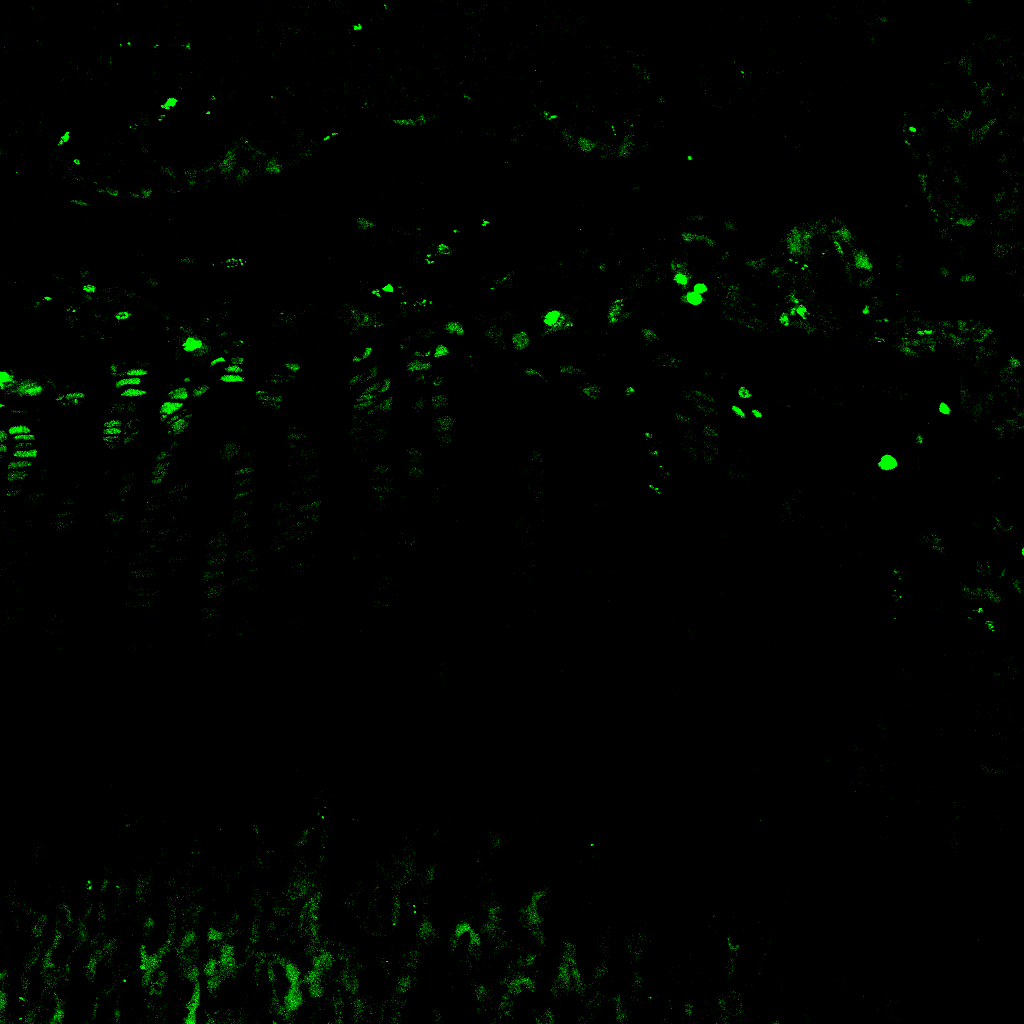

Supplement: Supplementary file 6 — Source Data Fig. 6 [file 44319_2024_93_MOESM6_ESM.zip › Figure6/6D/WT_17D_GP_edu_green.tif]

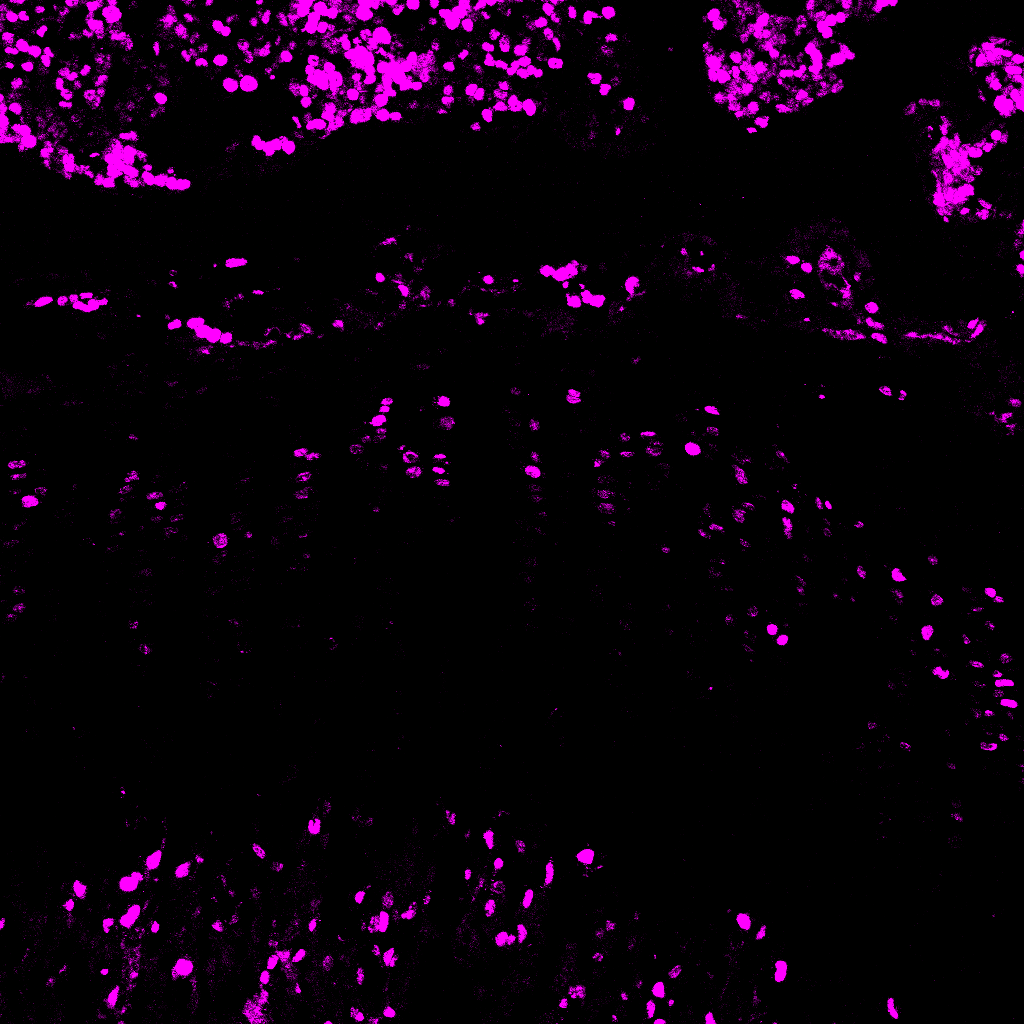

Supplement: Supplementary file 6 — Source Data Fig. 6 [file 44319_2024_93_MOESM6_ESM.zip › Figure6/6D/WT_17D_GP_ki67_magenta.tif]

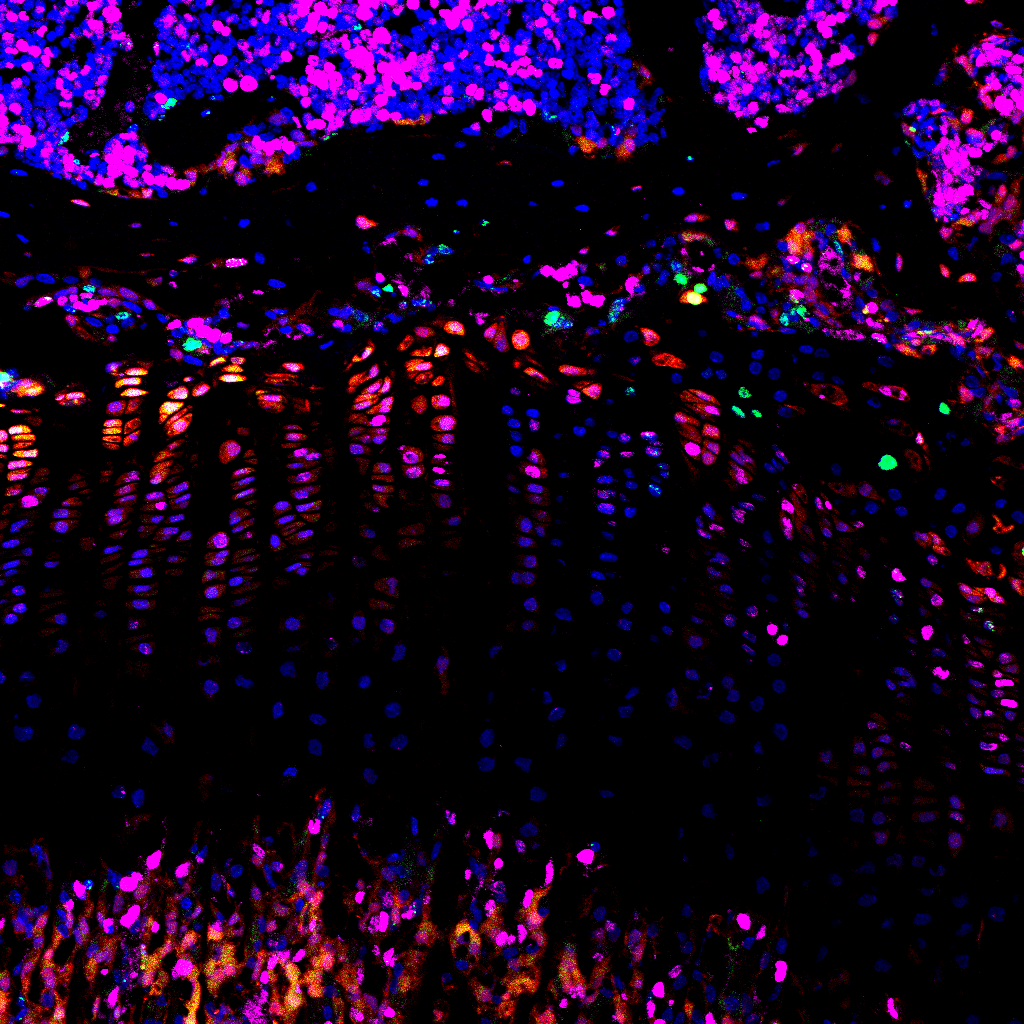

Supplement: Supplementary file 6 — Source Data Fig. 6 [file 44319_2024_93_MOESM6_ESM.zip › Figure6/6D/WT_17D_GP_merge.tif]

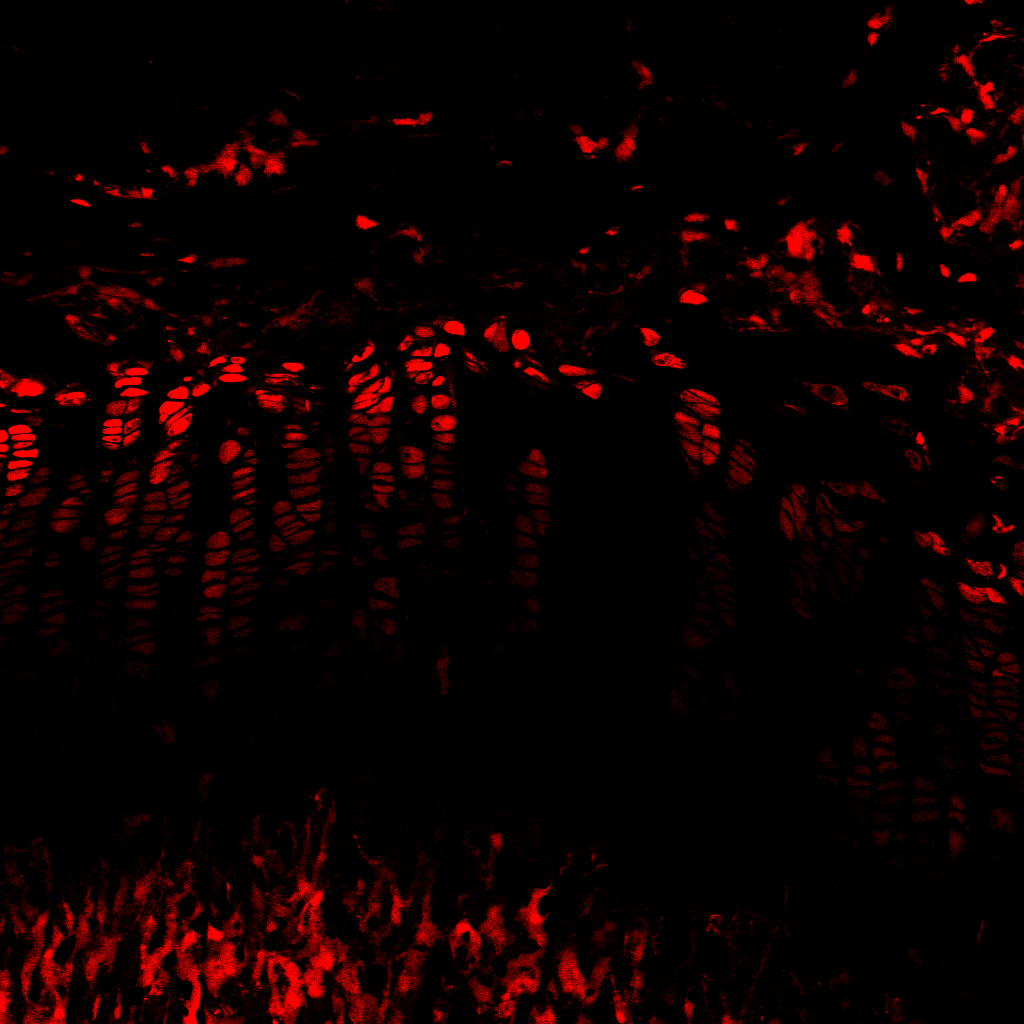

Supplement: Supplementary file 6 — Source Data Fig. 6 [file 44319_2024_93_MOESM6_ESM.zip › Figure6/6D/WT_17D_GP_td_red.tif]

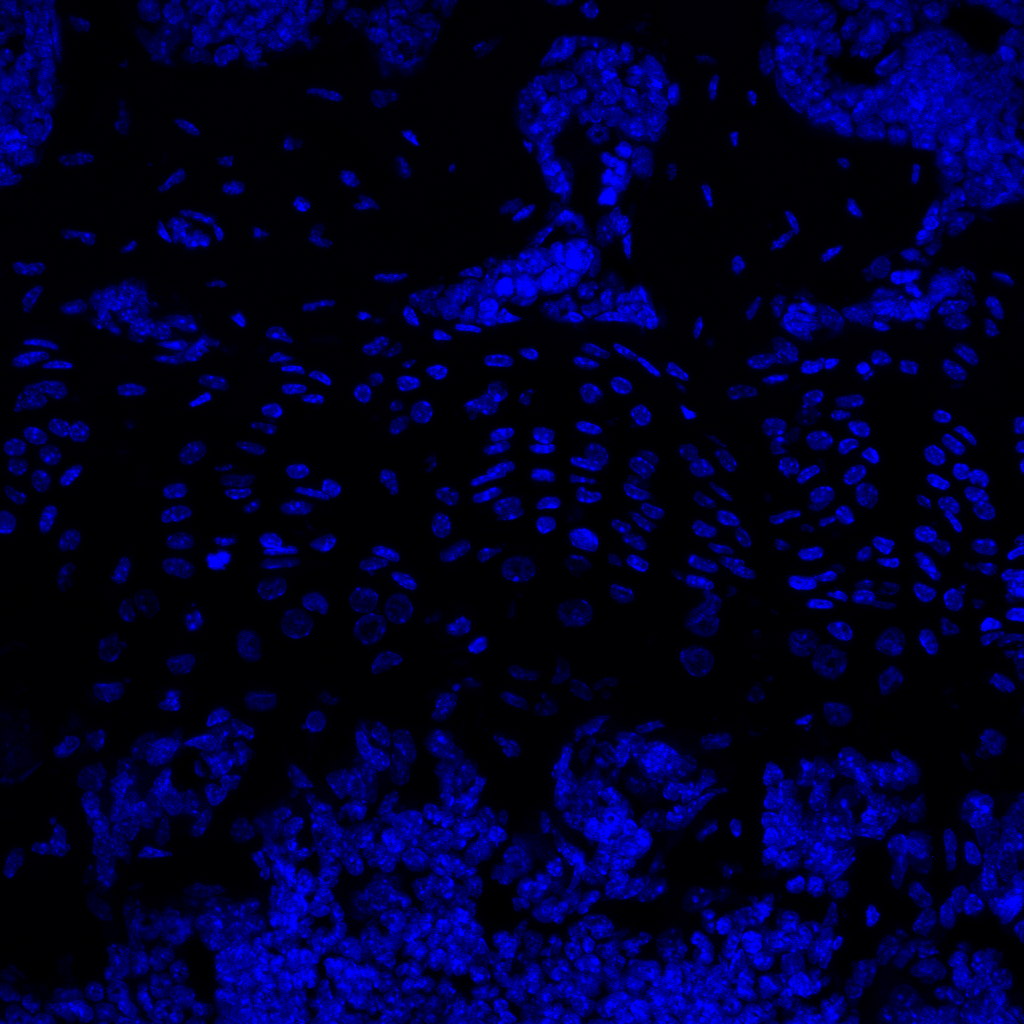

Supplement: Supplementary file 6 — Source Data Fig. 6 [file 44319_2024_93_MOESM6_ESM.zip › Figure6/6G/CKO_dapi_blue.tif]

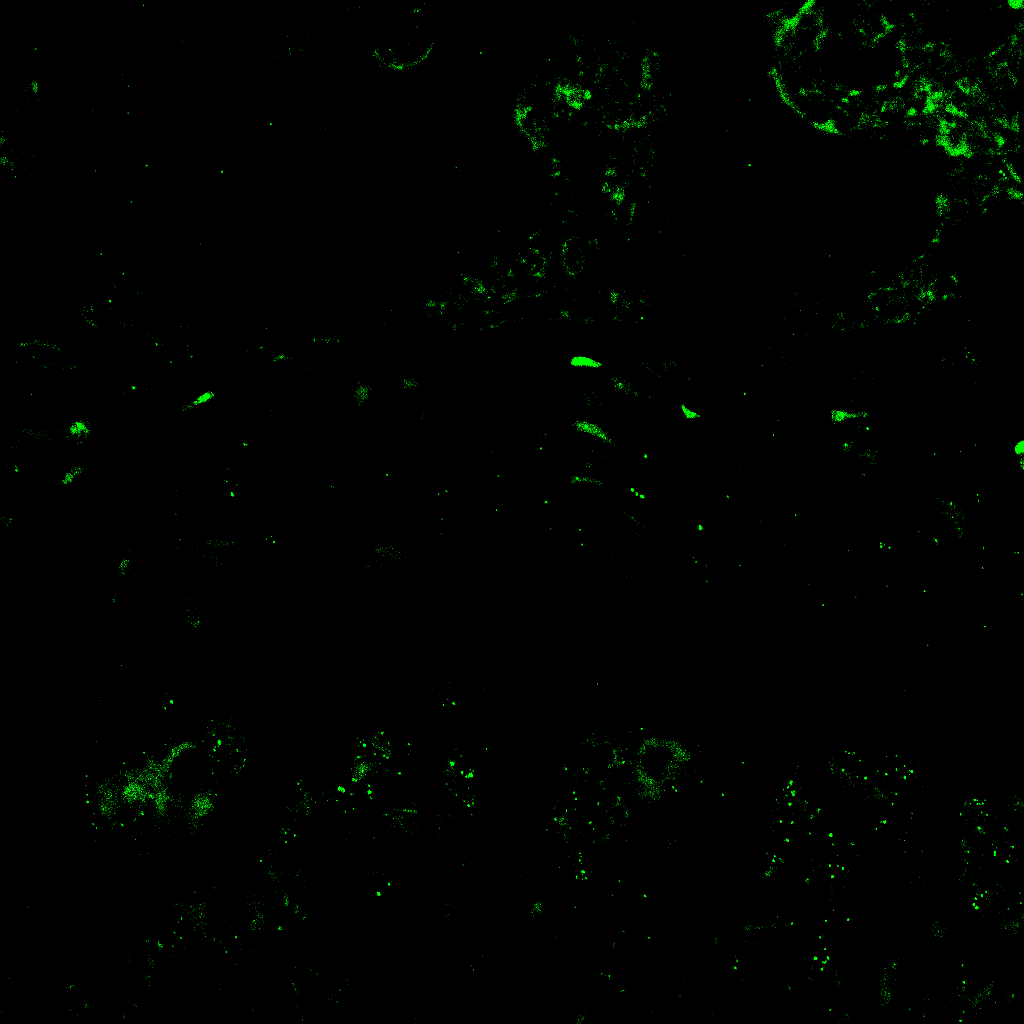

Supplement: Supplementary file 6 — Source Data Fig. 6 [file 44319_2024_93_MOESM6_ESM.zip › Figure6/6G/CKO_Gli1_green.tif]

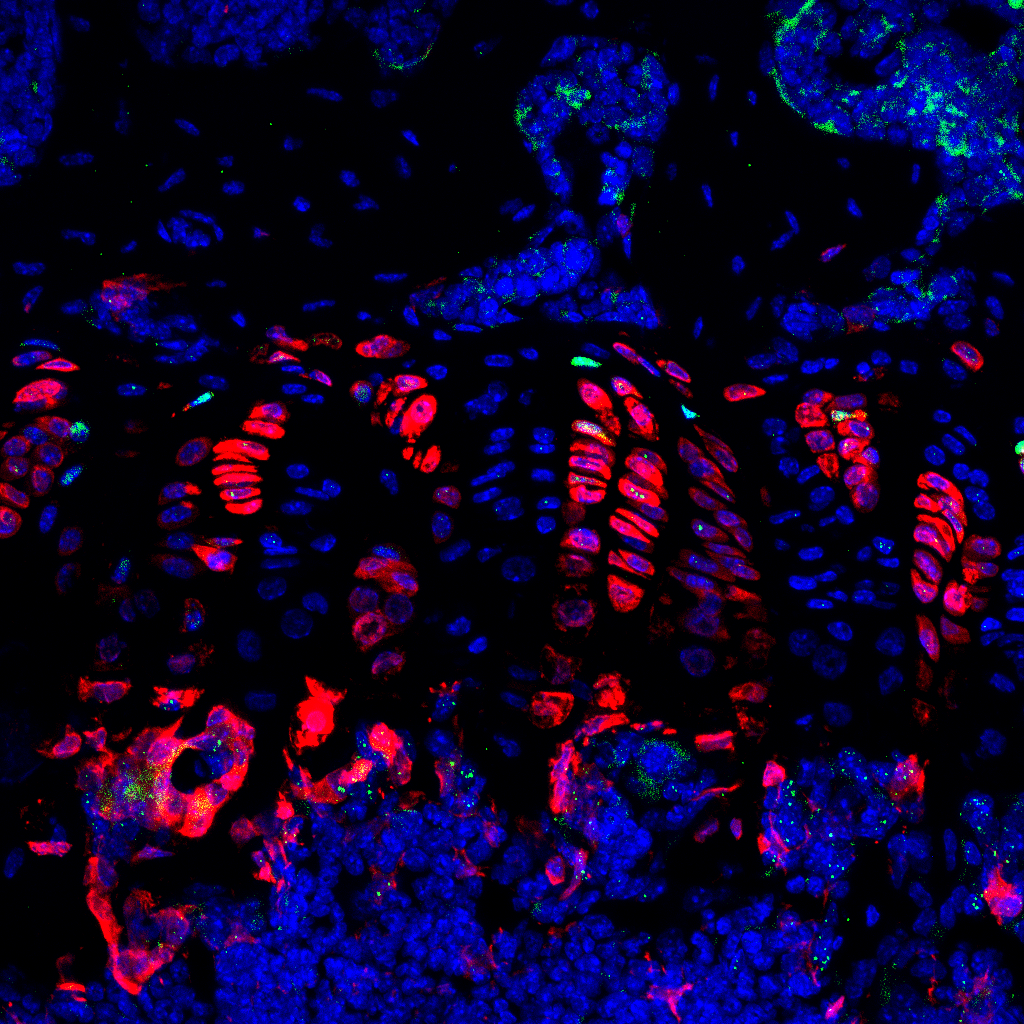

Supplement: Supplementary file 6 — Source Data Fig. 6 [file 44319_2024_93_MOESM6_ESM.zip › Figure6/6G/CKO_merge.tif]

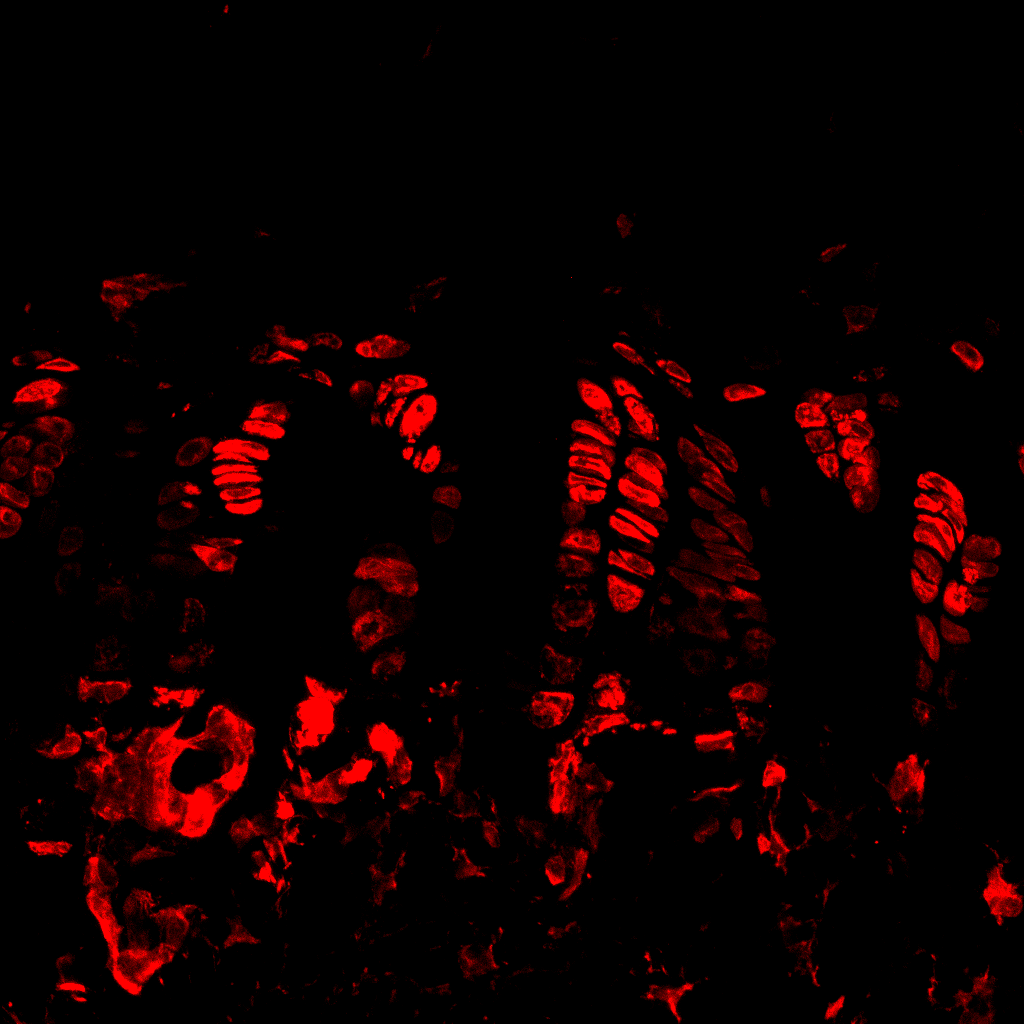

Supplement: Supplementary file 6 — Source Data Fig. 6 [file 44319_2024_93_MOESM6_ESM.zip › Figure6/6G/CKO_td_red.tif]

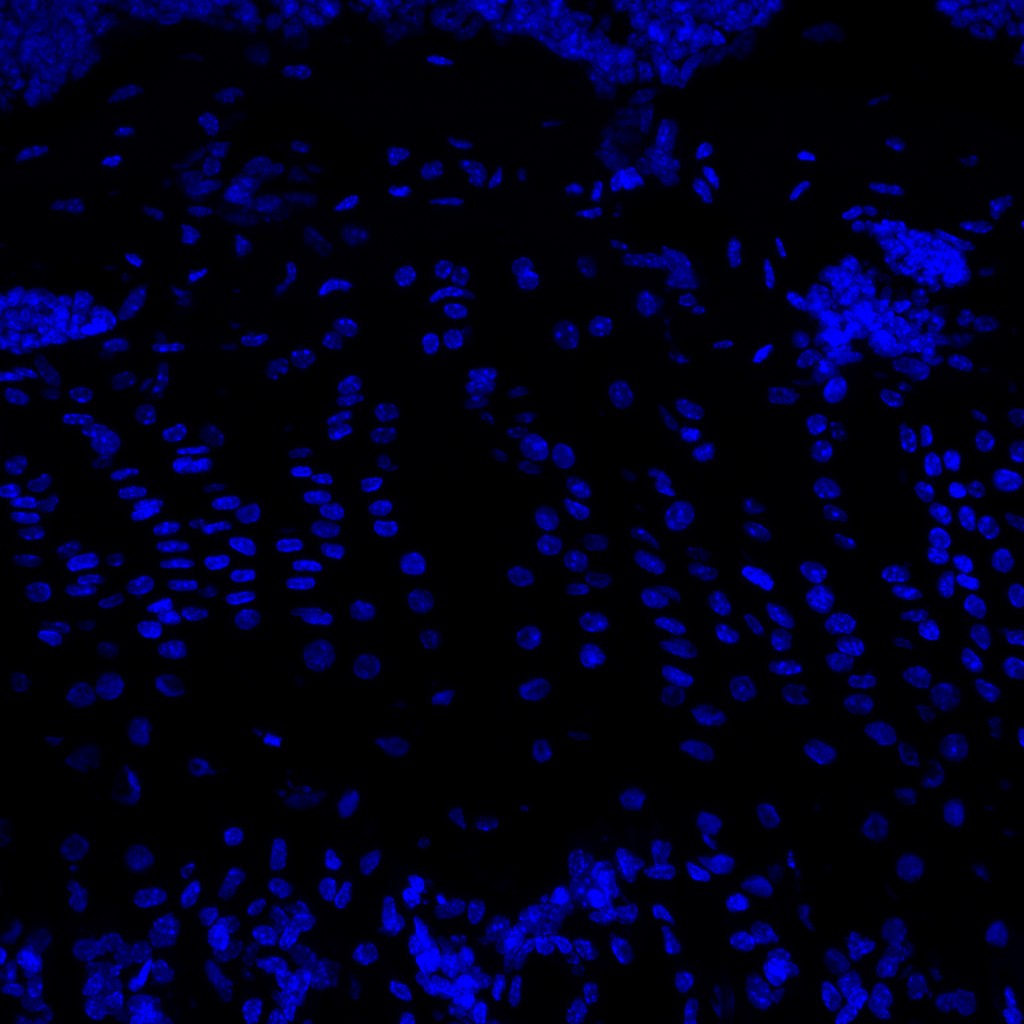

Supplement: Supplementary file 6 — Source Data Fig. 6 [file 44319_2024_93_MOESM6_ESM.zip › Figure6/6G/WT_dapi_blue.tif]

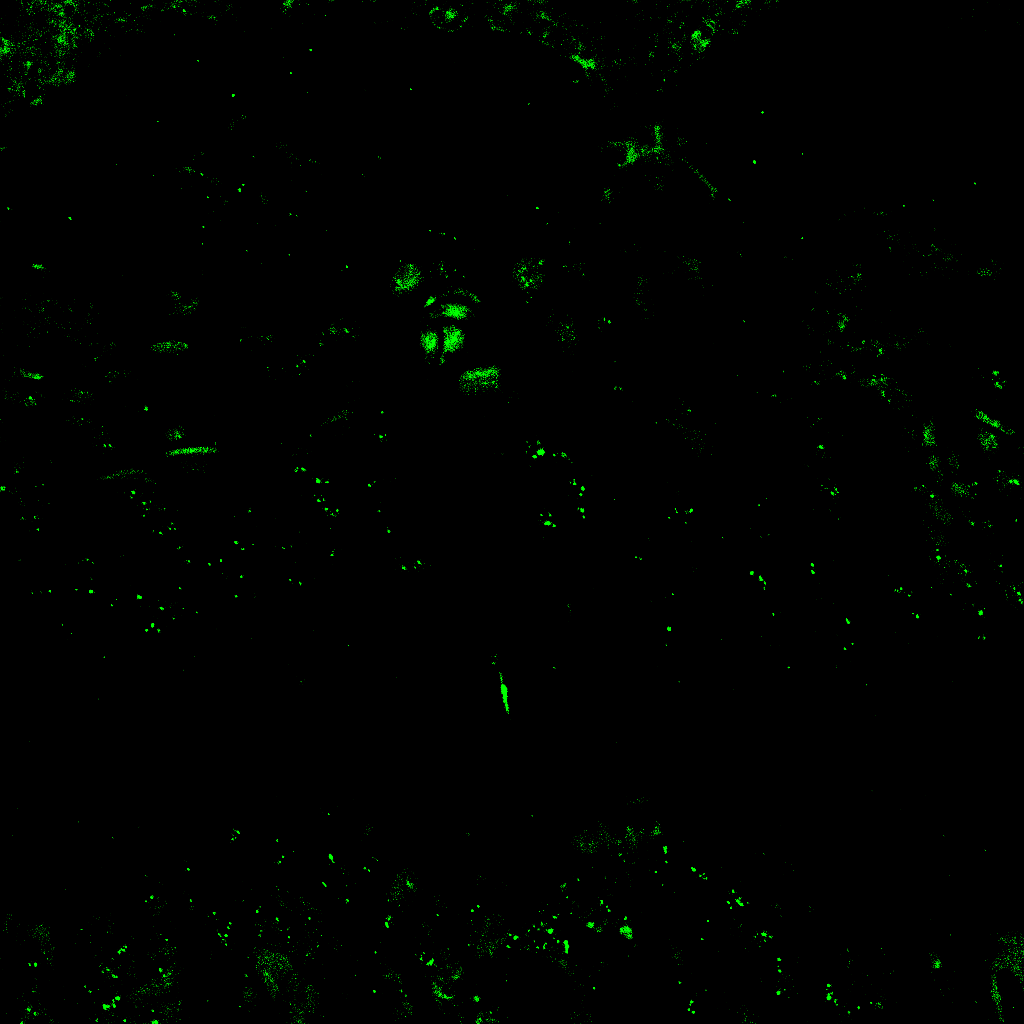

Supplement: Supplementary file 6 — Source Data Fig. 6 [file 44319_2024_93_MOESM6_ESM.zip › Figure6/6G/WT_Gli1_green.tif]

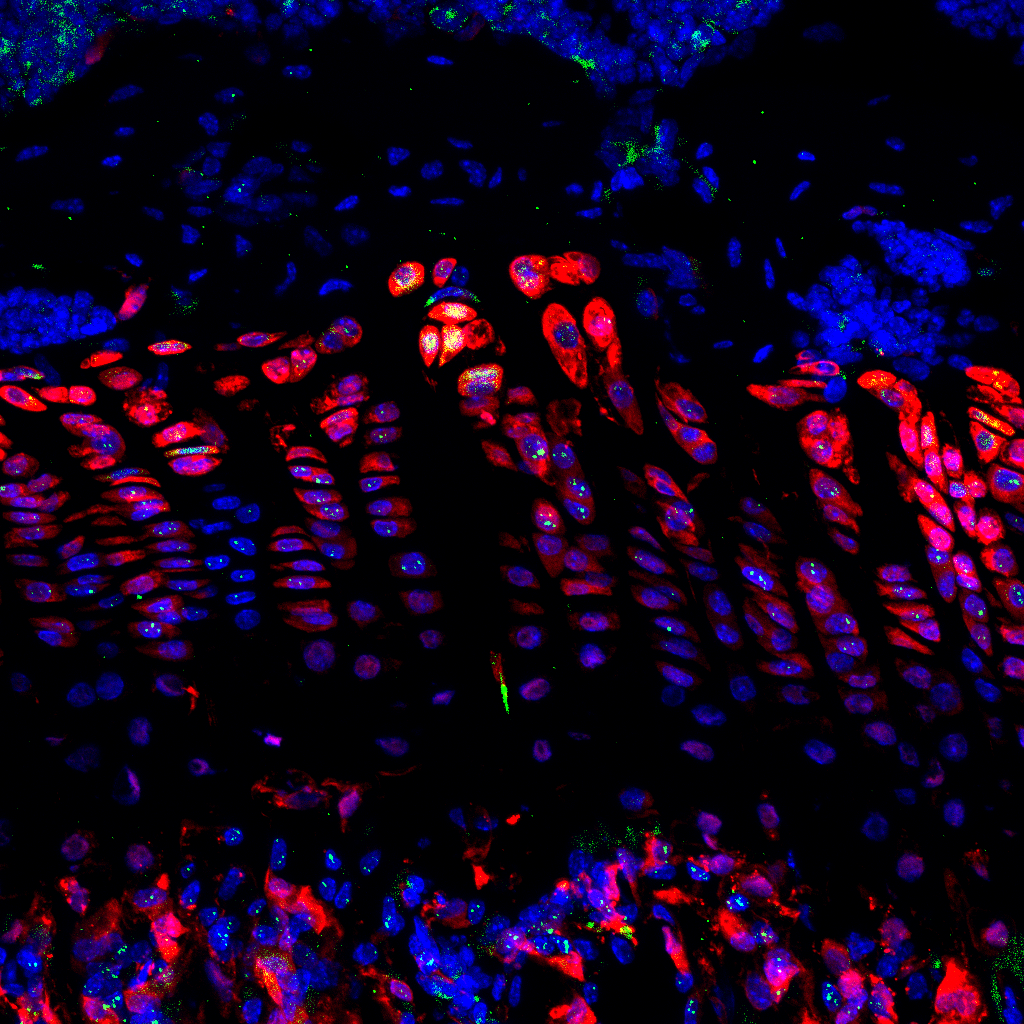

Supplement: Supplementary file 6 — Source Data Fig. 6 [file 44319_2024_93_MOESM6_ESM.zip › Figure6/6G/WT_merge.tif]

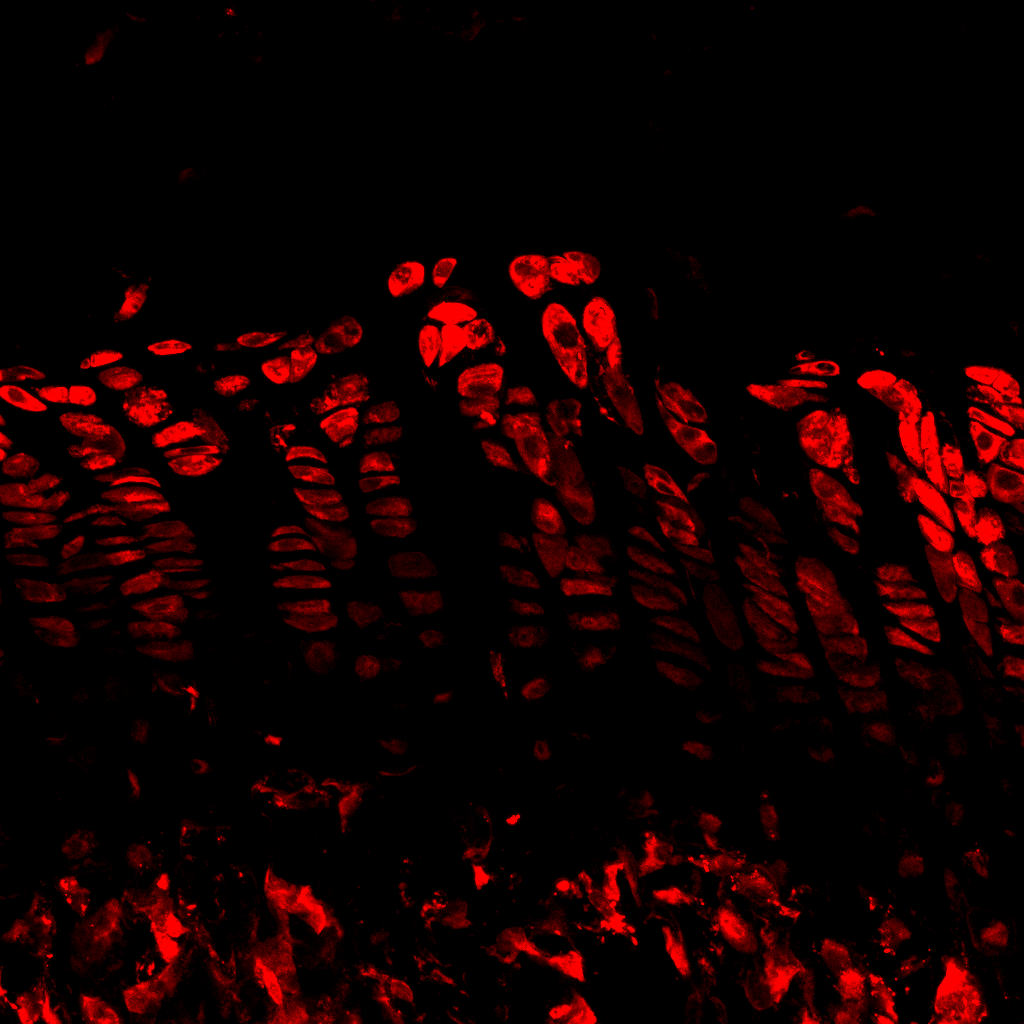

Supplement: Supplementary file 6 — Source Data Fig. 6 [file 44319_2024_93_MOESM6_ESM.zip › Figure6/6G/WT_td_red.tif]

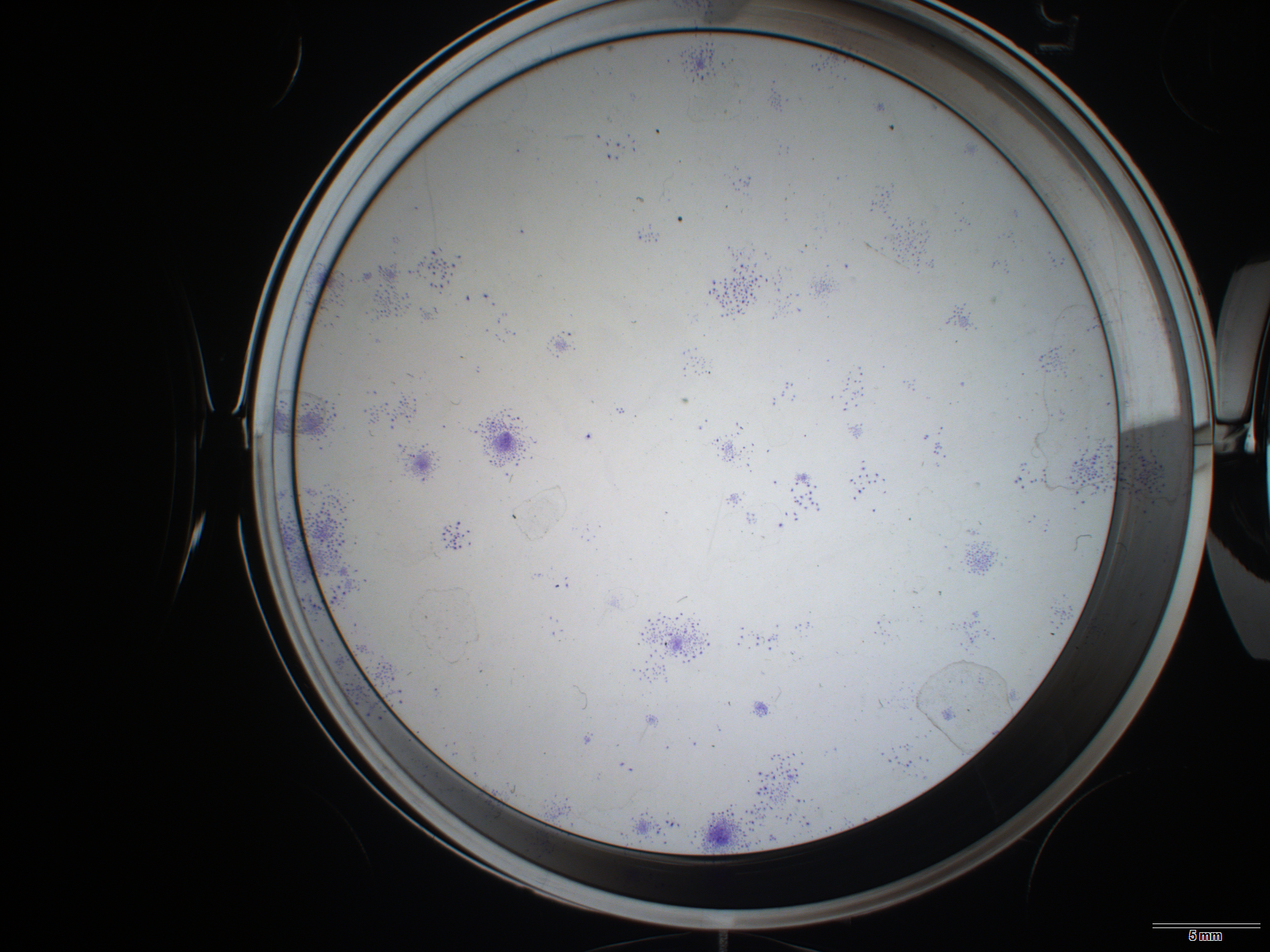

Supplement: Supplementary file 7 — Source Data Fig. 7 [file 44319_2024_93_MOESM7_ESM.zip › Figure7/7A/CKO-1.TIF]

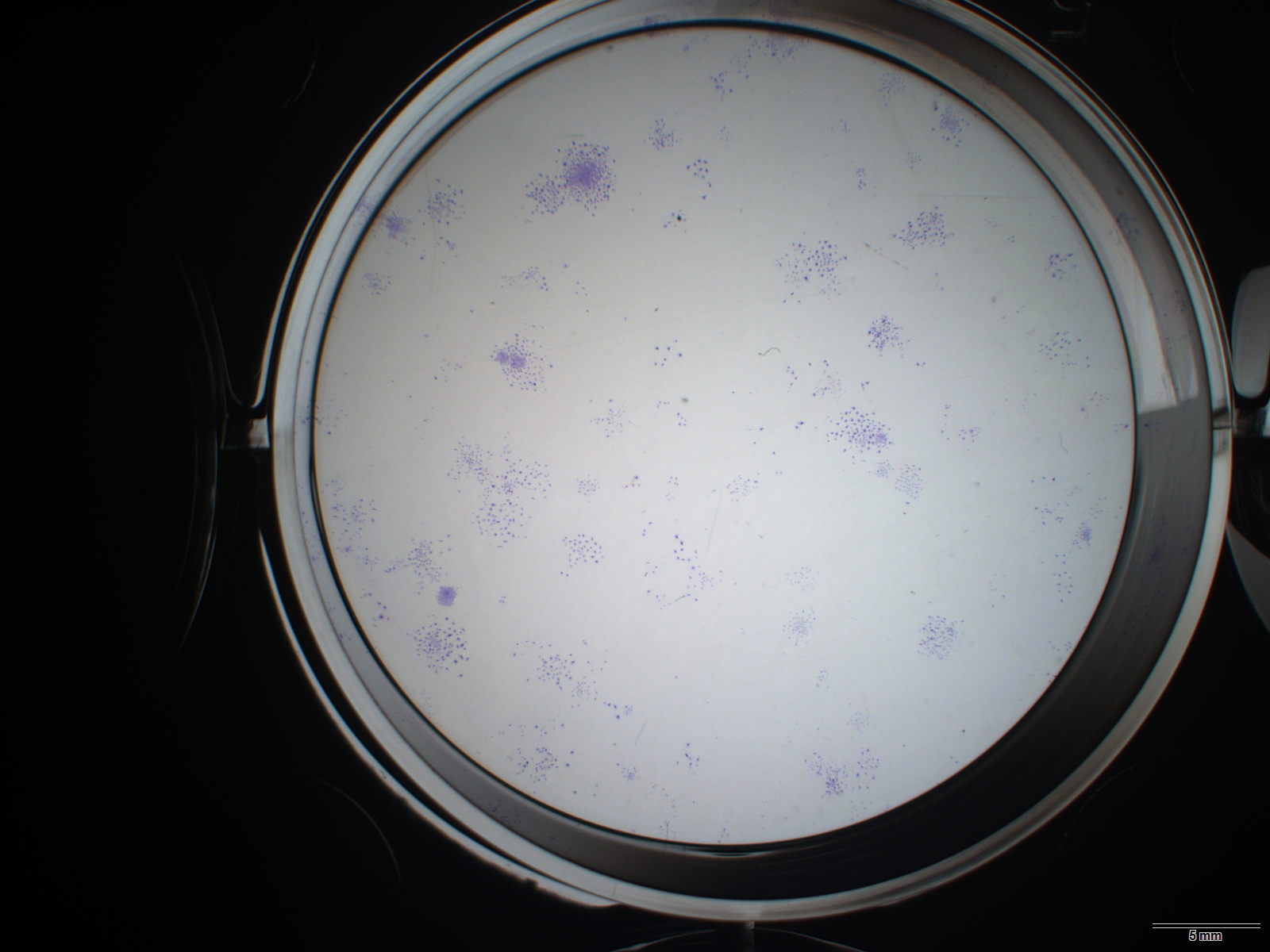

Supplement: Supplementary file 7 — Source Data Fig. 7 [file 44319_2024_93_MOESM7_ESM.zip › Figure7/7A/CKO-2.TIF]
