# Supplementary figures and images for: Gli1 labels progenitors during chondrogenesis in postnatal mice (part 3 of 3)
Source: EMBO Rep. 2024 Feb 26;25(4):12. doi: 10.1038/s44319-024-00093-x (PMC11014955; doi:10.1038/s44319-024-00093-x)

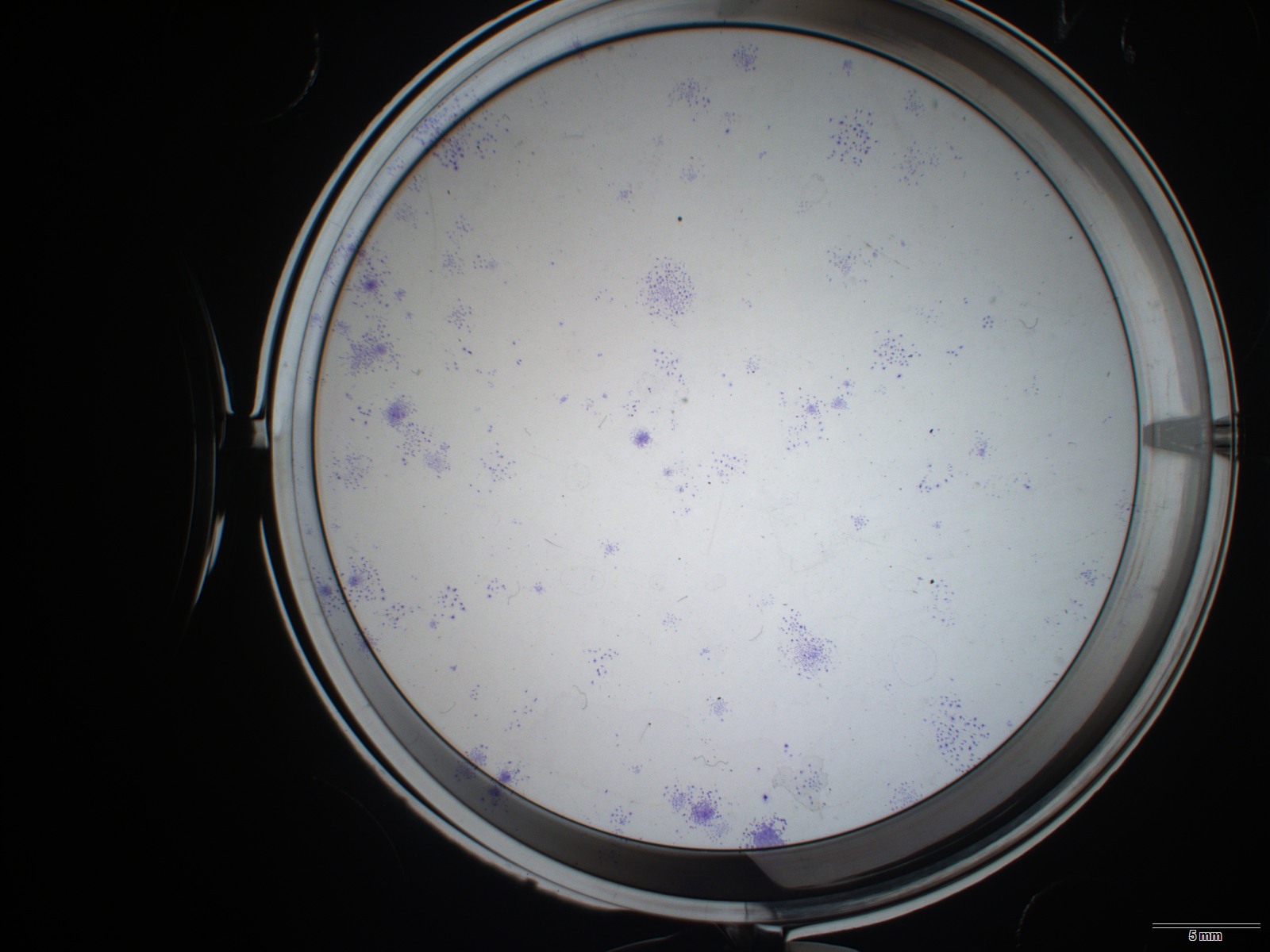

Supplement: Supplementary file 7 — Source Data Fig. 7 [file 44319_2024_93_MOESM7_ESM.zip › Figure7/7A/CKO-3.TIF]

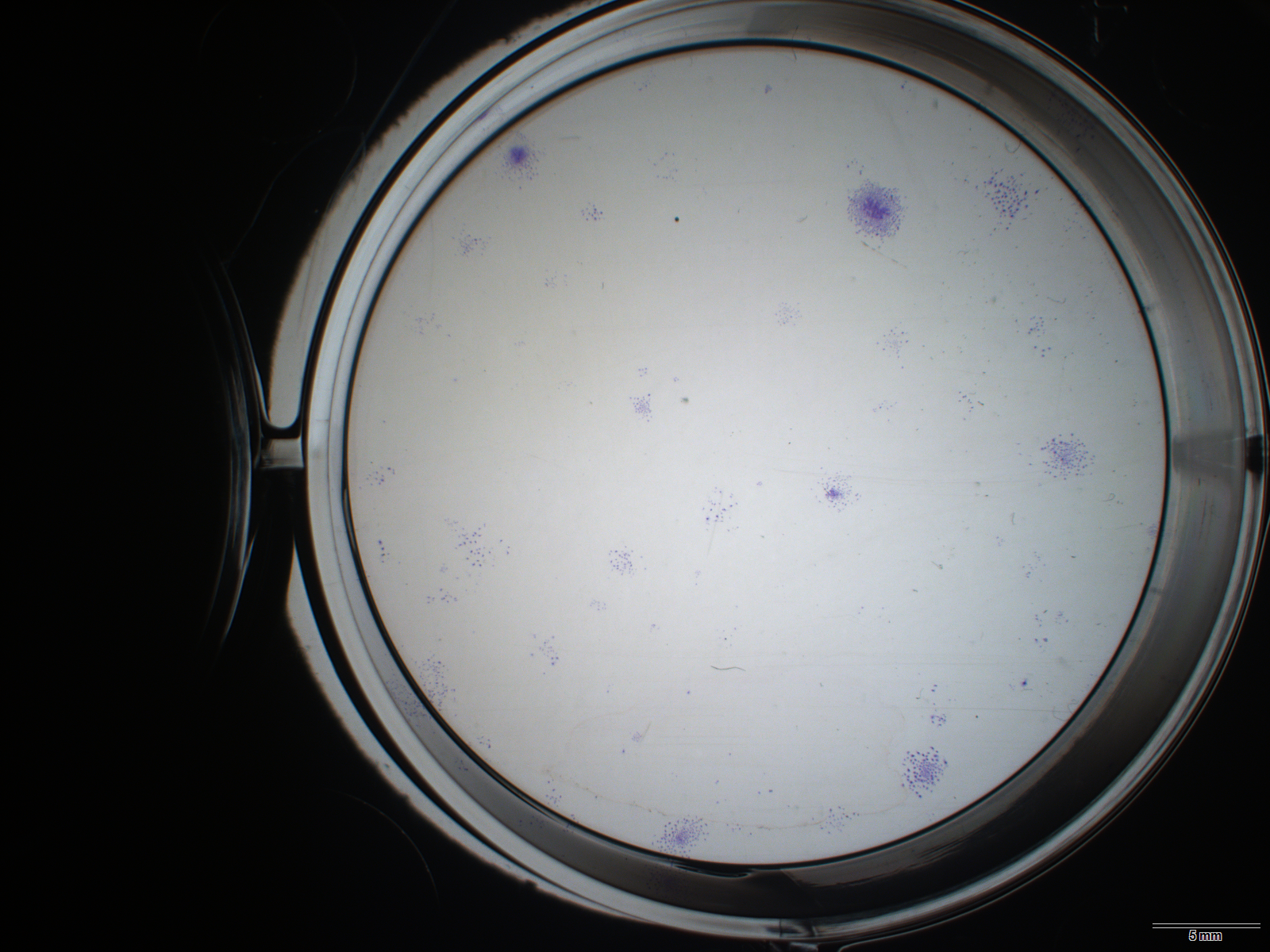

Supplement: Supplementary file 7 — Source Data Fig. 7 [file 44319_2024_93_MOESM7_ESM.zip › Figure7/7A/WT-1.TIF]

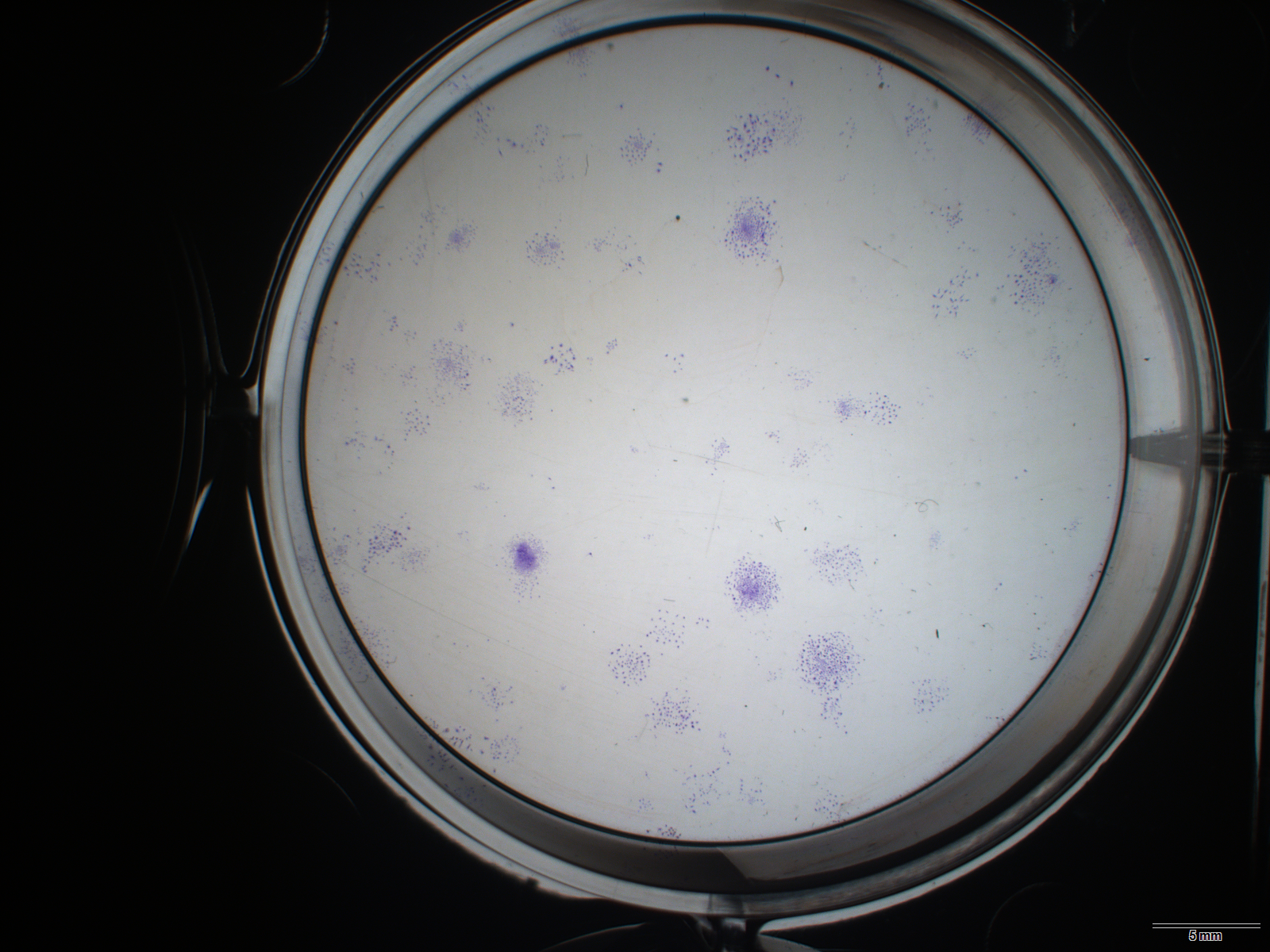

Supplement: Supplementary file 7 — Source Data Fig. 7 [file 44319_2024_93_MOESM7_ESM.zip › Figure7/7A/WT-2.TIF]

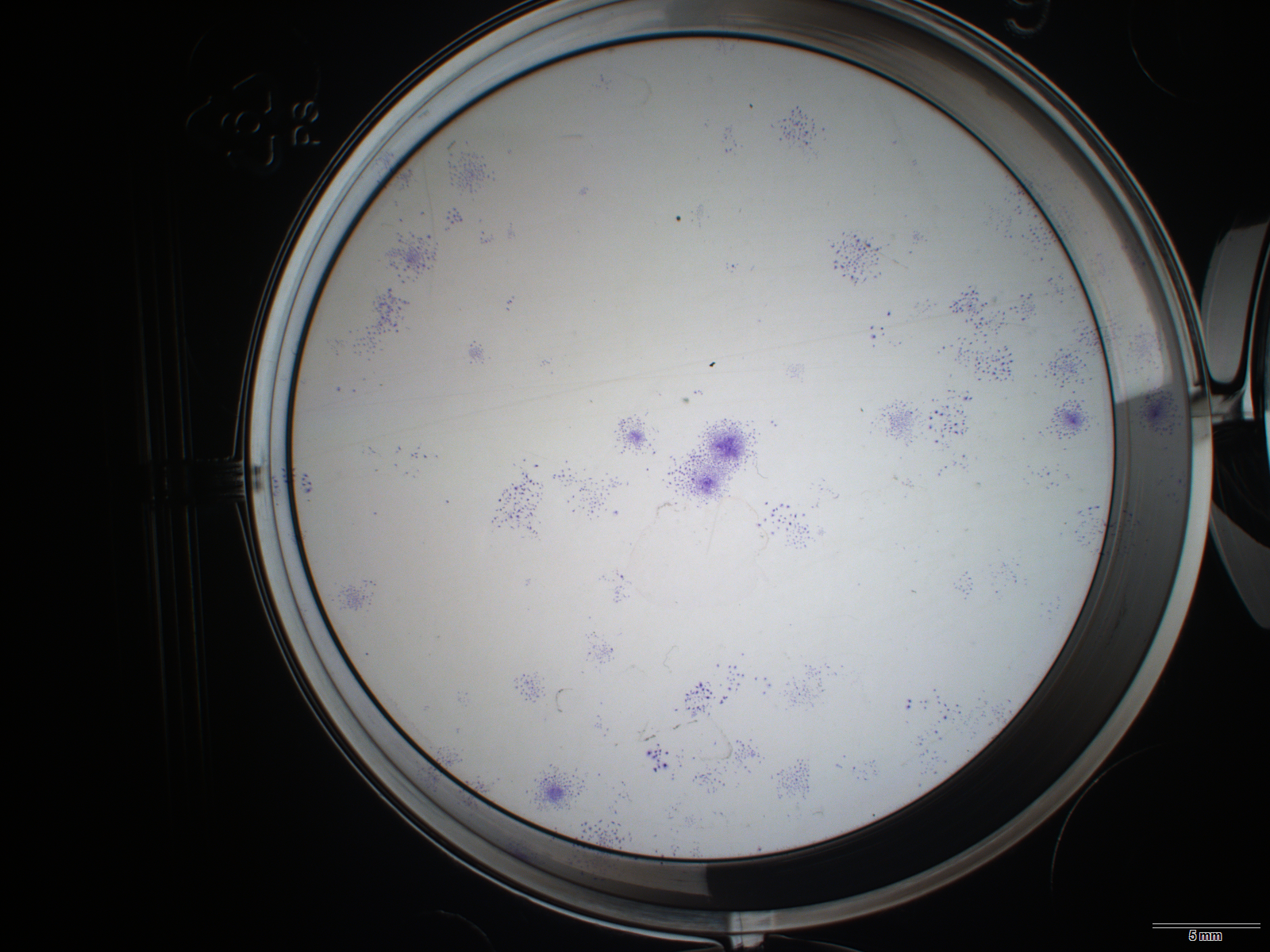

Supplement: Supplementary file 7 — Source Data Fig. 7 [file 44319_2024_93_MOESM7_ESM.zip › Figure7/7A/WT-3.TIF]

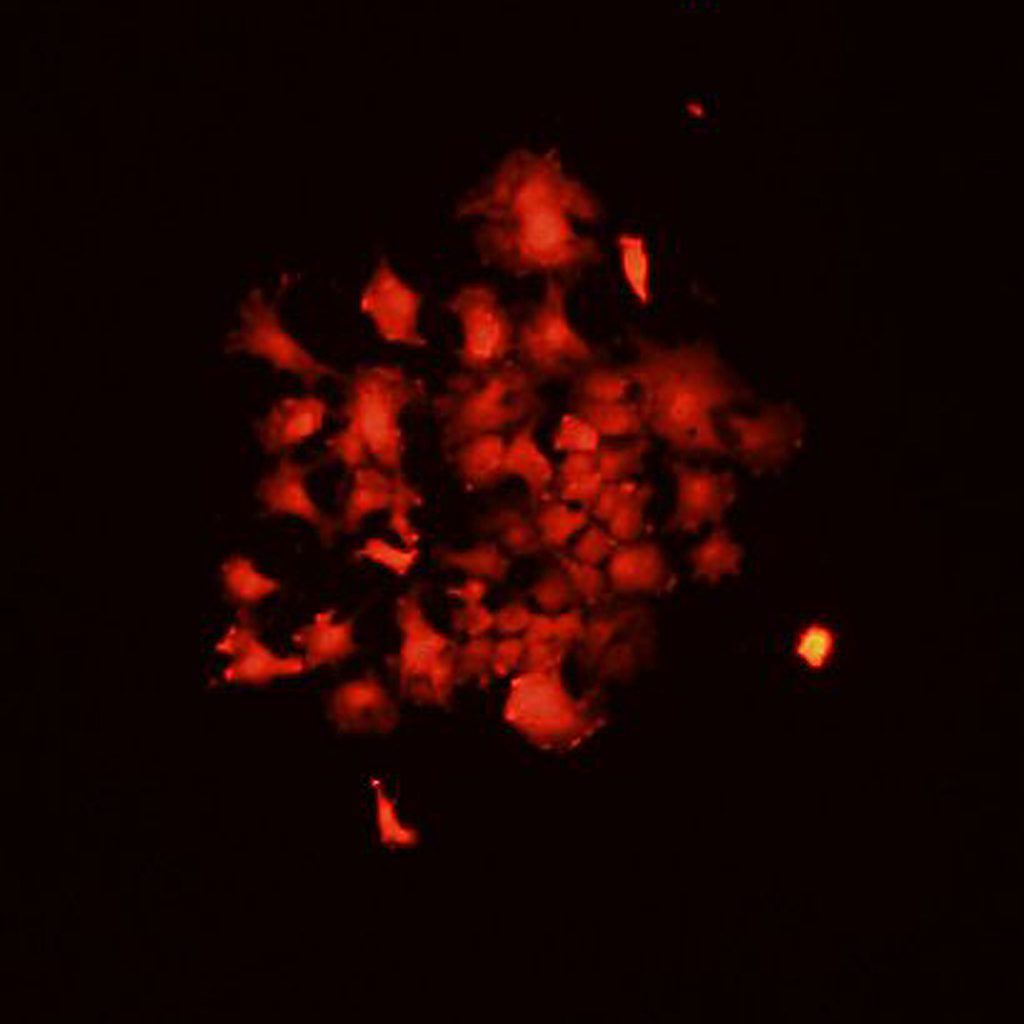

Supplement: Supplementary file 7 — Source Data Fig. 7 [file 44319_2024_93_MOESM7_ESM.zip › Figure7/7B/CKO.tif]

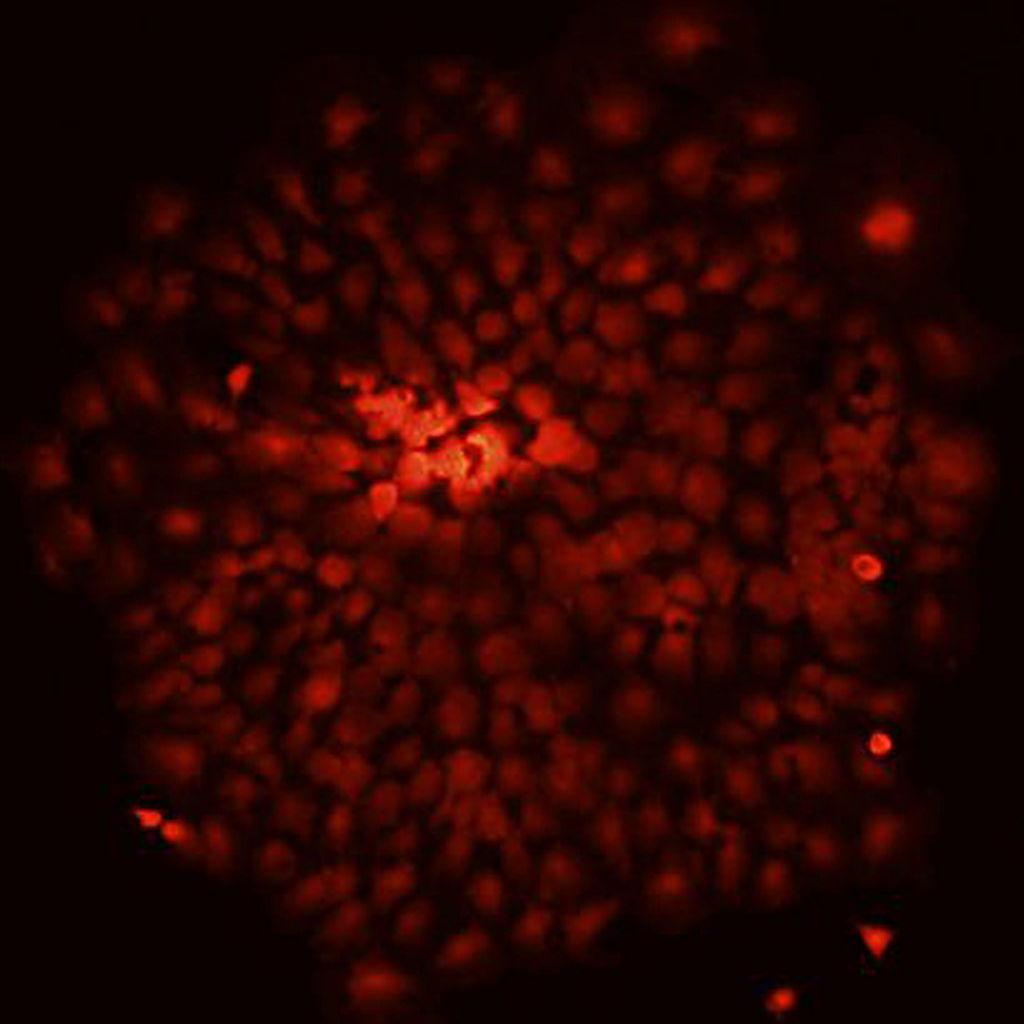

Supplement: Supplementary file 7 — Source Data Fig. 7 [file 44319_2024_93_MOESM7_ESM.zip › Figure7/7B/WT.tif]
