# Supplementary material for: Gli1 labels progenitors during chondrogenesis in postnatal mice
Source: EMBO Rep. 2024 Feb 26;25(4):12. doi: 10.1038/s44319-024-00093-x (PMC11014955; doi:10.1038/s44319-024-00093-x)
Supplement: Supplementary file 9 — Expanded View Figures [file 44319_2024_93_MOESM9_ESM.pdf]

Expanded View Figures

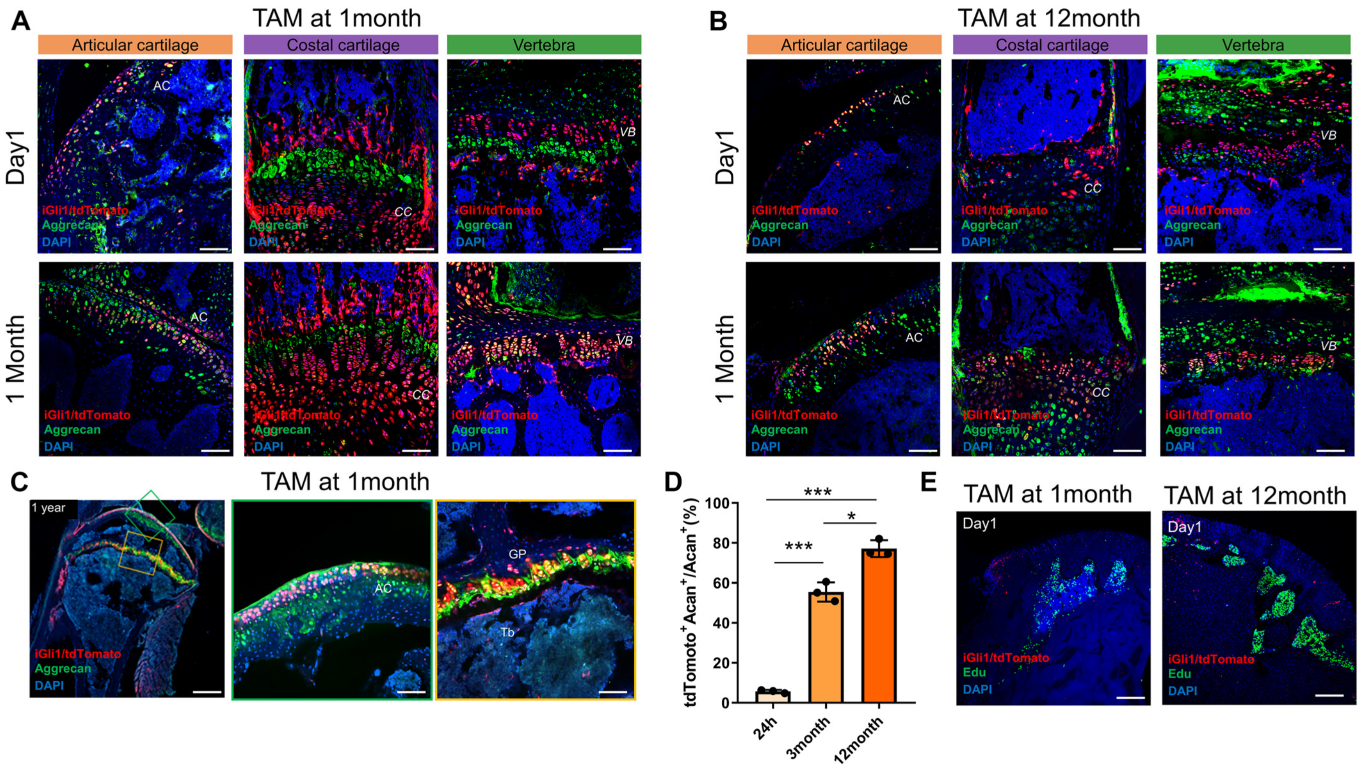

Figure EV1. (related to Fig. 1).

(A) *Gli1-CreER*^{T2}*; tdTomato* mice were administrated tamoxifen (TAM) at 1 month old and harvested after 1 day or 1 month. Representative confocal images from frozen sections of articular cartilage from tibia, costal cartilage and vertebrae. AC, articular cartilage; GP, growth plate; CC, costal cartilage; VB: vertebrae. Red: tdTomato; Green: aggrecan; Blue: DAPI. Scale bars = 100 μm. **(B)** *Gli1-CreER*^{T2}*; tdTomato* mice were administrated tamoxifen (TAM) at 12 months old and harvested after 1 day or 1 month. Representative confocal images from frozen sections of articular cartilage from tibia, costal cartilage and vertebrae. AC, articular cartilage; GP, growth plate; CC, costal cartilage; VB: vertebrae. Red: tdTomato; Green: aggrecan; Blue: DAPI. Scale bars = 100 μm. **(C)** *Gli1-CreER*^{T2}*; tdTomato* mice were administrated tamoxifen (TAM) at 1-month old and harvested after 12 months. Representative confocal images from frozen sections of the tibia. Boxed areas are shown at higher magnification in corresponding panels to the right. Green box, articular cartilage; Orange box, growth plate. GP, growth plate; Tb: trabecular bone. Red: tdTomato; Green: aggrecan; Blue: DAPI. bars = 100 μm. **(D)** The percentage of tdTomato$^+$ Acan$^+$ to Acan$^+$ cells was quantified. *Gli1-CreER*^{T2}*; tdTomato* mice were administrated tamoxifen (TAM) at 1 month old and harvested after 24 h, 3 months and 12 months, respectively. $n = 3$ mice per group, data are presented as mean ± s.d. Significance was determined using one-way ANOVA followed by Tukey's test. *$P < 0.05$, ***$P < 0.001$. **(E)** Representative confocal images of EdU staining of temporomandibular joint. Scale bars = 100 μm.

 © The Author(s)

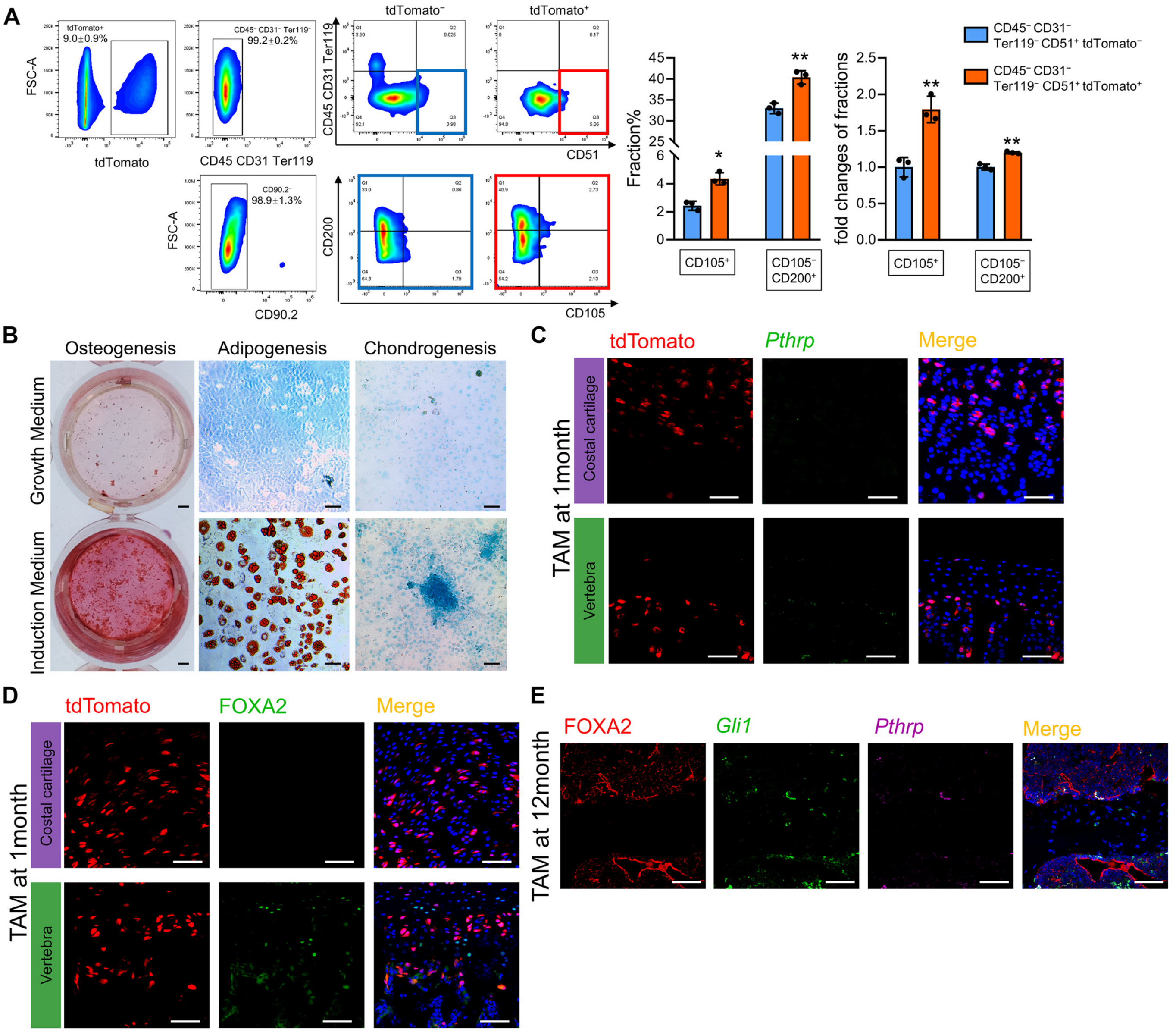

Figure EV2. (related to Fig. 2).

(**A**) Flow cytometry analysis of skeletal stem and progenitor cell-surface-marker in primary chondrogenic cells from growth plate. tdTomato$^-$, tdTomato$^-$ fraction of CD45$^-$CD31$^-$Ter119$^-$ cells; tdTomato$^+$, tdTomato$^+$ fraction of CD45$^-$CD31$^-$Ter119$^-$ cells. Blue box, CD45$^-$CD31$^-$Ter119$^-$CD51$^+$ tdTomato$^-$ fraction (Gli1$^-$). Red box, CD45$^-$CD31$^-$Ter119$^-$CD51$^+$ tdTomato$^+$ fraction (Gli1$^+$). The left bar graph showed the percentage of CD105$^-$CD200$^+$ and CD105$^+$ cells within Gli1$^-$ and Gli1$^+$ fractions. The right bar graph showed the fold change of Gli1$^+$ fractions compared with Gli1$^-$ fraction. $n = 3$ mice per group, data are presented as mean ± s.d. Significance was determined using unpaired *t*-tests. *$P < 0.05$, **$P < 0.01$. (**B**) Representative tri-lineage differentiation images of Gli1-CreER^{T2}–tdTomato$^+$ cells. Alizarin Red stain was used for osteogenesis; oil red stain was used for adipogenesis; toluidine blue stain was used for chondrogenesis. (**C**) Representative confocal images to monitor the expression of tdTomato, *Pthrp* in 1-month-old mice using RNAscope assay. (**D**) Representative confocal images of immunofluorescence staining of FOXA2 in costal cartilage and vertebrae from 1-month-old *Gli1-CreERT2; tdTomato* mice. (**E**) Representative confocal images to monitor the expression of *Gli1*, *Pthrp* and FOXA2 in the growth plate of proximal tibia from 12-month-old mice using RNAscope assay. Data Information: In (**B**), scale bars = 1 mm (Osteogenesis),100 μm (Adipogenesis, Chondrogenesis). In (**C–E**), scale bars = 25 μm.

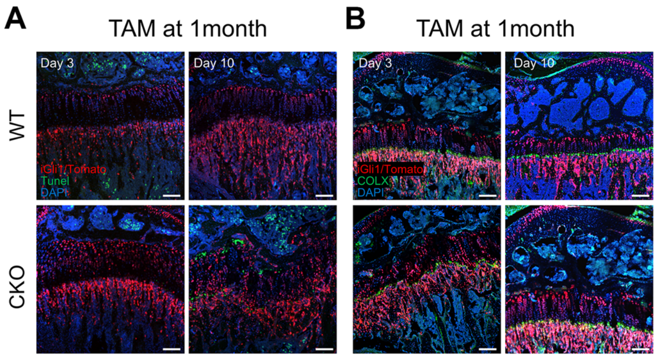

Figure EV3. (related to Fig. 5).

WT and BMPR1α CKO mice were administrated tamoxifen (TAM) at 1-month-old and harvested after 3 days or 10 days of chase respectively. (**A**) Representative images of TUNEL staining for apoptosis in growth plate region on indicated days. Green: TUNEL; Red: tdTomato; Blue: DAPI. (**B**) Immunofluorescence staining of COLX on frozen sections of growth plate on indicated days. Green: COLX. Scale bars = 100 µm.

 © The Author(s)

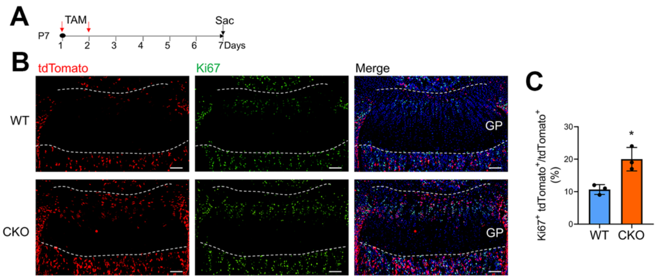

Figure EV4. (related to Fig. 6).

(**A**) The schematic graph of the chemical administration protocol. (**B**) Representative images of immunofluorescence staining of Ki67 on frozen sections of proximal tibia. Red: tdTomato; Green: Ki67; Blue: DAPI. The dashed line indicated the region of growth plate. Scale bars = 100 μm (**C**) The percentage of $Ki67^+$ $tdTomato^+$ cells in $tdTomato^+$ population in growth plate. $n = 3$ mice per group. Data are presented as mean ± s.d. Significance was determined using unpaired *t*-test (**C**). $^*P < 0.05$.
